# EPA Induces an Anti-Inflammatory Transcriptome in T Cells, Implicating a Triglyceride-Independent Pathway in Cardiovascular Risk Reduction

**DOI:** 10.1016/j.jacbts.2024.09.002

**Published:** 2024-10-30

**Authors:** Nathalie A. Reilly, Koen F. Dekkers, Jeroen Molenaar, Sinthuja Arumugam, Thomas B. Kuipers, Yavuz Ariyurek, Marten A. Hoeksema, J. Wouter Jukema, Bastiaan T. Heijmans

**Affiliations:** a Molecular Epidemiology, Department of Biomedical Data Sciences, Leiden University Medical Center, Leiden, the Netherlands; b Department of Cardiology, Leiden University Medical Center, Leiden, the Netherlands; c Sequencing Analysis Support Core, Department of Biomedical Data Sciences, Leiden University Medical Center, Leiden, the Netherlands; d Leiden Genome Technology Center, Department of Human Genetics, Leiden University Medical Center, the Netherlands; e Department of Medical Biochemistry, Amsterdam University Medical Center, University of Amsterdam, Amsterdam the Netherlands; f Netherlands Heart Institute, Utrecht, the Netherlands

**Keywords:** atherosclerosis, eicosapentaenoic acid, oleic acid, palmitic acid, T cells, transcriptomics

## Abstract

Twice-daily intake of purified eicosapentaenoic acid (EPA) reduces atherosclerotic cardiovascular disease risk in patients with high triglycerides, but its exact mechanism remains unclear. We exposed non-activated CD4^+^ T cells to 100μM EPA, oleic acid, palmitic acid, or control, and conducted RNA and ATAC-sequencing after 48 hours. EPA exposure downregulated immune response-related genes like *HLA-DRA*, *CD69*, and *IL2RA*, and upregulated oxidative stress prevention genes like *NQO1*. Transcription factor footprinting showed decreased GATA3 and PU.1, and increased REV-ERB. These effects were specific to EPA, suggesting it induces an anti-inflammatory transcriptomic landscape in CD4^+^ T cells, contributing to its observed cardiovascular benefits.

## Highlights

• The mechanism by which EPA reduces the risk of ASCVD in clinical trials remains unclear.
• EPA can induce an anti-inflammatory transcriptomic landscape in nonactivated CD4^+^ T cells in vitro.
• T cell reactions to palmitic and oleic acid reveal EPA’s unique anti-inflammatory transcriptomic response.
• Examining T cells during IPE interventions may reveal insights into EPA’s benefits independent of triglyceride reduction.

## Visual Abstract

**Figure:**
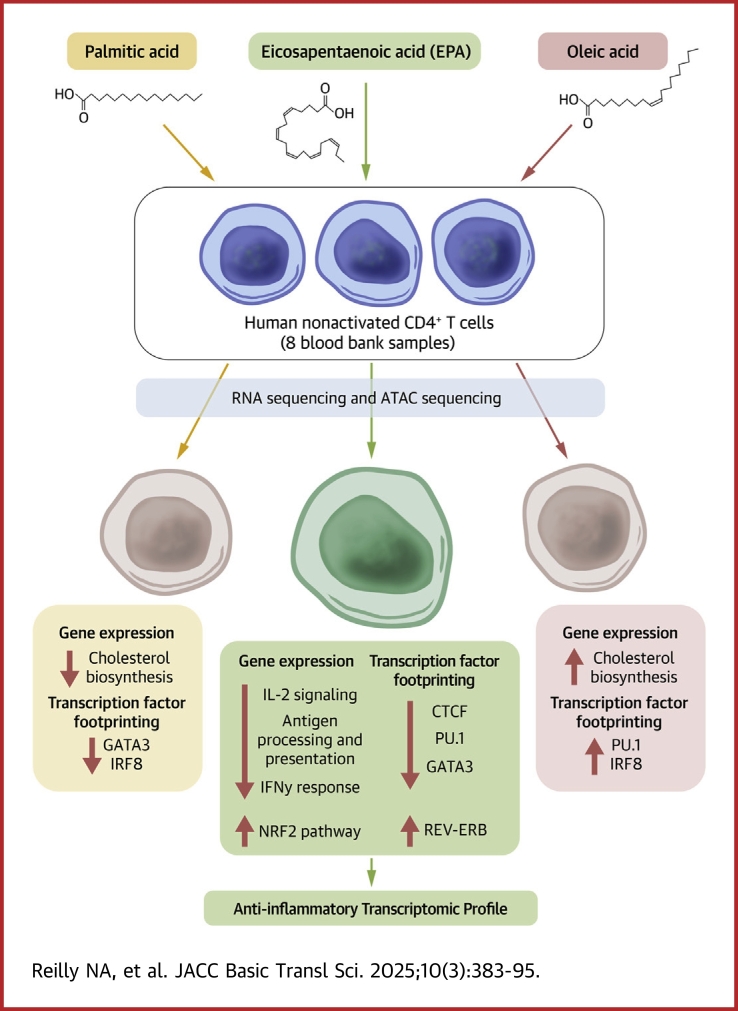

The risk of atherosclerotic cardiovascular disease (ASCVD) persists despite therapies that effectively control blood cholesterol levels, including statins and PCSK9 inhibitors.^1^, ^2^, ^3^ This residual risk has been attributed, in part, to elevated triglyceride levels in blood.^4^ Nevertheless, most triglyceride-influencing therapies, such as fibrates or niacin, have little cardiovascular benefit.^5^, ^6^, ^7^, ^8^, ^9^ However, there is one triglyceride-lowering drug that was found to strongly reduce ASCVD risk, namely, icosapent ethyl (IPE), which in the body is metabolized to eicosapentaenoic acid (EPA), a polyunsaturated fatty acid. The REDUCE-IT trial (A Study of AMR101 to Evaluate Its Ability to Reduce Cardiovascular Events in High-Risk Patients With Hypertriglyceridemia and on Statin; NCT01492361) showed that 4 g IPE administered as 2 g twice daily was superior to placebo in reducing triglycerides, cardiovascular events, and cardiovascular death among patients with high triglycerides and either known cardiovascular disease or at high risk for developing it, and who were already on statin therapy with relatively well controlled low-density lipoprotein (LDL) levels.^10^ The results of the trial and its interpretation has been much debated in literature.^11^^,^^12^ In particular, it remains largely unknown how EPA exerts its beneficial effects, and only limited studies have been carried out in model membranes or by examining whole blood.^13^, ^14^, ^15^

Atherosclerosis is regarded as a lipid-driven immune disease.^16^ As such, the majority of immune cells in the atherosclerotic plaque are T cells, of which half are CD4^+^.^17^^,^^18^ Furthermore, CD4^+^ T cells aggravate atherosclerosis in established mouse models.^19^^,^^20^ Therefore, the study of CD4^+^ T cells is a promising route to further understanding ASCVD, and investigating how EPA can influence these cells can indicate a potential mechanism underlying the beneficial effects of EPA on atherosclerosis. Interestingly, EPA was suggested to have anti-inflammatory properties, as indicated by a reduction in CD4^+^ T cell proliferation, decreased differentiation toward T helper (T_H_) 1 and T_H_17, and increased or no effect on differentiation toward T_H_2 and T regulatory (T_reg_) cells.^21^ However, these studies were largely carried out in mouse models or in vitro during T cell activation, under polarizing conditions, or by measuring general T cell markers.^22^, ^23^, ^24^, ^25^, ^26^, ^27^, ^28^ Thus, the effects of EPA on T cells remain incompletely understood and, in particular, it is unknown whether EPA can affect human CD4^+^ T cells in a nonactivated state, as they occur in the circulation and where the primary interaction with EPA takes place.

We aimed to further elucidate the effects of EPA on CD4^+^ T cells by performing transcriptomic analysis on nonactivated exposed cells. Furthermore, we assessed the specificity of the effects of EPA by exposing cells to 2 other fatty acids of different saturation, oleic acid (OA), a monounsaturated fatty acid, and palmitic acid (PA), a saturated fatty acid. To do so, we performed RNA and ATAC sequencing on nonactivated CD4^+^ T cells after 48 hours of exposure to EPA, OA, PA, or control. We show that EPA leads to a marked down-regulation of many anti-inflammatory genes in nonactivated CD4^+^ T cells compared with control. The pronounced and specific effects on the transcriptomics landscape contrasted with the relatively modest effects of OA and PA.

## Methods

### Peripheral blood CD4^+^ T cell isolation and culture conditions

CD4^+^ T cell isolation and fatty acid exposure model were based on our previously described in vitro model, with minor changes.^29^ To obtain nonactivated CD4^+^ T cells, peripheral blood mononuclear cells (PBMCs) were isolated from 8 different buffy coats of anonymous blood bank donors (Sanquin) by means of Ficoll Paque (Apotheek LUMC, 97902861) gradient centrifugation. All donors provided written informed consent in accordance with the protocol of the local institutional review board and the Medical Ethics committee of Sanquin blood supply in accordance with the Declaration of Helsinki. The sex of the cells could not be determined owing to the anonymity of the donors. However, RNA sequencing (RNA-seq) showed that, of 8 donors sequenced, 6 were female and 2 were male, which was accounted for during the statistical analysis by correcting for donor effect. Next, CD4^+^ T cells were purified from the PBMCs with the use of lyophilized human anti-CD4^+^ magnetically labeled microbeads (Miltenyi; 130-097-048) scaling the manufacturer’s instructions to one-third of the recommended volumes. CD4^+^ T cell purity was assessed on an LSR-II instrument at the Leiden University Medical Center Flow Cytometry Core Facility with the use of BD FACSDiva v9.0 software (BD Biosciences). Cells were stained with anti-CD3-PE (BD Biosciences; 345765), anti-CD4-APC (BD Biosciences; 345771), anti-CD8-FITC (BD Biosciences; 555634), and anti-CD14-PEcy7 (BD Biosciences; 560919) and resuspended in 1% paraformaldehyde (Apotheek LUMC; 120810-001) to fix the cells before acquisition. Purity was >98% for all donors.

Before fatty acid exposure, ∼1 × 10^8^ isolated cells were cultured overnight to allow the cells to return to a resting state after the stress of the isolation procedure. This was done in T75 flasks (Greiner Bio-One; 658-175) at a density of ∼2.5 × 10^6^ cells/mL in 5% fetal calf serum (FCS) (Bodinco BDC; 16941) Dulbecco Modified Eagle’s Serum (DMEM; Sigma; 05796), 1% Pen-Strep (Lonza; DE17-602E), and 1% Glutamax-1 (100×) (Gibco; 35050-038)) medium supplemented with 50 IU/mL interleukin-2 (IL-2; Peprotech; 200-02) and incubated at 37 °C under 5% CO_2_. To keep the cells in a nonactivated state, no additional stimulus was added. Any CD4^+^ T cells not used directly after the isolation were kept in DMEM supplemented with 30% FCS, 1% Pen-Strep, 1% Glutamax-1, and 20% dimethyl sulfoxide (WAK-Chemie Medical; WAK-DMSO-10) medium at a density of ∼25 × 10^6^ cells/mL and stored in liquid nitrogen.

**Figure 1:**
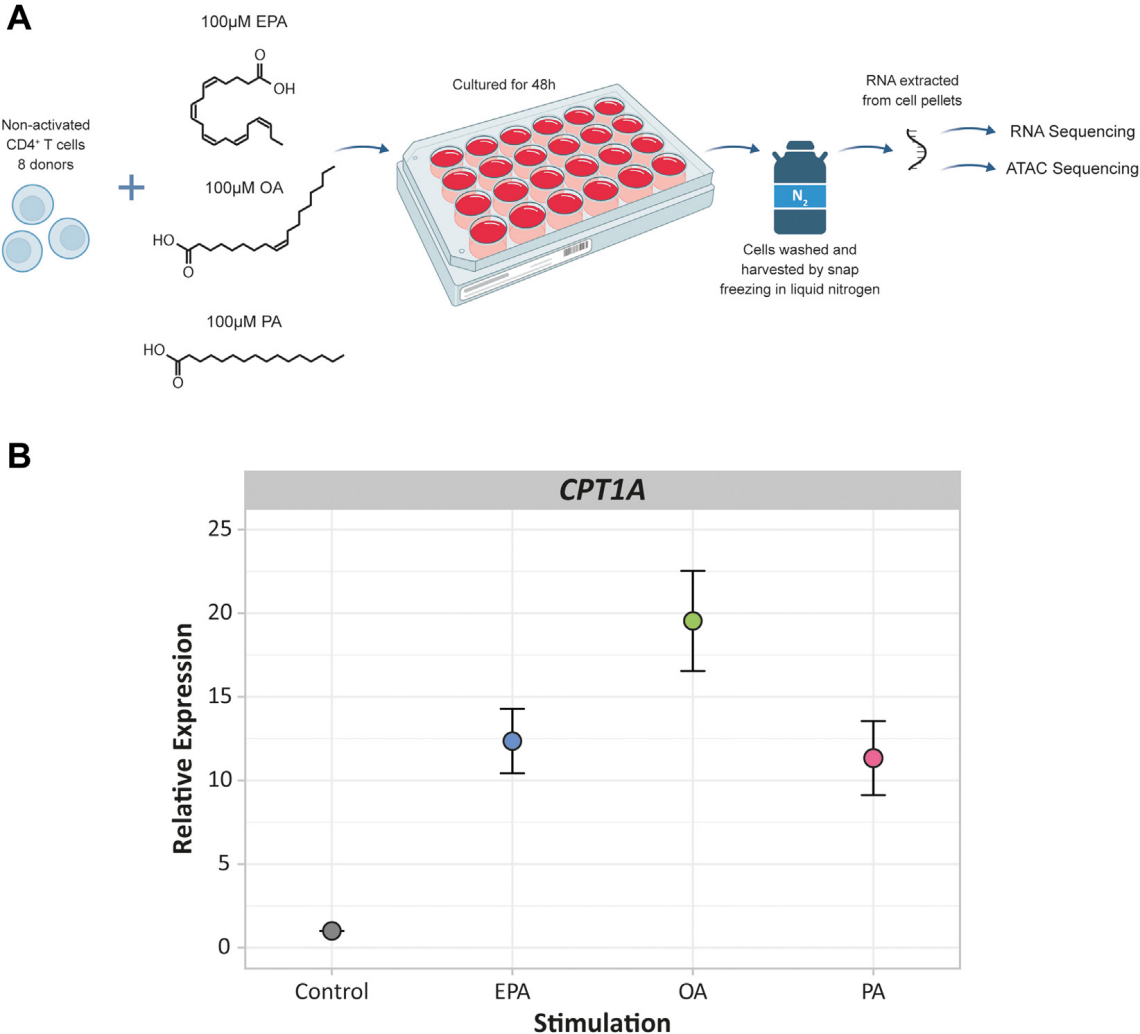
Experimental Set-Up and In Vitro Model Verification (A) Experimental set-up for RNA and ATAC sequencing of EPA-, OA-, and PA-exposed nonactivated CD4^+^ T cells; n = 8. (B) Dot plot showing the relative expression of *CPT1A* after 48 hours of fatty acid exposure as a confirmation of the in vitro model by means of real-time quantitative polymerase chain reaction. Values are colored by fatty acid. The mean fold change (± SEM) demonstrated that *CPT1A* was up-regulated for EPA (12.4 ± 1.9; *P* < 0.001), OA (19.5 ± 3.0; *P* < 0.001), and PA (11.3 ± 2.2; *P* < 0.003); n = 8. EPA = eicosapentaenoic acid; OA = oleic acid; PA = palmitic acid.

Next, nonactivated CD4^+^ T cells were cultured with either EPA (Cayman; 90110), OA (Sigma; O1383), PA (Cayman; 10006627), or control for 48 hours at 37 °C under 5% CO_2_. The cells were exposed for 48 hours based on previous findings when establishing our previously described in vitro model.^29^ To this end, CD4^+^ T cells from each donor were plated in a 24-well plate (density of ∼3.5 × 10^6^ cells/well) in 2 mL 5% FCS DMEM for each condition (Figure 1A). Cells were cultured in medium containing FCS to ensure cell viability during culture and to be more similar to physiologic conditions of the circulation, where other lipids are present. To assess the additional EPA, OA, or PA stimulus to the nonactivated CD4^+^ T cells due to FCS in the culture medium, an FCS sample was measured via the Shotgun Lipidomics Assistant method^30^ to estimate the fraction of fatty acids in the sample. The sample was prepped as previously described^31^ but with 2 modifications: a starting volume of 25 μL FCS and addition of 600 μL methyl *tert*-butyl ether instead of 575 μL during the first extraction. Free EPA was 0.02 μg/mL, and EPA as component of larger molecules, including cholesterol esters and sphingolipids, was 0.13 μg/mL. Free OA was 0.29 μg/mL, and OA as component of larger molecules, including cholesterol esters and sphingolipids, was 4.93 μg/mL. Free PA was 0.23 μg/mL, and PA as component of larger molecules, including cholesterol esters and sphingolipids, was 3.45 μg/mL.

PA was dissolved in high-performance liquid chromatography (HPLC)–grade ethanol (Fisher Scientific; 64-17-5) to a final concentration of 5 mg/mL to create a stock solution. The stock solution was vortexed briefly, sonicated in a sonicator (Branson; 2800) for 15 minutes, and heated for 15 minutes at 45 °C. A small portion of the stock was extracted into a glass HPLC vial (Agilent Technologies; 5182-0714) to a final concentration of 5,000 μg/mL. EPA and OA were dissolved from their stock in HPLC-grade ethanol to final concentrations of 25,000 and 30,000 μg/mL, respectively. The HPLC-grade ethanol was then evaporated before the fatty acids were complexed with fatty acid–free (FAF) bovine serum albumin (BSA) (Sigma; A7030) in a 2% FAF BSA DMEM mixture (2% FAF BSA, 1% Pen-Strep, 1% Glutamax-1 [100×]) to a concentration of 151.25 μg/mL for EPA, 141.25 μg/mL for OA, and 128.2 μg/mL for PA. Complexing fatty acids with BSA mimics physiologic conditions, because fatty acids are also bound to albumin in the human circulation.^32^ Each fatty acid was further diluted to the final concentrations of 100 μmol/L (30.25 μg/mL for EPA, 28.25 μg/mL for OA, and 25.64 μg/mL for PA) on addition to the cells. The concentration tested was kept equal to ensure that the cells were exposed to the same amount of fatty acid particles and not influenced by concentration differences. Fatty acid stocks were stored under argon gas at −20 °C to avoid oxidation.

As a control, HPLC-grade ethanol was evaporated in a glass HPLC vial before adding 2% FAF BSA DMEM medium and added to the cells. The amount of 2% FAF BSA DMEM added to the wells was equal for each condition to keep the volumes equivalent. The CD4^+^ T cells were cultured for 48 hours at 37 °C under 5% CO_2_. After exposure, the cells were washed and 1 × 10^5^ cells were used directly for ATAC sequencing preparation. Cells from the same donors for which ATAC sequencing was performed were later thawed from liquid nitrogen and exposed to the fatty acids as described previously. After 48 hours of exposure, the cells were washed and 3 × 10^6^ cells were flash frozen in liquid nitrogen and stored at −80 °C for RNA isolations. Cell viability and diameter were measured with the use of Via1-Cassette (Chemometec; 941-0012) on a NucleoCounter NC-200 (Chemometec; 900-0200) and found to be, respectively, >95% and on average 9 μm for each condition.

### RNA isolation

To isolate total RNA for RNA-seq and real-time (RT) quantitative polymerase chain reaction (qPCR), RNA was extracted from the cell samples with the use of the Quick-RNA Microprep Kit (Zymo; R1050) according to the manufacturer’s instructions. The RNA was quantified with the use of a Qubit 2.0 Fluorometer (Q32866) with the Qubit RNA BR Assay Kit (Thermofisher; Q10211) according to the manufacturer’s instructions. The RNA was placed over a second Zymo-Spin IC Column, washed, and a second DNase treatment performed to remove any residual DNA contamination from the samples. RNA integrity values of the samples were on average 7.8 ± SE 0.1 as determined with the use of an Agilent 2100 Bioanalyzer Instrument (G2939BA) and the Agilent RNA 6000 Nano Reagents (Agilent; 5067-1511). RNA was divided into 2 samples and stored at −80 °C, 1 μg for RNA-seq and the rest for cDNA synthesis and RT-qPCR measurements.

### Real-time qPCR

To measure the expression of *CPT1A* in all the cell samples, cDNA was synthesized with 200 ng of the stored RNA with the use of the Transcriptor First Strand cDNA Synthesis Kit (Roche; 04897030001) according to the manufacturer’s instructions. RT-qPCR for *CPT1A* (Thermofisher; Hs00912671_m1; 4331182) was performed with the TaqMan Fast Advanced Master Mix (Thermofisher; 4444557) with 10 ng cDNA per reaction on a QuantStudio 6 Real-Time PCR system (Applied Biosystems). All RT-qPCR reactions were performed in triplicate, and outliers were removed if the measured Ct value differed more than 0.5% from the mean. Relative gene expression levels (−ΔCt) were calculated using the average of Ct values of *RPL13A* (Thermofisher; Hs03043887_gH; 4448892) and *SDHA* (Thermofisher; Hs00188166_m1; 4453320) as internal control samples.^33^ The fold change was determined according to the 2^−ΔΔCt^ method, using the control as the reference.

### RNA-seq data generation and processing

RNA-seq was performed to determine the differences in the transcriptome of control vs fatty acid–exposed non-activated CD4^+^ T cells across time. The RNA from each of the samples was sent for sequencing (Macrogen). RNA-seq libraries were prepared from 200 ng RNA with the use of the Illumina Truseq stranded mRNA library prep (Illumina; 20020594) with a poly A selection. Both whole-transcriptome amplification and sequencing library preparations were performed in 2 96-well plates with 26 samples in one plate and 6 in another. Quality-control steps were included to determine total RNA quality and quantity, the optimal number of PCR preamplification cycles, and fragment size selection. No samples were eliminated from further downstream steps. Barcoded libraries were divided across 2 plates with 26 samples in one and 6 in the other and sequenced separately. Barcoded libraries were sequenced to a read depth of 20 million reads with the Novaseq 6000 (Illumina) to generate 100 basepair paired-end reads.

FastQ files are analyzed with the use of the RNAseq pipeline (v5.0.0) from BioWDL (zenodo.org/record/5109461), developed by SASC (LUMC). The pipeline performed preprocessing on the FastQ files (including quality control, quality trimming, and adapter clipping), read mapping, and expression quantification. *FastQC* (v0.11.9) is used to check raw reads and *Cutadapt* (v2.10) to perform adapter clipping. Reads are mapped to a reference genome (Ensembl v105) with the use of *STAR aligner* (v2.7.5a), and with *HTSeq Count* (v0.12.4) the number of assigned reads to genes per sample is determined. Based on Ensembl gene biotype annotation, we included only protein-coding genes for further downstream analysis (19,991 genes in total).

### ATAC sequencing analysis

After exposure, the 1 × 10^5^ cells were taken for ATAC sequencing and placed into DNA LoBind 1.5-mL tubes (Eppendorf; 2231000945). The cells were washed 3× in ice-cold phosphate-buffered saline solution (PBS)pH 7.4; Fresen; 15360679). The samples were then sent to the Leiden Genome Technology Center for library generation. The ATAC sequencing libraries were generated according to the Omni-ATAC protocol.^34^ Briefly, the nuclei were isolated by lysing the cells in ATAC Resuspension Buffer (RSB) (0.1% NP40 [Thermofisher; 85124], 0.1% Tween-20 [Thermofisher; 28320], and 0.01% digitonin [Promega; G9441]) for 3 minutes on ice. After washing the nuclei with 1 mL wash buffer (RSB and 0.1% Tween), the nuclei were centrifuged for 10 minutes at 4 °C. After removing the supernatant, carefully avoiding the pelleted nuclei, the nuclei were resuspended in PBS. The nuclei were counted and normalized to 25,000 cells with the use of the TC20 cell counter (BioRad; 1450102). The nuclei were combined with 25 μL 2× TD buffer (TrisHCl pH 7.5 [Thermofisher; 15567027], NaCl [Thermofisher; A57006], and MgCl_2_ [Thermofisher; AM9530G]), 2 μL Tn5 enzyme (Tn5 enzyme [Illumina; 15027865] and TD Tagment DNA Buffer [Illumina; 15027866]), 0.5 μL 1% digitonin, and 0.5 μL 10% Tween-20 up to a volume of 50 μL. The reaction was incubated at 37 °C for 30 minutes and then purified with the use of AMPure Beads (Beckman Coulter; A63881) with a ratio of 1.8× and eluted in 10 μL elution buffer (10 mmol/L Tris-HCl). The PCR was done with 2× Kapa HiFi Master Mix (Roche; 09420398001) and the barcoded primers described in the Omni-ATAC protocol. After the PCR, the products were dual size selected with the use of AMpure beads, first using 0.4×, followed directly by 1.2×. The ATAC sequencing libraries were checked on the Femto Pulse (Agilent; M5330AA) and pooled equimolarly for sequencing. No samples were eliminated from further downstream steps.

Barcoded libraries were sent out for sequencing (Macrogen). An additional round of quality control was performed, and the samples were then pooled and divided across 1 lane. Barcoded libraries were sequenced to a read depth of 30 million 150 basepair paired-end reads with Novaseq 6000 (Illumina).

FastQ files were analyzed with the use of the ChIP-seq pipeline from BioWDL, developed by SASC (LUMC). The pipeline performed preprocessing on the FastQ files (including quality control, quality trimming, and adapter clipping), read mapping, and peak calling. *FastQC* (v0.11.9) is used to check raw reads and *Cutadapt* (v2.10) to perform adapter clipping. Reads are mapped to a reference genome (Encode GRCh38) with the use of *BWA aligner* (v0.7.17), and *MACS2* (v2.1.2) is used to perform the peak calling. These peak files were then processed with the use of R (v4.3.0). Using *DiffBind* (v3.10.0), reads in the BAM files were counted for each peak. Next, the read counts per peak for each sample were merged to create 1 table containing all peaks and read counts of all the samples combined. De novo motif analysis was then performed with the use of HOMER.^35^

### Statistical analysis

All statistical analyses of both the qPCR data and the RNA-seq data were performed in R (v4.3.1).^36^ For qPCR, fold change data are expressed as the mean ± SEM. Change was evaluated using a paired 2-tailed Student’s *t*-test. *P* values <0.05 were considered to be statistically significant.

For the RNA-seq, we used the Bioconductor package *DESeq2*^37^ (v1.40.2) to test whether EPA, OA, or PA had an effect on gene expression compared with control. *DESeq2* fits a generalized linear model assuming the negative binomial distribution for the counts. The model expresses the logarithm of the average of the counts in terms of one or more predictors. In this case, we used 3 models that had each one of the fatty acids, subject identifier, and batch as predictors. By including the subject identifier and batch in the models, we account for the dependence between measurements within the same subject and between different batches of sequencing.^37^ Lowly expressed genes, ie, that did not have at least a count of 1 in half of the samples per fatty acid and control, were removed, resulting in 12,938 genes for EPA, 12,949 genes for OA, and 12,971 genes for PA. The Benjamini-Hochberg procedure was used to correct for multiple testing at a false discovery rate (FDR) of 5%.

Differentially expressed genes per fatty acid were divided into up-regulated or down-regulated based on the log2 fold change values. Ten human pathway databases (BioPlanet 2019, WikiPathways 2019 Human, KEGG 2019 Human, Elsevier Pathway Collection, BioCarta 2015, Reactome 2016, HumanCyc 2016, NCI-Nature 2016, Panther 2016, and MSigDB Hallmark 2020) were queried with the use of gene symbols, with 904 of 1,170 queried genes for EPA, 51 of 60 queried genes for OA, and 26 of 33 queried genes for PA, present in at least 1 database. The identified clusters were then mapped for pathway enrichment with the use of *clusterProfiler*^38^ (v4.8.3) with the background set to the 12,938 expressed genes for EPA, 12,949 expressed genes for OA, and 12,971 expressed genes for PA as determined above. Multiple testing correction with the Benjamini-Hochberg method at 5% FDR was performed over the combined results from the 10 databases. Pathways that included highly similar gene sets were grouped (Jaccard index >0.7), and only the most significantly enriched pathway per group was retained.

### Data availability

The data supporting the findings of this study are available within the paper and its supplemental files. All other data, including the raw files, are available at the Gene Expression Omnibus repository, accession GEO (main combined submission: GSE254749; RNA sequencing submission: GSE254695; and ATAC sequencing submission: GSE254468).

## Results

### Transcriptomic analysis of EPA exposed nonactivated CD4^+^ T cells

Nonactivated CD4^+^ T cells from 8 different donors were each exposed to 100 μmol/L EPA, OA, PA, or control for 48 hours (Figure 1A). Exposure did not affect cell viability or diameter (Supplemental Figures 1A and 1B). To confirm a response by the cells due to the fatty acid exposure, the expression of *CPT1A*, the rate-limiting enzyme in β-fatty acid oxidation, was measured. *CPT1A* expression increased compared with control (EPA: 12.4 ± SE 1.9-fold [*P* < 0.001]; OA: 19.5 ± SE 3.0-fold [*P* < 0.001]; PA: 11.3 ± SE 2.2-fold [*P* < 0.003]) (Figure 1B). This signifies a consistent response to EPA, OA, and PA exposure.

**Figure 2:**
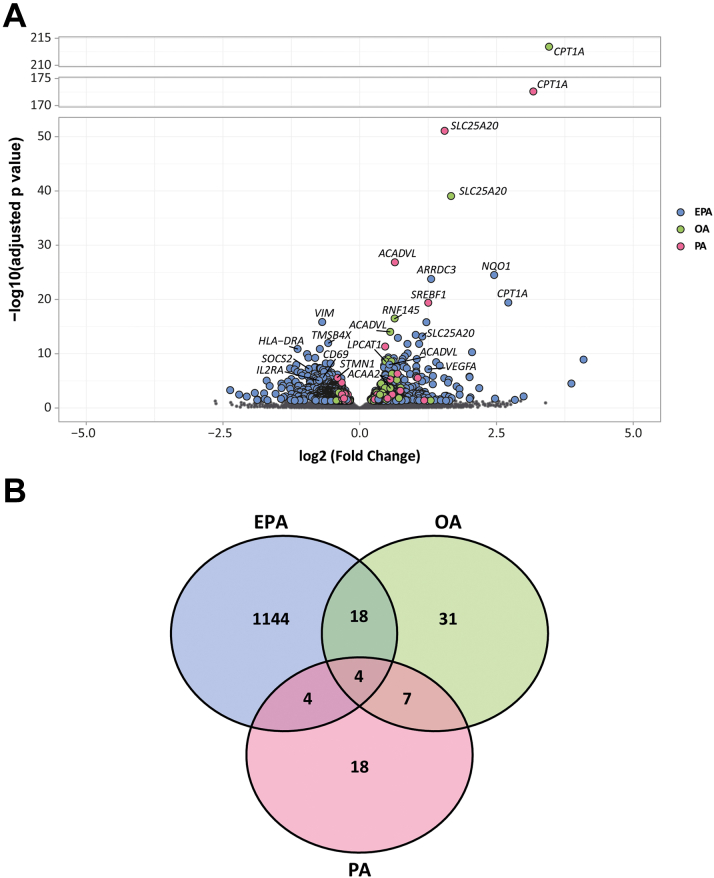
EPA, OA, and PA Exposure in Nonactivated CD4^+^ T Cells Induces Changes in Transcriptomics (A) Volcano plot showing the gene expression of nonactivated CD4^+^ T cells exposed to EPA, OA, or PA. All 19,991 protein-coding genes are shown for each fatty acid. DEGs are colored by fatty acid and denoted by a larger size. Nonsignificant genes are shown in grey and denoted by a smaller size. Log2 fold change is used to show the direction of gene expression. (B) Venn diagram showing the unique response of nonactivated CD4^+^ T cells to each fatty acid. Values are colored by fatty acid. There are 6 DEGs overlapping for all 3 fatty acids, 18 DEGs overlapping for EPA and OA, 4 DEGs overlapping for EPA and PA, and 7 DEGs overlapping for OA and PA. EPA = eicosapentaenoic acid; OA = oleic acid; PA = palmitic acid.

Next, we studied the transcriptomic response of CD4^+^ T cells to EPA, OA, and PA compared with control with the use of RNA-seq. The transcriptional response was compared with the control condition for each fatty acid. The number of differentially expressed genes (DEGs) and effect sizes were markedly larger for EPA, than for OA and PA (Figure 2A) and there was limited overlap between the DEGs of each fatty acid (Figure 2B). EPA induced 1,170 DEGs (*P*_FDR_ <0.05), 723 of which were down-regulated and 447 up-regulated (Supplemental Tables 1A and 1B). In contrast, OA induced 60 DEGs (*P*_FDR_ <0.05; 13 down-regulated and 47 up-regulated) (Supplemental Tables 1C and 1D). PA induced found 33 DEGs (*P*_FDR_ <0.05; 15 down-regulated and 18 up-regulated) (Supplemental Tables 1E and 1F). Despite the high specificity of the transcriptional response of each fatty acid, 4 genes were up-regulated on exposure of each one of the 3 fatty acids. These genes were involved in β-fatty acid oxidation (*CPT1A*, *SLC25A20*, *ACADVL*, and *ACAA2*) in line with a generic cellular response to fatty acid exposure regardless of the fatty acid type (Supplemental Table 1G).

**Figure 3:**
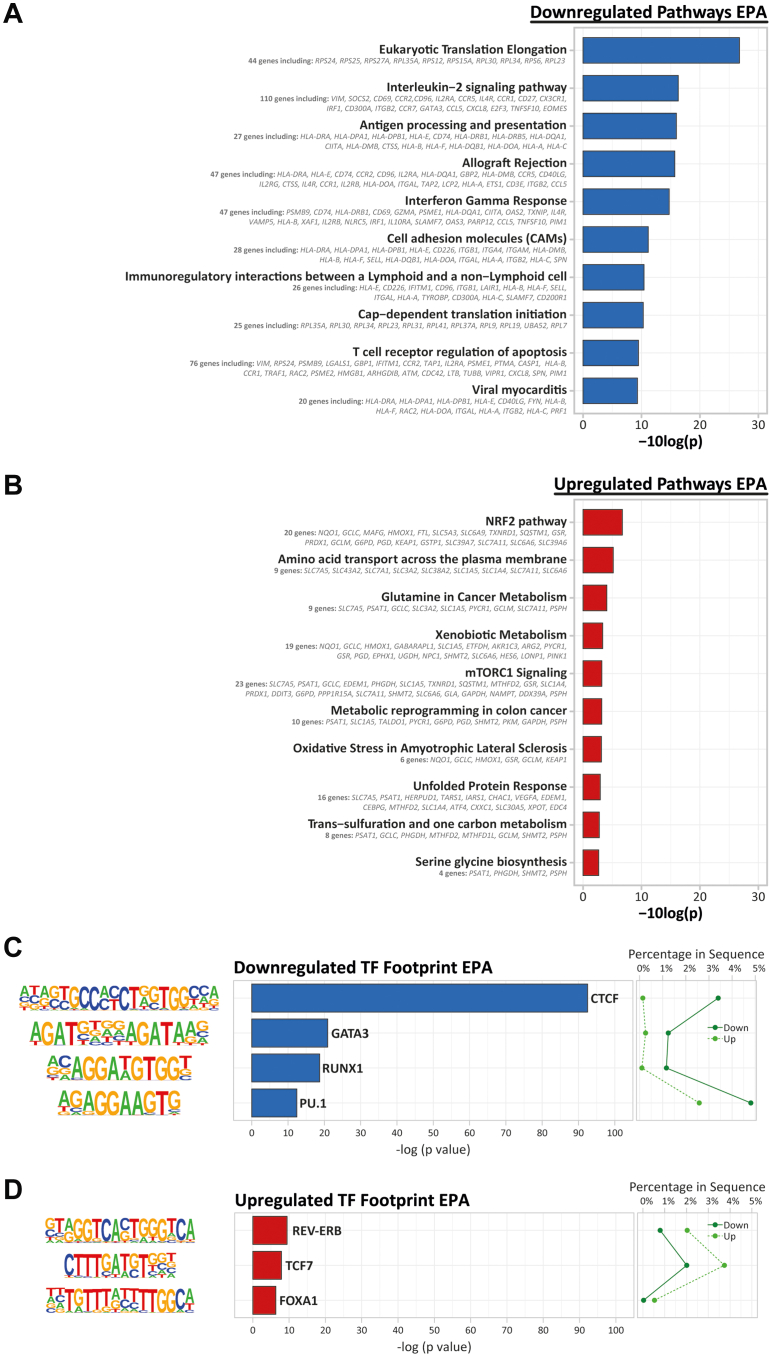
Up- and Down-Regulated Pathways and Transcription Factors in EPA-Exposed Nonactivated CD4^+^ T Cells (A) Pathway enrichment analysis of all down-regulated EPA DEGs generated with the use of *clusterProfiler* using 10 human pathway databases. Top 10 enrichments are shown. (B) Pathway enrichment analysis of all up-regulated EPA DEGs generated with the use of *clusterProfiler* using 10 human pathway databases. Top 10 enrichments are shown. (C) Known motif analysis on promotors of down- vs up-regulated EPA ATAC peaks. Enrichment of transcription factor (TF)–binding motifs was performed with the use of HOMER. Four motifs are shown with supplementing information on *P* value, percentage of genes in up-regulated gene set in down-regulated gene set, transcription factor name, −log(*P* value), and percentage in sequence. (D) Known motif analysis on promotors of up- vs down-regulated EPA ATAC peaks. Enrichment of transcription factor binding motifs was performed with the use of HOMER. Four motifs are shown with supplementing information on *P* value, percentage of genes in up-regulated gene set and in down-regulated gene set, transcription factor name, −log(*P* value), and percentage in sequence. EPA = eicosapentaenoic acid; TF = transcription factor.

We focused on the marked transcriptomic response of CD4^+^ T cells to EPA. First, we analyzed the 723 down-regulated genes in EPA-exposed nonactivated CD4^+^ T cells. The top 3 DEGs were *VIM* (vimentin), *TMSB4X* (thymosin beta-4 X-linked), and *HLA-DRA* (major histocompatibility complex, class II, DR alpha). *VIM* and *TMSB4X* both encode structures involved in the makeup of the cytoskeleton. HLA-DRA plays a central role in the immune response by presenting peptides to T cells. Remarkably, many other immune response genes were also down-regulated, including *SOCS2* (suppressor of cytokine signaling 2), *CD69* (CD69 molecule), and *IL2RA* (interleukin-2 receptor subunit alpha). SOCS2 is a negative regulator of cytokine receptor signaling, particularly of IGF1R, an insulin-like growth factor whose expression is associated with the development of T_H_17 over T_reg_ subsets. CD69 plays an integral part in T cell activation, and IL2RA is an important regulator of T cell differentiation. A strong down-regulation of immune-related processes was confirmed by a formal analysis of enriched biological processes. In particular, IL-2 signaling pathway (*P*_FDR_ <0.001; 110 DEGs), antigen processing and presentation (*P*_FDR_ <0.001; 27 DEGs), and interferon-γ response (*P*_FDR_ <0.001; 47 DEGs) were enriched (Figure 3A, Supplemental Table 1H). This indicates that EPA reduces immune-related gene expression in nonactivated CD4^+^ T cells.

Second, we analyzed the 447 up-regulated genes in EPA-exposed nonactivated CD4^+^ T cells. The top 3 DEGs were *NQO1* (NAD[P]H quinone dehydrogenase 1), *ARRDC3* (arrestin domain containing 3), and *CPT1A*. NQO1 is involved in protecting cells against oxidative stress, which can be caused by lipid peroxidation, and *ARRDC3* encodes a regulator of G protein–mediated signaling. Another gene of interest that was up-regulated was *VEGFA* (vascular endothelial growth factor A). The enzyme encoded by this gene is a proangiogenic molecule known to be involved in creating immunosuppressive environments. This immunosuppressive profile was further supported by a formal analysis of enriched biological processes, which showed up-regulation of the nuclear factor erythroid-2–related factor 2 (NRF2) pathway (*P*_FDR_ <0.001; 20 DEGs) (Figure 3B, Supplemental Table 1I). This pathway is the most important pathway for protecting cells against oxidative stress and has been shown to be involved in anti-inflammatory responses. Overall, these results suggests that EPA exposure can alter gene expression in nonactivated T cells toward an anti-inflammatory profile by decreasing immune response–related genes and increasing protective genes, such as those in the NRF2 pathway.

Next, we investigated whether specific transcription factors may underlie the differential gene expression. To do this, we examined the enrichment of transcription factor–binding motifs in loci that were more closed (down) vs more open (up) as determined by ATAC sequencing. The top EPA down-regulated motifs included CTCF, GATA3, RUNX1, and PU.1 (Figure 3C, Supplemental Table 1J). CTCF is a master regulator of chromatin looping and, moreover, is involved in effector cell differentiation.^39^^,^^40^ GATA3 and PU.1 are the key transcription factors for the development of T_H_2 and T_H_9 cells, respectively.^41^^,^^42^ RUNX1 is necessary for T cell maturation; knockouts of this transcription factor results in phenotypically and functionally immature T cells.^43^ We next examined the enrichment of transcription factor binding motifs in up-regulated versus down-regulated genes. EPA up-regulated motifs included REV-ERB, TCF7, and FOXA1 (Figure 3D, Supplemental Table 1K). REV-ERB is an antagonist of RORγt, the key transcription factor for the development of T_H_17 cells.^44^ TCF7 plays a role in the regulation of autoinflammatory T cell responses.^45^ FOXA1 is involved in giving T_reg_ cells their suppressive properties.^46^ These results further suggest that nonactivated CD4^+^ T cells may decrease their ability to induce an immune response or effector T cell profile after EPA exposure.

### Transcriptomic analysis of OA- and PA-exposed nonactivated CD4^+^ T cells

Nonactivated CD4^+^ T cells were also exposed to either OA or PA and differential gene expression was measured. In line with our previous experiments, we found that OA exposure led to the down-regulation of genes related to endogenous peptide antigen presentation (*HSPA5* and *PDIA3*; *P*_FDR_ <0.001; 2 DEGs) and electron transport chain and oxidative phosphorylation activity (*NDUFA12*, *NDUFB4*, and *ATP5F1C*; *P*_FDR_ <0.001; 3 DEGs), and up-regulation of cholesterol biosynthesis (*HMGCR*, *HMGCS1*, and *DHCR24*; *P*_FDR_ <0.001; 4 DEGs) (Supplemental Figures 2A and 2B, Supplemental Tables 1L and 1M). Exposure to PA induced an opposite response, with the down-regulation of genes related to cholesterol biosynthesis pathway (*HMGCR* and *SQLE*; *P*_FDR_ <0.05; 2 DEGs) and up-regulated beta fatty acid oxidation (*CPT1A*, *SLC25A20*, and *ACADVL*; *P*_FDR_ <0.001; 3 DEGs) (Supplemental Figures 2C and 2D, Supplemental Tables 1N and 1O). Thus, the changes in the transcriptome of OA- and PA-exposed cells seem to have a greater effect on genes involved in cellular metabolism, particularly cholesterol metabolism, compared with EPA.

The observed transcriptional responses were in line with the results of ATAC sequencing–based transcription factor footprint analysis. For OA, only 3 motifs were down-regulated, including RAR:RXR, a motif known to play a part in the development of T_reg_ over T_H_17 cells (Supplemental Figure 3A, Supplemental Table 1P). OA up-regulated motifs included PU.1, as was found previously,^29^ as well as IRF8, which is also involved in T_H_9 differentiation (Supplemental Figure 3B, Supplemental Table 1Q). For PA, down-regulated motifs included IRF8 and GATA3 (Supplemental Figure 3C, Supplemental Table 1R) and up-regulated motifs included REV-ERB (Supplemental Figure 3D, Supplemental Table 1). OA and PA showed reversed effects on cholesterol metabolism processes, which were mirrored in opposite associations with transcription factor–binding motifs, indicating fatty acid–specific responses in nonactivated CD4^+^ T cells.

## Discussion

IPE, the highly purified form of EPA, has been associated with reduced triglycerides, cardiovascular events, and cardiovascular death in individuals with relatively well controlled LDL levels, even when corrected for placebo response in the mineral oil control group, LDL, and C-reactive protein in the REDUCE-IT trial.^10^^,^^47^, ^48^, ^49^, ^50^ Trial outcomes and interpretation have been widely debated, and the mechanisms by which EPA exerts its beneficial effects remain incompletely understood.^11^^,^^12^ We show that EPA exposure can already produce distinct changes in T cells before activation by decreasing the expression of immune-response genes and increasing the expression of genes involved in oxidative stress protection. This is further supported by changes in transcription factor–binding sites in our ATAC-sequencing motif analysis, indicating a change in the epigenetic landscape of EPA-exposed T cells. Furthermore, we show that EPA induces a unique response in nonactivated CD4^+^ T cells, because 2 other fatty acids of varying degrees of saturation, OA and PA, generated a smaller yet distinct effect on gene expression profiles in T cells compared with control. Our findings suggest that different fatty acids in the circulation can induce diverse effects on T cell transcriptomics, and that EPA exposure in particular may poise T cells to have clearer anti-inflammatory responses. These results underscore a potential mechanism by which EPA may mitigate ASCVD risk, suggesting its anti-inflammatory impact on T cells as a contributing factor. This is particularly noteworthy because T cells constitute more than half of the immune cell population within atherosclerotic plaques.^17^^,^^18^

Our results show that EPA exposure, but not OA nor PA, leads to a strong down-regulation of immune response–related genes. In particular, genes involved in antigen processing and presentation were down-regulated in EPA-exposed cells, denoted by, among others, the decreased expression of 14 different HLA genes. This gene group is crucial in inducing immune responses^51^ and has also been found to be associated with T cell activation and effector memory phenotype in CD4^+^ T cells.^52^ In addition, genes involved in IL-2 signaling were down-regulated, which is required for T cell activation.^53^ Down-regulation of genes in these pathways suggests that EPA-exposed T cells may have a reduced ability to initiate an immune response, a key component of inflammatory responses in atherosclerotic plaques.^54^ This result can support the finding that higher plasma EPA levels are associated with lower CVD risk in humans.^55^ Furthermore, genes involved in proinflammatory pathways, such as interferon-γ response, were down-regulated in EPA exposed cells. Interferon-γ is primarily produced by the proinflammatory T cell subset T_H_1 cells, which have also been found to decrease on EPA exposure.^27^^,^^56^, ^57^, ^58^ Moreover, the key transcription factors in T_H_2 and T_H_9 differentiation, GATA3 and PU.1, were found to be decreased in our motif analysis. Whereas T_H_2 cells have inconclusive effects on ASCVD, T_H_9 cells have been shown to aggravate it.^59^, ^60^, ^61^ Thus, EPA exposure decreased genes involved in immune response and proinflammatory pathways, as well as suggests a reduced ability for key T cell differentiation transcription factors to bind.

In further support of EPA’s anti-inflammatory properties on nonactivated CD4^+^ T cells, we found that genes involved in the NRF2 pathway were up-regulated on EPA exposure. This pathway mainly functions in preventing oxidative stress in cells by activating genes involved in detoxification and removal of reactive oxygen species.^62^ However, the NRF2 pathway has also been shown to aid in the anti-inflammatory responses of macrophages^63^ and has been suggested as a beneficial pleiotropic effect of statins,^64^ because oxidative stress has been found to be a risk factor for ASCVD.^65^ We also found an increased footprint for the transcription factors REV-ERB, TCF7, and FOXA1. These transcription factors are each involved in regulating T cell responses and generating a more anti-inflammatory T cell profile.^44^, ^45^, ^46^ Overall, these data indicate that nonactivated CD4^+^ T cells can already acquire an anti-inflammatory transcriptomic profile, which may play a role in the anti-inflammatory properties observed of EPA in clinical trials.

EPA has a distinct effect on CD4^+^ T cells. This is observed by our analysis of the effects of OA and PA on nonactivated CD4^+^ T cells. The number of DEGs and effect sizes were smaller with OA and PA exposure and distinctly different. Interestingly, OA and PA had opposing effects on cholesterol biosynthesis–related genes, with OA up-regulating and PA down-regulating genes in this pathway. Up-regulation of cholesterol biosynthesis has been related to the development of T_H_17 cells by controlling RORγt activity, the key transcription factor in T_H_17 differentiation.^66^^,^^67^ This observation can be further supported by OA down-regulating the RAR:RXR motif, which is involved in generating T_reg_ cells over T_H_17, and PA up-regulating REV-ERB.^44^^,^^68^ The results of OA exposure also show the robustness of our approach because our findings here match what was found previously by our group.^29^ These data suggest that our model is robust and each fatty acid induces its own unique response in nonactivated CD4^+^ T cells.

### Study strengths and limitations

We show that EPA exposure has beneficial anti-inflammatory effects on nonactivated CD4^+^ T cells. This is relevant because T cells are largely nonactivated in the circulation and it is in the circulation where T cells will encounter EPA when individuals are treated with IPE to reduce ASCVD risk. Nevertheless, our results are in line with experiments on activated T cells, which showed that EPA exposure decreased proliferation,^22^, ^23^, ^24^, ^25^, ^26^ decreased T_H_1 and T_H_17 populations,^27^^,^^28^ and had no effect on or increased T_H_2 and T_reg_ populations.^26^, ^27^, ^28^ Therefore, the encounter with EPA in a nonactivated state may induce transcriptomic changes that influence the functional changes after activation. Furthermore, our use of nonactivated CD4^+^ T cells with no additional selection towards naïve, effector, memory, or specific T_H_ subsets, as well as in culture medium containing other lipids, more closely represents the diversity of T cells and environment of the circulation in which EPA exposure takes place. In addition, we utilized OA and PA, 2 fatty acids of varying degrees of saturation to establish the distinct effects of EPA. Nevertheless, this does not rule out that other fatty acids may have marked effects on non-activated CD4^+^ T cells as well.^21^ Another limitation of our study is that we used an in vitro model, and the effects of EPA on T cells in vivo should be studied in the context of trials of IPE. However, in mouse models, EPA supplementation has also been shown to reduce cholesterol levels,^69^ whereas in humans, the effects of EPA on ASCVD risk were independent from LDL lowering.^48^ Therefore, using a validated in vitro model provides valuable insights to study the effects of EPA on human CD4^+^ T cells.

## Conclusions

Our data show that EPA produces a strong and specific anti-inflammatory transcriptional profile in nonactivated CD4^+^ T cells, composed of both the down-regulation of immune-related genes and the up-regulation of antioxidant genes. This profile is supported by transcription factor motif analysis and by the analysis of 2 other fatty acids of varying degrees of saturation. Our results contribute to the debate on how EPA exerts beneficial effects in human ASCVD. Our study indicates that the beneficial effects observed for EPA, as shown in clinical trials, can start in the circulation by inducing an anti-inflammatory transcriptional profile in nonactivated T cells with potentially anti-atherosclerotic properties.

Perspectives **COMPETENCY IN MEDICAL KNOWLEDGE:** We report that EPA induces an anti-inflammatory transcriptomic profile in non-activated CD4^+^ T cells. This observation is important to better understand the mechanism through which EPA reduces cardiovascular disease risk in studies such as the REDUCE-IT trial.

**TRANSLATIONAL OUTLOOK:** The REDUCE-IT trial showed that intervention with IPE, which the body metabolizes to EPA, reduces the risk of cardiovascular events and death in patients with high triglycerides. The findings of that trial have sparked considerable debate because the results appeared to occur regardless of the attained triglyceride level after 1 year.^10^, ^11^, ^12^ Testing T cells from individuals undergoing IPE interventions can provide additional insights into how EPA exerts its beneficial effects independently from triglyceride reduction.

## Funding Support and Author Disclosures

The authors’ work is supported by the Dutch Cardiovascular Alliance (Dutch Heart Foundation, Dutch Federation of University Medical Centers, Netherlands Organization for Health Research and Development, and Royal Netherlands Academy of Sciences) for the GENIUSII project Generating the Best Evidence-Based Pharmaceutical Targets for Atherosclerosis (CVON2017-20). The authors have reported that they have no relationships relevant to the contents of this paper to disclose.

## References

[1] Lawler P.R., Bhatt D.L., Godoy L.C. (2021). Targeting cardiovascular inflammation: next steps in clinical translation. Eur Heart J.

[2] Sabatine M.S., Giugliano R.P., Keech A.C. (2017). Evolocumab and clinical outcomes in patients with cardiovascular disease. N Engl J Med.

[3] Schade D.S., Eaton R.P. (2018). Residual cardiovascular risk—is inflammation the primary cause?. World J Cardiovasc Dis.

[4] Lawler P.R., Kotrri G., Koh M. (2020). Real-world risk of cardiovascular outcomes associated with hypertriglyceridaemia among individuals with atherosclerotic cardiovascular disease and potential eligibility for emerging therapies. Eur Heart J.

[5] ACCORD Study Group (2010). Effects of intensive blood-pressure control in type 2 diabetes mellitus. N Engl J Med.

[6] Keech A., Simes R.J., Barter P. (2005). Effects of long-term fenofibrate therapy on cardiovascular events in 9795 people with type 2 diabetes mellitus (the FIELD study): randomised controlled trial. Lancet.

[7] Das Pradhan A., Glynn R.J., Fruchart J.C. (2022). Triglyceride lowering with pemafibrate to reduce cardiovascular risk. N Engl J Med.

[8] AIM-HIGH Investigators (2011). Niacin in patients with low HDL cholesterol levels receiving intensive statin therapy. N Engl J Med.

[9] Landray M.J., Haynes R. (2014). Effects of extended-release niacin with laropiprant in high-risk patients. N Engl J Med.

[10] Bhatt D.L., Steg P.G., Miller M. (2019). Cardiovascular risk reduction with icosapent ethyl for hypertriglyceridemia. N Engl J Med.

[11] Kastelein J.P.J., Stroes E.S.G. (2019). FISHing for the miracle of eicosapentaenoic acid. N Engl J Med.

[12] Steg P.G., Bhatt D.L. (2021). The reduction in cardiovascular risk in REDUCE-IT is due to eicosapentaenoic acid in icosapent ethyl. Eur Heart J.

[13] Mason R.P., Jacob R.F., Shrivastava S., Sherratt S.C.R., Chattopadhyay A. (2016). Eicosapentaenoic acid reduces membrane fluidity, inhibits cholesterol domain formation, and normalizes bilayer width in atherosclerotic-like model membranes. Biochim Biophys Acta.

[14] Tsunoda F., Lamon-Fava S., Asztalos B.F. (2015). Effects of oral eicosapentaenoic acid versus docosahexaenoic acid on human peripheral blood mononuclear cell gene expression. Atherosclerosis.

[15] Vors C., Allaire J., Marin J. (2017). Inflammatory gene expression in whole blood cells after EPA vs DHA supplementation: results from the ComparED study. Atherosclerosis.

[16] Wolf D., Ley K. (2019). Immunity and inflammation in atherosclerosis. Circ Res.

[17] Fernandez D.M., Rahman A.H., Fernandez N.F. (2019). Single-cell immune landscape of human atherosclerotic plaques. Nat Med.

[18] Depuydt M.A., Prange K.H., Slenders L. (2020). Microanatomy of the human atherosclerotic plaque by single-cell transcriptomics. Circ Res.

[19] Zhou X., Robertson A.K., Hjerpe C., Hansson G.K. (2006). Adoptive transfer of CD4+ T cells reactive to modified low-density lipoprotein aggravates atherosclerosis. Arterioscle. Thromb Vasc Biol.

[20] Zhou X., Nicoletti A., Elhage R., Hansson G.K. (2000). Transfer of CD4+ T cells aggravates atherosclerosis in immunodeficient apolipoprotein E knockout mice. Circulation.

[21] Reilly N.A., Lutgens E., Kuiper J., Heijmans B.T., Wouter Jukema J. (2021). Effects of fatty acids on T cell function: role in atherosclerosis. Nat Rev Cardiol.

[22] Fan Y.Y., Fuentes N.R., Hou T.Y. (2018). Remodelling of primary human CD4+ T cell plasma membrane order by n-3 PUFA. Br J Nutr.

[23] Gorjão R., Cury-Boaventura M.F., de Lima T.M., Curi R. (2007). Regulation of human lymphocyte proliferation by fatty acids. Cell Biochem Funct.

[24] Ly L.H., Smith R., Switzer K.C., Chapkin R.S., McMurray D.N. (2006). Dietary eicosapentaenoic acid modulates CTLA-4 expression in murine CD4+ T-cells. Prostaglandins Leukot Essent Fatty Acids.

[25] Jolly C.A., Jiang Y.H., Chapkin R.S., McMurray D.N. (1997). Dietary (n-3)polyunsaturated fatty acids suppress murine lymphoproliferation, interleukin-2 secretion, and the formation of diacylglycerol and ceramide. J Nutr.

[26] Merzouk S.A., Saker M., Reguig K.B. (2008). n-3 polyunsaturated fatty acids modulate in vitro T cell function in type I diabetic patients. Lipids.

[27] Bi X., Li F., Liu S. (2017). ω-3 polyunsaturated fatty acids ameliorate type 1 diabetes and autoimmunity. J Clin Invest.

[28] Monk J.M., Hou T.Y., Turk H.F., McMurray D.N., Chapkin R.S. (2013). n3 PUFAs reduce mouse CD4+ T-cell ex vivo polarization into T_H_17 cells. J Nutr.

[29] Reilly N.A., Sonnet F., Dekkers K.F. (2024). Oleic acid triggers metabolic rewiring of T cells poising them for T helper 9 differentiation. iScience.

[30] Su B., Bettcher L.F., Hsieh W.Y. (2021). A DMS shotgun lipidomics workflow application to facilitate high-throughput, comprehensive lipidomics. J Am Soc Mass Spectrom.

[31] Ghorasaini M., Tsezou K.I., Verhoeven A. (2022). Congruence and complementarity of differential mobility spectrometry and NMR spectroscopy for plasma lipidomics. Metabolites.

[32] van der Vusse G.J. (2009). Albumin as fatty acid transporter. Drug Metab Pharmacokinet.

[33] Ledderose C., Heyn J., Limbeck E., Kreth S. (2011). Selection of reliable reference genes for quantitative real-time PCR in human T cells and neutrophils. BMC Res Notes.

[34] Corces M.R., Trevino A.E., Hamilton E.G. (2017). An improved ATAC-seq protocol reduces background and enables interrogation of frozen tissues. Nat Methods.

[35] Heinz S., Benner C., Spann N. (2010). Simple combinations of lineage-determining transcription factors prime cis-regulatory elements required for macrophage and B cell identities. Mol Cell.

[36] R Core Team (2023).

[37] Love M.I., Huber W., Anders S. (2014). Moderated estimation of fold change and dispersion for RNA-seq data with DESeq2. Genome Biol.

[38] Yu G., Wang L.G., Han Y., He Q.Y. (2012). clusterProfiler: an R package for comparing biological themes among gene clusters. OMICS.

[39] Phillips J.E., Corces V.G. (2009). CTCF: master weaver of the genome. Cell.

[40] Zhao X., Zhu S., Peng W., Xue H.H. (2022). The interplay of transcription and genome topology programs T cell development and differentiation. J Immunol.

[41] Nakayama T., Hirahara K., Onodera A. (2017). T_H_2 cells in health and disease. Annu Rev Immunol.

[42] Angkasekwinai P. (2019). T_H_9 cells in allergic disease. Curr Allergy Asthma Rep.

[43] Hsu F.C., Shapiro M.J., Dash B. (2016). An essential role for the transcription factor Runx1 in T cell maturation. Sci Rep.

[44] Mosure S.A., Wilson A.N., Solt L.A. (2022). Targeting nuclear receptors for T_H_17-mediated inflammation: REV-ERBerations of circadian rhythm and metabolism. Immunometabolism.

[45] Mammadli M., Suo L., Sen J.M., Karimi M. (2023). TCF-1 is required for CD4 T cell persistence functions during alloimmunity. Int J Mol Sci.

[46] Liu Y., Carlsson R., Comabella M. (2014). FoxA1 directs the lineage and immunosuppressive properties of a novel regulatory T cell population in EAE and MS. Nat Med.

[47] Olshansky B., Chung M.K., Budoff M.J. (2020). Mineral oil: safety and use as placebo in REDUCE-IT and other clinical studies. Eur Heart J Suppl.

[48] Sharretts D. (November 14, 2019). FDA Briefing Document for the Endocrinologic and Metabolic Drugs Advisory Committee Meeting. https://wayback.archive-it.org/7993/20201227020822/https://www.fda.gov/media/132477/download.

[49] Amarin Pharmaceuticals Ireland Limited (November 14, 2019). Advisory Committee Briefing Document for the Endocrinologic and Metabolic Drugs Advisory Committee Meeting. https://wayback.archive-it.org/7993/20201227020827/https://www.fda.gov/media/132479/download.

[50] European Medicines Agency (January 28, 2021). Assessment report: Vazkepa. Committee for Medicinal Products for Human Use. https://www.ema.europa.eu/en/documents/assessment-report/vazkepa-epar-public-assessment-report_en.pdf.

[51] Klein J., Sato A. (2000). The HLA system. N Engl J Med.

[52] Tippalagama R., Singhania A., Dubelko P. (2021). HLA-DR marks recently divided antigen-specific effector CD4 T cells in active tuberculosis patients. J Immunol.

[53] Chapman N.M., Boothby M.R., Chi H. (2020). Metabolic coordination of T cell quiescence and activation. Nat Rev Immunol.

[54] Grivel J.C., Ivanova O., Pinegina N. (2011). Activation of T lymphocytes in atherosclerotic plaques. Arterioscle. Thromb Vasc Biol.

[55] de Oliveira Otto MC., Wu J.H., Baylin A. (2013). Circulating and dietary omega-3 and omega-6 polyunsaturated fatty acids and incidence of CVD in the Multi-Ethnic Study of Atherosclerosis. J Am Heart Assoc.

[56] Zhang P., Smith R., Chapkin R.S., McMurray D.N. (2005). Dietary (n-3)polyunsaturated fatty acids modulate murine T_H_1/T_H_2 balance toward the T_H_2 pole by suppression of T_H_1 development. J Nutr.

[57] Zhang P., Kim W., Zhou L. (2006). Dietary fish oil inhibits antigen-specific murine T_H_1 cell development by suppression of clonal expansion. J Nutr.

[58] Switzer K.C., McMurray D.N., Morris J.S., Chapkin R.S. (2003). (n-3)Polyunsaturated fatty acids promote activation-induced cell death in murine T lymphocytes. J Nutr.

[59] Zhang W., Tang T., Nie D. (2015). IL-9 aggravates the development of atherosclerosis in ApoE2/2 mice. Cardiovasc. Res.

[60] Gregersen I., Skjelland M., Holm S. (2013). Increased systemic and local interleukin 9 levels in patients with carotid and coronary atherosclerosis. PLoS One.

[61] Li Q., Ming T., Wang Y. (2017). Increased T_H_9 cells and IL-9 levels accelerate disease progression in experimental atherosclerosis. Am J Transl Res.

[62] Nguyen T., Nioi P., Pickett C.B. (2009). The Nrf2–antioxidant response element signaling pathway and its activation by oxidative stress. J Biol Chem.

[63] Wang L., He C. (2022). Nrf2-mediated anti-inflammatory polarization of macrophages as therapeutic targets for osteoarthritis. Front Immunol.

[64] Mansouri A., Reiner Z., Ruscica M. (2022). Antioxidant effects of statins by modulating Nrf2 and Nrf2/HO-1 signaling in different diseases. J Clin Med.

[65] Batty M., Bennett M.R., Yu E. (2022). The role of oxidative stress in atherosclerosis. Cells.

[66] Kanno T., Nakajima T., Miyako K., Endo Y. (2023). Lipid metabolism in T_H_17 cell function. Pharmacol Ther.

[67] Kidani Y., Bensinger S.J. (2017). Reviewing the impact of lipid synthetic flux on T_H_17 function. Curr Opin Immunol.

[68] Takeuchi H., Yokota-Nakatsuma A., Ohoka Y. (2013). Retinoid X receptor agonists modulate Foxp3+ regulatory T cell and T_H_17 cell differentiation with differential dependence on retinoic acid receptor activation. J Immunol.

[69] Laguna-Fernandez A., Checa A., Carracedo M. (2018). ERV1/ChemR23 signaling protects against atherosclerosis by modifying oxidized low-density lipoprotein uptake and phagocytosis in macrophages. Circulation.

