# Supplementary material for: EPA Induces an Anti-Inflammatory Transcriptome in T Cells, Implicating a Triglyceride-Independent Pathway in Cardiovascular Risk Reduction
Source: JACC Basic Transl Sci. 2024 Oct 30;10(3):383–95. doi: 10.1016/j.jacbts.2024.09.002 (PMC12013851; doi:10.1016/j.jacbts.2024.09.002)
Supplement: Supplemental Material [file mmc1.pdf]

Supplemental Figure 1: Verification of viability and cell diameter post-exposure for RNA sequencing. (a) Bar plot showing the average cell viability and standard error in percent, as determined by Via1-Cassette™ on a NucleoCounter® NC-200™.The mean ± SEM of the cell viability of exposed cells was 97.7 ± 0.4% for control, 97.4 ± 0.3% ($p > 0.05$) for EPA, 97.5 ± 0.5% ($p > 0.05$) for OA, 98.0 ± 0.4% ($p > 0.05$) for PA cells at 48. Thus, there was no effect on $CD4^+$ T cell viability after 48 hour exposure, n = 8. **(b)** Dot plot showing the average cell diameter and standard error in µm, as determined by Via1-Cassette™ on a NucleoCounter® NC-200™. The mean ± SEM cell diameter of exposed cells was 9.1 ± 0.03 µm for control, 9.1 ± 0.02 µm ($p > 0.05$) for EPA, 9.0 ± 0.04 µm ($p > 0.05$) for OA, and 9.1 ± 0.03 µm ($p > 0.05$) PA cells at 48h. Thus, there was no effect on $CD4^+$ T cell diameter after 48h exposure, n = 8. Abbreviations, EPA = eicosapentaenoic acid, h = hours, OA = oleic acid, PA = palmitic acid.

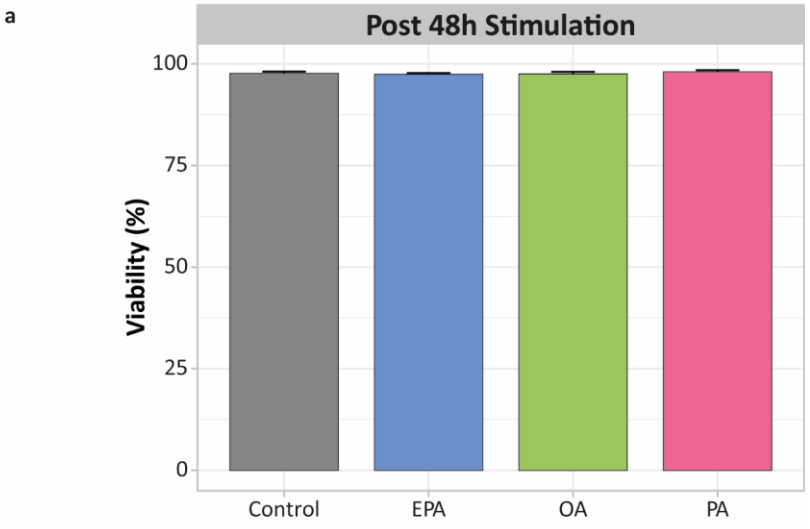

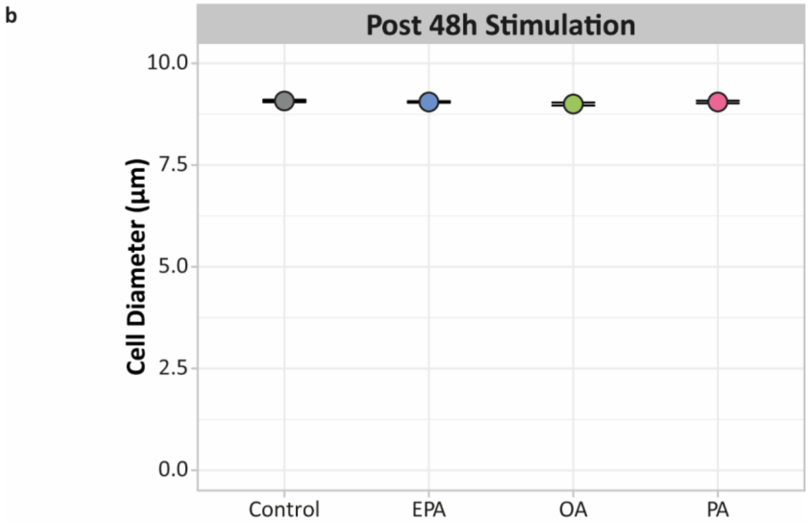

Supplemental Figure 2: Up- and downregulated pathways in PA and OA-exposed non-activated $CD4^+$ T cells. (a) Pathway enrichment analysis of all downregulated OA DEGs generated using *clusterProfiler* using 10 human pathway databases. Top 5 enrichments are shown. **(b)** Pathway enrichment analysis of all upregulated OA DEGs generated using *clusterProfiler* using 10 human pathway databases. Top 5 enrichments are shown. **(c)** Pathway enrichment analysis of all downregulated PA DEGs generated using *clusterProfiler* using 10 human pathway databases. Top 5 enrichments are shown. **(d)** Pathway enrichment analysis of all upregulated PA DEGs generated using *clusterProfiler* using 10 human pathway databases. Top 5 enrichments are shown. Abbreviations, OA = oleic acid, PA = palmitic acid.

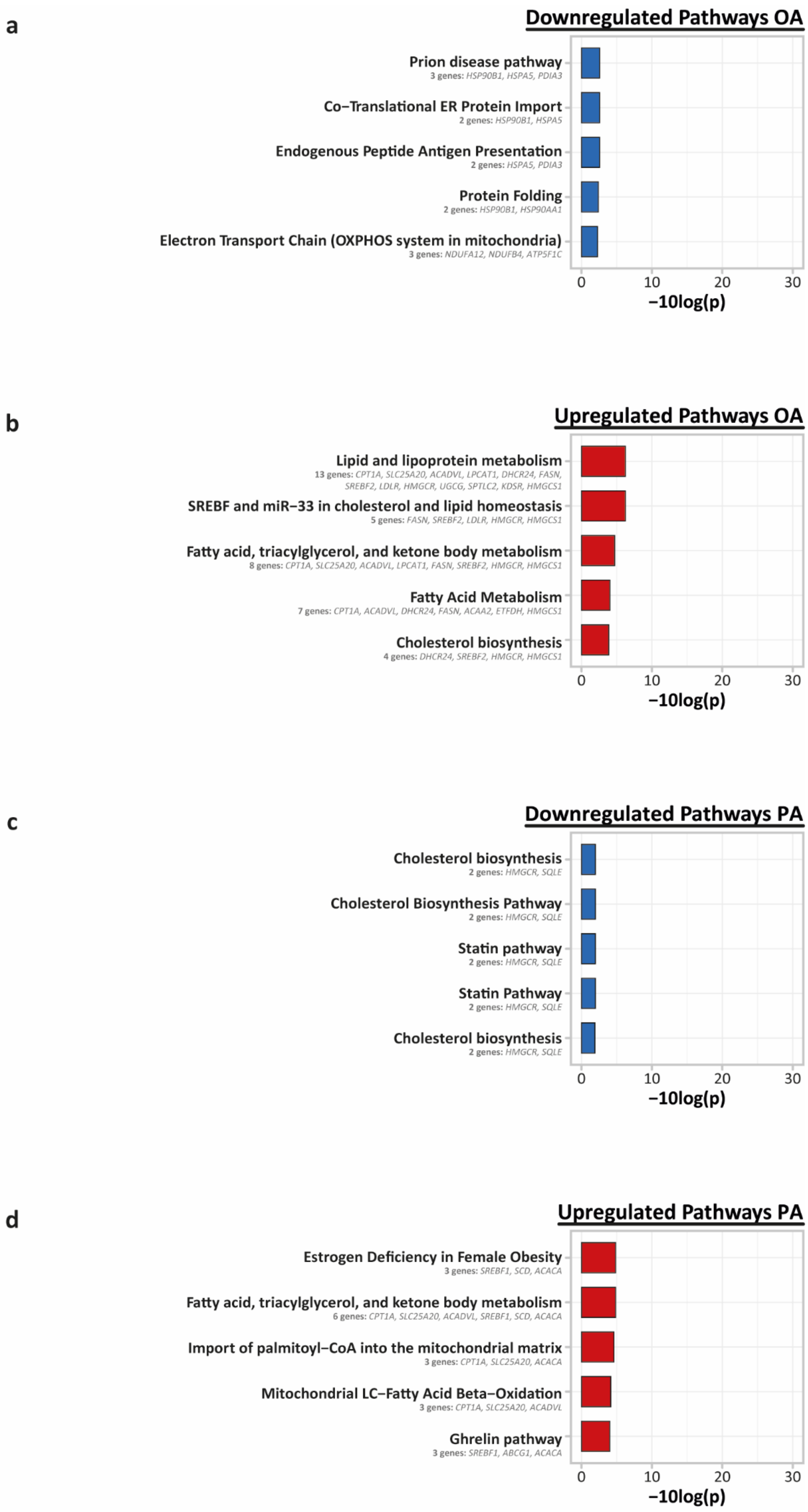
a
Downregulated Pathways OA
Prion disease pathway
3 genes: HSP90B1, HSPA5, PDIA3
Co−Translational ER Protein Import
2 genes: HSP90B1, HSPA5
Endogenous Peptide Antigen Presentation
2 genes: HSPA5, PDIA3
Protein Folding
2 genes: HSP90B1, HSP90AA1
Electron Transport Chain (OXPHOS system in mitochondria)
3 genes: NDUFA12, NDUFB4, ATP5F1C
0
10
20
30
−10log(p)
b
Upregulated Pathways OA
Lipid and lipoprotein metabolism
13 genes: CPT1A, SLC25A20, ACADVL, LPCAT1, DHCR24, FASN, SREBF2, LDLR, HMGCR, UGCG, SPTLC2, KDSR, HMGCS1
SREBF and miR−33 in cholesterol and lipid homeostasis
5 genes: FASN, SREBF2, LDLR, HMGCR, HMGCS1
Fatty acid, triacylglycerol, and ketone body metabolism
8 genes: CPT1A, SLC25A20, ACADVL, LPCAT1, FASN, SREBF2, HMGCR, HMGCS1
Fatty Acid Metabolism
7 genes: CPT1A, ACADVL, DHCR24, FASN, ACAA2, ETFDH, HMGCS1
Cholesterol biosynthesis
4 genes: DHCR24, SREBF2, HMGCR, HMGCS1
0
10
20
30
−10log(p)
c
Downregulated Pathways PA
Cholesterol biosynthesis
2 genes: HMGCR, SQLE
Cholesterol Biosynthesis Pathway
2 genes: HMGCR, SQLE
Statin pathway
2 genes: HMGCR, SQLE
Statin Pathway
2 genes: HMGCR, SQLE
Cholesterol biosynthesis
2 genes: HMGCR, SQLE
0
10
20
30
−10log(p)
d
Upregulated Pathways PA
Estrogen Deficiency in Female Obesity
3 genes: SREBF1, SCD, ACACA
Fatty acid, triacylglycerol, and ketone body metabolism
6 genes: CPT1A, SLC25A20, ACADVL, SREBF1, SCD, ACACA
Import of palmitoyl−CoA into the mitochondrial matrix
3 genes: CPT1A, SLC25A20, ACACA
Mitochondrial LC−Fatty Acid Beta−Oxidation
3 genes: CPT1A, SLC25A20, ACADVL
Ghrelin pathway
3 genes: SREBF1, ABCG1, ACACA
0
10
20
30
−10log(p)

Supplemental Figure 3: Up- and downregulated transcription factors in PA and OA-exposed non-activated CD4$^+$ T cells. (a) Known motif analysis on promotors of down versus upregulated OA ATAC peaks. Enrichment of transcription factor binding motifs was performed using HOMER. 3 motifs are shown with supplementing information on p-value, percentage of genes in upregulated gene set and percentage of genes in downregulated gene set, transcription factor name, -log(p-value), and percentage in sequence. **(b)** Known motif analysis on promotors of up versus downregulated OA ATAC peaks. Enrichment of transcription factor binding motifs was performed using HOMER. 4 motifs are shown with supplementing information on p-value, percentage of genes in upregulated gene set and percentage of genes in downregulated gene set, transcription factor name, -log(p-value), and percentage in sequence. **(c)** Known motif analysis on promotors of down versus upregulated PA ATAC peaks. Enrichment of transcription factor binding motifs was performed using HOMER. 3 motifs are shown with supplementing information on p-value, percentage of genes in upregulated gene set and percentage of genes in downregulated gene set, transcription factor name, -log(p-value), and percentage in sequence. **(d)** Known motif analysis on promotors of up versus downregulated PA ATAC peaks. Enrichment of transcription factor binding motifs was performed using HOMER. 4 motifs are shown with supplementing information on p-value, percentage of genes in upregulated gene set and percentage of genes in downregulated gene set, transcription factor name, -log(p-value), and percentage in sequence. Abbreviations, OA = oleic acid, PA = palmitic acid, TF = transcription factor.

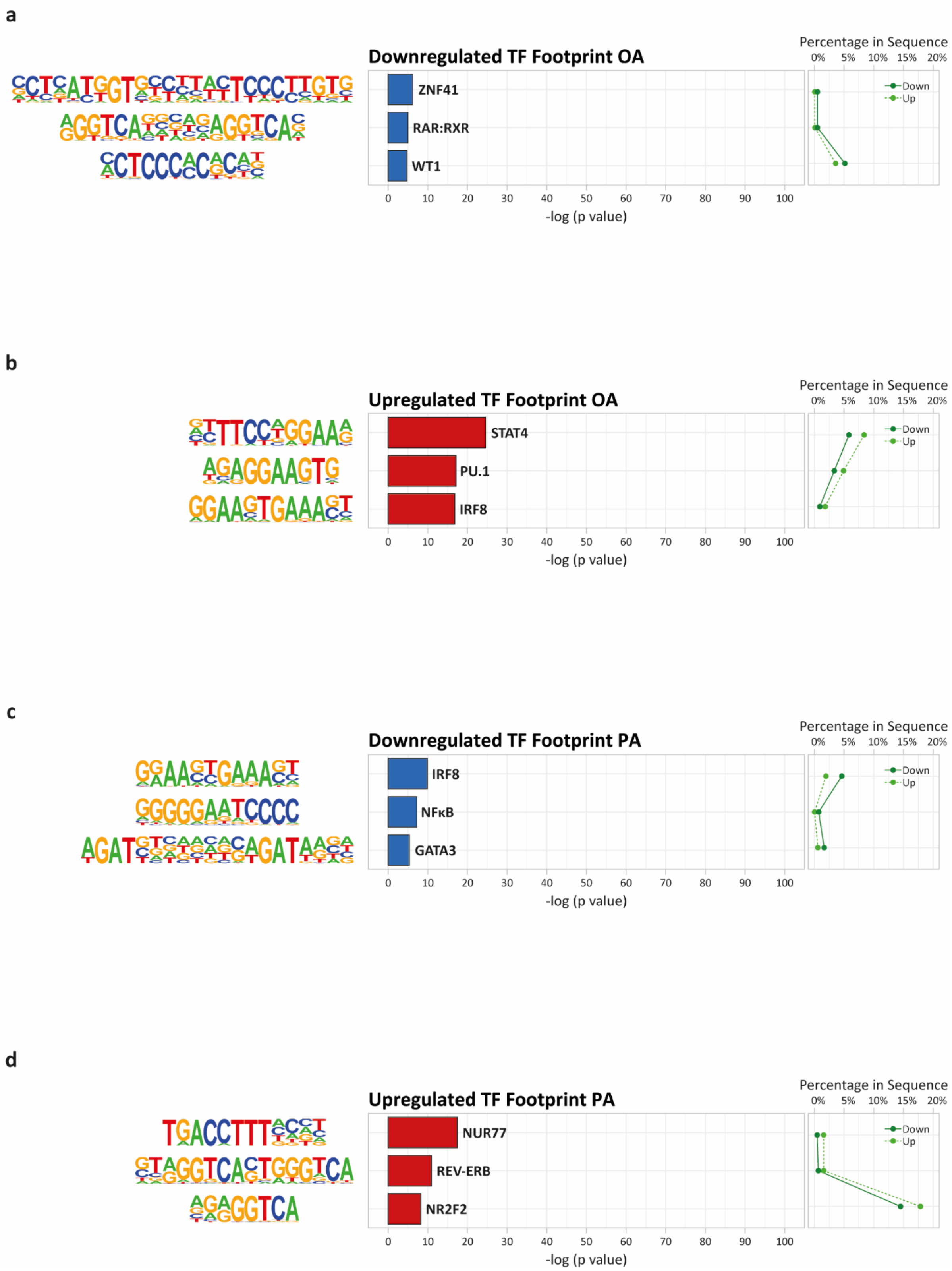
a
Downregulated TF Footprint OA
Percentage in Sequence
0% 5% 10% 15% 20%
Down
Up
ZNF41
RAR:RXR
WT1
0 10 20 30 40 50 60 70 80 90 100
-log (p value)
b
Upregulated TF Footprint OA
Percentage in Sequence
0% 5% 10% 15% 20%
Down
Up
STAT4
PU.1
IRF8
0 10 20 30 40 50 60 70 80 90 100
-log (p value)
c
Downregulated TF Footprint PA
Percentage in Sequence
0% 5% 10% 15% 20%
Down
Up
IRF8
NFκB
GATA3
0 10 20 30 40 50 60 70 80 90 100
-log (p value)
d
Upregulated TF Footprint PA
Percentage in Sequence
0% 5% 10% 15% 20%
Down
Up
NUR77
REV-ERB
NR2F2
0 10 20 30 40 50 60 70 80 90 100
-log (p value)

Supplemental Table 1: Changes in gene expression and CD4$^+$ T cell markers due to EPA, PA, or OA exposure. (a) Downregulated DEGs for EPA exposed non-activated CD4$^+$ T cells in order of significance along with their Ensembl ID, Gene Symbol, Gene Name, UniProt ID, chromosome location, start site, end site, base mean, log2 fold change, log fold change standard error, test statistic, p value, and adjusted p value. **(b)** Upregulated DEGs for EPA exposed non-activated CD4$^+$ T cells in order of significance along with their Ensembl ID, Gene Symbol, Gene Name, UniProt ID, chromosome location, start site, end site, base mean, log2 fold change, log fold change standard error, test statistic, p value, and adjusted p value. **(c)** Downregulated DEGs for OA exposed non-activated CD4$^+$ T cells in order of significance along with their Ensembl ID, Gene Symbol, Gene Name, UniProt ID, chromosome location, start site, end site, base mean, log2 fold change, log fold change standard error, test statistic, p value, and adjusted p value. **(d)** Upregulated DEGs for OA exposed non-activated CD4$^+$ T cells in order of significance along with their Ensembl ID, Gene Symbol, Gene Name, UniProt ID, chromosome location, start site, end site, base mean, log2 fold change, log fold change standard error, test statistic, p value, and adjusted p value. **(e)** Downregulated DEGs for PA exposed non-activated CD4$^+$ T cells in order of significance along with their Ensembl ID, Gene Symbol, Gene Name, UniProt ID, chromosome location, start site, end site, base mean, log2 fold change, log fold change standard error, test statistic, p value, and adjusted p value. **(f)** Upregulated DEGs for PA exposed non-activated CD4$^+$ T cells in order of significance along with their Ensembl ID, Gene Symbol, Gene Name, UniProt ID, chromosome location, start site, end site, base mean, log2 fold change, log fold change standard error, test statistic, p value, and adjusted p value. **(g)** Table showing additional information on the genes that overlap between the different fatty acids. Information on Gene Symbol, Ensembl ID, chromosome location, log2 fold change of that gene in each specific fatty acid, p value of that gene in each specific fatty acid, adjusted p value of that gene in each specific fatty acid, and whether the direction of gene expression is the same in each fatty acid is included. **(h)** All significant pathway enrichment analysis of all downregulated EPA DEGs generated using *clusterProfiler* using 10 human pathway databases. All enriched pathways are shown with information on Pathway Term, Database pathway derives from, number of DEGs that overlap with that databases gene set for that pathway, p value, adjusted p value, gene names of all DEGs included in that pathway, number of DEGs in that pathway that were upregulated, number of DEGs in that pathway that were downregulated, and which group that term clusters into based on the Jaccard index > 0.70. **(i)** All significant pathway enrichment analysis of all upregulated EPA DEGs generated using *clusterProfiler* using 10 human pathway databases. All enriched pathways are shown with information on Pathway Term, Database pathway derives from, number of DEGs that overlap with that databases gene set for that pathway, p value, adjusted p value, gene names of all DEGs included in that pathway, number of DEGs in that pathway that were upregulated, number of DEGs in that pathway that were downregulated, and which group that term clusters into based on the Jaccard index > 0.70. **(j)** Known motif analysis on promotors of down versus upregulated EPA ATAC peaks. All enriched transcription factors are shown in order of significance. Enrichment of transcription factor binding motifs was performed using HOMER. Information on rank, motif, name of transcription factor, p value, log p value, q value (Benjamini), number of target sequences with motif, percent of targets sequences with motif, number of background sequences with motif, and percent of background sequences with motif. **(k)** Known motif analysis on promotors of up versus downregulated EPA ATAC peaks. All enriched transcription factors are shown in order of significance. Enrichment of transcription factor binding motifs was performed using HOMER. Information on rank, motif, name of transcription factor, p value, log p value, q value (Benjamini), number of target sequences with motif, percent of targets sequences with motif, number of background sequences with motif, and percent of background sequences with motif. **(l)** All significant pathway enrichment

analysis of all downregulated OA DEGs generated using *clusterProfiler* using 10 human pathway databases. All enriched pathways are shown with information on Pathway Term, Database pathway derives from, number of DEGs that overlap with that databases gene set for that pathway, p value, adjusted p value, gene names of all DEGs included in that pathway, number of DEGs in that pathway that were upregulated, number of DEGs in that pathway that were downregulated, and which group that term clusters into based on the Jaccard index > 0.70. **(m)** All significant pathway enrichment analysis of all upregulated OA DEGs generated using *clusterProfiler* using 10 human pathway databases. All enriched pathways are shown with information on Pathway Term, Database pathway derives from, number of DEGs that overlap with that databases gene set for that pathway, p value, adjusted p value, gene names of all DEGs included in that pathway, number of DEGs in that pathway that were upregulated, number of DEGs in that pathway that were downregulated, and which group that term clusters into based on the Jaccard index > 0.70. **(n)** All significant pathway enrichment analysis of all downregulated PA DEGs generated using *clusterProfiler* using 10 human pathway databases. All enriched pathways are shown with information on Pathway Term, Database pathway derives from, number of DEGs that overlap with that databases gene set for that pathway, p value, adjusted p value, gene names of all DEGs included in that pathway, number of DEGs in that pathway that were upregulated, number of DEGs in that pathway that were downregulated, and which group that term clusters into based on the Jaccard index > 0.70. **(o)** All significant pathway enrichment analysis of all upregulated PA DEGs generated using *clusterProfiler* using 10 human pathway databases. All enriched pathways are shown with information on Pathway Term, Database pathway derives from, number of DEGs that overlap with that databases gene set for that pathway, p value, adjusted p value, gene names of all DEGs included in that pathway, number of DEGs in that pathway that were upregulated, number of DEGs in that pathway that were downregulated, and which group that term clusters into based on the Jaccard index > 0.70. **(p)** Known motif analysis on promotors of down versus upregulated OA ATAC peaks. All enriched transcription factors are shown in order of significance. Enrichment of transcription factor binding motifs was performed using HOMER. Information on rank, motif, name of transcription factor, p value, log p value, q value (Benjamini), number of target sequences with motif, percent of targets sequences with motif, number of background sequences with motif, and percent of background sequences with motif. **(q)** Known motif analysis on promotors of up versus downregulated OA ATAC peaks. All enriched transcription factors are shown in order of significance. Enrichment of transcription factor binding motifs was performed using HOMER. Information on rank, motif, name of transcription factor, p value, log p value, q value (Benjamini), number of target sequences with motif, percent of targets sequences with motif, number of background sequences with motif, and percent of background sequences with motif. **(r)** Known motif analysis on promotors of down versus upregulated PA ATAC peaks. All enriched transcription factors are shown in order of significance. Enrichment of transcription factor binding motifs was performed using HOMER. Information on rank, motif, name of transcription factor, p value, log p value, q value (Benjamini), number of target sequences with motif, percent of targets sequences with motif, number of background sequences with motif, and percent of background sequences with motif. **(s)** Known motif analysis on promotors of up versus downregulated PA ATAC peaks. All enriched transcription factors are shown in order of significance. Enrichment of transcription factor binding motifs was performed using HOMER. Information on rank, motif, name of transcription factor, p value, log p value, q value (Benjamini), number of target sequences with motif, percent of targets sequences with motif, number of background sequences with motif, and percent of background sequences with motif.

a

Order	Ensembl ID	Gene Symbol	Gene Name	UniProt
1	ENSG00000026025	***VIM***	vimentin	P08670
2	ENSG00000205542	***TMSB4X***	thymosin beta 4 X-linked	P62328
3	ENSG00000204287	***HLA-DRA***	major histocompatibility complex, class II, DR alpha	P01903
4	ENSG00000138326	***RPS24***	ribosomal protein S24	P62847
5	ENSG00000231389	***HLA-DPA1***	major histocompatibility complex, class II, DP alpha 1	P20036
6	ENSG00000026950	***BTN3A1***	butyrophilin subfamily 3 member A1	O00481
7	ENSG00000106952	***TNFSF8***	TNF superfamily member 8	P32971
8	ENSG00000034510	***TMSB10***	thymosin beta 10	P63313
9	ENSG00000118181	***RPS25***	ribosomal protein S25	P62851
10	ENSG00000223865	***HLA-DPB1***	major histocompatibility complex, class II, DP beta 1	P04440
11	ENSG00000186470	***BTN3A2***	butyrophilin subfamily 3 member A2	P78410
12	ENSG00000240065	***PSMB9***	proteasome 20S subunit beta 9	P28065
13	ENSG00000111801	***BTN3A3***	butyrophilin subfamily 3 member A3	O00478
14	ENSG00000100097	***LGALS1***	galectin 1	P09382
15	ENSG00000204592	***HLA-E***	major histocompatibility complex, class I, E	P13747
16	ENSG00000255150	***EID3***	EP300 interacting inhibitor of differentiation 3	Q8N140
17	ENSG00000120833	***SOCS2***	suppressor of cytokine signaling 2	O14508
18	ENSG00000160856	***FCRL3***	Fc receptor like 3	Q96P31
19	ENSG00000019582	***CD74***	CD74 molecule	P04233
20	ENSG00000008517	***IL32***	interleukin 32	P24001
21	ENSG00000185880	***TRIM69***	tripartite motif containing 69	Q86WT6
22	ENSG00000131981	***LGALS3***	galectin 3	P17931
23	ENSG00000113088	***GZMK***	granzyme K	P49863
24	ENSG00000107736	***CDH23***	cadherin related 23	Q9H251
25	ENSG00000243335	***KCTD7***	potassium channel tetramerization domain containing 7	Q96MP8
26	ENSG00000114737	***CISH***	cytokine inducible SH2 containing protein	Q9NSE2
27	ENSG00000196126	***HLA-DRB1***	major histocompatibility complex, class II, DR beta 1	P01911
28	ENSG00000130755	***GMFG***	glia maturation factor gamma	O60234
29	ENSG00000143947	***RPS27A***	ribosomal protein S27a	P62979
30	ENSG00000101608	***MYL12A***	myosin light chain 12A	P19105
31	ENSG00000166710	***B2M***	beta-2-microglobulin	P61769

32	ENSG00000186088	***GSAP***	gamma-secretase activating protein	A4D1B5
33	ENSG00000160191	***PDE9A***	phosphodiesterase 9A	O76083
34	ENSG00000186049	***KRT73***	keratin 73	Q86Y46
35	ENSG00000127585	***FBXL16***	F-box and leucine rich repeat protein 16	Q8N461
36	ENSG00000172794	***RAB37***	RAB37, member RAS oncogene family	P18077
37	ENSG00000182899	***RPL35A***	ribosomal protein L35a	P18077
38	ENSG00000139537	***CCDC65***	coiled-coil domain containing 65	Q8IXS2
39	ENSG00000110848	***CD69***	CD69 molecule	Q07108
40	ENSG00000110665	***C11orf21***	chromosome 11 open reading frame 21	Q9P2W6
41	ENSG00000090382	***LYZ***	lysozyme	P61626
42	ENSG00000160310	***PRMT2***	protein arginine methyltransferase 2	P55345
43	ENSG00000117228	***GBP1***	guanylate binding protein 1	P32455
44	ENSG00000112306	***RPS12***	ribosomal protein S12	P25398
45	ENSG00000150637	***CD226***	CD226 molecule	Q15762
46	ENSG00000185885	***IFITM1***	interferon induced transmembrane protein 1	P13164
47	ENSG00000121807	***CCR2***	C-C motif chemokine receptor 2	P41597
48	ENSG00000197635	***DPP4***	dipeptidyl peptidase 4	P27487
49	ENSG00000168394	***TAP1***	transporter 1, ATP binding cassette subfamily B member	Q03518
50	ENSG00000198840	***MT-ND3***	mitochondrially encoded NADH:ubiquinone oxidoreductase core subunit 3	P03897
51	ENSG00000221890	***NPTXR***	neuronal pentraxin receptor	O95502
52	ENSG00000134419	***RPS15A***	ribosomal protein S15a	P62244
53	ENSG00000071575	***TRIB2***	tribbles pseudokinase 2	Q92519
54	ENSG00000145649	***GZMA***	granzyme A	P12544
55	ENSG00000153283	***CD96***	CD96 molecule	P40200
56	ENSG00000164483	***SAMD3***	sterile alpha motif domain containing 3	Q8N6K7
57	ENSG00000134460	***IL2RA***	interleukin 2 receptor subunit alpha	P01589
58	ENSG00000143167	***GPA33***	glycoprotein A33	Q99795
59	ENSG00000181847	***TIGIT***	T cell immunoreceptor with Ig and ITIM domains	Q495A1
60	ENSG00000179144	***GIMAP7***	GTPase, IMAP family member 7	Q8NHV1
61	ENSG00000167914	***GSDMA***	gasdermin A	Q96QA5
62	ENSG00000150093	***ITGB1***	integrin subunit beta 1	P05556
63	ENSG00000059588	***TARBP1***	TAR (HIV-1) RNA binding protein 1	Q13395
64	ENSG00000129521	***EGLN3***	egl-9 family hypoxia inducible factor 3	Q9H6Z9
65	ENSG00000116574	***RHOU***	ras homolog family member U	Q7L0Q8
66	ENSG00000115232	***ITGA4***	integrin subunit alpha 4	P13612

67	ENSG00000141753	***IGFBP4***	insulin like growth factor binding protein 4	P22692
68	ENSG00000080854	***IGSF9B***	immunoglobulin superfamily member 9B	Q9UPX0
69	ENSG00000119714	***GPR68***	G protein-coupled receptor 68	Q15743
70	ENSG00000092010	***PSME1***	proteasome activator subunit 1	Q06323
71	ENSG00000021300	***PLEKHB1***	pleckstrin homology domain containing B1	Q9UF11
72	ENSG00000156482	***RPL30***	ribosomal protein L30	P62888
73	ENSG00000162909	***CAPN2***	calpain 2	P17655
74	ENSG00000109475	***RPL34***	ribosomal protein L34	P49207
75	ENSG00000110455	***ACCS***	1-aminocyclopropane-1-carboxylate synthase homolog (inactive)	Q96QU6
76	ENSG00000187514	***PTMA***	prothymosin alpha	P06454
77	ENSG00000146285	***SCML4***	Scm polycomb group protein like 4	Q8N228
78	ENSG00000196683	***TOMM7***	translocase of outer mitochondrial membrane 7	Q9P0U1
79	ENSG00000277586	***NEFL***	neurofilament light chain	P07196
80	ENSG00000137154	***RPS6***	ribosomal protein S6	P62753
81	ENSG00000172340	***SUCLG2***	succinate-CoA ligase GDP-forming subunit beta	Q96I99
82	ENSG00000271447	***MMP28***	matrix metallopeptidase 28	Q9H239
83	ENSG00000178573	***MAF***	MAF bZIP transcription factor	O75444
84	ENSG00000125691	***RPL23***	ribosomal protein L23	P62829
85	ENSG00000228474	***OST4***	oligosaccharyltransferase complex subunit 4, non-catalytic	P0C6T2
86	ENSG00000141293	***SKAP1***	src kinase associated phosphoprotein 1	Q86WV1
87	ENSG00000162241	***SLC25A45***	solute carrier family 25 member 45	Q8N413
88	ENSG00000198727	***MT-CYB***	mitochondrially encoded cytochrome b	P00156
89	ENSG00000163191	***S100A11***	S100 calcium binding protein A11	P31949
90	ENSG00000198176	***TFDP1***	transcription factor Dp-1	Q14186
91	ENSG00000147408	***CSGALNACT1***	chondroitin sulfate N-acetylgalactosaminyltransferase 1	Q8TDX6
92	ENSG00000167863	***ATP5PD***	ATP synthase peripheral stalk subunit d	O75947
93	ENSG00000137492	***THAP12***	THAP domain containing 12	O43422
94	ENSG00000169896	***ITGAM***	integrin subunit alpha M	P11215
95	ENSG00000107960	***STN1***	STN1 subunit of CST complex	Q9H668
96	ENSG00000213741	***RPS29***	ribosomal protein S29	P62273
97	ENSG00000197956	***S100A6***	S100 calcium binding protein A6	P06703
98	ENSG00000159496	***RGL4***	ral guanine nucleotide dissociation stimulator like 4	Q8IZJ4
99	ENSG00000071082	***RPL31***	ribosomal protein L31	P62899
100	ENSG00000167768	***KRT1***	keratin 1	P04264
101	ENSG00000204472	***AIF1***	allograft inflammatory factor 1	P55008

102	ENSG00000198502	***HLA-DRB5***	major histocompatibility complex, class II, DR beta 5	Q30154
103	ENSG00000169442	***CD52***	CD52 molecule	P31358
104	ENSG00000171863	***RPS7***	ribosomal protein S7	P62081
105	ENSG00000196735	***HLA-DQA1***	major histocompatibility complex, class II, DQ alpha 1	P01909
106	ENSG00000171858	***RPS21***	ribosomal protein S21	P63220
107	ENSG00000093072	***ADA2***	adenosine deaminase 2	Q9NZK5
108	ENSG00000008988	***RPS20***	ribosomal protein S20	P60866
109	ENSG00000196284	***SUPT3H***	SPT3 homolog, SAGA and STAGA complex component	O75486
110	ENSG00000162777	***DENND2D***	DENN domain containing 2D	Q9H6A0
111	ENSG00000159388	***BTG2***	BTG anti-proliferation factor 2	P78543
112	ENSG00000179583	***CIITA***	class II major histocompatibility complex transactivator	P33076
113	ENSG00000125384	***PTGER2***	prostaglandin E receptor 2	P43116
114	ENSG00000162645	***GBP2***	guanylate binding protein 2	P32456
115	ENSG00000239697	***TNFSF12***	TNF superfamily member 12	O43508
116	ENSG00000186468	***RPS23***	ribosomal protein S23	P62266
117	ENSG00000133321	***PLAAT4***	phospholipase A and acyltransferase 4	Q9UL19
118	ENSG00000100650	***SRSF5***	serine and arginine rich splicing factor 5	Q13243
119	ENSG00000197448	***GSTK1***	glutathione S-transferase kappa 1	Q9Y2Q3
120	ENSG00000168237	***GLYCTK***	glycerate kinase	Q8IVS8
121	ENSG00000142937	***RPS8***	ribosomal protein S8	P62241
122	ENSG00000137752	***CASP1***	caspase 1	P29466
123	ENSG00000242574	***HLA-DMB***	major histocompatibility complex, class II, DM beta	P28068
124	ENSG00000117091	***CD48***	CD48 molecule	P09326
125	ENSG00000099860	***GADD45B***	growth arrest and DNA damage inducible beta	O75293
126	ENSG00000143499	***SMYD2***	SET and MYND domain containing 2	Q9NRG4
127	ENSG00000167613	***LAIR1***	leukocyte associated immunoglobulin like receptor 1	Q6GTX8
128	ENSG00000151692	***RNF144A***	ring finger protein 144A	P50876
129	ENSG00000154814	***OXNAD1***	oxidoreductase NAD binding domain containing 1	Q96HP4
130	ENSG00000161381	***PLXDC1***	plexin domain containing 1	Q8IUK5
131	ENSG00000155265	***GOLGA7B***	golgin A7 family member B	Q2TAP0
132	ENSG00000107833	***NPM3***	nucleophosmin/nucleoplasmin 3	O75607
133	ENSG00000120915	***EPHX2***	epoxide hydrolase 2	P34913
134	ENSG00000126246	***IGFLR1***	IGF like family receptor 1	Q9H665
135	ENSG00000102760	***RGCC***	regulator of cell cycle	Q9H4X1
136	ENSG00000111319	***SCNN1A***	sodium channel epithelial 1 subunit alpha	P37088

137	ENSG00000102524	***TNFSF13B***	TNF superfamily member 13b	Q9Y275
138	ENSG00000124172	***ATP5F1E***	ATP synthase F1 subunit epsilon	P56381
139	ENSG00000177409	***SAMD9L***	sterile alpha motif domain containing 9 like	Q8IVG5
140	ENSG00000117616	***RSRP1***	arginine and serine rich protein 1	Q9BUV0
141	ENSG00000184613	***NELL2***	neural EGFL like 2	Q99435
142	ENSG00000110934	***BIN2***	bridging integrator 2	Q9UBW5
143	ENSG00000197622	***CDC42SE1***	CDC42 small effector 1	Q9NRR8
144	ENSG00000204681	***GABBR1***	gamma-aminobutyric acid type B receptor subunit 1	Q9UBS5
145	ENSG00000106560	***GIMAP2***	GTPase, IMAP family member 2	Q9UG22
146	ENSG00000158869	***FCER1G***	Fc epsilon receptor Ig	P30273
147	ENSG00000189043	***NDUFA4***	NDUFA4 mitochondrial complex associated	O00483
148	ENSG00000110700	***RPS13***	ribosomal protein S13	P62277
149	ENSG00000204475	***NCR3***	natural cytotoxicity triggering receptor 3	O14931
150	ENSG00000122026	***RPL21***	ribosomal protein L21	P46778
151	ENSG00000125743	***SNRPD2***	small nuclear ribonucleoprotein D2 polypeptide	P62316
152	ENSG00000196154	***S100A4***	S100 calcium binding protein A4	P26447
153	ENSG00000266472	***MRPS21***	mitochondrial ribosomal protein S21	P82921
154	ENSG00000154760	***SLFN13***	schlafen family member 13	Q68D06
155	ENSG00000133574	***GIMAP4***	GTPase, IMAP family member 4	Q9NUV9
156	ENSG00000139679	***LPAR6***	lysophosphatidic acid receptor 6	P43657
157	ENSG00000128951	***DUT***	deoxyuridine triphosphatase	P33316
158	ENSG00000281106	***TMEM272***	transmembrane protein 272	A0A1B0GTI8
159	ENSG00000005102	***MEOX1***	mesenchyme homeobox 1	P50221
160	ENSG00000153563	***CD8A***	CD8 subunit alpha	P01732
161	ENSG00000145592	***RPL37***	ribosomal protein L37	P61927
162	ENSG00000232112	***TMA7***	translation machinery associated 7 homolog	Q9Y2S6
163	ENSG00000111335	***OAS2***	2'-5'-oligoadenylate synthetase 2	P29728
164	ENSG00000228253	***MT-ATP8***	mitochondrially encoded ATP synthase membrane subunit 8	P03928
165	ENSG00000143384	***MCL1***	MCL1 apoptosis regulator, BCL2 family member	Q07820
166	ENSG00000133048	***CHI3L1***	chitinase 3 like 1	P36222
167	ENSG00000160791	***CCR5***	C-C motif chemokine receptor 5	P51681
168	ENSG00000198242	***RPL23A***	ribosomal protein L23a	P62750
169	ENSG00000142676	***RPL11***	ribosomal protein L11	P62913
170	ENSG00000102245	***CD40LG***	CD40 ligand	P29965
171	ENSG00000156738	***MS4A1***	membrane spanning 4-domains A1	P11836

172	ENSG00000256043	***CTSO***	cathepsin O	P43234
173	ENSG00000198467	***TPM2***	tropomyosin 2	P07951
174	ENSG00000106603	***COA1***	cytochrome c oxidase assembly factor 1	Q9GZY4
175	ENSG00000177721	***ANXA2R***	annexin A2 receptor	Q3ZCQ2
176	ENSG00000177954	***RPS27***	ribosomal protein S27	P42677
177	ENSG00000135596	***MICAL1***	microtubule associated monooxygenase, calponin and LIM domain containing 1	Q8TDZ2
178	ENSG00000156467	***UQCRB***	ubiquinol-cytochrome c reductase binding protein	P14927
179	ENSG00000139714	***MORN3***	MORN repeat containing 3	Q6PF18
180	ENSG00000111863	***ADTRP***	androgen dependent TFPI regulating protein	Q96IZ2
181	ENSG00000082074	***FYB1***	FYN binding protein 1	O15117
182	ENSG00000144713	***RPL32***	ribosomal protein L32	P62910
183	ENSG00000197540	***GZMM***	granzyme M	P51124
184	ENSG00000188641	***DPYD***	dihydropyrimidine dehydrogenase	Q12882
185	ENSG00000166508	***MCM7***	minichromosome maintenance complex component 7	P33993
186	ENSG00000160991	***ORAI2***	ORAI calcium release-activated calcium modulator 2	Q96SN7
187	ENSG00000275835	***TUBGCP5***	tubulin gamma complex associated protein 5	Q96RT8
188	ENSG00000172005	***MAL***	mal, T cell differentiation protein	P21145
189	ENSG00000128284	***APOL3***	apolipoprotein L3	O95236
190	ENSG00000179715	***PCED1B***	PC-esterase domain containing 1B	Q96HM7
191	ENSG00000131469	***RPL27***	ribosomal protein L27	P61353
192	ENSG00000166750	***SLFN5***	schlafen family member 5	Q08AF3
193	ENSG00000145425	***RPS3A***	ribosomal protein S3A	P61247
194	ENSG00000112303	***VNN2***	vanin 2	O95498
195	ENSG00000116824	***CD2***	CD2 molecule	P06729
196	ENSG00000125430	***HS3ST3B1***	heparan sulfate-glucosamine 3-sulfotransferase 3B1	Q9Y662
197	ENSG00000189060	***H1-0***	H1.0 linker histone	P07305
198	ENSG00000182183	***SHISAL2A***	shisa like 2A	Q6UWV7
199	ENSG00000119403	***PHF19***	PHD finger protein 19	Q5T6S3
200	ENSG00000009790	***TRAF3IP3***	TRAF3 interacting protein 3	Q9Y228
201	ENSG00000213366	***GSTM2***	glutathione S-transferase mu 2	P28161
202	ENSG00000213719	***CLIC1***	chloride intracellular channel 1	O00299
203	ENSG00000010810	***FYN***	FYN proto-oncogene, Src family tyrosine kinase	P06241
204	ENSG00000187554	***TLR5***	toll like receptor 5	O60602
205	ENSG00000133030	***MPRIP***	myosin phosphatase Rho interacting protein	Q6WCQ1
206	ENSG00000204219	***TCEA3***	transcription elongation factor A3	O75764

207	ENSG00000154027	***AK5***	adenylate kinase 5	Q9Y6K8
208	ENSG00000204397	***CARD16***	caspase recruitment domain family member 16	Q5EG05
209	ENSG00000184730	***APOBR***	apolipoprotein B receptor	Q0VD83
210	ENSG00000147168	***IL2RG***	interleukin 2 receptor subunit gamma	P31785
211	ENSG00000265972	***TXNIP***	thioredoxin interacting protein	Q9H3M7
212	ENSG00000163131	***CTSS***	cathepsin S	P25774
213	ENSG00000122986	***HVCN1***	hydrogen voltage gated channel 1	Q96D96
214	ENSG00000134830	***C5AR2***	complement C5a receptor 2	Q9P296
215	ENSG00000154451	***GBP5***	guanylate binding protein 5	Q96PP8
216	ENSG00000089041	***P2RX7***	purinergic receptor P2X 7	Q99572
217	ENSG00000239713	***APOBEC3G***	apolipoprotein B mRNA editing enzyme catalytic subunit 3G	Q9HC16
218	ENSG00000166432	***ZMAT1***	zinc finger matrin-type 1	Q5H9K5
219	ENSG00000244509	***APOBEC3C***	apolipoprotein B mRNA editing enzyme catalytic subunit 3C	Q9NRW3
220	ENSG00000077238	***IL4R***	interleukin 4 receptor	P24394
221	ENSG00000114942	***EEF1B2***	eukaryotic translation elongation factor 1 beta 2	P24534
222	ENSG00000053371	***AKR7A2***	aldo-keto reductase family 7 member A2	O43488
223	ENSG00000258728	***ENSG00000258728***	novel protein (GALT-IL11RA readthrough)	
224	ENSG00000154217	***PITPNC1***	phosphatidylinositol transfer protein cytoplasmic 1	Q9UKF7
225	ENSG00000182463	***TSHZ2***	teashirt zinc finger homeobox 2	Q9NRE2
226	ENSG00000215845	***TSTD1***	thiosulfate sulfurtransferase like domain containing 1	Q8NFU3
227	ENSG00000198918	***RPL39***	ribosomal protein L39	P62891
228	ENSG00000102738	***MRPS31***	mitochondrial ribosomal protein S31	Q92665
229	ENSG00000042493	***CAPG***	capping actin protein, gelsolin like	P40121
230	ENSG00000129675	***ARHGEF6***	Rac/Cdc42 guanine nucleotide exchange factor 6	Q15052
231	ENSG00000107317	***PTGDS***	prostaglandin D2 synthase	P41222
232	ENSG00000125148	***MT2A***	metallothionein 2A	P02795
233	ENSG00000183087	***GAS6***	growth arrest specific 6	Q14393
234	ENSG00000103187	***COTL1***	coactosin like F-actin binding protein 1	Q14019
235	ENSG00000149177	***PTPRJ***	protein tyrosine phosphatase receptor type J	Q12913
236	ENSG00000109452	***INPP4B***	inositol polyphosphate-4-phosphatase type II B	O15327
237	ENSG00000128928	***IVD***	isovaleryl-CoA dehydrogenase	P26440
238	ENSG00000154079	***SDHAF4***	succinate dehydrogenase complex assembly factor 4	Q5VUM1
239	ENSG00000198520	***ARMH1***	armadillo like helical domain containing 1	Q6PIY5
240	ENSG00000165168	***CYBB***	cytochrome b-245 beta chain	P04839
241	ENSG00000072818	***ACAP1***	ArfGAP with coiled-coil, ankyrin repeat and PH domains 1	Q15027

242	ENSG00000075142	***SRI***	sorcin	P30626
243	ENSG00000172824	***CES4A***	carboxylesterase 4A	Q5XG92
244	ENSG00000102007	***PLP2***	proteolipid protein 2	Q04941
245	ENSG00000140465	***CYP1A1***	cytochrome P450 family 1 subfamily A member 1	P04798
246	ENSG00000092841	***MYL6***	myosin light chain 6	P60660
247	ENSG00000149292	***TTC12***	tetratricopeptide repeat domain 12	Q9H892
248	ENSG00000172809	***RPL38***	ribosomal protein L38	P63173
249	ENSG00000129103	***SUMF2***	sulfatase modifying factor 2	Q8NBJ7
250	ENSG00000189067	***LITAF***	lipopolysaccharide induced TNF factor	Q99732
251	ENSG00000183172	***SMDT1***	single-pass membrane protein with aspartate rich tail 1	Q9H4I9
252	ENSG00000168899	***VAMP5***	vesicle associated membrane protein 5	O95183
253	ENSG00000204264	***PSMB8***	proteasome 20S subunit beta 8	P28062
254	ENSG00000167286	***CD3D***	CD3 delta subunit of T-cell receptor complex	P04234
255	ENSG00000196646	***ZNF136***	zinc finger protein 136	P52737
256	ENSG00000184900	***SUMO3***	small ubiquitin like modifier 3	P55854
257	ENSG00000198668	***CALM1***	calmodulin 1	P0DP23
258	ENSG00000171476	***HOPX***	HOP homeobox	Q9BPY8
259	ENSG00000234745	***HLA-B***	major histocompatibility complex, class I, B	P01889
260	ENSG00000154102	***C16orf74***	chromosome 16 open reading frame 74	Q96GX8
261	ENSG00000170915	***PAQR8***	progestin and adipoQ receptor family member 8	Q8TEZ7
262	ENSG00000100450	***GZMH***	granzyme H	P20718
263	ENSG00000197958	***RPL12***	ribosomal protein L12	P30050
264	ENSG00000173114	***LRRN3***	leucine rich repeat neuronal 3	Q9H3W5
265	ENSG00000204642	***HLA-F***	major histocompatibility complex, class I, F	P30511
266	ENSG00000148908	***RGS10***	regulator of G protein signaling 10	O43665
267	ENSG00000185650	***ZFP36L1***	ZFP36 ring finger protein like 1	Q07352
268	ENSG00000143502	***SUSD4***	sushi domain containing 4	Q5VX71
269	ENSG00000122034	***GTF3A***	general transcription factor IIIA	Q92664
270	ENSG00000188404	***SELL***	selectin L	P14151
271	ENSG00000070081	***NUCB2***	nucleobindin 2	P80303
272	ENSG00000243317	***STMP1***	short transmembrane mitochondrial protein 1	E0CX11
273	ENSG00000169241	***SLC50A1***	solute carrier family 50 member 1	Q9BRV3
274	ENSG00000157654	***PALM2AKAP2***	PALM2 and AKAP2 fusion	
275	ENSG00000180902	***D2HGDH***	D-2-hydroxyglutarate dehydrogenase	Q8N465
276	ENSG00000184384	***MAML2***	mastermind like transcriptional coactivator 2	Q8IZL2

277	ENSG00000145982	***FARS2***	phenylalanyl-tRNA synthetase 2, mitochondrial	O95363
278	ENSG00000145569	***OTULINL***	OTU deubiquitinase with linear linkage specificity like	Q9NUU6
279	ENSG00000116771	***AGMAT***	agmatinase	Q9BSE5
280	ENSG00000115875	***SRSF7***	serine and arginine rich splicing factor 7	Q16629
281	ENSG00000102317	***RBM3***	RNA binding motif protein 3	P98179
282	ENSG00000126882	***FAM78A***	family with sequence similarity 78 member A	Q5JUQ0
283	ENSG00000196531	***NACA***	nascent polypeptide associated complex subunit alpha	Q13765
284	ENSG00000069275	***NUCKS1***	nuclear casein kinase and cyclin dependent kinase substrate 1	Q9H1E3
285	ENSG00000132530	***XAF1***	XIAP associated factor 1	Q6GPH4
286	ENSG00000180096	***SEPTIN1***	septin 1	Q8WYJ6
287	ENSG00000163823	***CCR1***	C-C motif chemokine receptor 1	P32246
288	ENSG00000028137	***TNFRSF1B***	TNF receptor superfamily member 1B	P20333
289	ENSG00000100351	***GRAP2***	GRB2 related adaptor protein 2	O75791
290	ENSG00000187994	***RINL***	Ras and Rab interactor like	Q6ZS11
291	ENSG00000079263	***SP140***	SP140 nuclear body protein	Q13342
292	ENSG00000133678	***TMEM254***	transmembrane protein 254	Q8TBM7
293	ENSG00000100385	***IL2RB***	interleukin 2 receptor subunit beta	P14784
294	ENSG00000171148	***TADA3***	transcriptional adaptor 3	O75528
295	ENSG00000056558	***TRAF1***	TNF receptor associated factor 1	Q13077
296	ENSG00000186854	***TRABD2A***	TraB domain containing 2A	Q86V40
297	ENSG00000075826	***SEC31B***	SEC31 homolog B, COPII coat complex component	Q9NQW1
298	ENSG00000077549	***CAPZB***	capping actin protein of muscle Z-line subunit beta	P47756
299	ENSG00000116288	***PARK7***	Parkinsonism associated deglycase	Q99497
300	ENSG00000126264	***HCST***	hematopoietic cell signal transducer	Q9UBK5
301	ENSG00000167257	***RNF214***	ring finger protein 214	Q8ND24
302	ENSG00000103489	***XYLT1***	xylosyltransferase 1	Q86Y38
303	ENSG00000010610	***CD4***	CD4 molecule	P01730
304	ENSG00000132749	***TESMIN***	testis expressed metallothionein like protein	Q9Y4I5
305	ENSG00000179344	***HLA-DQB1***	major histocompatibility complex, class II, DQ beta 1	P01920
306	ENSG00000149600	***COMMD7***	COMM domain containing 7	Q86VX2
307	ENSG00000136235	***GPNMB***	glycoprotein nmb	Q14956
308	ENSG00000170430	***MGMT***	O-6-methylguanine-DNA methyltransferase	P16455
309	ENSG00000173917	***HOXB2***	homeobox B2	P14652
310	ENSG00000165929	***TC2N***	tandem C2 domains, nuclear	Q8N9U0
311	ENSG00000131378	***RFTN1***	raftlin, lipid raft linker 1	Q14699

312	ENSG00000160593	***JAML***	junction adhesion molecule like	Q86YT9
313	ENSG00000133943	***DGLUCY***	D-glutamate cyclase	Q7Z3D6
314	ENSG00000115840	***SLC25A12***	solute carrier family 25 member 12	O75746
315	ENSG00000136286	***MYO1G***	myosin IG	B0I1T2
316	ENSG00000132475	***H3-3B***	H3.3 histone B	P84243
317	ENSG00000128340	***RAC2***	Rac family small GTPase 2	P15153
318	ENSG00000149970	***CNKSR2***	connector enhancer of kinase suppressor of Ras 2	Q8WXI2
319	ENSG00000152465	***NMT2***	N-myristoyltransferase 2	O60551
320	ENSG00000146674	***IGFBP3***	insulin like growth factor binding protein 3	P17936
321	ENSG00000167766	***ZNF83***	zinc finger protein 83	P51522
322	ENSG00000186469	***GNG2***	G protein subunit gamma 2	P59768
323	ENSG00000145882	***PCYOX1L***	prenylcysteine oxidase 1 like	Q8NBM8
324	ENSG00000135318	***NT5E***	5'-nucleotidase ecto	P21589
325	ENSG00000182472	***CAPN12***	calpain 12	Q6ZSI9
326	ENSG00000198168	***SVIP***	small VCP interacting protein	Q8NHG7
327	ENSG00000138449	***SLC40A1***	solute carrier family 40 member 1	Q9NP59
328	ENSG00000180822	***PSMG4***	proteasome assembly chaperone 4	Q5JS54
329	ENSG00000104783	***KCNN4***	potassium calcium-activated channel subfamily N member 4	O15554
330	ENSG00000133639	***BTG1***	BTG anti-proliferation factor 1	P62324
331	ENSG00000119408	***NEK6***	NIMA related kinase 6	Q9HC98
332	ENSG00000174444	***RPL4***	ribosomal protein L4	P36578
333	ENSG00000198932	***GPRASP1***	G protein-coupled receptor associated sorting protein 1	Q5JY77
334	ENSG00000163584	***RPL22L1***	ribosomal protein L22 like 1	Q6P5R6
335	ENSG00000213658	***LAT***	linker for activation of T cells	O43561
336	ENSG00000198938	***MT-CO3***	mitochondrially encoded cytochrome c oxidase III	P00414
337	ENSG00000204228	***HSD17B8***	hydroxysteroid 17-beta dehydrogenase 8	Q92506
338	ENSG00000204252	***HLA-DOA***	major histocompatibility complex, class II, DO alpha	P06340
339	ENSG00000109943	***CRTAM***	cytotoxic and regulatory T cell molecule	O95727
340	ENSG00000005844	***ITGAL***	integrin subunit alpha L	P20701
341	ENSG00000169020	***ATP5ME***	ATP synthase membrane subunit e	P56385
342	ENSG00000136929	***HEMGN***	hemogen	Q9BXL5
343	ENSG00000150593	***PDCD4***	programmed cell death 4	Q53EL6
344	ENSG00000140853	***NLRC5***	NLR family CARD domain containing 5	Q86WI3
345	ENSG00000198755	***RPL10A***	ribosomal protein L10a	P62906
346	ENSG00000134765	***DSC1***	desmocollin 1	Q08554

347	ENSG00000178773	***CPNE7***	copine 7	Q9UBL6
348	ENSG00000104886	***PLEKHJ1***	pleckstrin homology domain containing J1	Q9NW61
349	ENSG00000171843	***MLLT3***	MLLT3 super elongation complex subunit	P42568
350	ENSG00000126756	***UXT***	ubiquitously expressed prefoldin like chaperone	Q9UBK9
351	ENSG00000139193	***CD27***	CD27 molecule	P26842
352	ENSG00000131634	***TMEM204***	transmembrane protein 204	Q9BSN7
353	ENSG00000100911	***PSME2***	proteasome activator subunit 2	Q9UL46
354	ENSG00000167077	***MEI1***	meiotic double-stranded break formation protein 1	Q5TIA1
355	ENSG00000140395	***WDR61***	SKI8 subunit of superkiller complex	Q9GZS3
356	ENSG00000170486	***KRT72***	keratin 72	Q14CN4
357	ENSG00000156411	***ATP5MJ***	ATP synthase membrane subunit j	P56378
358	ENSG00000204267	***TAP2***	transporter 2, ATP binding cassette subfamily B member	Q03519
359	ENSG00000229117	***RPL41***	ribosomal protein L41	P62945
360	ENSG00000113532	***ST8SIA4***	ST8 alpha-N-acetyl-neuraminide alpha-2,8-sialyltransferase 4	Q92187
361	ENSG00000165617	***DACT1***	dishevelled binding antagonist of beta catenin 1	Q9NYF0
362	ENSG00000163297	***ANTXR2***	ANTXR cell adhesion molecule 2	P58335
363	ENSG00000105088	***OLFM2***	olfactomedin 2	O95897
364	ENSG00000107902	***LHPP***	phospholysine phosphohistidine inorganic pyrophosphate phosphatase	Q9H008
365	ENSG00000043462	***LCP2***	lymphocyte cytosolic protein 2	Q13094
366	ENSG00000213203	***GIMAP1***	GTPase, IMAP family member 1	Q8WWP7
367	ENSG00000107672	***NSMCE4A***	NSE4 homolog A, SMC5-SMC6 complex component	Q9NXX6
368	ENSG00000136161	***RCBTB2***	RCC1 and BTB domain containing protein 2	O95199
369	ENSG00000164182	***NDUFAF2***	NADH:ubiquinone oxidoreductase complex assembly factor 2	Q8N183
370	ENSG00000206503	***HLA-A***	major histocompatibility complex, class I, A	P04439
371	ENSG00000182162	***P2RY8***	P2Y receptor family member 8	Q86VZ1
372	ENSG00000028277	***POU2F2***	POU class 2 homeobox 2	P09086
373	ENSG00000011600	***TYROBP***	transmembrane immune signaling adaptor TYROBP	O43914
374	ENSG00000253719	***ATXN7L3B***	ataxin 7 like 3B	Q96GX2
375	ENSG00000161791	***FMNL3***	formin like 3	Q8IVF7
376	ENSG00000176049	***JAKMIP2***	janus kinase and microtubule interacting protein 2	Q96AA8
377	ENSG00000110063	***DCPS***	decapping enzyme, scavenger	Q96C86
378	ENSG00000143365	***RORC***	RAR related orphan receptor C	P51449
379	ENSG00000197283	***SYNGAP1***	synaptic Ras GTPase activating protein 1	Q96PV0
380	ENSG00000007944	***MYLIP***	myosin regulatory light chain interacting protein	Q8WY64
381	ENSG00000183662	***TAFA1***	TAFA chemokine like family member 1	Q7Z5A9

382	ENSG00000197756	***RPL37A***	ribosomal protein L37a	P61513
383	ENSG00000166598	***HSP90B1***	heat shock protein 90 beta family member 1	P14625
384	ENSG00000134996	***OSTF1***	osteoclast stimulating factor 1	Q92882
385	ENSG00000186517	***ARHGAP30***	Rho GTPase activating protein 30	Q7Z6I6
386	ENSG00000105374	***NKG7***	natural killer cell granule protein 7	Q16617
387	ENSG00000163106	***HPGDS***	hematopoietic prostaglandin D synthase	O60760
388	ENSG00000133985	***TTC9***	tetratricopeptide repeat domain 9	Q92623
389	ENSG00000146701	***MDH2***	malate dehydrogenase 2	P40926
390	ENSG00000198888	***MT-ND1***	mitochondrially encoded NADH:ubiquinone oxidoreductase core subunit 1	P03886
391	ENSG00000124942	***AHNAK***	AHNAK nucleoprotein	Q09666
392	ENSG00000125354	***SEPTIN6***	septin 6	Q14141
393	ENSG00000183691	***NOG***	noggin	Q13253
394	ENSG00000166801	***FAM111A***	FAM111 trypsin like peptidase A	Q96PZ2
395	ENSG00000104852	***SNRNP70***	small nuclear ribonucleoprotein U1 subunit 70	P08621
396	ENSG00000143224	***PPOX***	protoporphyrinogen oxidase	P50336
397	ENSG00000155368	***DBI***	diazepam binding inhibitor, acyl-CoA binding protein	P07108
398	ENSG00000005513	***SOX8***	SRY-box transcription factor 8	P57073
399	ENSG00000144579	***CTDSP1***	CTD small phosphatase 1	Q9GZU7
400	ENSG00000116157	***GPX7***	glutathione peroxidase 7	Q96SL4
401	ENSG00000103415	***HMOX2***	heme oxygenase 2	P30519
402	ENSG00000196262	***PPIA***	peptidylprolyl isomerase A	P62937
403	ENSG00000099622	***CIRBP***	cold inducible RNA binding protein	Q14011
404	ENSG00000104325	***DECR1***	2,4-dienoyl-CoA reductase 1	Q16698
405	ENSG00000183688	***RFLNB***	refilin B	Q8N5W9
406	ENSG00000163682	***RPL9***	ribosomal protein L9	P32969
407	ENSG00000115523	***GNLY***	granulysin	P22749
408	ENSG00000108654	***DDX5***	DEAD-box helicase 5	P17844
409	ENSG00000162591	***MEGF6***	multiple EGF like domains 6	O75095
410	ENSG00000101040	***ZMYND8***	zinc finger MYND-type containing 8	Q9ULU4
411	ENSG00000089220	***PEBP1***	phosphatidylethanolamine binding protein 1	P30086
412	ENSG00000122694	***GLIPR2***	GLI pathogenesis related 2	Q9H4G4
413	ENSG00000111913	***RIPOR2***	RHO family interacting cell polarization regulator 2	Q9Y4F9
414	ENSG00000134954	***ETS1***	ETS proto-oncogene 1, transcription factor	P14921
415	ENSG00000102575	***ACP5***	acid phosphatase 5, tartrate resistant	P13686
416	ENSG00000115866	***DARS1***	aspartyl-tRNA synthetase 1	P14868

417	ENSG00000172985	***SH3RF3***	SH3 domain containing ring finger 3	Q8TEJ3
418	ENSG00000168329	***CX3CR1***	C-X3-C motif chemokine receptor 1	P49238
419	ENSG00000143870	***PDIA6***	protein disulfide isomerase family A member 6	Q15084
420	ENSG00000135960	***EDAR***	ectodysplasin A receptor	Q9UNE0
421	ENSG00000106733	***NMRK1***	nicotinamide riboside kinase 1	Q9NWW6
422	ENSG00000198763	***MT-ND2***	mitochondrially encoded NADH:ubiquinone oxidoreductase core subunit 2	P03891
423	ENSG00000134594	***RAB33A***	RAB33A, member RAS oncogene family	Q14088
424	ENSG00000141556	***TBCD***	tubulin folding cofactor D	Q9BTW9
425	ENSG00000196950	***SLC39A10***	solute carrier family 39 member 10	Q9ULF5
426	ENSG00000171848	***RRM2***	ribonucleotide reductase regulatory subunit M2	P31350
427	ENSG00000140511	***HAPLN3***	hyaluronan and proteoglycan link protein 3	Q96S86
428	ENSG00000167528	***ZNF641***	zinc finger protein 641	Q96N77
429	ENSG00000124256	***ZBP1***	Z-DNA binding protein 1	Q9H171
430	ENSG00000137312	***FLOT1***	flotillin 1	O75955
431	ENSG00000261371	***PECAM1***	platelet and endothelial cell adhesion molecule 1	P16284
432	ENSG00000126247	***CAPNS1***	calpain small subunit 1	P04632
433	ENSG00000143409	***MINDY1***	MINDY lysine 48 deubiquitinase 1	Q8N5J2
434	ENSG00000082512	***TRAF5***	TNF receptor associated factor 5	O00463
435	ENSG00000104324	***CPQ***	carboxypeptidase Q	Q9Y646
436	ENSG00000205268	***PDE7A***	phosphodiesterase 7A	Q13946
437	ENSG00000167664	***TMIGD2***	transmembrane and immunoglobulin domain containing 2	Q96BF3
438	ENSG00000189403	***HMGB1***	high mobility group box 1	P09429
439	ENSG00000215788	***TNFRSF25***	TNF receptor superfamily member 25	Q93038
440	ENSG00000124193	***SRSF6***	serine and arginine rich splicing factor 6	Q13247
441	ENSG00000198899	***MT-ATP6***	mitochondrially encoded ATP synthase membrane subunit 6	P00846
442	ENSG00000198851	***CD3E***	CD3 epsilon subunit of T-cell receptor complex	P07766
443	ENSG00000007392	***LUC7L***	LUC7 like	Q9NQ29
444	ENSG00000148484	***RSU1***	Ras suppressor protein 1	Q15404
445	ENSG00000125347	***IRF1***	interferon regulatory factor 1	P10914
446	ENSG00000120925	***RNF170***	ring finger protein 170	Q96K19
447	ENSG00000171130	***ATP6V0E2***	ATPase H+ transporting V0 subunit e2	Q8NHE4
448	ENSG00000002822	***MAD1L1***	mitotic arrest deficient 1 like 1	Q9Y6D9
449	ENSG00000174500	***GCSAM***	germinal center associated signaling and motility	Q8N6F7
450	ENSG00000111348	***ARHGDIB***	Rho GDP dissociation inhibitor beta	P52566
451	ENSG00000008283	***CYB561***	cytochrome b561	P49447

452	ENSG00000117643	***MAN1C1***	mannosidase alpha class 1C member 1	Q9NR34
453	ENSG00000160213	***CSTB***	cystatin B	P04080
454	ENSG00000112782	***CLIC5***	chloride intracellular channel 5	Q9NZA1
455	ENSG00000118507	***AKAP7***	A-kinase anchoring protein 7	Q9P0M2
456	ENSG00000198886	***MT-ND4***	mitochondrially encoded NADH:ubiquinone oxidoreductase core subunit 4	P03905
457	ENSG00000183397	***TEKTIP1***	tektin bundle interacting protein 1	A6NCJ1
458	ENSG00000213626	***LBH***	LBH regulator of WNT signaling pathway	Q53QV2
459	ENSG00000185745	***IFIT1***	interferon induced protein with tetratricopeptide repeats 1	P09914
460	ENSG00000104133	***SPG11***	SPG11 vesicle trafficking associated, spatacsin	Q96JI7
461	ENSG00000171530	***TBCA***	tubulin folding cofactor A	O75347
462	ENSG00000167851	***CD300A***	CD300a molecule	Q9UGN4
463	ENSG00000122406	***RPL5***	ribosomal protein L5	P46777
464	ENSG00000160255	***ITGB2***	integrin subunit beta 2	P05107
465	ENSG00000155158	***TTC39B***	tetratricopeptide repeat domain 39B	Q5VTQ0
466	ENSG00000105953	***OGDH***	oxoglutarate dehydrogenase	Q02218
467	ENSG00000164530	***PI16***	peptidase inhibitor 16	Q6UXB8
468	ENSG00000170846	***ENSG00000170846***	novel protein, similar to Morf4 family associated protein 1	B2RBV5
469	ENSG00000135272	***MDFIC***	MyoD family inhibitor domain containing	Q9P1T7
470	ENSG00000185043	***CIB1***	calcium and integrin binding 1	Q99828
471	ENSG00000189283	***FHIT***	fragile histidine triad diadenosine triphosphatase	P49789
472	ENSG00000171791	***BCL2***	BCL2 apoptosis regulator	P10415
473	ENSG00000149311	***ATM***	ATM serine/threonine kinase	Q13315
474	ENSG00000138640	***FAM13A***	family with sequence similarity 13 member A	O94988
475	ENSG00000135362	***PRR5L***	proline rich 5 like	Q6MZQ0
476	ENSG00000164398	***ACSL6***	acyl-CoA synthetase long chain family member 6	Q9UKU0
477	ENSG00000143933	***CALM2***	calmodulin 2	P0DP24
478	ENSG00000107949	***BCCIP***	BRCA2 and CDKN1A interacting protein	Q9P287
479	ENSG00000135074	***ADAM19***	ADAM metallopeptidase domain 19	Q9H013
480	ENSG00000070831	***CDC42***	cell division cycle 42	P60953
481	ENSG00000188917	***TRMT2B***	tRNA methyltransferase 2 homolog B	Q96GJ1
482	ENSG00000165502	***RPL36AL***	ribosomal protein L36a like	Q969Q0
483	ENSG00000189233	***NUGGC***	nuclear GTPase, germinal center associated	Q68CJ6
484	ENSG00000132109	***TRIM21***	tripartite motif containing 21	P19474
485	ENSG00000122543	***OCM***	oncomodulin	P0CE72
486	ENSG00000127922	***SEM1***	SEM1 26S proteasome subunit	Q6ZVN7

487	ENSG00000131370	***SH3BP5***	SH3 domain binding protein 5	O60239
488	ENSG00000133561	***GIMAP6***	GTPase, IMAP family member 6	Q6P9H5
489	ENSG00000172922	***RNASEH2C***	ribonuclease H2 subunit C	Q8TDP1
490	ENSG00000130638	***ATXN10***	ataxin 10	Q9UBB4
491	ENSG00000072135	***PTPN18***	protein tyrosine phosphatase non-receptor type 18	Q99952
492	ENSG00000114391	***RPL24***	ribosomal protein L24	P83731
493	ENSG00000100100	***PIK3IP1***	phosphoinositide-3-kinase interacting protein 1	Q96FE7
494	ENSG00000089009	***RPL6***	ribosomal protein L6	Q02878
495	ENSG00000242259	***C22orf39***	chromosome 22 open reading frame 39	Q6P5X5
496	ENSG00000204525	***HLA-C***	major histocompatibility complex, class I, C	P10321
497	ENSG00000178732	***GP5***	glycoprotein V platelet	P40197
498	ENSG00000110324	***IL10RA***	interleukin 10 receptor subunit alpha	Q13651
499	ENSG00000132424	***PNISR***	PNN interacting serine and arginine rich protein	Q8TF01
500	ENSG00000184898	***RBM43***	RNA binding motif protein 43	Q6ZSC3
501	ENSG00000158805	***ZNF276***	zinc finger protein 276	Q8N554
502	ENSG00000158710	***TAGLN2***	transgelin 2	P37802
503	ENSG00000172757	***CFL1***	cofilin 1	P23528
504	ENSG00000167615	***LENG8***	leukocyte receptor cluster member 8	Q96PV6
505	ENSG00000100105	***PATZ1***	POZ/BTB and AT hook containing zinc finger 1	Q9HBE1
506	ENSG00000148773	***MKI67***	marker of proliferation Ki-67	P46013
507	ENSG00000244165	***P2RY11***	purinergic receptor P2Y11	Q96G91
508	ENSG00000213903	***LTB4R***	leukotriene B4 receptor	Q15722
509	ENSG00000227507	***LTB***	lymphotoxin beta	Q06643
510	ENSG00000182809	***CRIP2***	cysteine rich protein 2	P52943
511	ENSG00000213654	***GPSM3***	G protein signaling modulator 3	Q9Y4H4
512	ENSG00000137038	***DMAC1***	distal membrane arm assembly component 1	Q96GE9
513	ENSG00000196155	***PLEKHG4***	pleckstrin homology and RhoGEF domain containing G4	Q58EX7
514	ENSG00000159445	***THEM4***	thioesterase superfamily member 4	Q5T1C6
515	ENSG00000188868	***ZNF563***	zinc finger protein 563	Q8TA94
516	ENSG00000126759	***CFP***	complement factor properdin	P27918
517	ENSG00000184903	***IMMP2L***	inner mitochondrial membrane peptidase subunit 2	Q96T52
518	ENSG00000148660	***CAMK2G***	calcium/calmodulin dependent protein kinase II gamma	Q13555
519	ENSG00000136104	***RNASEH2B***	ribonuclease H2 subunit B	Q5TBB1
520	ENSG00000134072	***CAMK1***	calcium/calmodulin dependent protein kinase I	Q14012
521	ENSG00000110013	***SIAE***	sialic acid acetylesterase	Q9HAT2

522	ENSG00000137486	***ARRB1***	arrestin beta 1	P49407
523	ENSG00000134825	***TMEM258***	transmembrane protein 258	P61165
524	ENSG00000126353	***CCR7***	C-C motif chemokine receptor 7	P32248
525	ENSG00000091592	***NLRP1***	NLR family pyrin domain containing 1	Q9C000
526	ENSG00000167889	***MGAT5B***	alpha-1,6-mannosylglycoprotein 6-beta-N-acetylglucosaminyltransferase B	Q3V5L5
527	ENSG00000160014	***CALM3***	calmodulin 3	P0DP25
528	ENSG00000081237	***PTPRC***	protein tyrosine phosphatase receptor type C	P08575
529	ENSG00000188603	***CLN3***	CLN3 lysosomal/endosomal transmembrane protein, battenin	Q13286
530	ENSG00000164120	***HPGD***	15-hydroxyprostaglandin dehydrogenase	P15428
531	ENSG00000113851	***CRBN***	cereblon	Q96SW2
532	ENSG00000168421	***RHOH***	ras homolog family member H	Q15669
533	ENSG00000170959	***DCDC1***	doublecortin domain containing 1	M0R2J8
534	ENSG00000174837	***ADGRE1***	adhesion G protein-coupled receptor E1	Q14246
535	ENSG00000148444	***COMMD3***	COMM domain containing 3	Q9UBI1
536	ENSG00000164674	***SYTL3***	synaptotagmin like 3	Q4VX76
537	ENSG00000132932	***ATP8A2***	ATPase phospholipid transporting 8A2	Q9NTI2
538	ENSG00000027075	***PRKCH***	protein kinase C eta	P24723
539	ENSG00000276234	***TADA2A***	transcriptional adaptor 2A	O75478
540	ENSG00000106785	***TRIM14***	tripartite motif containing 14	Q14142
541	ENSG00000237190	***CDKN2AIPNL***	CDKN2A interacting protein N-terminal like	Q96HQ2
542	ENSG00000198805	***PNP***	purine nucleoside phosphorylase	P00491
543	ENSG00000112763	***BTN2A1***	butyrophilin subfamily 2 member A1	Q7KYR7
544	ENSG00000178425	***NT5DC1***	5'-nucleotidase domain containing 1	Q5TFE4
545	ENSG00000196230	***TUBB***	tubulin beta class I	P07437
546	ENSG00000133256	***PDE6B***	phosphodiesterase 6B	P35913
547	ENSG00000101160	***CTSZ***	cathepsin Z	Q9UBR2
548	ENSG00000114026	***OGG1***	8-oxoguanine DNA glycosylase	O15527
549	ENSG00000163629	***PTPN13***	protein tyrosine phosphatase non-receptor type 13	Q12923
550	ENSG00000137642	***SORL1***	sortilin related receptor 1	Q92673
551	ENSG00000132589	***FLOT2***	flotillin 2	Q14254
552	ENSG00000099139	***PCSK5***	proprotein convertase subtilisin/kexin type 5	Q92824
553	ENSG00000157191	***NECAP2***	NECAP endocytosis associated 2	Q9NVZ3
554	ENSG00000196700	***ZNF512B***	zinc finger protein 512B	Q96KM6
555	ENSG00000026751	***SLAMF7***	SLAM family member 7	Q9NQ25
556	ENSG00000100784	***RPS6KA5***	ribosomal protein S6 kinase A5	O75582

557	ENSG00000278195	***SSTR3***	somatostatin receptor 3	P32745
558	ENSG00000173200	***PARP15***	poly(ADP-ribose) polymerase family member 15	Q460N3
559	ENSG00000147533	***GOLGA7***	golgin A7	Q7Z5G4
560	ENSG00000179010	***MRFAP1***	Morf4 family associated protein 1	Q9Y605
561	ENSG00000163466	***ARPC2***	actin related protein 2/3 complex subunit 2	O15144
562	ENSG00000197077	***KIAA1671***	KIAA1671	Q9BY89
563	ENSG00000168404	***MLKL***	mixed lineage kinase domain like pseudokinase	Q8NB16
564	ENSG00000074966	***TXK***	TXK tyrosine kinase	P42681
565	ENSG00000111229	***ARPC3***	actin related protein 2/3 complex subunit 3	O15145
566	ENSG00000151883	***PARP8***	poly(ADP-ribose) polymerase family member 8	Q8N3A8
567	ENSG00000108298	***RPL19***	ribosomal protein L19	P84098
568	ENSG00000155657	***TTN***	titin	Q8WZ42
569	ENSG00000152229	***PSTPIP2***	proline-serine-threonine phosphatase interacting protein 2	Q9H939
570	ENSG00000120913	***PDLIM2***	PDZ and LIM domain 2	Q96JY6
571	ENSG00000182871	***COL18A1***	collagen type XVIII alpha 1 chain	P39060
572	ENSG00000044574	***HSPA5***	heat shock protein family A (Hsp70) member 5	P11021
573	ENSG00000101082	***SLA2***	Src like adaptor 2	Q9H6Q3
574	ENSG00000173559	***NABP1***	nucleic acid binding protein 1	Q96AH0
575	ENSG00000183690	***EFHC2***	EF-hand domain containing 2	Q5JST6
576	ENSG00000111331	***OAS3***	2'-5'-oligoadenylate synthetase 3	Q9Y6K5
577	ENSG00000138180	***CEP55***	centrosomal protein 55	Q53EZ4
578	ENSG00000170873	***MTSS1***	MTSS I-BAR domain containing 1	O43312
579	ENSG00000102678	***FGF9***	fibroblast growth factor 9	P31371
580	ENSG00000165475	***CRYL1***	crystallin lambda 1	Q9Y2S2
581	ENSG00000221983	***UBA52***	ubiquitin A-52 residue ribosomal protein fusion product 1	P62987
582	ENSG00000115042	***FAHD2A***	fumarylacetoacetate hydrolase domain containing 2A	Q96GK7
583	ENSG00000141576	***RNF157***	ring finger protein 157	Q96PX1
584	ENSG00000232388	***SMIM26***	small integral membrane protein 26	A0A096LP01
585	ENSG00000182195	***LDOC1***	LDOC1 regulator of NFKB signaling	O95751
586	ENSG00000059378	***PARP12***	poly(ADP-ribose) polymerase family member 12	Q9H0J9
587	ENSG00000197093	***GAL3ST4***	galactose-3-O-sulfotransferase 4	Q96RP7
588	ENSG00000000971	***CFH***	complement factor H	P08603
589	ENSG00000174151	***CYB561D1***	cytochrome b561 family member D1	Q8N8Q1
590	ENSG00000176209	***SMIM19***	small integral membrane protein 19	Q96E16
591	ENSG00000159640	***ACE***	angiotensin I converting enzyme	P12821

592	ENSG00000100823	***APEX1***	apurinic/apyrimidinic endodeoxyribonuclease 1	P27695
593	ENSG00000107485	***GATA3***	GATA binding protein 3	P23771
594	ENSG00000080573	***COL5A3***	collagen type V alpha 3 chain	P25940
595	ENSG00000111860	***CEP85L***	centrosomal protein 85 like	Q5SZL2
596	ENSG00000160185	***UBASH3A***	ubiquitin associated and SH3 domain containing A	P57075
597	ENSG00000101347	***SAMHD1***	SAM and HD domain containing deoxynucleoside triphosphate triphosphohydrolase 1	Q9Y3Z3
598	ENSG00000047644	***WWC3***	WWC family member 3	Q9ULE0
599	ENSG00000117360	***PRPF3***	pre-mRNA processing factor 3	O43395
600	ENSG00000134363	***FST***	follistatin	P19883
601	ENSG00000177383	***MAGEF1***	MAGE family member F1	Q9HAY2
602	ENSG00000162639	***HENMT1***	HEN methyltransferase 1	Q5T8I9
603	ENSG00000029363	***BCLAF1***	BCL2 associated transcription factor 1	Q9NYF8
604	ENSG00000271503	***CCL5***	C-C motif chemokine ligand 5	P13501
605	ENSG00000103479	***RBL2***	RB transcriptional corepressor like 2	Q08999
606	ENSG00000158856	***DMTN***	dematin actin binding protein	Q08495
607	ENSG00000070718	***AP3M2***	adaptor related protein complex 3 subunit mu 2	P53677
608	ENSG00000049239	***H6PD***	hexose-6-phosphate dehydrogenase/glucose 1-dehydrogenase	O95479
609	ENSG00000145703	***IQGAP2***	IQ motif containing GTPase activating protein 2	Q13576
610	ENSG00000142173	***COL6A2***	collagen type VI alpha 2 chain	P12110
611	ENSG00000114812	***VIPR1***	vasoactive intestinal peptide receptor 1	P32241
612	ENSG00000008513	***ST3GAL1***	ST3 beta-galactoside alpha-2,3-sialyltransferase 1	Q11201
613	ENSG00000142534	***RPS11***	ribosomal protein S11	P62280
614	ENSG00000114023	***FAM162A***	family with sequence similarity 162 member A	Q96A26
615	ENSG00000275395	***FCGBP***	Fc gamma binding protein	Q9Y6R7
616	ENSG00000101842	***VSIG1***	V-set and immunoglobulin domain containing 1	Q86XK7
617	ENSG00000140350	***ANP32A***	acidic nuclear phosphoprotein 32 family member A	P39687
618	ENSG00000124570	***SERPINB6***	serpin family B member 6	P35237
619	ENSG00000173208	***ABCD2***	ATP binding cassette subfamily D member 2	Q9UBJ2
620	ENSG00000115425	***PECR***	peroxisomal trans-2-enoyl-CoA reductase	Q9BY49
621	ENSG00000169429	***CXCL8***	C-X-C motif chemokine ligand 8	P10145
622	ENSG00000184182	***UBE2F***	ubiquitin conjugating enzyme E2 F (putative)	Q969M7
623	ENSG00000085265	***FCN1***	ficolin 1	O00602
624	ENSG00000151552	***QDPR***	quinoid dihydropteridine reductase	P09417
625	ENSG00000197471	***SPN***	sialophorin	P16150
626	ENSG00000100201	***DDX17***	DEAD-box helicase 17	Q92841

627	ENSG00000139631	***CSAD***	cysteine sulfinic acid decarboxylase	Q9Y600
628	ENSG00000112242	***E2F3***	E2F transcription factor 3	O00716
629	ENSG00000136444	***RSAD1***	radical S-adenosyl methionine domain containing 1	Q9HA92
630	ENSG00000163154	***TNFAIP8L2***	TNF alpha induced protein 8 like 2	Q6P589
631	ENSG00000164258	***NDUFS4***	NADH:ubiquinone oxidoreductase subunit S4	O43181
632	ENSG00000188895	***MSL1***	MSL complex subunit 1	Q68DK7
633	ENSG00000239779	***WBP1***	WW domain binding protein 1	Q96G27
634	ENSG00000075188	***NUP37***	nucleoporin 37	Q8NFH4
635	ENSG00000035141	***FAM136A***	family with sequence similarity 136 member A	Q96C01
636	ENSG00000148468	***FAM171A1***	family with sequence similarity 171 member A1	Q5VUB5
637	ENSG00000169084	***DHRSX***	dehydrogenase/reductase X-linked	Q8N5I4
638	ENSG00000121858	***TNFSF10***	TNF superfamily member 10	P50591
639	ENSG00000135414	***GDF11***	growth differentiation factor 11	O95390
640	ENSG00000078401	***EDN1***	endothelin 1	P05305
641	ENSG00000197747	***S100A10***	S100 calcium binding protein A10	P60903
642	ENSG00000162545	***CAMK2N1***	calcium/calmodulin dependent protein kinase II inhibitor 1	Q7Z7J9
643	ENSG00000122122	***SASH3***	SAM and SH3 domain containing 3	O75995
644	ENSG00000144476	***ACKR3***	atypical chemokine receptor 3	P25106
645	ENSG00000212907	***MT-ND4L***	mitochondrially encoded NADH:ubiquinone oxidoreductase core subunit 4L	P03901
646	ENSG00000243147	***MRPL33***	mitochondrial ribosomal protein L33	O75394
647	ENSG00000111653	***ING4***	inhibitor of growth family member 4	Q9UNL4
648	ENSG00000162711	***NLRP3***	NLR family pyrin domain containing 3	Q96P20
649	ENSG00000145833	***DDX46***	DEAD-box helicase 46	Q7L014
650	ENSG00000168653	***NDUFS5***	NADH:ubiquinone oxidoreductase subunit S5	O43920
651	ENSG00000188243	***COMMD6***	COMM domain containing 6	Q7Z4G1
652	ENSG00000163606	***CD200R1***	CD200 receptor 1	Q8TD46
653	ENSG00000127951	***FGL2***	fibrinogen like 2	Q14314
654	ENSG00000013563	***DNASE1L1***	deoxyribonuclease 1 like 1	P49184
655	ENSG00000157978	***LDLRAP1***	low density lipoprotein receptor adaptor protein 1	Q5SW96
656	ENSG00000147604	***RPL7***	ribosomal protein L7	P18124
657	ENSG00000146826	***TRAPPC14***	trafficking protein particle complex subunit 14	Q8WVR3
658	ENSG00000103811	***CTSH***	cathepsin H	P09668
659	ENSG00000019102	***VSIG2***	V-set and immunoglobulin domain containing 2	Q96IQ7
660	ENSG00000137507	***LRRC32***	leucine rich repeat containing 32	Q14392
661	ENSG00000132635	***PCED1A***	PC-esterase domain containing 1A	Q9H1Q7

662	ENSG00000007255	***TRAPPC6A***	trafficking protein particle complex subunit 6A	O75865
663	ENSG00000122042	***UBL3***	ubiquitin like 3	O95164
664	ENSG00000182700	***IGIP***	IgA inducing protein	A6NJ69
665	ENSG00000152795	***HNRNPDL***	heterogeneous nuclear ribonucleoprotein D like	O14979
666	ENSG00000137078	***SIT1***	signaling threshold regulating transmembrane adaptor 1	Q9Y3P8
667	ENSG00000144218	***AFF3***	ALF transcription elongation factor 3	P51826
668	ENSG00000107281	***NPDC1***	neural proliferation, differentiation and control 1	Q9NQX5
669	ENSG00000136111	***TBC1D4***	TBC1 domain family member 4	O60343
670	ENSG00000169714	***CNBP***	CCHC-type zinc finger nucleic acid binding protein	P62633
671	ENSG00000163568	***AIM2***	absent in melanoma 2	O14862
672	ENSG00000108848	***LUC7L3***	LUC7 like 3 pre-mRNA splicing factor	O95232
673	ENSG00000113558	***SKP1***	S-phase kinase associated protein 1	P63208
674	ENSG00000090263	***MRPS33***	mitochondrial ribosomal protein S33	Q9Y291
675	ENSG00000120727	***PAIP2***	poly(A) binding protein interacting protein 2	Q9BPZ3
676	ENSG00000088986	***DYNLL1***	dynein light chain LC8-type 1	P63167
677	ENSG00000231500	***RPS18***	ribosomal protein S18	P62269
678	ENSG00000181804	***SLC9A9***	solute carrier family 9 member A9	Q8IVB4
679	ENSG00000185189	***NRBP2***	nuclear receptor binding protein 2	Q9NSY0
680	ENSG00000105639	***JAK3***	Janus kinase 3	P52333
681	ENSG00000138964	***PARVG***	parvin gamma	Q9HBI0
682	ENSG00000205336	***ADGRG1***	adhesion G protein-coupled receptor G1	Q9Y653
683	ENSG00000117676	***RPS6KA1***	ribosomal protein S6 kinase A1	Q15418
684	ENSG00000107020	***PLGRKT***	plasminogen receptor with a C-terminal lysine	Q9HBL7
685	ENSG00000186265	***BTLA***	B and T lymphocyte associated	Q7Z6A9
686	ENSG00000152234	***ATP5F1A***	ATP synthase F1 subunit alpha	P25705
687	ENSG00000106605	***BLVRA***	biliverdin reductase A	P53004
688	ENSG00000151062	***CACNA2D4***	calcium voltage-gated channel auxiliary subunit alpha2delta 4	Q7Z3S7
689	ENSG00000187713	***TMEM203***	transmembrane protein 203	Q969S6
690	ENSG00000134905	***CARS2***	cysteinyl-tRNA synthetase 2, mitochondrial	Q9HA77
691	ENSG00000153898	***MCOLN2***	mucolipin TRP cation channel 2	Q8IZK6
692	ENSG00000176390	***CRLF3***	cytokine receptor like factor 3	Q8IUI8
693	ENSG00000127152	***BCL11B***	BCL11 transcription factor B	Q9C0K0
694	ENSG00000149476	***TKFC***	triokinase and FMN cyclase	Q3LXA3
695	ENSG00000164687	***FABP5***	fatty acid binding protein 5	Q01469
696	ENSG00000154059	***IMPACT***	impact RWD domain protein	Q9P2X3

697	ENSG00000204271	***SPIN3***	spindlin family member 3	Q5JUX0
698	ENSG00000145743	***FBXL17***	F-box and leucine rich repeat protein 17	Q9UF56
699	ENSG00000078081	***LAMP3***	lysosomal associated membrane protein 3	Q9UQV4
700	ENSG00000115525	***ST3GAL5***	ST3 beta-galactoside alpha-2,3-sialyltransferase 5	Q9UNP4
701	ENSG00000177971	***IMP3***	IMP U3 small nucleolar ribonucleoprotein 3	Q9NV31
702	ENSG00000147155	***EBP***	EBP cholestenol delta-isomerase	Q15125
703	ENSG00000263465	***SRSF8***	serine and arginine rich splicing factor 8	Q9BRL6
704	ENSG00000084072	***PPIE***	peptidylprolyl isomerase E	Q9UNP9
705	ENSG00000187837	***H1-2***	H1.2 linker histone, cluster member	P16403
706	ENSG00000089280	***FUS***	FUS RNA binding protein	P35637
707	ENSG00000272047	***GTF2H5***	general transcription factor IIH subunit 5	Q6ZYL4
708	ENSG00000163479	***SSR2***	signal sequence receptor subunit 2	P43308
709	ENSG00000009307	***CSDE1***	cold shock domain containing E1	O75534
710	ENSG00000137193	***PIM1***	Pim-1 proto-oncogene, serine/threonine kinase	P11309
711	ENSG00000159733	***ZFYVE28***	zinc finger FYVE-type containing 28	Q9HCC9
712	ENSG00000090924	***PLEKHG2***	pleckstrin homology and RhoGEF domain containing G2	Q9H7P9
713	ENSG00000180644	***PRF1***	perforin 1	P14222
714	ENSG00000205784	***ARRDC5***	arrestin domain containing 5	A6NEK1
715	ENSG00000164587	***RPS14***	ribosomal protein S14	P62263
716	ENSG00000123329	***ARHGAP9***	Rho GTPase activating protein 9	Q9BRR9
717	ENSG00000136514	***RTP4***	receptor transporter protein 4	Q96DX8
718	ENSG00000066923	***STAG3***	stromal antigen 3	Q9UJ98
719	ENSG00000131171	***SH3BGRL***	SH3 domain binding glutamate rich protein like	O75368
720	ENSG00000139428	***MMAB***	metabolism of cobalamin associated B	Q96EY8
721	ENSG00000163508	***EOMES***	eomesodermin	O95936
722	ENSG00000135899	***SP110***	SP110 nuclear body protein	Q9HB58
723	ENSG00000143149	***ALDH9A1***	aldehyde dehydrogenase 9 family member A1	P49189

Table 1a continued

Order	chr	start	end	baseMean	log2FoldChange	lfcSE	stat	pvalue	padj
1	10	17228241	17237593	10461.483	-0.686	0.075	-9.177	4.44E-20	1.44E-16
2	X	12975110	12977227	48176.611	-0.577	0.072	-8.062	7.52E-16	1.08E-12
3	6	32439878	32445046	633.049	-1.137	0.147	-7.709	1.27E-14	1.32E-11
4	10	78033760	78056813	18476.569	-0.730	0.095	-7.703	1.33E-14	1.32E-11
5	6	33064569	33080775	417.419	-0.963	0.130	-7.393	1.43E-13	1.16E-10
6	6	26402237	26415208	2825.734	-0.939	0.131	-7.170	7.52E-13	5.12E-10
7	9	114893343	114930595	1146.126	-0.816	0.114	-7.145	9.00E-13	5.83E-10
8	2	84905656	84906671	16350.584	-0.622	0.092	-6.761	1.37E-11	6.08E-09
9	11	119015712	119018691	30745.712	-0.533	0.079	-6.757	1.41E-11	6.08E-09
10	6	33075990	33089696	318.848	-1.159	0.174	-6.660	2.74E-11	1.11E-08
11	6	26365159	26378320	3097.909	-0.814	0.125	-6.504	7.80E-11	2.65E-08
12	6	32844136	32859851	851.254	-0.822	0.127	-6.454	1.09E-10	3.44E-08
13	6	26440472	26453415	2129.494	-0.732	0.114	-6.424	1.32E-10	4.08E-08
14	22	37675636	37679802	476.452	-0.723	0.113	-6.388	1.68E-10	4.93E-08
15	6	30489509	30494194	21754.754	-0.713	0.112	-6.381	1.76E-10	4.96E-08
16	12	104303739	104305205	109.010	-1.274	0.200	-6.381	1.76E-10	4.96E-08
17	12	93569814	93583487	272.816	-0.932	0.147	-6.342	2.27E-10	6.12E-08
18	1	157674321	157700769	201.018	-1.190	0.189	-6.296	3.06E-10	7.76E-08
19	5	150401637	150412969	2645.553	-0.622	0.099	-6.257	3.92E-10	9.74E-08
20	16	3065297	3082192	4786.915	-0.671	0.107	-6.250	4.11E-10	1.00E-07
21	15	44728988	44767829	867.798	-0.572	0.092	-6.216	5.10E-10	1.22E-07
22	14	55124110	55145423	568.618	-0.653	0.107	-6.111	9.88E-10	2.28E-07
23	5	55024256	55034570	365.982	-1.139	0.187	-6.107	1.02E-09	2.31E-07
24	10	71396920	71815947	268.652	-1.072	0.177	-6.061	1.35E-09	3.02E-07
25	7	66628881	66649067	1002.502	-0.512	0.085	-6.056	1.40E-09	3.07E-07
26	3	50606489	50611774	599.263	-0.822	0.137	-5.984	2.18E-09	4.55E-07
27	6	32578769	32589848	300.924	-1.002	0.168	-5.966	2.43E-09	5.00E-07
28	19	39328353	39342372	3215.210	-0.548	0.092	-5.959	2.54E-09	5.14E-07
29	2	55231903	55235853	45091.864	-0.408	0.069	-5.901	3.60E-09	6.96E-07
30	18	3247481	3256236	7915.620	-0.504	0.086	-5.894	3.78E-09	7.19E-07
31	15	44711487	44718851	93971.046	-0.554	0.095	-5.856	4.74E-09	8.63E-07

32	7	77310751	77416349	351.806	-0.786	0.135	-5.824	5.76E-09	1.03E-06
33	21	42653621	42775509	192.409	-1.055	0.181	-5.821	5.84E-09	1.04E-06
34	12	52607570	52618559	361.159	-0.950	0.165	-5.770	7.94E-09	1.35E-06
35	16	692498	705808	883.432	-0.610	0.106	-5.741	9.39E-09	1.58E-06
36	17	74670578	74747335	526.433	-0.832	0.146	-5.703	1.18E-08	1.90E-06
37	3	197950190	197956610	17043.006	-0.397	0.070	-5.678	1.36E-08	2.13E-06
38	12	48904110	48931840	436.340	-0.732	0.129	-5.665	1.47E-08	2.24E-06
39	12	9752486	9760901	724.928	-0.665	0.118	-5.660	1.51E-08	2.26E-06
40	11	2295628	2303049	895.238	-0.844	0.149	-5.659	1.52E-08	2.26E-06
41	12	69348381	69354234	579.953	-0.792	0.140	-5.648	1.62E-08	2.39E-06
42	21	46635595	46665124	3489.918	-0.390	0.069	-5.634	1.77E-08	2.57E-06
43	1	89051882	89065360	1283.946	-0.652	0.116	-5.597	2.18E-08	3.09E-06
44	6	132814569	132817564	39488.341	-0.335	0.060	-5.578	2.44E-08	3.39E-06
45	18	69831158	69961803	966.065	-0.631	0.114	-5.541	3.00E-08	4.09E-06
46	11	310041	315272	615.967	-0.826	0.149	-5.532	3.17E-08	4.27E-06
47	3	46353864	46360940	203.930	-1.251	0.227	-5.511	3.57E-08	4.77E-06
48	2	161992245	162074394	1328.324	-0.517	0.094	-5.507	3.65E-08	4.82E-06
49	6	32845209	32853816	1976.361	-0.573	0.105	-5.472	4.45E-08	5.72E-06
50	MT	10059	10404	18002.696	-0.618	0.113	-5.471	4.46E-08	5.72E-06
51	22	38818452	38844028	312.296	-0.713	0.131	-5.446	5.15E-08	6.41E-06
52	16	18781295	18790383	6172.548	-0.576	0.106	-5.428	5.71E-08	6.97E-06
53	2	12716910	12742734	6208.861	-0.696	0.128	-5.422	5.90E-08	7.12E-06
54	5	55102646	55110252	420.463	-0.789	0.146	-5.409	6.34E-08	7.52E-06
55	3	111292719	111665750	5783.795	-0.419	0.078	-5.403	6.55E-08	7.70E-06
56	6	130144315	130365425	270.300	-0.704	0.130	-5.401	6.64E-08	7.74E-06
57	10	6010689	6062370	485.901	-0.742	0.138	-5.390	7.05E-08	8.15E-06
58	1	167052836	167166479	759.421	-0.897	0.167	-5.382	7.38E-08	8.45E-06
59	3	114276913	114310288	1301.551	-0.695	0.130	-5.366	8.03E-08	9.06E-06
60	7	150514872	150521073	7153.513	-0.342	0.064	-5.366	8.05E-08	9.06E-06
61	17	39953263	39977768	54.052	-1.701	0.317	-5.364	8.16E-08	9.06E-06
62	10	32887273	33005792	6720.878	-0.429	0.080	-5.363	8.20E-08	9.06E-06
63	1	234391313	234479179	956.315	-0.615	0.116	-5.296	1.18E-07	1.27E-05
64	14	33924227	34462774	139.881	-0.911	0.172	-5.292	1.21E-07	1.28E-05
65	1	228735479	228746664	106.727	-1.312	0.248	-5.280	1.29E-07	1.36E-05
66	2	181457202	181538940	7088.737	-0.623	0.118	-5.270	1.36E-07	1.40E-05

67	17	40443450	40457725	75.710	-1.172	0.223	-5.264	1.41E-07	1.42E-05
68	11	133896438	133956968	974.027	-0.575	0.109	-5.261	1.44E-07	1.43E-05
69	14	91232532	91253925	225.048	-0.779	0.149	-5.233	1.67E-07	1.62E-05
70	14	24136163	24138967	2929.877	-0.427	0.083	-5.162	2.44E-07	2.34E-05
71	11	73646178	73662819	967.818	-0.480	0.093	-5.158	2.49E-07	2.37E-05
72	8	98024851	98046469	34612.897	-0.407	0.079	-5.154	2.55E-07	2.41E-05
73	1	223701593	223776018	3685.471	-0.391	0.076	-5.149	2.62E-07	2.44E-05
74	4	108620569	108630412	22702.990	-0.379	0.074	-5.142	2.72E-07	2.52E-05
75	11	44065925	44084237	393.600	-0.908	0.177	-5.140	2.75E-07	2.52E-05
76	2	231706895	231713551	17882.971	-0.416	0.081	-5.130	2.90E-07	2.64E-05
77	6	107702154	107824317	1377.014	-0.576	0.112	-5.127	2.95E-07	2.67E-05
78	7	22812628	22822849	4772.891	-0.363	0.071	-5.123	3.01E-07	2.70E-05
79	8	24950955	24956721	282.028	-0.722	0.141	-5.122	3.03E-07	2.70E-05
80	9	19375715	19380236	63822.878	-0.333	0.065	-5.112	3.20E-07	2.83E-05
81	3	67360460	67654612	869.446	-0.528	0.104	-5.096	3.46E-07	3.05E-05
82	17	35756249	35795707	88.104	-1.432	0.281	-5.091	3.56E-07	3.09E-05
83	16	79585843	79600737	1003.771	-0.526	0.104	-5.070	3.98E-07	3.41E-05
84	17	38847860	38853764	20647.373	-0.337	0.067	-5.061	4.17E-07	3.55E-05
85	2	27070472	27071654	1804.468	-0.454	0.090	-5.038	4.71E-07	3.96E-05
86	17	48133442	48430275	1818.253	-0.407	0.081	-5.033	4.83E-07	4.01E-05
87	11	65375192	65383701	363.334	-0.722	0.144	-5.013	5.36E-07	4.39E-05
88	MT	14747	15887	43909.079	-0.501	0.100	-5.006	5.57E-07	4.51E-05
89	1	152032506	152047907	1942.915	-0.391	0.078	-4.995	5.89E-07	4.67E-05
90	13	113584721	113641473	679.002	-0.469	0.094	-4.971	6.67E-07	5.27E-05
91	8	19404161	19758029	1647.529	-0.491	0.099	-4.963	6.96E-07	5.45E-05
92	17	75038863	75046985	1445.354	-0.415	0.084	-4.942	7.73E-07	6.03E-05
93	11	76349898	76381132	2088.080	-0.487	0.099	-4.929	8.26E-07	6.36E-05
94	16	31259967	31332892	82.913	-1.092	0.223	-4.895	9.86E-07	7.46E-05
95	10	103877569	103918184	594.069	-0.493	0.101	-4.893	9.92E-07	7.46E-05
96	14	49570984	49599164	16778.488	-0.505	0.103	-4.891	1.00E-06	7.50E-05
97	1	153534599	153536244	3425.524	-0.424	0.087	-4.882	1.05E-06	7.75E-05
98	22	23688136	23699176	77.833	-1.402	0.287	-4.881	1.06E-06	7.77E-05
99	2	101002229	101024032	25631.613	-0.360	0.074	-4.865	1.14E-06	8.31E-05
100	12	52674736	52680407	218.548	-1.130	0.233	-4.858	1.19E-06	8.58E-05
101	6	31615217	31617021	104.778	-1.121	0.231	-4.855	1.20E-06	8.63E-05

102	6	32517353	32530287	72.566	-1.647	0.340	-4.849	1.24E-06	8.86E-05
103	1	26317958	26320523	9598.486	-0.512	0.106	-4.837	1.32E-06	9.30E-05
104	2	3575260	3580920	19328.305	-0.410	0.085	-4.832	1.35E-06	9.45E-05
105	6	32628179	32647062	140.888	-1.282	0.265	-4.830	1.36E-06	9.49E-05
106	20	62387103	62388520	12362.876	-0.371	0.077	-4.829	1.37E-06	9.50E-05
107	22	17178790	17258235	2169.420	-0.530	0.110	-4.823	1.41E-06	9.73E-05
108	8	56067254	56074510	28915.544	-0.329	0.068	-4.819	1.44E-06	9.88E-05
109	6	44809317	45377953	261.604	-0.682	0.142	-4.811	1.50E-06	1.02E-04
110	1	111185969	111204535	2425.221	-0.394	0.082	-4.803	1.56E-06	1.05E-04
111	1	203305491	203309602	4043.722	-0.317	0.066	-4.802	1.57E-06	1.05E-04
112	16	10866222	10943021	135.628	-0.917	0.191	-4.800	1.59E-06	1.05E-04
113	14	52314305	52328598	479.400	-0.783	0.163	-4.799	1.59E-06	1.05E-04
114	1	89106132	89150456	5031.501	-0.427	0.089	-4.785	1.71E-06	1.11E-04
115	17	7549058	7557890	342.927	-0.630	0.132	-4.785	1.71E-06	1.11E-04
116	5	82273320	82278396	31028.761	-0.317	0.066	-4.785	1.71E-06	1.11E-04
117	11	63536808	63546462	503.483	-0.510	0.107	-4.779	1.76E-06	1.14E-04
118	14	69726900	69772005	13238.399	-0.318	0.067	-4.740	2.14E-06	1.36E-04
119	7	143244093	143270854	8446.157	-0.498	0.105	-4.734	2.20E-06	1.39E-04
120	3	52287089	52295257	364.418	-0.693	0.147	-4.729	2.26E-06	1.42E-04
121	1	44775251	44778779	36651.200	-0.341	0.072	-4.715	2.41E-06	1.51E-04
122	11	105025443	105035250	862.065	-0.482	0.102	-4.712	2.45E-06	1.52E-04
123	6	32934629	32941028	95.532	-0.929	0.197	-4.711	2.47E-06	1.53E-04
124	1	160678746	160711831	4926.237	-0.420	0.089	-4.703	2.57E-06	1.58E-04
125	19	2476122	2478259	413.579	-0.604	0.128	-4.703	2.57E-06	1.58E-04
126	1	214281102	214337131	837.840	-0.425	0.090	-4.696	2.65E-06	1.62E-04
127	19	54351384	54370558	885.489	-0.494	0.105	-4.694	2.68E-06	1.63E-04
128	2	6917412	7068286	1061.470	-0.466	0.100	-4.680	2.87E-06	1.73E-04
129	3	16265160	16350299	1087.544	-0.498	0.107	-4.666	3.07E-06	1.84E-04
130	17	39063313	39154394	355.568	-0.969	0.208	-4.661	3.14E-06	1.86E-04
131	10	97849843	97871580	625.927	-0.673	0.144	-4.660	3.17E-06	1.87E-04
132	10	101781325	101783413	106.246	-0.933	0.201	-4.652	3.29E-06	1.94E-04
133	8	27490781	27545564	846.424	-0.624	0.135	-4.641	3.47E-06	2.03E-04
134	19	35738801	35742453	142.452	-0.849	0.183	-4.638	3.52E-06	2.05E-04
135	13	41457550	41470871	1803.801	-0.469	0.101	-4.637	3.54E-06	2.05E-04
136	12	6346843	6377730	35.985	-1.431	0.309	-4.626	3.72E-06	2.12E-04

137	13	108251240	108308484	314.511	-0.744	0.161	-4.614	3.95E-06	2.23E-04
138	20	59025475	59032345	4487.617	-0.362	0.079	-4.600	4.22E-06	2.36E-04
139	7	93130056	93148385	1119.552	-0.545	0.119	-4.596	4.31E-06	2.40E-04
140	1	25242249	25338213	3407.712	-0.463	0.101	-4.581	4.63E-06	2.56E-04
141	12	44508275	44921848	4826.426	-0.415	0.091	-4.569	4.91E-06	2.70E-04
142	12	51281038	51324668	6672.704	-0.369	0.081	-4.567	4.94E-06	2.71E-04
143	1	151050971	151070325	11388.598	-0.387	0.085	-4.555	5.24E-06	2.82E-04
144	6	29555629	29633976	885.931	-0.457	0.100	-4.547	5.45E-06	2.91E-04
145	7	150685697	150693641	2531.928	-0.363	0.080	-4.536	5.72E-06	3.05E-04
146	1	161215234	161220699	71.183	-1.270	0.280	-4.533	5.82E-06	3.07E-04
147	7	10931943	10940153	1253.650	-0.344	0.076	-4.533	5.83E-06	3.07E-04
148	11	17074388	17077715	19413.667	-0.305	0.067	-4.528	5.95E-06	3.12E-04
149	6	31588895	31593006	136.912	-0.782	0.173	-4.519	6.23E-06	3.25E-04
150	13	27251362	27256691	40159.528	-0.355	0.079	-4.511	6.46E-06	3.35E-04
151	19	45687460	45692569	2065.834	-0.386	0.086	-4.503	6.71E-06	3.48E-04
152	1	153543613	153550136	4662.547	-0.407	0.091	-4.501	6.77E-06	3.49E-04
153	1	150293861	150308979	1201.667	-0.394	0.088	-4.498	6.87E-06	3.53E-04
154	17	35435096	35448837	451.756	-0.619	0.138	-4.492	7.04E-06	3.60E-04
155	7	150567369	150573953	6709.384	-0.349	0.078	-4.486	7.25E-06	3.69E-04
156	13	48389567	48444704	2393.767	-0.578	0.129	-4.475	7.63E-06	3.86E-04
157	15	48331011	48343373	1077.499	-0.411	0.092	-4.472	7.75E-06	3.90E-04
158	13	51813347	51845177	223.779	-0.699	0.156	-4.470	7.84E-06	3.93E-04
159	17	43640389	43661922	98.494	-1.031	0.231	-4.456	8.36E-06	4.15E-04
160	2	86784610	86808396	123.896	-0.941	0.211	-4.455	8.37E-06	4.15E-04
161	5	40825262	40835222	24541.646	-0.377	0.085	-4.452	8.51E-06	4.20E-04
162	3	48440257	48444208	2902.987	-0.385	0.087	-4.448	8.67E-06	4.25E-04
163	12	112978395	113011723	2114.550	-0.339	0.076	-4.444	8.81E-06	4.30E-04
164	MT	8366	8572	3268.004	-0.548	0.124	-4.436	9.17E-06	4.45E-04
165	1	150560895	150579738	6485.160	-0.369	0.083	-4.432	9.32E-06	4.50E-04
166	1	203178931	203186704	27.809	-2.368	0.538	-4.402	1.07E-05	5.10E-04
167	3	46370946	46376206	111.158	-1.138	0.259	-4.395	1.11E-05	5.25E-04
168	17	28719985	28724359	27808.558	-0.370	0.084	-4.386	1.16E-05	5.46E-04
169	1	23691742	23696835	35301.039	-0.262	0.060	-4.378	1.20E-05	5.60E-04
170	X	136648158	136660390	1120.059	-0.502	0.115	-4.374	1.22E-05	5.65E-04
171	11	60455846	60470752	290.496	-0.689	0.158	-4.362	1.29E-05	5.96E-04

172	4	155921580	155953912	387.626	-0.583	0.134	-4.360	1.30E-05	5.96E-04
173	9	35681992	35690056	806.672	-0.635	0.146	-4.356	1.33E-05	6.06E-04
174	7	43608456	43729717	785.057	-0.365	0.084	-4.354	1.33E-05	6.08E-04
175	5	43039233	43043170	700.462	-0.600	0.138	-4.348	1.37E-05	6.19E-04
176	1	153990762	153992155	61525.606	-0.403	0.093	-4.346	1.39E-05	6.24E-04
177	6	109444062	109465968	1576.918	-0.571	0.131	-4.345	1.40E-05	6.25E-04
178	8	96222947	96235546	4467.509	-0.388	0.089	-4.341	1.42E-05	6.32E-04
179	12	121648742	121672631	261.889	-0.745	0.172	-4.336	1.45E-05	6.42E-04
180	6	11712054	11807046	365.522	-0.717	0.165	-4.336	1.45E-05	6.42E-04
181	5	39105252	39274528	8478.502	-0.409	0.094	-4.335	1.46E-05	6.42E-04
182	3	12834485	12841582	31019.159	-0.302	0.070	-4.327	1.51E-05	6.61E-04
183	19	544034	549924	275.507	-0.694	0.161	-4.323	1.54E-05	6.69E-04
184	1	97077743	97995000	894.432	-0.471	0.109	-4.313	1.61E-05	7.00E-04
185	7	100092728	100101940	2581.307	-0.391	0.091	-4.302	1.69E-05	7.21E-04
186	7	102433106	102456825	693.430	-0.415	0.097	-4.302	1.69E-05	7.21E-04
187	15	22983192	23039572	412.746	-0.540	0.126	-4.302	1.69E-05	7.21E-04
188	2	95025677	95053992	1519.507	-0.394	0.092	-4.300	1.71E-05	7.22E-04
189	22	36140330	36166177	1921.885	-0.320	0.074	-4.300	1.71E-05	7.22E-04
190	12	47079603	47236662	947.091	-0.401	0.093	-4.297	1.73E-05	7.29E-04
191	17	42998273	43002959	22437.799	-0.364	0.085	-4.297	1.73E-05	7.29E-04
192	17	35243071	35273655	7444.841	-0.481	0.112	-4.295	1.75E-05	7.31E-04
193	4	151099624	151104642	68041.007	-0.347	0.081	-4.289	1.79E-05	7.49E-04
194	6	132743870	132763459	465.253	-0.555	0.129	-4.288	1.80E-05	7.49E-04
195	1	116754430	116769229	4559.800	-0.380	0.089	-4.286	1.82E-05	7.53E-04
196	17	14301081	14349404	493.881	-0.485	0.114	-4.268	1.98E-05	8.17E-04
197	22	37805229	37807432	698.999	-0.557	0.131	-4.266	1.99E-05	8.20E-04
198	1	52633168	52669683	264.826	-0.655	0.154	-4.251	2.13E-05	8.75E-04
199	9	120855651	120894896	2861.899	-0.394	0.093	-4.244	2.20E-05	8.99E-04
200	1	209756032	209782320	10544.230	-0.385	0.091	-4.237	2.27E-05	9.22E-04
201	1	109668022	109709551	369.060	-0.653	0.155	-4.220	2.45E-05	9.89E-04
202	6	31730581	31739763	1676.240	-0.387	0.092	-4.214	2.51E-05	1.01E-03
203	6	111660332	111873452	8641.200	-0.336	0.080	-4.202	2.65E-05	1.06E-03
204	1	223109404	223143248	64.706	-1.122	0.267	-4.199	2.68E-05	1.07E-03
205	17	17042457	17217679	5647.844	-0.316	0.075	-4.191	2.78E-05	1.10E-03
206	1	23380909	23424748	388.357	-0.522	0.125	-4.189	2.80E-05	1.10E-03

207	1	77282019	77559966	1434.786	-1.024	0.245	-4.186	2.84E-05	1.11E-03
208	11	105041326	105101431	332.896	-0.471	0.113	-4.183	2.88E-05	1.12E-03
209	16	28494643	28498964	567.509	-0.561	0.134	-4.182	2.89E-05	1.13E-03
210	X	71107404	71112108	2340.276	-0.364	0.087	-4.179	2.93E-05	1.13E-03
211	1	145992435	145996579	17907.424	-0.278	0.066	-4.179	2.93E-05	1.13E-03
212	1	150730079	150765957	1575.682	-0.343	0.082	-4.171	3.03E-05	1.17E-03
213	12	110627841	110704950	234.315	-0.599	0.144	-4.169	3.07E-05	1.18E-03
214	19	47332175	47347329	47.098	-1.090	0.262	-4.160	3.18E-05	1.22E-03
215	1	89256189	89272860	2955.831	-0.531	0.128	-4.156	3.24E-05	1.23E-03
216	12	121132819	121188032	180.061	-0.715	0.172	-4.149	3.34E-05	1.27E-03
217	22	39077067	39087743	633.879	-0.483	0.117	-4.146	3.39E-05	1.28E-03
218	X	101882288	101932031	913.433	-0.605	0.146	-4.136	3.53E-05	1.32E-03
219	22	39014257	39020352	765.472	-0.424	0.103	-4.133	3.58E-05	1.34E-03
220	16	27313668	27364778	2859.522	-0.403	0.098	-4.129	3.65E-05	1.36E-03
221	2	206159585	206162928	18429.640	-0.296	0.072	-4.122	3.75E-05	1.39E-03
222	1	19303965	19312144	302.199	-0.622	0.151	-4.111	3.94E-05	1.45E-03
223	9	34646631	34661886	363.221	-0.562	0.137	-4.110	3.95E-05	1.45E-03
224	17	67377281	67697256	1655.005	-0.404	0.099	-4.105	4.05E-05	1.47E-03
225	20	52972358	53495330	857.991	-0.582	0.142	-4.105	4.05E-05	1.47E-03
226	1	161037631	161038977	491.075	-0.613	0.149	-4.102	4.10E-05	1.48E-03
227	X	119786504	119791630	20994.938	-0.341	0.083	-4.099	4.15E-05	1.49E-03
228	13	40729128	40771190	675.751	-0.406	0.099	-4.090	4.31E-05	1.55E-03
229	2	85394753	85418432	292.104	-0.656	0.160	-4.087	4.37E-05	1.56E-03
230	X	136665547	136780932	3968.814	-0.294	0.072	-4.071	4.69E-05	1.67E-03
231	9	136975092	136981742	17.252	-1.918	0.472	-4.067	4.76E-05	1.68E-03
232	16	56608584	56609497	169.072	-0.646	0.159	-4.066	4.78E-05	1.68E-03
233	13	113820549	113864076	256.644	-0.896	0.220	-4.065	4.81E-05	1.69E-03
234	16	84565596	84618078	3196.151	-0.337	0.083	-4.053	5.06E-05	1.76E-03
235	11	47980559	48170839	1803.159	-0.360	0.089	-4.050	5.12E-05	1.78E-03
236	4	142023160	142847432	2416.165	-0.452	0.112	-4.048	5.17E-05	1.79E-03
237	15	40405485	40435947	796.384	-0.385	0.095	-4.044	5.26E-05	1.82E-03
238	6	70566917	70589569	119.093	-0.706	0.175	-4.042	5.30E-05	1.83E-03
239	1	44674692	44725591	533.895	-0.607	0.150	-4.040	5.34E-05	1.84E-03
240	X	37780059	37813461	82.186	-1.002	0.248	-4.034	5.49E-05	1.88E-03
241	17	7336529	7351477	4675.156	-0.451	0.112	-4.026	5.68E-05	1.94E-03

242	7	88205115	88226993	763.848	-0.372	0.093	-4.023	5.74E-05	1.95E-03
243	16	66988589	67009758	266.259	-0.625	0.155	-4.023	5.75E-05	1.95E-03
244	X	49171898	49175235	1444.328	-0.373	0.093	-4.021	5.80E-05	1.96E-03
245	15	74719542	74725536	24.122	-1.748	0.435	-4.016	5.92E-05	1.99E-03
246	12	56158346	56163496	3566.568	-0.324	0.081	-4.006	6.18E-05	2.06E-03
247	11	113314579	113383544	410.722	-0.501	0.125	-4.006	6.18E-05	2.06E-03
248	17	74203582	74210655	11015.740	-0.312	0.078	-4.005	6.20E-05	2.06E-03
249	7	56064002	56080670	2293.840	-0.303	0.076	-4.003	6.25E-05	2.07E-03
250	16	11547722	11636381	3427.360	-0.288	0.072	-4.002	6.28E-05	2.07E-03
251	22	42079691	42084284	936.837	-0.371	0.093	-4.000	6.33E-05	2.08E-03
252	2	85584431	85593406	179.871	-0.648	0.162	-3.998	6.40E-05	2.10E-03
253	6	32840717	32844679	1715.952	-0.454	0.114	-3.997	6.41E-05	2.10E-03
254	11	118339075	118342705	3208.634	-0.365	0.091	-3.997	6.43E-05	2.10E-03
255	19	12163064	12189871	435.809	-0.449	0.112	-3.994	6.49E-05	2.12E-03
256	21	44805617	44818779	1092.093	-0.347	0.087	-3.992	6.55E-05	2.13E-03
257	14	90396502	90408268	10507.298	-0.302	0.076	-3.980	6.89E-05	2.23E-03
258	4	56647988	56681899	335.071	-0.756	0.190	-3.972	7.13E-05	2.29E-03
259	6	31353872	31357188	32929.227	-0.807	0.204	-3.965	7.34E-05	2.35E-03
260	16	85690084	85751129	200.927	-0.631	0.160	-3.955	7.66E-05	2.43E-03
261	6	52361421	52407777	1089.519	-0.418	0.106	-3.952	7.76E-05	2.45E-03
262	14	24606480	24609699	40.136	-1.539	0.390	-3.951	7.78E-05	2.45E-03
263	9	127447674	127451406	22463.768	-0.290	0.073	-3.944	8.00E-05	2.51E-03
264	7	111091006	111125454	4577.737	-0.446	0.113	-3.944	8.02E-05	2.51E-03
265	6	29722775	29738528	2026.762	-1.246	0.316	-3.939	8.18E-05	2.55E-03
266	10	119499817	119542719	2088.651	-0.271	0.069	-3.934	8.35E-05	2.59E-03
267	14	68787660	68796253	3278.535	-0.375	0.095	-3.934	8.36E-05	2.59E-03
268	1	223220819	223364233	84.398	-0.950	0.242	-3.929	8.54E-05	2.64E-03
269	13	27424619	27435823	5959.497	-0.263	0.067	-3.923	8.76E-05	2.68E-03
270	1	169690665	169711702	16819.105	-0.285	0.073	-3.917	8.95E-05	2.74E-03
271	11	17208153	17349980	1389.826	-0.352	0.090	-3.914	9.08E-05	2.77E-03
272	7	135662496	135693418	466.344	-0.438	0.112	-3.909	9.28E-05	2.82E-03
273	1	155135344	155138857	629.978	-0.357	0.091	-3.908	9.30E-05	2.82E-03
274	9	109498325	110172512	350.571	-0.592	0.152	-3.899	9.64E-05	2.90E-03
275	2	241734602	241768816	594.910	-0.529	0.136	-3.897	9.76E-05	2.92E-03
276	11	95976598	96343195	1498.908	-0.368	0.095	-3.893	9.90E-05	2.96E-03

277	6	5261044	5829192	221.745	-0.527	0.135	-3.892	9.93E-05	2.96E-03
278	5	14581792	14616180	771.764	-0.351	0.090	-3.886	1.02E-04	3.02E-03
279	1	15571699	15585051	302.005	-0.619	0.159	-3.886	1.02E-04	3.02E-03
280	2	38743599	38751494	4906.948	-0.269	0.069	-3.885	1.02E-04	3.03E-03
281	X	48574449	48581162	3890.119	-0.232	0.060	-3.883	1.03E-04	3.04E-03
282	9	131258076	131276510	2342.424	-0.359	0.093	-3.877	1.06E-04	3.12E-03
283	12	56712305	56731628	14395.253	-0.298	0.077	-3.862	1.13E-04	3.30E-03
284	1	205712822	205750182	2429.565	-0.317	0.082	-3.861	1.13E-04	3.30E-03
285	17	6755447	6775647	998.188	-0.461	0.119	-3.859	1.14E-04	3.31E-03
286	16	30378135	30395991	2549.156	-0.438	0.114	-3.851	1.18E-04	3.40E-03
287	3	46201711	46208313	18.882	-2.204	0.573	-3.850	1.18E-04	3.41E-03
288	1	12166991	12209228	1880.823	-0.432	0.112	-3.844	1.21E-04	3.49E-03
289	22	39901084	39973721	1592.102	-0.323	0.084	-3.842	1.22E-04	3.51E-03
290	19	38867830	38878275	824.142	-0.377	0.098	-3.836	1.25E-04	3.55E-03
291	2	230203110	230313215	611.482	-0.399	0.104	-3.835	1.25E-04	3.56E-03
292	10	80078646	80092557	168.061	-0.554	0.144	-3.834	1.26E-04	3.57E-03
293	22	37125843	37175054	1193.084	-0.504	0.132	-3.832	1.27E-04	3.59E-03
294	3	9779967	9793011	1332.077	-0.324	0.085	-3.832	1.27E-04	3.59E-03
295	9	120902393	120929173	3185.300	-0.389	0.101	-3.830	1.28E-04	3.59E-03
296	2	84821650	84907008	2085.765	-0.508	0.133	-3.830	1.28E-04	3.59E-03
297	10	100486646	100519864	1126.085	-0.509	0.133	-3.820	1.34E-04	3.72E-03
298	1	19338775	19485539	4686.885	-0.281	0.074	-3.806	1.41E-04	3.91E-03
299	1	7954291	7985505	1661.073	-0.284	0.075	-3.805	1.42E-04	3.92E-03
300	19	35902529	35904377	492.838	-1.149	0.302	-3.804	1.43E-04	3.93E-03
301	11	117232625	117286454	662.077	-0.421	0.111	-3.802	1.44E-04	3.95E-03
302	16	17101769	17470960	1781.734	-0.341	0.090	-3.801	1.44E-04	3.95E-03
303	12	6786858	6820799	5962.862	-0.408	0.107	-3.797	1.46E-04	3.98E-03
304	11	68707440	68751520	61.533	-0.881	0.232	-3.797	1.46E-04	3.98E-03
305	6	32659467	32668383	374.552	-0.618	0.163	-3.796	1.47E-04	3.99E-03
306	20	32702699	32743467	368.037	-0.452	0.119	-3.792	1.49E-04	4.04E-03
307	7	23235967	23275108	81.706	-0.790	0.208	-3.790	1.51E-04	4.06E-03
308	10	129467190	129770983	178.060	-0.550	0.145	-3.789	1.51E-04	4.07E-03
309	17	48540894	48545109	498.993	-0.444	0.117	-3.788	1.52E-04	4.08E-03
310	14	91779746	91867536	4551.442	-0.368	0.098	-3.773	1.61E-04	4.27E-03
311	3	16313574	16514026	1061.182	-0.304	0.081	-3.766	1.66E-04	4.38E-03

312	11	118193725	118225094	3179.351	-0.339	0.090	-3.765	1.66E-04	4.38E-03
313	14	91060333	91225632	1184.488	-0.321	0.085	-3.765	1.66E-04	4.38E-03
314	2	171783405	171999859	300.888	-0.483	0.128	-3.764	1.67E-04	4.39E-03
315	7	44962662	44979088	1439.511	-0.326	0.087	-3.763	1.68E-04	4.40E-03
316	17	75776434	75785893	8485.511	-0.382	0.102	-3.761	1.69E-04	4.42E-03
317	22	37225270	37244448	4211.629	-0.411	0.109	-3.758	1.72E-04	4.46E-03
318	X	21372801	21654695	224.144	-0.541	0.144	-3.755	1.74E-04	4.50E-03
319	10	15102584	15168693	1292.484	-0.475	0.126	-3.754	1.74E-04	4.51E-03
320	7	45912245	45921874	188.927	-0.725	0.193	-3.751	1.76E-04	4.56E-03
321	19	52594060	52690504	1706.113	-0.491	0.131	-3.745	1.81E-04	4.65E-03
322	14	51826195	51979342	1644.144	-0.313	0.084	-3.744	1.81E-04	4.65E-03
323	5	149358037	149369653	591.022	-0.476	0.127	-3.744	1.81E-04	4.66E-03
324	6	85449584	85495791	188.571	-0.698	0.187	-3.741	1.83E-04	4.69E-03
325	19	38730187	38769904	161.813	-0.704	0.188	-3.741	1.84E-04	4.70E-03
326	11	22813799	22830299	907.658	-0.359	0.096	-3.739	1.85E-04	4.71E-03
327	2	189560590	189583758	941.506	-0.367	0.098	-3.736	1.87E-04	4.76E-03
328	6	3231403	3303373	341.045	-0.416	0.111	-3.734	1.88E-04	4.78E-03
329	19	43766533	43780976	423.870	-0.628	0.168	-3.734	1.88E-04	4.78E-03
330	12	92140278	92145846	8106.341	-0.280	0.075	-3.732	1.90E-04	4.81E-03
331	9	124257606	124353307	85.402	-0.782	0.210	-3.729	1.92E-04	4.84E-03
332	15	66498015	66524532	54197.361	-0.274	0.073	-3.727	1.94E-04	4.89E-03
333	X	102651092	102659083	1510.120	-0.374	0.100	-3.723	1.97E-04	4.94E-03
334	3	170864875	170870208	606.330	-0.361	0.097	-3.712	2.06E-04	5.13E-03
335	16	28984826	28990784	447.622	-0.569	0.153	-3.711	2.06E-04	5.13E-03
336	MT	9207	9990	65054.855	-0.369	0.100	-3.708	2.09E-04	5.18E-03
337	6	33204655	33206831	202.762	-0.636	0.172	-3.708	2.09E-04	5.18E-03
338	6	33004182	33009591	130.152	-0.790	0.213	-3.702	2.14E-04	5.28E-03
339	11	122838500	122872643	58.118	-1.106	0.299	-3.700	2.15E-04	5.28E-03
340	16	30472658	30523567	9634.608	-0.364	0.098	-3.699	2.17E-04	5.30E-03
341	4	672436	674330	680.061	-0.367	0.099	-3.698	2.17E-04	5.30E-03
342	9	97926791	97944856	157.885	-0.603	0.163	-3.698	2.17E-04	5.30E-03
343	10	110871795	110900006	5380.286	-0.342	0.093	-3.694	2.20E-04	5.37E-03
344	16	56989485	57083531	4440.453	-0.320	0.087	-3.693	2.22E-04	5.38E-03
345	6	35468401	35470785	22146.684	-0.251	0.068	-3.690	2.24E-04	5.41E-03
346	18	31129236	31162856	145.824	-0.903	0.245	-3.686	2.28E-04	5.47E-03

347	16	89575758	89597246	40.146	-1.104	0.300	-3.678	2.35E-04	5.64E-03
348	19	2230084	2237704	628.380	-0.359	0.098	-3.673	2.40E-04	5.74E-03
349	9	20341669	20622499	1199.089	-0.355	0.097	-3.667	2.45E-04	5.83E-03
350	X	47651796	47659180	1613.985	-0.305	0.083	-3.664	2.49E-04	5.91E-03
351	12	6444955	6451718	1711.063	-0.525	0.144	-3.660	2.52E-04	5.96E-03
352	16	1528688	1555580	2084.547	-0.348	0.095	-3.655	2.58E-04	6.07E-03
353	14	24143362	24147570	1668.301	-0.318	0.087	-3.654	2.59E-04	6.08E-03
354	22	41699503	41799456	494.577	-0.587	0.161	-3.653	2.59E-04	6.09E-03
355	15	78277835	78299703	621.372	-0.381	0.104	-3.653	2.60E-04	6.09E-03
356	12	52585589	52601538	769.010	-1.012	0.277	-3.649	2.63E-04	6.16E-03
357	14	103912288	103928269	1063.167	-0.325	0.089	-3.646	2.66E-04	6.20E-03
358	6	32821833	32838739	802.518	-0.430	0.118	-3.645	2.67E-04	6.21E-03
359	12	56116590	56117967	9423.318	-0.302	0.083	-3.643	2.70E-04	6.24E-03
360	5	100806933	100903282	315.903	-0.467	0.128	-3.640	2.72E-04	6.29E-03
361	14	58633967	58648321	137.913	-0.631	0.174	-3.639	2.74E-04	6.31E-03
362	4	79901146	80125454	1157.066	-0.301	0.083	-3.628	2.86E-04	6.54E-03
363	19	9853718	9936515	166.059	-0.619	0.171	-3.627	2.87E-04	6.56E-03
364	10	124461823	124617888	183.892	-0.523	0.144	-3.626	2.88E-04	6.58E-03
365	5	170246233	170297815	3417.877	-0.308	0.085	-3.623	2.91E-04	6.64E-03
366	7	150716606	150724284	2551.683	-0.385	0.106	-3.622	2.92E-04	6.64E-03
367	10	121957091	121975217	1245.830	-0.303	0.084	-3.621	2.94E-04	6.66E-03
368	13	48488963	48533256	845.602	-0.462	0.128	-3.619	2.96E-04	6.68E-03
369	5	60945177	61154531	266.579	-0.513	0.142	-3.616	2.99E-04	6.73E-03
370	6	29941260	29945884	21847.003	-0.410	0.113	-3.616	2.99E-04	6.73E-03
371	X	1462581	1537185	9110.599	-0.323	0.090	-3.603	3.14E-04	7.04E-03
372	19	42086110	42196585	1193.246	-0.430	0.119	-3.601	3.17E-04	7.10E-03
373	19	35904401	35908295	35.904	-1.354	0.376	-3.601	3.18E-04	7.10E-03
374	12	74537835	74545430	2591.302	-0.245	0.068	-3.596	3.23E-04	7.20E-03
375	12	49636499	49708165	873.545	-0.400	0.111	-3.596	3.23E-04	7.20E-03
376	5	147585438	147782775	108.577	-0.802	0.223	-3.591	3.30E-04	7.32E-03
377	11	126304060	126350005	320.431	-0.404	0.113	-3.589	3.32E-04	7.35E-03
378	1	151806071	151831845	239.610	-0.602	0.168	-3.588	3.33E-04	7.36E-03
379	6	33419661	33453689	1547.093	-0.329	0.092	-3.586	3.36E-04	7.40E-03
380	6	16129086	16148248	656.114	-0.416	0.116	-3.585	3.37E-04	7.42E-03
381	3	68004247	68545621	40.586	-1.123	0.314	-3.579	3.45E-04	7.56E-03

382	2	216498825	216579180	17824.562	-0.284	0.079	-3.578	3.46E-04	7.58E-03
383	12	103930107	103953931	8842.561	-0.305	0.085	-3.575	3.50E-04	7.64E-03
384	9	75088514	75147265	1103.506	-0.277	0.078	-3.569	3.58E-04	7.77E-03
385	1	161046946	161069970	7047.150	-0.264	0.074	-3.568	3.59E-04	7.79E-03
386	19	51371606	51372701	197.611	-0.739	0.207	-3.567	3.62E-04	7.80E-03
387	4	94298535	94342876	14.209	-2.066	0.580	-3.562	3.67E-04	7.88E-03
388	14	70641916	70675366	1222.655	-0.318	0.089	-3.553	3.81E-04	8.12E-03
389	7	76048051	76067508	1313.657	-0.309	0.087	-3.552	3.82E-04	8.12E-03
390	MT	3307	4262	28227.458	-0.304	0.086	-3.552	3.82E-04	8.12E-03
391	11	62433542	62556235	23957.668	-0.286	0.080	-3.552	3.82E-04	8.12E-03
392	X	119615724	119693370	7855.176	-0.264	0.074	-3.552	3.83E-04	8.12E-03
393	17	56593699	56595611	777.200	-0.906	0.255	-3.551	3.83E-04	8.12E-03
394	11	59142748	59155039	2042.331	-0.419	0.118	-3.546	3.90E-04	8.23E-03
395	19	49085419	49108605	8343.917	-0.390	0.110	-3.546	3.90E-04	8.23E-03
396	1	161166056	161178013	649.274	-0.370	0.105	-3.543	3.96E-04	8.30E-03
397	2	119366924	119372550	1106.646	-0.281	0.079	-3.543	3.96E-04	8.30E-03
398	16	981770	986979	112.471	-0.698	0.197	-3.540	4.01E-04	8.37E-03
399	2	218398256	218405941	2128.121	-0.733	0.207	-3.537	4.04E-04	8.44E-03
400	1	52602371	52609051	318.151	-0.435	0.123	-3.535	4.07E-04	8.47E-03
401	16	4474690	4510347	1125.298	-0.394	0.111	-3.532	4.13E-04	8.58E-03
402	7	44796680	44824564	11660.477	-0.245	0.069	-3.531	4.14E-04	8.58E-03
403	19	1259384	1274880	5348.078	-0.323	0.092	-3.530	4.16E-04	8.61E-03
404	8	90001405	90053633	727.381	-0.378	0.107	-3.529	4.18E-04	8.63E-03
405	17	439978	445939	1503.528	-0.393	0.111	-3.528	4.18E-04	8.63E-03
406	4	39452587	39458931	40652.073	-0.270	0.076	-3.527	4.20E-04	8.66E-03
407	2	85685175	85698852	386.651	-0.635	0.180	-3.522	4.29E-04	8.81E-03
408	17	64498254	64508199	25874.585	-0.279	0.079	-3.514	4.41E-04	9.02E-03
409	1	3487951	3611508	2601.191	-0.389	0.111	-3.509	4.51E-04	9.16E-03
410	20	47209214	47356889	839.836	-0.356	0.101	-3.508	4.51E-04	9.16E-03
411	12	118136124	118145584	2406.146	-0.248	0.071	-3.507	4.53E-04	9.17E-03
412	9	36136536	36163913	298.875	-0.434	0.124	-3.507	4.53E-04	9.17E-03
413	6	24804282	25042170	12389.742	-0.251	0.072	-3.504	4.57E-04	9.25E-03
414	11	128458761	128587558	31527.400	-0.275	0.079	-3.503	4.60E-04	9.28E-03
415	19	11574660	11579008	287.255	-0.513	0.147	-3.502	4.62E-04	9.30E-03
416	2	135905881	135986100	1447.530	-0.313	0.089	-3.499	4.67E-04	9.38E-03

417	2	109129205	109504634	134.216	-0.619	0.177	-3.497	4.70E-04	9.42E-03
418	3	39263495	39281735	94.900	-0.867	0.248	-3.496	4.72E-04	9.44E-03
419	2	10783391	10837977	1531.986	-0.269	0.077	-3.496	4.73E-04	9.44E-03
420	2	108894471	108989372	341.631	-0.466	0.133	-3.494	4.76E-04	9.49E-03
421	9	75060573	75088217	1538.813	-0.322	0.092	-3.492	4.79E-04	9.53E-03
422	MT	4470	5511	40274.364	-0.321	0.092	-3.488	4.86E-04	9.64E-03
423	X	130171962	130184870	192.929	-0.493	0.142	-3.482	4.98E-04	9.86E-03
424	17	82752042	82945914	2143.448	-0.341	0.098	-3.479	5.04E-04	9.96E-03
425	2	195575977	195737702	606.332	-0.499	0.144	-3.475	5.11E-04	1.009E-02
426	2	10120698	10211725	25.759	-1.393	0.402	-3.469	5.23E-04	1.026E-02
427	15	88877294	88895597	1126.054	-0.875	0.252	-3.467	5.26E-04	1.030E-02
428	12	48337180	48351414	858.688	-0.336	0.097	-3.466	5.29E-04	1.036E-02
429	20	57603846	57620576	146.759	-0.631	0.182	-3.460	5.39E-04	1.052E-02
430	6	30727709	30742732	2059.836	-0.311	0.090	-3.454	5.53E-04	1.075E-02
431	17	64319415	64413776	604.697	-0.348	0.101	-3.449	5.62E-04	1.090E-02
432	19	36139953	36150353	2997.339	-0.288	0.084	-3.448	5.65E-04	1.093E-02
433	1	150996549	151008376	537.127	-0.458	0.133	-3.445	5.72E-04	1.102E-02
434	1	211326615	211374946	1769.690	-0.322	0.094	-3.437	5.88E-04	1.127E-02
435	8	96645242	97149654	167.907	-0.646	0.188	-3.436	5.91E-04	1.131E-02
436	8	65714334	65842322	3753.794	-0.379	0.111	-3.432	5.99E-04	1.140E-02
437	19	4292232	4302431	317.683	-0.413	0.120	-3.431	6.01E-04	1.142E-02
438	13	30456704	30617597	4823.294	-0.290	0.084	-3.429	6.05E-04	1.146E-02
439	1	6460786	6466175	3981.629	-0.366	0.107	-3.427	6.11E-04	1.149E-02
440	20	43457893	43466046	3836.622	-0.316	0.092	-3.427	6.11E-04	1.149E-02
441	MT	8527	9207	40508.367	-0.328	0.096	-3.422	6.22E-04	1.164E-02
442	11	118304730	118316175	9697.905	-0.285	0.083	-3.419	6.27E-04	1.173E-02
443	16	188969	229463	2016.505	-0.298	0.087	-3.419	6.29E-04	1.174E-02
444	10	16590611	16817463	1761.609	-0.273	0.080	-3.418	6.32E-04	1.177E-02
445	5	132440440	132508719	1679.630	-0.357	0.105	-3.417	6.34E-04	1.177E-02
446	8	42849637	42897290	356.363	-0.488	0.143	-3.416	6.35E-04	1.177E-02
447	7	149872968	149891204	1373.297	-0.345	0.101	-3.406	6.58E-04	1.218E-02
448	7	1815793	2233243	158.854	-0.597	0.176	-3.403	6.66E-04	1.230E-02
449	3	112120839	112133270	385.060	-0.518	0.152	-3.403	6.67E-04	1.230E-02
450	12	14942031	14961728	17809.253	-0.247	0.073	-3.403	6.67E-04	1.230E-02
451	17	63432304	63446354	733.175	-0.355	0.104	-3.401	6.71E-04	1.234E-02

452	1	25616791	25786206	1274.362	-0.342	0.101	-3.399	6.75E-04	1.239E-02
453	21	43772511	43776330	1574.318	-0.235	0.069	-3.398	6.78E-04	1.243E-02
454	6	45880827	46080348	169.119	-0.543	0.160	-3.396	6.83E-04	1.249E-02
455	6	131135467	131283535	176.148	-0.726	0.214	-3.396	6.84E-04	1.249E-02
456	MT	10760	12137	72474.243	-0.297	0.088	-3.394	6.90E-04	1.255E-02
457	19	3539171	3544030	34.438	-1.262	0.372	-3.391	6.96E-04	1.262E-02
458	2	30231534	30323730	7993.416	-0.287	0.085	-3.385	7.12E-04	1.281E-02
459	10	89392546	89406487	141.582	-0.589	0.174	-3.384	7.14E-04	1.283E-02
460	15	44554818	44663688	2078.965	-0.312	0.092	-3.380	7.24E-04	1.298E-02
461	5	77691166	77868780	1402.157	-0.309	0.091	-3.375	7.38E-04	1.320E-02
462	17	74466399	74484794	307.524	-0.398	0.118	-3.363	7.72E-04	1.378E-02
463	1	92832013	92841924	35319.852	-0.220	0.066	-3.360	7.80E-04	1.387E-02
464	21	44885953	44931989	3137.265	-0.445	0.133	-3.354	7.98E-04	1.408E-02
465	9	15163622	15307360	637.413	-0.358	0.107	-3.353	8.01E-04	1.410E-02
466	7	44606572	44709066	2085.876	-0.291	0.087	-3.349	8.10E-04	1.421E-02
467	6	36948263	36964837	171.678	-0.800	0.239	-3.349	8.10E-04	1.421E-02
468	4	6663396	6676755	1157.256	-0.298	0.089	-3.341	8.35E-04	1.452E-02
469	7	114922094	115019917	1760.533	-0.328	0.098	-3.339	8.42E-04	1.460E-02
470	15	90229975	90234047	1494.838	-0.286	0.086	-3.331	8.66E-04	1.499E-02
471	3	59747277	61251459	1430.188	-0.740	0.222	-3.329	8.72E-04	1.504E-02
472	18	63123346	63320128	2437.363	-0.285	0.086	-3.326	8.82E-04	1.515E-02
473	11	108223044	108369102	15503.815	-0.297	0.089	-3.326	8.82E-04	1.515E-02
474	4	88725955	89111398	380.294	-0.546	0.164	-3.322	8.93E-04	1.529E-02
475	11	36296288	36465204	235.026	-0.461	0.139	-3.322	8.93E-04	1.529E-02
476	5	131949973	132012243	694.451	-0.439	0.132	-3.321	8.97E-04	1.534E-02
477	2	47160084	47176921	3730.200	-0.280	0.084	-3.319	9.03E-04	1.542E-02
478	10	125823546	125853695	815.876	-0.280	0.084	-3.316	9.13E-04	1.553E-02
479	5	157395534	157575775	679.105	-0.326	0.098	-3.316	9.13E-04	1.553E-02
480	1	22025511	22101360	5588.905	-0.247	0.074	-3.313	9.24E-04	1.566E-02
481	X	101009346	101052116	745.292	-0.360	0.109	-3.312	9.25E-04	1.566E-02
482	14	49618530	49620626	4464.379	-0.243	0.073	-3.311	9.29E-04	1.572E-02
483	8	28021964	28083936	63.843	-0.794	0.240	-3.311	9.31E-04	1.572E-02
484	11	4384897	4393702	598.900	-0.335	0.101	-3.310	9.34E-04	1.573E-02
485	7	5879827	5886363	108.898	-0.791	0.239	-3.310	9.34E-04	1.573E-02
486	7	96481626	96709880	602.653	-0.359	0.109	-3.299	9.69E-04	1.624E-02

487	3	15254353	15341368	1054.516	-0.321	0.098	-3.292	9.95E-04	1.657E-02
488	7	150625375	150632648	7249.542	-0.292	0.089	-3.292	9.96E-04	1.657E-02
489	11	65714005	65720818	1027.088	-0.292	0.089	-3.289	1.01E-03	1.670E-02
490	22	45671798	45845307	861.187	-0.316	0.096	-3.286	1.02E-03	1.682E-02
491	2	130356045	130375405	1344.668	-0.300	0.091	-3.283	1.03E-03	1.691E-02
492	3	101681091	101686718	14025.183	-0.219	0.067	-3.282	1.03E-03	1.691E-02
493	22	31281594	31292534	8293.329	-0.277	0.085	-3.282	1.03E-03	1.691E-02
494	12	112405189	112418838	28814.160	-0.230	0.070	-3.282	1.03E-03	1.691E-02
495	22	19351368	19447711	503.133	-0.346	0.106	-3.280	1.04E-03	1.699E-02
496	6	31268749	31272130	14164.397	-0.780	0.238	-3.279	1.04E-03	1.699E-02
497	3	194394821	194399266	79.106	-0.679	0.207	-3.277	1.05E-03	1.709E-02
498	11	117986370	118003037	6812.684	-0.336	0.103	-3.270	1.07E-03	1.748E-02
499	6	99397629	99425331	7489.888	-0.332	0.102	-3.267	1.09E-03	1.764E-02
500	2	151247940	151261863	344.552	-0.569	0.174	-3.264	1.10E-03	1.779E-02
501	16	89720400	89740925	2854.175	-0.262	0.080	-3.263	1.10E-03	1.779E-02
502	1	159918107	159925507	3398.455	-0.327	0.100	-3.256	1.13E-03	1.821E-02
503	11	65823022	65862026	8098.359	-0.264	0.081	-3.253	1.14E-03	1.834E-02
504	19	54448887	54462016	8015.241	-0.353	0.109	-3.251	1.15E-03	1.843E-02
505	22	31325804	31346346	890.343	-0.404	0.124	-3.249	1.16E-03	1.853E-02
506	10	128096659	128126423	43.584	-1.055	0.325	-3.243	1.18E-03	1.887E-02
507	19	10111693	10115372	153.015	-0.555	0.171	-3.241	1.19E-03	1.895E-02
508	14	24311450	24318036	412.833	-0.388	0.120	-3.239	1.20E-03	1.906E-02
509	6	31580525	31582522	7792.351	-0.329	0.102	-3.231	1.23E-03	1.955E-02
510	14	105472962	105480162	55.943	-1.031	0.320	-3.226	1.26E-03	1.986E-02
511	6	32190766	32195523	5005.723	-0.325	0.101	-3.224	1.27E-03	1.997E-02
512	9	7796500	7888380	497.176	-0.367	0.114	-3.222	1.27E-03	2.006E-02
513	16	67277510	67289499	508.328	-0.417	0.130	-3.220	1.28E-03	2.012E-02
514	1	151870866	151909637	4449.906	-0.297	0.092	-3.220	1.28E-03	2.012E-02
515	19	12317477	12333720	192.327	-0.452	0.140	-3.220	1.28E-03	2.012E-02
516	X	47623172	47630305	167.104	-0.574	0.178	-3.220	1.28E-03	2.012E-02
517	7	110662644	111562517	90.487	-0.580	0.180	-3.219	1.29E-03	2.018E-02
518	10	73812501	73874591	1373.040	-0.338	0.105	-3.217	1.30E-03	2.026E-02
519	13	50909747	51024120	938.947	-0.299	0.093	-3.216	1.30E-03	2.030E-02
520	3	9757347	9769992	21.402	-1.211	0.377	-3.216	1.30E-03	2.030E-02
521	11	124633113	124695707	145.510	-0.597	0.186	-3.215	1.31E-03	2.034E-02

522	11	75260122	75351705	466.864	-0.397	0.124	-3.214	1.31E-03	2.034E-02
523	11	61768501	61792802	516.358	-0.329	0.102	-3.214	1.31E-03	2.034E-02
524	17	40553769	40565472	4552.059	-0.320	0.100	-3.213	1.32E-03	2.036E-02
525	17	5499427	5619424	6562.386	-0.369	0.115	-3.209	1.33E-03	2.056E-02
526	17	76868404	76950393	21.048	-1.439	0.449	-3.205	1.35E-03	2.071E-02
527	19	46601074	46610782	4555.528	-0.317	0.099	-3.205	1.35E-03	2.071E-02
528	1	198638457	198757476	27100.521	-0.227	0.071	-3.205	1.35E-03	2.071E-02
529	16	28474111	28495575	178.849	-0.451	0.141	-3.200	1.37E-03	2.096E-02
530	4	174490175	174523154	88.779	-0.787	0.246	-3.198	1.38E-03	2.113E-02
531	3	3144628	3179727	1902.657	-0.296	0.093	-3.195	1.40E-03	2.130E-02
532	4	40191053	40246967	2514.539	-0.282	0.088	-3.188	1.43E-03	2.170E-02
533	11	30830369	31369810	21.664	-1.736	0.545	-3.183	1.46E-03	2.208E-02
534	19	6887566	6940459	248.227	-0.537	0.169	-3.182	1.46E-03	2.209E-02
535	10	22316386	22320306	227.059	-0.540	0.171	-3.169	1.53E-03	2.299E-02
536	6	158650014	158764876	194.062	-0.493	0.156	-3.166	1.54E-03	2.315E-02
537	13	25371974	26025851	112.813	-0.641	0.202	-3.165	1.55E-03	2.320E-02
538	14	61187559	61550976	3711.753	-0.208	0.066	-3.164	1.56E-03	2.327E-02
539	17	37406886	37479725	478.790	-0.304	0.096	-3.164	1.56E-03	2.327E-02
540	9	98069275	98119222	1787.102	-0.281	0.089	-3.162	1.57E-03	2.341E-02
541	5	134402065	134411881	269.089	-0.384	0.122	-3.158	1.59E-03	2.368E-02
542	14	20468954	20477089	1242.482	-0.259	0.082	-3.158	1.59E-03	2.368E-02
543	6	26457904	26476621	1414.824	-0.263	0.083	-3.153	1.62E-03	2.403E-02
544	6	116100851	116249497	712.785	-0.363	0.115	-3.150	1.63E-03	2.416E-02
545	6	30717435	30725538	5007.264	-0.196	0.062	-3.149	1.64E-03	2.420E-02
546	4	625573	670782	202.585	-0.476	0.151	-3.146	1.65E-03	2.438E-02
547	20	58985686	59008238	343.614	-0.382	0.121	-3.146	1.66E-03	2.440E-02
548	3	9749944	9788219	300.557	-0.365	0.116	-3.144	1.67E-03	2.453E-02
549	4	86594315	86815171	338.009	-0.366	0.117	-3.142	1.68E-03	2.468E-02
550	11	121452314	121633763	16843.203	-0.282	0.090	-3.140	1.69E-03	2.475E-02
551	17	28879335	28897733	3858.471	-0.317	0.101	-3.138	1.70E-03	2.491E-02
552	9	75890644	76362975	223.550	-0.475	0.152	-3.138	1.70E-03	2.492E-02
553	1	16440721	16460078	3149.295	-0.223	0.071	-3.137	1.71E-03	2.494E-02
554	20	63956704	63969930	669.358	-0.367	0.117	-3.134	1.72E-03	2.515E-02
555	1	160739057	160754821	210.831	-0.483	0.154	-3.134	1.73E-03	2.518E-02
556	14	90847861	91060641	660.959	-0.321	0.102	-3.131	1.74E-03	2.537E-02

557	22	37204237	37212477	88.690	-0.805	0.257	-3.130	1.75E-03	2.542E-02
558	3	122577628	122639047	1360.528	-0.355	0.114	-3.126	1.77E-03	2.553E-02
559	8	41488200	41511095	1735.240	-0.260	0.083	-3.126	1.77E-03	2.553E-02
560	4	6640091	6642729	2204.267	-0.211	0.067	-3.124	1.79E-03	2.566E-02
561	2	218217141	218254356	7216.267	-0.175	0.056	-3.124	1.79E-03	2.566E-02
562	22	24952716	25197448	306.966	-0.374	0.120	-3.121	1.80E-03	2.583E-02
563	16	74671855	74700960	515.336	-0.320	0.102	-3.121	1.80E-03	2.583E-02
564	4	48066393	48134250	801.833	-0.383	0.123	-3.118	1.82E-03	2.603E-02
565	12	110434823	110450422	3045.850	-0.227	0.073	-3.117	1.83E-03	2.605E-02
566	5	50665899	50846519	2035.179	-0.298	0.096	-3.113	1.85E-03	2.636E-02
567	17	39200283	39204840	43375.856	-0.234	0.075	-3.110	1.87E-03	2.655E-02
568	2	178525989	178830802	282.541	-0.529	0.171	-3.102	1.92E-03	2.715E-02
569	18	45983536	46072272	163.493	-0.548	0.177	-3.102	1.92E-03	2.715E-02
570	8	22578279	22598025	566.952	-0.363	0.117	-3.102	1.92E-03	2.718E-02
571	21	45405165	45513720	437.428	-0.565	0.182	-3.096	1.96E-03	2.757E-02
572	9	125234853	125241382	5509.378	-0.237	0.076	-3.095	1.97E-03	2.764E-02
573	20	36612318	36646196	676.306	-0.387	0.125	-3.091	1.99E-03	2.793E-02
574	2	191678068	191741097	705.600	-0.380	0.123	-3.090	2.00E-03	2.796E-02
575	X	44147872	44343672	43.534	-1.093	0.354	-3.090	2.00E-03	2.801E-02
576	12	112938051	112976460	737.322	-0.325	0.105	-3.087	2.02E-03	2.816E-02
577	10	93496612	93529092	10.714	-1.867	0.605	-3.087	2.02E-03	2.816E-02
578	8	124550784	124728473	1025.635	-0.356	0.115	-3.087	2.02E-03	2.816E-02
579	13	21671073	21704498	94.407	-0.626	0.203	-3.084	2.04E-03	2.831E-02
580	13	20403666	20525873	167.562	-0.505	0.164	-3.082	2.05E-03	2.848E-02
581	19	18571730	18577550	13443.413	-0.226	0.073	-3.082	2.06E-03	2.852E-02
582	2	95402708	95416616	265.788	-0.380	0.123	-3.080	2.07E-03	2.861E-02
583	17	76142465	76240493	1163.154	-0.284	0.092	-3.079	2.08E-03	2.869E-02
584	20	18567347	18569563	248.661	-0.398	0.129	-3.076	2.10E-03	2.893E-02
585	X	141111605	141177129	200.335	-0.410	0.133	-3.075	2.11E-03	2.895E-02
586	7	140023749	140062951	1262.408	-0.239	0.078	-3.075	2.11E-03	2.895E-02
587	7	100159244	100168617	527.137	-0.475	0.155	-3.074	2.11E-03	2.895E-02
588	1	196652043	196747504	194.660	-0.400	0.130	-3.074	2.11E-03	2.895E-02
589	1	109494052	109502932	1062.032	-0.250	0.081	-3.074	2.11E-03	2.895E-02
590	8	42541155	42555195	582.581	-0.328	0.107	-3.072	2.13E-03	2.909E-02
591	17	63477061	63498380	109.780	-0.825	0.269	-3.072	2.13E-03	2.909E-02

592	14	20455191	20457772	2395.165	-0.254	0.083	-3.070	2.14E-03	2.920E-02
593	10	8045378	8075198	1075.455	-0.275	0.089	-3.068	2.15E-03	2.924E-02
594	19	9959561	10010504	201.994	-0.733	0.239	-3.068	2.15E-03	2.924E-02
595	6	118460772	118710075	1250.842	-0.394	0.129	-3.068	2.15E-03	2.925E-02
596	21	42403447	42447684	1792.422	-0.342	0.112	-3.063	2.19E-03	2.972E-02
597	20	36890229	36951893	9241.343	-0.206	0.067	-3.062	2.20E-03	2.975E-02
598	X	10015254	10144474	921.966	-0.283	0.093	-3.059	2.22E-03	3.002E-02
599	1	150321479	150353233	1798.099	-0.256	0.084	-3.058	2.22E-03	3.002E-02
600	5	53480626	53487134	340.950	-0.511	0.167	-3.055	2.25E-03	3.035E-02
601	3	184710364	184712064	595.883	-0.307	0.101	-3.052	2.27E-03	3.056E-02
602	1	108648290	108661526	507.964	-0.291	0.095	-3.050	2.29E-03	3.078E-02
603	6	136256627	136289851	3652.601	-0.304	0.100	-3.048	2.30E-03	3.090E-02
604	17	35871491	35880793	2162.987	-0.367	0.120	-3.046	2.32E-03	3.099E-02
605	16	53433977	53491648	10509.915	-0.266	0.087	-3.045	2.33E-03	3.114E-02
606	8	22048995	22082527	194.174	-0.440	0.145	-3.041	2.36E-03	3.147E-02
607	8	42152946	42171673	882.812	-0.277	0.091	-3.041	2.36E-03	3.147E-02
608	1	9234774	9271337	1025.467	-0.318	0.105	-3.036	2.39E-03	3.178E-02
609	5	76403285	76708132	2288.113	-0.234	0.077	-3.035	2.40E-03	3.185E-02
610	21	46098112	46132848	628.971	-0.482	0.159	-3.032	2.43E-03	3.217E-02
611	3	42489299	42537573	1702.178	-0.361	0.119	-3.030	2.45E-03	3.232E-02
612	8	133454848	133571940	3478.537	-0.223	0.073	-3.030	2.45E-03	3.232E-02
613	19	49496365	49499708	37418.352	-0.281	0.093	-3.029	2.46E-03	3.239E-02
614	3	122384161	122412334	448.688	-0.376	0.124	-3.023	2.50E-03	3.289E-02
615	19	39863323	39934626	987.294	-0.445	0.147	-3.021	2.52E-03	3.304E-02
616	X	108044970	108079184	711.091	-0.337	0.111	-3.020	2.53E-03	3.304E-02
617	15	68778535	68820897	2645.889	-0.216	0.072	-3.020	2.53E-03	3.304E-02
618	6	2948159	2972165	802.006	-0.382	0.127	-3.019	2.54E-03	3.317E-02
619	12	39550033	39619803	322.508	-0.379	0.126	-3.017	2.55E-03	3.326E-02
620	2	215996329	216082955	237.752	-0.367	0.122	-3.017	2.55E-03	3.329E-02
621	4	73740541	73743716	51.731	-1.261	0.418	-3.015	2.57E-03	3.338E-02
622	2	237966827	238042782	283.364	-0.390	0.130	-3.014	2.58E-03	3.347E-02
623	9	134903232	134917912	11.195	-1.873	0.622	-3.011	2.60E-03	3.369E-02
624	4	17460261	17512206	216.250	-0.400	0.133	-3.011	2.61E-03	3.372E-02
625	16	29662979	29670876	3567.919	-0.218	0.073	-3.006	2.65E-03	3.419E-02
626	22	38483438	38507660	20188.172	-0.272	0.091	-3.004	2.66E-03	3.433E-02

627	12	53157663	53180925	501.295	-0.310	0.103	-3.001	2.69E-03	3.465E-02
628	6	20401879	20493714	1339.483	-0.344	0.115	-3.000	2.70E-03	3.473E-02
629	17	50478860	50485974	1318.915	-0.303	0.101	-2.995	2.74E-03	3.525E-02
630	1	151156649	151159749	662.486	-0.393	0.131	-2.994	2.76E-03	3.535E-02
631	5	53560633	53683338	318.088	-0.351	0.117	-2.990	2.79E-03	3.572E-02
632	17	40121971	40136917	3349.491	-0.197	0.066	-2.989	2.79E-03	3.573E-02
633	2	74458400	74460891	213.939	-0.405	0.135	-2.986	2.83E-03	3.606E-02
634	12	102073103	102120120	216.055	-0.432	0.145	-2.985	2.83E-03	3.606E-02
635	2	70295975	70302090	941.638	-0.338	0.113	-2.985	2.83E-03	3.606E-02
636	10	15211643	15371289	144.935	-0.563	0.189	-2.984	2.84E-03	3.609E-02
637	X	2219506	2502805	146.298	-0.443	0.148	-2.983	2.85E-03	3.620E-02
638	3	172505508	172523475	825.924	-0.264	0.088	-2.982	2.87E-03	3.632E-02
639	12	55743122	55757264	419.610	-0.297	0.100	-2.977	2.91E-03	3.682E-02
640	6	12290361	12297194	13.133	-1.687	0.567	-2.977	2.92E-03	3.687E-02
641	1	151982915	151993859	2328.236	-0.295	0.099	-2.975	2.93E-03	3.692E-02
642	1	20482391	20486210	135.706	-0.632	0.212	-2.974	2.94E-03	3.697E-02
643	X	129779949	129795201	2576.128	-0.309	0.104	-2.973	2.95E-03	3.704E-02
644	2	236567787	236582354	60.750	-0.769	0.259	-2.972	2.96E-03	3.715E-02
645	MT	10470	10766	6119.181	-0.272	0.091	-2.971	2.96E-03	3.715E-02
646	2	27771717	27988087	537.241	-0.266	0.090	-2.969	2.99E-03	3.748E-02
647	12	6650301	6663142	1033.723	-0.264	0.089	-2.966	3.02E-03	3.769E-02
648	1	247416156	247449108	188.114	-0.521	0.176	-2.964	3.04E-03	3.790E-02
649	5	134758771	134855133	2273.186	-0.251	0.085	-2.963	3.05E-03	3.798E-02
650	1	39026318	39034636	1013.950	-0.268	0.090	-2.962	3.05E-03	3.798E-02
651	13	75525214	75549439	1589.539	-0.296	0.100	-2.961	3.07E-03	3.816E-02
652	3	112921205	112975103	319.442	-0.377	0.127	-2.957	3.11E-03	3.841E-02
653	7	77193369	77199848	88.693	-0.667	0.226	-2.957	3.11E-03	3.841E-02
654	X	154401236	154412112	371.939	-0.481	0.163	-2.957	3.11E-03	3.841E-02
655	1	25543606	25568886	4832.770	-0.294	0.100	-2.956	3.12E-03	3.847E-02
656	8	73290242	73295789	41821.711	-0.215	0.073	-2.955	3.13E-03	3.851E-02
657	7	100154420	100158723	1056.310	-0.299	0.101	-2.954	3.13E-03	3.857E-02
658	15	78921058	78949574	376.229	-0.419	0.142	-2.952	3.16E-03	3.875E-02
659	11	124747474	124752255	26.491	-1.016	0.345	-2.949	3.19E-03	3.899E-02
660	11	76657524	76670747	29.524	-1.100	0.373	-2.945	3.23E-03	3.935E-02
661	20	2835314	2841190	1089.430	-0.454	0.154	-2.944	3.24E-03	3.945E-02

662	19	45162928	45178237	320.570	-0.449	0.153	-2.942	3.26E-03	3.963E-02
663	13	29764371	29850617	1333.411	-0.285	0.097	-2.942	3.27E-03	3.963E-02
664	5	140125937	140129392	170.360	-0.544	0.185	-2.936	3.33E-03	4.025E-02
665	4	82422565	82430462	11112.034	-0.205	0.070	-2.933	3.36E-03	4.054E-02
666	9	35649295	35650931	687.316	-0.336	0.114	-2.931	3.38E-03	4.068E-02
667	2	99545419	100192428	107.564	-0.677	0.232	-2.925	3.45E-03	4.140E-02
668	9	137039463	137046179	250.130	-0.446	0.152	-2.924	3.45E-03	4.140E-02
669	13	75283503	75482169	4381.688	-0.274	0.094	-2.924	3.46E-03	4.140E-02
670	3	129167827	129183922	7537.590	-0.187	0.064	-2.917	3.53E-03	4.208E-02
671	1	159062484	159147096	73.149	-0.691	0.237	-2.914	3.56E-03	4.240E-02
672	17	50719565	50756219	6042.428	-0.327	0.112	-2.914	3.57E-03	4.240E-02
673	5	134148935	134176964	2346.800	-0.289	0.099	-2.913	3.58E-03	4.249E-02
674	7	141002610	141015228	597.674	-0.295	0.101	-2.912	3.59E-03	4.262E-02
675	5	139341587	139369720	2517.960	-0.251	0.086	-2.911	3.60E-03	4.265E-02
676	12	120469850	120498493	927.314	-0.257	0.088	-2.911	3.60E-03	4.265E-02
677	6	33272075	33276511	45577.741	-0.210	0.072	-2.911	3.60E-03	4.265E-02
678	3	143265222	143848485	235.717	-0.404	0.139	-2.909	3.62E-03	4.278E-02
679	8	143833583	143840973	330.249	-0.417	0.143	-2.909	3.63E-03	4.278E-02
680	19	17824780	17848071	2123.566	-0.293	0.101	-2.908	3.64E-03	4.283E-02
681	22	44172956	44219533	2530.650	-0.350	0.120	-2.907	3.65E-03	4.293E-02
682	16	57610652	57665580	67.042	-0.805	0.277	-2.905	3.68E-03	4.317E-02
683	1	26529761	26575030	2045.935	-0.226	0.078	-2.903	3.69E-03	4.326E-02
684	9	5357971	5437925	233.141	-0.403	0.139	-2.902	3.70E-03	4.335E-02
685	3	112463966	112499472	195.469	-0.454	0.157	-2.900	3.74E-03	4.363E-02
686	18	46080248	46104334	6224.733	-0.175	0.060	-2.897	3.77E-03	4.394E-02
687	7	43758680	43807342	206.755	-0.451	0.156	-2.897	3.77E-03	4.396E-02
688	12	1791963	1918666	181.758	-0.445	0.154	-2.896	3.78E-03	4.399E-02
689	9	137204082	137205648	696.569	-0.258	0.089	-2.893	3.82E-03	4.427E-02
690	13	110641412	110713603	1169.610	-0.246	0.085	-2.893	3.82E-03	4.427E-02
691	1	84925583	84997113	124.463	-0.486	0.168	-2.892	3.83E-03	4.436E-02
692	17	30769388	30824692	2028.441	-0.221	0.076	-2.890	3.85E-03	4.449E-02
693	14	99169287	99272197	3882.834	-0.275	0.095	-2.890	3.86E-03	4.449E-02
694	11	61333220	61353295	304.371	-0.346	0.120	-2.889	3.87E-03	4.451E-02
695	8	81280536	81284777	82.130	-0.581	0.201	-2.887	3.88E-03	4.463E-02
696	18	24426634	24453531	333.685	-0.392	0.136	-2.887	3.89E-03	4.463E-02

697	X	56818298	56995827	548.386	-0.349	0.121	-2.884	3.93E-03	4.499E-02
698	5	107859035	108382098	292.632	-0.413	0.143	-2.883	3.94E-03	4.516E-02
699	3	183122215	183163839	29.956	-1.189	0.412	-2.882	3.95E-03	4.518E-02
700	2	85837120	85905199	411.472	-0.385	0.134	-2.880	3.98E-03	4.543E-02
701	15	75639085	75648706	1227.514	-0.216	0.075	-2.878	4.00E-03	4.551E-02
702	X	48521799	48528716	192.291	-0.422	0.147	-2.875	4.04E-03	4.590E-02
703	11	95066919	95071225	2384.240	-0.214	0.075	-2.871	4.09E-03	4.638E-02
704	1	39692182	39763914	1127.396	-0.231	0.080	-2.871	4.10E-03	4.640E-02
705	6	26055740	26056470	161.208	-0.446	0.155	-2.870	4.10E-03	4.640E-02
706	16	31180138	31191605	8640.823	-0.222	0.077	-2.870	4.11E-03	4.644E-02
707	6	158168350	158199344	331.894	-0.362	0.126	-2.869	4.12E-03	4.649E-02
708	1	156009048	156020951	4563.404	-0.216	0.075	-2.867	4.14E-03	4.668E-02
709	1	114716913	114758676	9024.500	-0.184	0.064	-2.863	4.20E-03	4.723E-02
710	6	37170152	37175428	2709.781	-0.233	0.082	-2.860	4.23E-03	4.758E-02
711	4	2269582	2418651	352.870	-0.333	0.116	-2.859	4.25E-03	4.772E-02
712	19	39412669	39428415	447.362	-0.339	0.118	-2.859	4.25E-03	4.772E-02
713	10	70597348	70602759	437.593	-0.389	0.136	-2.859	4.26E-03	4.772E-02
714	19	4890437	4902896	129.824	-0.603	0.211	-2.856	4.29E-03	4.806E-02
715	5	150442635	150449739	30165.159	-0.234	0.082	-2.854	4.32E-03	4.829E-02
716	12	57472264	57488814	3565.938	-0.322	0.113	-2.852	4.35E-03	4.851E-02
717	3	187368385	187372076	103.478	-0.638	0.224	-2.852	4.35E-03	4.853E-02
718	7	100177563	100221488	263.318	-0.412	0.145	-2.849	4.39E-03	4.889E-02
719	X	81202102	81298547	1127.117	-0.329	0.115	-2.847	4.42E-03	4.914E-02
720	12	109553715	109573580	201.478	-0.501	0.176	-2.846	4.43E-03	4.919E-02
721	3	27715949	27722711	130.733	-0.526	0.185	-2.845	4.43E-03	4.925E-02
722	2	230165186	230225729	1706.526	-0.220	0.077	-2.843	4.47E-03	4.953E-02
723	1	165662216	165698562	716.088	-0.248	0.087	-2.841	4.50E-03	4.976E-02

b

Order	Ensembl ID	Gene Symbol	Gene Name	UniProt
1	ENSG00000181019	***NQO1***	NAD(P)H quinone dehydrogenase 1	P15559
2	ENSG00000113369	***ARRDC3***	arrestin domain containing 3	Q96B67
3	ENSG00000110090	***CPT1A***	carnitine palmitoyltransferase 1A	P50416
4	ENSG00000103257	***SLC7A5***	solute carrier family 7 member 5	Q01650
5	ENSG00000115758	***ODC1***	ornithine decarboxylase 1	P11926
6	ENSG00000178537	***SLC25A20***	solute carrier family 25 member 20	O43772
7	ENSG00000090861	***AARS1***	alanyl-tRNA synthetase 1	P49588
8	ENSG00000135069	***PSAT1***	phosphoserine aminotransferase 1	Q9Y617
9	ENSG00000001084	***GCLC***	glutamate-cysteine ligase catalytic subunit	P48506
10	ENSG00000167703	***SLC43A2***	solute carrier family 43 member 2	Q8N370
11	ENSG00000125534	***PPDPF***	pancreatic progenitor cell differentiation and proliferation factor	Q9H3Y8
12	ENSG00000172613	***RAD9A***	RAD9 checkpoint clamp component A	Q99638
13	ENSG00000139514	***SLC7A1***	solute carrier family 7 member 1	P30825
14	ENSG00000051108	***HERPUD1***	homocysteine inducible ER protein with ubiquitin like domain 1	Q15011
15	ENSG00000197063	***MAFG***	MAF bZIP transcription factor G	O15525
16	ENSG00000116852	***KIF21B***	kinesin family member 21B	O75037
17	ENSG00000100292	***HMOX1***	heme oxygenase 1	P09601
18	ENSG00000198830	***HMGN2***	high mobility group nucleosomal binding domain 2	P05204
19	ENSG00000113407	***TARS1***	threonyl-tRNA synthetase 1	P26639
20	ENSG00000162413	***KLHL21***	kelch like family member 21	Q9UJP4
21	ENSG00000168003	***SLC3A2***	solute carrier family 3 member 2	P08195
22	ENSG00000072778	***ACADVL***	acyl-CoA dehydrogenase very long chain	P49748
23	ENSG00000196305	***IARS1***	isoleucyl-tRNA synthetase 1	P41252
24	ENSG00000128965	***CHAC1***	ChaC glutathione specific gamma-glutamylcyclotransferase 1	Q9BUX1
25	ENSG00000082898	***XPO1***	exportin 1	O14980
26	ENSG00000139112	***GABARAPL1***	GABA type A receptor associated protein like 1	Q9H0R8
27	ENSG00000087086	***FTL***	ferritin light chain	P02792
28	ENSG00000134294	***SLC38A2***	solute carrier family 38 member 2	Q96QD8
29	ENSG00000166986	***MARS1***	methionyl-tRNA synthetase 1	P56192
30	ENSG00000198743	***SLC5A3***	solute carrier family 5 member 3	P53794
31	ENSG00000172534	***HCFC1***	host cell factor C1	P51610

32	ENSG00000112715	***VEGFA***	vascular endothelial growth factor A	P15692
33	ENSG00000134109	***EDEM1***	ER degradation enhancing alpha-mannosidase like protein 1	Q92611
34	ENSG00000092621	***PHGDH***	phosphoglycerate dehydrogenase	O43175
35	ENSG00000112195	***TREML2***	triggering receptor expressed on myeloid cells like 2	Q5T2D2
36	ENSG00000103249	***CLCN7***	chloride voltage-gated channel 7	P51798
37	ENSG00000105281	***SLC1A5***	solute carrier family 1 member 5	Q15758
38	ENSG00000110619	***CARS1***	cysteinyl-tRNA synthetase 1	P49589
39	ENSG00000166501	***PRKCB***	protein kinase C beta	P05771
40	ENSG00000143815	***LBR***	lamin B receptor	Q14739
41	ENSG00000196517	***SLC6A9***	solute carrier family 6 member 9	P48067
42	ENSG00000138678	***GPAT3***	glycerol-3-phosphate acyltransferase 3	Q53EU6
43	ENSG00000185633	***NDUFA4L2***	NDUFA4 mitochondrial complex associated like 2	Q9NRX3
44	ENSG00000174130	***TLR6***	toll like receptor 6	Q9Y2C9
45	ENSG00000153879	***CEBPG***	CCAAT enhancer binding protein gamma	P53567
46	ENSG00000111981	***ULBP1***	UL16 binding protein 1	Q9BZM6
47	ENSG00000146540	***C7orf50***	chromosome 7 open reading frame 50	Q9BRJ6
48	ENSG00000171503	***ETFDH***	electron transfer flavoprotein dehydrogenase	Q16134
49	ENSG00000198431	***TXNRD1***	thioredoxin reductase 1	Q16881
50	ENSG00000106105	***GARS1***	glycyl-tRNA synthetase 1	P41250
51	ENSG00000161011	***SQSTM1***	sequestosome 1	Q13501
52	ENSG00000162616	***DNAJB4***	DnaJ heat shock protein family (Hsp40) member B4	Q9UDY4
53	ENSG00000198342	***ZNF442***	zinc finger protein 442	Q9H7R0
54	ENSG00000196139	***AKR1C3***	aldo-keto reductase family 1 member C3	P42330
55	ENSG00000104980	***TIMM44***	translocase of inner mitochondrial membrane 44	O43615
56	ENSG00000100439	***ABHD4***	abhydrolase domain containing 4, N-acyl phospholipase B	Q8TB40
57	ENSG00000113758	***DBN1***	drebrin 1	Q16643
58	ENSG00000065911	***MTHFD2***	methylenetetrahydrofolate dehydrogenase (NADP+ dependent) 2, methenyltetrahydrofolate cyclohydrolase	P13995
59	ENSG00000068323	***TFE3***	transcription factor binding to IGHM enhancer 3	P19532
60	ENSG00000120254	***MTHFD1L***	methylenetetrahydrofolate dehydrogenase (NADP+ dependent) 1 like	Q6UB35
61	ENSG00000108179	***PPIF***	peptidylprolyl isomerase F	P30405
62	ENSG00000177156	***TALDO1***	transaldolase 1	P37837
63	ENSG00000040531	***CTNS***	cystinosin, lysosomal cystine transporter	O60931
64	ENSG00000171606	***ZNF274***	zinc finger protein 274	Q96GC6
65	ENSG00000081181	***ARG2***	arginase 2	P78540

66	ENSG00000133056	***PIK3C2B***	phosphatidylinositol-4-phosphate 3-kinase catalytic subunit type 2 beta	O00750
67	ENSG00000087842	***PIR***	pirin	O00625
68	ENSG00000103245	***CIAO3***	cytosolic iron-sulfur assembly component 3	Q9H6Q4
69	ENSG00000160953	***PWWP3A***	PWWP domain containing 3A, DNA repair factor	Q2TAK8
70	ENSG00000132478	***UNK***	unk zinc finger	Q9C0B0
71	ENSG00000132846	***ZBED3***	zinc finger BED-type containing 3	Q96IU2
72	ENSG00000109854	***HTATIP2***	HIV-1 Tat interactive protein 2	Q9BUP3
73	ENSG00000134684	***YARS1***	tyrosyl-tRNA synthetase 1	P54577
74	ENSG00000105327	***BBC3***	BCL2 binding component 3	Q9BXH1
75	ENSG00000177169	***ULK1***	unc-51 like autophagy activating kinase 1	O75385
76	ENSG00000217128	***FNIP1***	folliculin interacting protein 1	Q8TF40
77	ENSG00000162298	***SYVN1***	synoviolin 1	Q86TM6
78	ENSG00000183010	***PYCR1***	pyrroline-5-carboxylate reductase 1	P32322
79	ENSG00000130766	***SESN2***	sestrin 2	P58004
80	ENSG00000166123	***GPT2***	glutamic--pyruvic transaminase 2	Q8TD30
81	ENSG00000197044	***ZNF441***	zinc finger protein 441	Q8N8Z8
82	ENSG00000155849	***ELMO1***	engulfment and cell motility 1	Q92556
83	ENSG00000154040	***CABYR***	calcium binding tyrosine phosphorylation regulated	O75952
84	ENSG00000113504	***SLC12A7***	solute carrier family 12 member 7	Q9Y666
85	ENSG00000128165	***ADM2***	adrenomedullin 2	Q7Z4H4
86	ENSG00000176463	***SLCO3A1***	solute carrier organic anion transporter family member 3A1	Q9UIG8
87	ENSG00000141101	***NOB1***	NIN1 (RPN12) binding protein 1 homolog	Q9ULX3
88	ENSG00000104687	***GSR***	glutathione-disulfide reductase	P00390
89	ENSG00000197124	***ZNF682***	zinc finger protein 682	O95780
90	ENSG00000169016	***E2F6***	E2F transcription factor 6	O75461
91	ENSG00000168566	***SNRNP48***	small nuclear ribonucleoprotein U11/U12 subunit 48	Q6IEG0
92	ENSG00000115657	***ABCB6***	ATP binding cassette subfamily B member 6 (Langereis blood group)	Q9NP58
93	ENSG00000123360	***PDE1B***	phosphodiesterase 1B	Q01064
94	ENSG00000115902	***SLC1A4***	solute carrier family 1 member 4	P43007
95	ENSG00000167535	***CACNB3***	calcium voltage-gated channel auxiliary subunit beta 3	P54284
96	ENSG00000114573	***ATP6V1A***	ATPase H+ transporting V1 subunit A	P38606
97	ENSG00000095794	***CREM***	cAMP responsive element modulator	Q03060
98	ENSG00000164715	***LMTK2***	lemur tyrosine kinase 2	Q8IWU2
99	ENSG00000198722	***UNC13B***	unc-13 homolog B	O14795
100	ENSG00000274070	***CASTOR2***	cytosolic arginine sensor for mTORC1 subunit 2	A6NHX0

101	ENSG00000102032	***RENBP***	renin binding protein	P51606
102	ENSG00000165434	***PGM2L1***	phosphoglucomutase 2 like 1	Q6PCE3
103	ENSG00000117450	***PRDX1***	peroxiredoxin 1	Q06830
104	ENSG00000138448	***ITGAV***	integrin subunit alpha V	P06756
105	ENSG00000204386	***NEU1***	neuraminidase 1	Q99519
106	ENSG00000101574	***METTL4***	methyltransferase 4, N6-adenosine	Q8N3J2
107	ENSG00000196867	***ZFP28***	ZFP28 zinc finger protein	Q8NHY6
108	ENSG00000119326	***CTNNAL1***	catenin alpha like 1	Q9UBT7
109	ENSG00000175197	***DDIT3***	DNA damage inducible transcript 3	P35638
110	ENSG00000162377	***COA7***	cytochrome c oxidase assembly factor 7	Q96BR5
111	ENSG00000068903	***SIRT2***	sirtuin 2	Q8IXJ6
112	ENSG00000079819	***EPB41L2***	erythrocyte membrane protein band 4.1 like 2	O43491
113	ENSG00000103404	***USP31***	ubiquitin specific peptidase 31	Q70CQ4
114	ENSG00000166783	***MARF1***	meiosis regulator and mRNA stability factor 1	Q9Y4F3
115	ENSG00000161202	***DVL3***	dishevelled segment polarity protein 3	Q92997
116	ENSG00000242802	***AP5Z1***	adaptor related protein complex 5 subunit zeta 1	O43299
117	ENSG00000131584	***ACAP3***	ArfGAP with coiled-coil, ankyrin repeat and PH domains 3	Q96P50
118	ENSG00000198355	***PIM3***	Pim-3 proto-oncogene, serine/threonine kinase	Q86V86
119	ENSG00000059377	***TBXAS1***	thromboxane A synthase 1	P24557
120	ENSG00000089159	***PXN***	paxillin	P49023
121	ENSG00000169957	***ZNF768***	zinc finger protein 768	Q9H5H4
122	ENSG00000123836	***PFKFB2***	6-phosphofructo-2-kinase/fructose-2,6-biphosphatase 2	O60825
123	ENSG00000198369	***SPRED2***	sprouty related EVH1 domain containing 2	Q7Z698
124	ENSG00000005486	***RHBDD2***	rhomboid domain containing 2	Q6NTF9
125	ENSG00000141504	***SAT2***	spermidine/spermine N1-acetyltransferase family member 2	Q96F10
126	ENSG00000023909	***GCLM***	glutamate-cysteine ligase modifier subunit	P48507
127	ENSG00000187961	***KLHL17***	kelch like family member 17	Q6TDP4
128	ENSG00000177380	***PPFIA3***	PTPRF interacting protein alpha 3	O75145
129	ENSG00000160211	***G6PD***	glucose-6-phosphate dehydrogenase	P11413
130	ENSG00000103042	***SLC38A7***	solute carrier family 38 member 7	Q9NVC3
131	ENSG00000111912	***NCOA7***	nuclear receptor coactivator 7	Q8NI08
132	ENSG00000011021	***CLCN6***	chloride voltage-gated channel 6	P51797
133	ENSG00000154803	***FLCN***	folliculin	Q8NFG4
134	ENSG00000142657	***PGD***	phosphogluconate dehydrogenase	P52209
135	ENSG00000196821	***ILRUN***	inflammation and lipid regulator with UBA-like and NBR1-like domains	Q9H6K1

136	ENSG00000162231	***NXF1***	nuclear RNA export factor 1	Q9UBU9
137	ENSG00000143819	***EPHX1***	epoxide hydrolase 1	P07099
138	ENSG00000139618	***BRCA2***	BRCA2 DNA repair associated	P51587
139	ENSG00000180881	***CAPS2***	calcyphosine 2	Q9BXY5
140	ENSG00000005007	***UPF1***	UPF1 RNA helicase and ATPase	Q92900
141	ENSG00000123374	***CDK2***	cyclin dependent kinase 2	P24941
142	ENSG00000079999	***KEAP1***	kelch like ECH associated protein 1	Q14145
143	ENSG00000169504	***CLIC4***	chloride intracellular channel 4	Q9Y696
144	ENSG00000164916	***FOXK1***	forkhead box K1	P85037
145	ENSG00000085719	***CPNE3***	copine 3	O75131
146	ENSG00000010404	***IDS***	iduronate 2-sulfatase	P22304
147	ENSG00000092820	***EZR***	ezrin	P15311
148	ENSG00000068976	***PYGM***	glycogen phosphorylase, muscle associated	P11217
149	ENSG00000109756	***RAPGEF2***	Rap guanine nucleotide exchange factor 2	Q9Y4G8
150	ENSG00000164050	***PLXNB1***	plexin B1	O43157
151	ENSG00000169962	***TAS1R3***	taste 1 receptor member 3	Q7RTX0
152	ENSG00000205581	***HMGN1***	high mobility group nucleosome binding domain 1	P05114
153	ENSG00000135637	***CCDC142***	coiled-coil domain containing 142	Q17RM4
154	ENSG00000109814	***UGDH***	UDP-glucose 6-dehydrogenase	O60701
155	ENSG00000120733	***KDM3B***	lysine demethylase 3B	Q7LBC6
156	ENSG00000164663	***USP49***	ubiquitin specific peptidase 49	Q70CQ1
157	ENSG00000102100	***SLC35A2***	solute carrier family 35 member A2	P78381
158	ENSG00000180198	***RCC1***	regulator of chromosome condensation 1	P18754
159	ENSG00000113716	***HMGXB3***	HMG-box containing 3	Q12766
160	ENSG00000204256	***BRD2***	bromodomain containing 2	P25440
161	ENSG00000121101	***TEX14***	testis expressed 14, intercellular bridge forming factor	Q8IWB6
162	ENSG00000264006	***AKR1C8P***	aldo-keto reductase family 1 member C8	Q5T2L2
163	ENSG00000162066	***AMDHD2***	amidohydrolase domain containing 2	Q9Y303
164	ENSG00000166289	***PLEKHF1***	pleckstrin homology and FYVE domain containing 1	Q96S99
165	ENSG00000182095	***TNRC18***	trinucleotide repeat containing 18	O15417
166	ENSG00000102316	***MAGED2***	MAGE family member D2	Q9UNF1
167	ENSG00000205710	***C17orf107***	chromosome 17 open reading frame 107	Q6ZR85
168	ENSG00000160972	***PPP1R16A***	protein phosphatase 1 regulatory subunit 16A	Q96I34
169	ENSG00000196182	***STK40***	serine/threonine kinase 40	Q8N2I9
170	ENSG00000003393	***ALS2***	alsin Rho guanine nucleotide exchange factor ALS2	Q96Q42

171	ENSG00000108828	***VAT1***	vesicle amine transport 1	Q99536
172	ENSG00000089157	***RPLP0***	ribosomal protein lateral stalk subunit P0	P05388
173	ENSG00000112290	***WASF1***	WASP family member 1	Q92558
174	ENSG00000197147	***LRRC8B***	leucine rich repeat containing 8 VRAC subunit B	Q6P9F7
175	ENSG00000128272	***ATF4***	activating transcription factor 4	P18848
176	ENSG00000124789	***NUP153***	nucleoporin 153	P49790
177	ENSG00000086015	***MAST2***	microtubule associated serine/threonine kinase 2	Q6P0Q8
178	ENSG00000111602	***TIMELESS***	timeless circadian regulator	Q9UNS1
179	ENSG00000174885	***NLRP6***	NLR family pyrin domain containing 6	P59044
180	ENSG00000101544	***ADNP2***	ADNP homeobox 2	Q6IQ32
181	ENSG00000186432	***KPNA4***	karyopherin subunit alpha 4	O00629
182	ENSG00000110841	***PPFIBP1***	PPFIA binding protein 1	Q86W92
183	ENSG00000108932	***SLC16A6***	solute carrier family 16 member 6	O15403
184	ENSG00000197081	***IGF2R***	insulin like growth factor 2 receptor	P11717
185	ENSG00000134265	***NAPG***	NSF attachment protein gamma	Q99747
186	ENSG00000274750	***H3C6***	H3 clustered histone 6	P68431
187	ENSG00000141458	***NPC1***	NPC intracellular cholesterol transporter 1	O15118
188	ENSG00000181472	***ZBTB2***	zinc finger and BTB domain containing 2	Q8N680
189	ENSG00000104375	***STK3***	serine/threonine kinase 3	Q13188
190	ENSG00000165280	***VCP***	valosin containing protein	P55072
191	ENSG00000131471	***AOC3***	amine oxidase copper containing 3	Q16853
192	ENSG00000087074	***PPP1R15A***	protein phosphatase 1 regulatory subunit 15A	O75807
193	ENSG00000084207	***GSTP1***	glutathione S-transferase pi 1	P09211
194	ENSG00000065491	***TBC1D22B***	TBC1 domain family member 22B	Q9NU19
195	ENSG00000164171	***ITGA2***	integrin subunit alpha 2	P17301
196	ENSG00000031698	***SARS1***	seryl-tRNA synthetase 1	P49591
197	ENSG00000137876	***RSL24D1***	ribosomal L24 domain containing 1	Q9UHA3
198	ENSG00000114554	***PLXNA1***	plexin A1	Q9UIW2
199	ENSG00000125633	***CCDC93***	coiled-coil domain containing 93	Q567U6
200	ENSG00000006704	***GTF2IRD1***	GTF2I repeat domain containing 1	Q9UHL9
201	ENSG00000134030	***CTIF***	cap binding complex dependent translation initiation factor	O43310
202	ENSG00000030582	***GRN***	granulin precursor	P28799
203	ENSG00000087088	***BAX***	BCL2 associated X, apoptosis regulator	Q07812
204	ENSG00000101849	***TBL1X***	transducin beta like 1 X-linked	O60907
205	ENSG00000139926	***FRMD6***	FERM domain containing 6	Q96NE9

206	ENSG00000100889	***PCK2***	phosphoenolpyruvate carboxykinase 2, mitochondrial	Q16822
207	ENSG00000101773	***RBBP8***	RB binding protein 8, endonuclease	Q99708
208	ENSG00000012171	***SEMA3B***	semaphorin 3B	Q13214
209	ENSG00000112473	***SLC39A7***	solute carrier family 39 member 7	Q92504
210	ENSG00000124422	***USP22***	ubiquitin specific peptidase 22	Q9UPT9
211	ENSG00000159082	***SYNJ1***	synaptojanin 1	O43426
212	ENSG00000099290	***WASHC2A***	WASH complex subunit 2A	Q641Q2
213	ENSG00000197381	***ADARB1***	adenosine deaminase RNA specific B1	P78563
214	ENSG00000152620	***NADK2***	NAD kinase 2, mitochondrial	Q4G0N4
215	ENSG00000164970	***FAM219A***	family with sequence similarity 219 member A	Q8IW50
216	ENSG00000111328	***CDK2AP1***	cyclin dependent kinase 2 associated protein 1	O14519
217	ENSG00000100380	***ST13***	ST13 Hsp70 interacting protein	P50502
218	ENSG00000157796	***WDR19***	WD repeat domain 19	Q8NEZ3
219	ENSG00000122862	***SRGN***	serglycin	P10124
220	ENSG00000151012	***SLC7A11***	solute carrier family 7 member 11	Q9UPY5
221	ENSG00000172661	***WASHC2C***	WASH complex subunit 2C	Q9Y4E1
222	ENSG00000188566	***NDOR1***	NADPH dependent diflavin oxidoreductase 1	Q9UHB4
223	ENSG00000167555	***ZNF528***	zinc finger protein 528	Q3MIS6
224	ENSG00000100485	***SOS2***	SOS Ras/Rho guanine nucleotide exchange factor 2	Q07890
225	ENSG00000116717	***GADD45A***	growth arrest and DNA damage inducible alpha	P24522
226	ENSG00000182199	***SHMT2***	serine hydroxymethyltransferase 2	P34897
227	ENSG00000158615	***PPP1R15B***	protein phosphatase 1 regulatory subunit 15B	Q5SWA1
228	ENSG00000160447	***PKN3***	protein kinase N3	Q6P5Z2
229	ENSG00000184445	***KNTC1***	kinetochore associated 1	P50748
230	ENSG00000118960	***HS1BP3***	HCLS1 binding protein 3	Q53T59
231	ENSG00000076201	***PTPN23***	protein tyrosine phosphatase non-receptor type 23	Q9H3S7
232	ENSG00000110046	***ATG2A***	autophagy related 2A	Q2TAZ0
233	ENSG00000183091	***NEB***	nebulin	P20929
234	ENSG00000168397	***ATG4B***	autophagy related 4B cysteine peptidase	Q9Y4P1
235	ENSG00000149308	***NPAT***	nuclear protein, coactivator of histone transcription	Q14207
236	ENSG00000164068	***RNF123***	ring finger protein 123	Q5XPI4
237	ENSG00000171786	***NHLH1***	nescient helix-loop-helix 1	Q02575
238	ENSG00000143493	***INTS7***	integrator complex subunit 7	Q9NVH2
239	ENSG00000067225	***PKM***	pyruvate kinase M1/2	P14618
240	ENSG00000167110	***GOLGA2***	golgin A2	Q08379

241	ENSG00000196498	***NCOR2***	nuclear receptor corepressor 2	Q9Y618
242	ENSG00000112182	***BACH2***	BTB domain and CNC homolog 2	Q9BYV9
243	ENSG00000150961	***SEC24D***	SEC24 homolog D, COPII coat complex component	O94855
244	ENSG00000154589	***LY96***	lymphocyte antigen 96	Q9Y6Y9
245	ENSG00000267041	***ZNF850***	zinc finger protein 850	A8MQ14
246	ENSG00000106012	***IQCE***	IQ motif containing E	Q6IPM2
247	ENSG00000158545	***ZC3H18***	zinc finger CCCH-type containing 18	Q86VM9
248	ENSG00000143367	***TUFT1***	tuftelin 1	Q9NNX1
249	ENSG00000077721	***UBE2A***	ubiquitin conjugating enzyme E2 A	P49459
250	ENSG00000108344	***PSMD3***	proteasome 26S subunit, non-ATPase 3	O43242
251	ENSG00000143630	***HCN3***	hyperpolarization activated cyclic nucleotide gated potassium channel 3	Q9P1Z3
252	ENSG00000173530	***TNFRSF10D***	TNF receptor superfamily member 10d	Q9UBN6
253	ENSG00000154832	***CXXC1***	CXXC finger protein 1	Q9P0U4
254	ENSG00000131389	***SLC6A6***	solute carrier family 6 member 6	P31641
255	ENSG00000132024	***CC2D1A***	coiled-coil and C2 domain containing 1A	Q6P1N0
256	ENSG00000144749	***LRIG1***	leucine rich repeats and immunoglobulin like domains 1	Q96JA1
257	ENSG00000165233	***CARD19***	caspase recruitment domain family member 19	Q96LW7
258	ENSG00000110721	***CHKA***	choline kinase alpha	P35790
259	ENSG00000134910	***STT3A***	STT3 oligosaccharyltransferase complex catalytic subunit A	P46977
260	ENSG00000023171	***GRAMD1B***	GRAM domain containing 1B	Q3KR37
261	ENSG00000198408	***OGA***	O-GlcNAcase	O60502
262	ENSG00000160117	***ANKLE1***	ankyrin repeat and LEM domain containing 1	Q8NAG6
263	ENSG00000174106	***LEMD3***	LEM domain containing 3	Q9Y2U8
264	ENSG00000100852	***ARHGAP5***	Rho GTPase activating protein 5	Q13017
265	ENSG00000157881	***PANK4***	pantothenate kinase 4 (inactive)	Q9NVE7
266	ENSG00000168672	***LRATD2***	LRAT domain containing 2	Q96KN1
267	ENSG00000131653	***TRAF7***	TNF receptor associated factor 7	Q6Q0C0
268	ENSG00000196923	***PDLIM7***	PDZ and LIM domain 7	Q9NR12
269	ENSG00000135677	***GNS***	glucosamine (N-acetyl)-6-sulfatase	P15586
270	ENSG00000037042	***TUBG2***	tubulin gamma 2	Q9NRH3
271	ENSG00000104731	***KLHDC4***	kelch domain containing 4	Q8TBB5
272	ENSG00000105939	***ZC3HAV1***	zinc finger CCCH-type containing, antiviral 1	Q7Z2W4
273	ENSG00000113742	***CPEB4***	cytoplasmic polyadenylation element binding protein 4	Q17RY0
274	ENSG00000064419	***TNPO3***	transportin 3	Q9Y5L0
275	ENSG00000134440	***NARS1***	asparaginyl-tRNA synthetase 1	O43776

276	ENSG00000149115	***TNKS1BP1***	tankyrase 1 binding protein 1	Q9C0C2
277	ENSG00000177084	***POLE***	DNA polymerase epsilon, catalytic subunit	Q07864
278	ENSG00000186951	***PPARA***	peroxisome proliferator activated receptor alpha	Q07869
279	ENSG00000168924	***LETM1***	leucine zipper and EF-hand containing transmembrane protein 1	O95202
280	ENSG00000130643	***CALY***	calcyon neuron specific vesicular protein	Q9NYX4
281	ENSG00000179604	***CDC42EP4***	CDC42 effector protein 4	Q9H3Q1
282	ENSG00000198380	***GFPT1***	glutamine--fructose-6-phosphate transaminase 1	Q06210
283	ENSG00000163600	***ICOS***	inducible T cell costimulator	Q9Y6W8
284	ENSG00000173276	***ZBTB21***	zinc finger and BTB domain containing 21	Q9ULJ3
285	ENSG00000171121	***KCNMB3***	potassium calcium-activated channel subfamily M regulatory beta subunit 3	Q9NPA1
286	ENSG00000124782	***RREB1***	ras responsive element binding protein 1	Q92766
287	ENSG00000119402	***FBXW2***	F-box and WD repeat domain containing 2	Q9UKT8
288	ENSG00000060749	***QSER1***	glutamine and serine rich 1	Q2KHR3
289	ENSG00000085644	***ZNF213***	zinc finger protein 213	O14771
290	ENSG00000175606	***TMEM70***	transmembrane protein 70	Q9BUB7
291	ENSG00000168071	***CCDC88B***	coiled-coil domain containing 88B	A6NC98
292	ENSG00000214655	***ZSWIM8***	zinc finger SWIM-type containing 8	A7E2V4
293	ENSG00000256087	***ZNF432***	zinc finger protein 432	O94892
294	ENSG00000123213	***NLN***	neurolysin	Q9BYT8
295	ENSG00000125089	***SH3TC1***	SH3 domain and tetratricopeptide repeats 1	Q8TE82
296	ENSG00000204839	***MROH6***	maestro heat like repeat family member 6	A6NGR9
297	ENSG00000124788	***ATXN1***	ataxin 1	P54253
298	ENSG00000265491	***RNF115***	ring finger protein 115	Q9Y4L5
299	ENSG00000144485	***HES6***	hes family bHLH transcription factor 6	Q96HZ4
300	ENSG00000176095	***IP6K1***	inositol hexakisphosphate kinase 1	Q92551
301	ENSG00000033100	***CHPF2***	chondroitin polymerizing factor 2	Q9P2E5
302	ENSG00000061676	***NCKAP1***	NCK associated protein 1	Q9Y2A7
303	ENSG00000020256	***ZFP64***	ZFP64 zinc finger protein	Q9NTW7
304	ENSG00000185896	***LAMP1***	lysosomal associated membrane protein 1	P11279
305	ENSG00000069849	***ATP1B3***	ATPase Na+/K+ transporting subunit beta 3	P54709
306	ENSG00000185133	***INPP5J***	inositol polyphosphate-5-phosphatase J	Q15735
307	ENSG00000115295	***CLIP4***	CAP-Gly domain containing linker protein family member 4	Q8N3C7
308	ENSG00000106266	***SNX8***	sorting nexin 8	Q9Y5X2
309	ENSG00000108349	***CASC3***	CASC3 exon junction complex subunit	O15234
310	ENSG00000125733	***TRIP10***	thyroid hormone receptor interactor 10	Q15642

311	ENSG00000150756	***ATPSCKMT***	ATP synthase c subunit lysine N-methyltransferase	Q6P4H8
312	ENSG00000213988	***ZNF90***	zinc finger protein 90	Q03938
313	ENSG00000198720	***ANKRD13B***	ankyrin repeat domain 13B	Q86YJ7
314	ENSG00000168488	***ATXN2L***	ataxin 2 like	Q8WWM7
315	ENSG00000116254	***CHD5***	chromodomain helicase DNA binding protein 5	Q8TDI0
316	ENSG00000177302	***TOP3A***	DNA topoisomerase III alpha	Q13472
317	ENSG00000145214	***DGKQ***	diacylglycerol kinase theta	P52824
318	ENSG00000025770	***NCAPH2***	non-SMC condensin II complex subunit H2	Q6IBW4
319	ENSG00000164463	***CREBRF***	CREB3 regulatory factor	Q8IUR6
320	ENSG00000100461	***RBM23***	RNA binding motif protein 23	Q86U06
321	ENSG00000162694	***EXTL2***	exostosin like glycosyltransferase 2	Q9UBQ6
322	ENSG00000177628	***GBA***	glucosylceramidase beta 1	P04062
323	ENSG00000085063	***CD59***	CD59 molecule (CD59 blood group)	P13987
324	ENSG00000166886	***NAB2***	NGFI-A binding protein 2	Q15742
325	ENSG00000086758	***HUWE1***	HECT, UBA and WWE domain containing E3 ubiquitin protein ligase 1	Q7Z6Z7
326	ENSG00000164104	***HMGB2***	high mobility group box 2	P26583
327	ENSG00000139718	***SETD1B***	SET domain containing 1B, histone lysine methyltransferase	Q9UPS6
328	ENSG00000106397	***PLOD3***	procollagen-lysine,2-oxoglutarate 5-dioxygenase 3	O60568
329	ENSG00000102393	***GLA***	galactosidase alpha	P06280
330	ENSG00000160867	***FGFR4***	fibroblast growth factor receptor 4	P22455
331	ENSG00000123066	***MED13L***	mediator complex subunit 13L	Q71F56
332	ENSG00000155506	***LARP1***	La ribonucleoprotein 1, translational regulator	Q6PKG0
333	ENSG00000104853	***CLPTM1***	CLPTM1 regulator of GABA type A receptor forward trafficking	O96005
334	ENSG00000163293	***NIPAL1***	NIPA like domain containing 1	Q6NVV3
335	ENSG00000080845	***DLGAP4***	DLG associated protein 4	Q9Y2H0
336	ENSG00000167315	***ACAA2***	acetyl-CoA acyltransferase 2	P42765
337	ENSG00000115421	***PAPOLG***	poly(A) polymerase gamma	Q9BWT3
338	ENSG00000264522	***OTUD7B***	OTU deubiquitinase 7B	Q6GQQ9
339	ENSG00000145740	***SLC30A5***	solute carrier family 30 member 5	Q8TAD4
340	ENSG00000090674	***MCOLN1***	mucolipin TRP cation channel 1	Q9GZU1
341	ENSG00000127616	***SMARCA4***	SWI/SNF related, matrix associated, actin dependent regulator of chromatin, subfamily a, member 4	P51532
342	ENSG00000100350	***FOXRED2***	FAD dependent oxidoreductase domain containing 2	Q8IWF2
343	ENSG00000111640	***GAPDH***	glyceraldehyde-3-phosphate dehydrogenase	P04406
344	ENSG00000184575	***XPOT***	exportin for tRNA	O43592

345	ENSG00000119041	***GTF3C3***	general transcription factor IIIC subunit 3	Q9Y5Q9
346	ENSG00000100644	***HIF1A***	hypoxia inducible factor 1 subunit alpha	Q16665
347	ENSG00000105726	***ATP13A1***	ATPase 13A1	Q9HD20
348	ENSG00000127481	***UBR4***	ubiquitin protein ligase E3 component n-recognin 4	Q5T4S7
349	ENSG00000165476	***REEP3***	receptor accessory protein 3	Q6NUK4
350	ENSG00000011275	***RNF216***	ring finger protein 216	Q9NWF9
351	ENSG00000126003	***PLAGL2***	PLAG1 like zinc finger 2	Q9UPG8
352	ENSG00000163141	***BNIPL***	BCL2 interacting protein like	Q7Z465
353	ENSG00000173064	***HECTD4***	HECT domain E3 ubiquitin protein ligase 4	Q9Y4D8
354	ENSG00000008710	***PKD1***	polycystin 1, transient receptor potential channel interacting	P98161
355	ENSG00000172673	***THEMIS***	thymocyte selection associated	Q8N1K5
356	ENSG00000130706	***ADRM1***	ADRM1 26S proteasome ubiquitin receptor	Q16186
357	ENSG00000163155	***LYSMD1***	LysM domain containing 1	Q96S90
358	ENSG00000148384	***INPP5E***	inositol polyphosphate-5-phosphatase E	Q9NRR6
359	ENSG00000077463	***SIRT6***	sirtuin 6	Q8N6T7
360	ENSG00000120694	***HSPH1***	heat shock protein family H (Hsp110) member 1	Q92598
361	ENSG00000184154	***LRRC51***	leucine rich repeat containing 51	Q96E66
362	ENSG00000116017	***ARID3A***	AT-rich interaction domain 3A	Q99856
363	ENSG00000130382	***MLLT1***	MLLT1 super elongation complex subunit	Q03111
364	ENSG00000205726	***ITSN1***	intersectin 1	Q15811
365	ENSG00000106404	***CLDN15***	claudin 15	P56746
366	ENSG00000139613	***SMARCC2***	SWI/SNF related, matrix associated, actin dependent regulator of chromatin subfamily c member 2	Q8TAQ2
367	ENSG00000004478	***FKBP4***	FKBP prolyl isomerase 4	Q02790
368	ENSG00000233822	***H2BC15***	H2B clustered histone 15	Q99877
369	ENSG00000143569	***UBAP2L***	ubiquitin associated protein 2 like	Q14157
370	ENSG00000197136	***PCNX3***	pecanex 3	Q9H6A9
371	ENSG00000223547	***ZNF844***	zinc finger protein 844	Q08AG5
372	ENSG00000008256	***CYTH3***	cytohesin 3	O43739
373	ENSG00000112305	***SMAP1***	small ArfGAP 1	Q8IYB5
374	ENSG00000143368	***SF3B4***	splicing factor 3b subunit 4	Q15427
375	ENSG00000173153	***ESRRA***	estrogen related receptor alpha	P11474
376	ENSG00000162923	***WDR26***	WD repeat domain 26	Q9H7D7
377	ENSG00000118496	***FBXO30***	F-box protein 30	Q8TB52
378	ENSG00000159147	***DONSON***	DNA replication fork stabilization factor DONSON	Q9NYP3
379	ENSG00000130545	***CRB3***	crumbs cell polarity complex component 3	Q9BUF7

380	ENSG00000138646	***HERC5***	HECT and RLD domain containing E3 ubiquitin protein ligase 5	Q9UII4
381	ENSG00000065526	***SPEN***	spen family transcriptional repressor	Q96T58
382	ENSG00000140854	***KATNB1***	katanin regulatory subunit B1	Q9BVA0
383	ENSG00000113916	***BCL6***	BCL6 transcription repressor	P41182
384	ENSG00000146859	***TMEM140***	transmembrane protein 140	Q9NV12
385	ENSG00000162702	***ZNF281***	zinc finger protein 281	Q9Y2X9
386	ENSG00000130726	***TRIM28***	tripartite motif containing 28	Q13263
387	ENSG00000132613	***MTSS2***	MTSS I-BAR domain containing 2	Q765P7
388	ENSG00000196365	***LONP1***	lon peptidase 1, mitochondrial	P36776
389	ENSG00000158828	***PINK1***	PTEN induced kinase 1	Q9BXM7
390	ENSG00000105835	***NAMPT***	nicotinamide phosphoribosyltransferase	P43490
391	ENSG00000104897	***SF3A2***	splicing factor 3a subunit 2	Q15428
392	ENSG00000100918	***REC8***	REC8 meiotic recombination protein	O95072
393	ENSG00000116793	***PHTF1***	putative homeodomain transcription factor 1	Q9UMS5
394	ENSG00000204262	***COL5A2***	collagen type V alpha 2 chain	P05997
395	ENSG00000162885	***B3GALNT2***	beta-1,3-N-acetylgalactosaminyltransferase 2	Q8NCR0
396	ENSG00000102057	***KCND1***	potassium voltage-gated channel subfamily D member 1	Q9NSA2
397	ENSG00000204569	***PPP1R10***	protein phosphatase 1 regulatory subunit 10	Q96QC0
398	ENSG00000176994	***SMCR8***	SMCR8-C9orf72 complex subunit	Q8TEV9
399	ENSG00000123136	***DDX39A***	DExD-box helicase 39A	O00148
400	ENSG00000179134	***SAMD4B***	sterile alpha motif domain containing 4B	Q5PRF9
401	ENSG00000156599	***ZDHHC5***	zinc finger DHHC-type palmitoyltransferase 5	Q9C0B5
402	ENSG00000102996	***MMP15***	matrix metallopeptidase 15	P51511
403	ENSG00000072364	***AFF4***	ALF transcription elongation factor 4	Q9UHB7
404	ENSG00000121064	***SCPEP1***	serine carboxypeptidase 1	Q9HB40
405	ENSG00000198198	***SZT2***	SZT2 subunit of KICSTOR complex	Q5T011
406	ENSG00000158406	***H4C8***	H4 clustered histone 8	P62805
407	ENSG00000115568	***ZNF142***	zinc finger protein 142	P52746
408	ENSG00000142528	***ZNF473***	zinc finger protein 473	Q8WTR7
409	ENSG00000136720	***HS6ST1***	heparan sulfate 6-O-sulfotransferase 1	O60243
410	ENSG00000166233	***ARIH1***	ariadne RBR E3 ubiquitin protein ligase 1	Q9Y4X5
411	ENSG00000152684	***PELO***	pelota mRNA surveillance and ribosome rescue factor	Q9BRX2
412	ENSG00000165501	***LRR1***	leucine rich repeat protein 1	Q96L50
413	ENSG00000124209	***RAB22A***	RAB22A, member RAS oncogene family	Q9UL26
414	ENSG00000187801	***ZFP69B***	ZFP69 zinc finger protein B	Q9UJL9

415	ENSG00000070495	***JMJD6***	jumonji domain containing 6, arginine demethylase and lysine hydroxylase	Q6NYC1
416	ENSG00000141424	***SLC39A6***	solute carrier family 39 member 6	Q13433
417	ENSG00000068400	***GRIPAP1***	GRIP1 associated protein 1	Q4V328
418	ENSG00000160796	***NBEAL2***	neurobeachin like 2	Q6ZNJ1
419	ENSG00000185163	***DDX51***	DEAD-box helicase 51	Q8N8A6
420	ENSG00000174996	***KLC2***	kinesin light chain 2	Q9H0B6
421	ENSG00000146733	***PSPH***	phosphoserine phosphatase	P78330
422	ENSG00000142686	***C1orf216***	chromosome 1 open reading frame 216	Q8TAB5
423	ENSG00000185347	***TEDC1***	tubulin epsilon and delta complex 1	Q86SX3
424	ENSG00000127837	***AAMP***	angio associated migratory cell protein	Q13685
425	ENSG00000186130	***ZBTB6***	zinc finger and BTB domain containing 6	Q15916
426	ENSG00000185477	***GPRIN3***	GPRIN family member 3	Q6ZVF9
427	ENSG00000125772	***GPCPD1***	glycerophosphocholine phosphodiesterase 1	Q9NPB8
428	ENSG00000189042	***ZNF567***	zinc finger protein 567	Q8N184
429	ENSG00000170881	***RNF139***	ring finger protein 139	Q8WU17
430	ENSG00000138735	***PDE5A***	phosphodiesterase 5A	O76074
431	ENSG00000125505	***MBOAT7***	membrane bound O-acyltransferase domain containing 7	Q96N66
432	ENSG00000112742	***TTK***	TTK protein kinase	P33981
433	ENSG00000143375	***CGN***	cingulin	Q9P2M7
434	ENSG00000137166	***FOXP4***	forkhead box P4	Q8IVH2
435	ENSG00000123358	***NR4A1***	nuclear receptor subfamily 4 group A member 1	P22736
436	ENSG00000152223	***EPG5***	ectopic P-granules 5 autophagy tethering factor	Q9HCE0
437	ENSG00000152939	***MARVELD2***	MARVEL domain containing 2	Q8N4S9
438	ENSG00000170871	***KIAA0232***	KIAA0232	Q92628
439	ENSG00000038358	***EDC4***	enhancer of mRNA decapping 4	Q6P2E9
440	ENSG00000171219	***CDC42BPG***	CDC42 binding protein kinase gamma	Q6DT37
441	ENSG00000102977	***ACD***	ACD shelterin complex subunit and telomerase recruitment factor	Q96AP0
442	ENSG00000141568	***FOXK2***	forkhead box K2	Q01167
443	ENSG00000065268	***WDR18***	WD repeat domain 18	Q9BV38
444	ENSG00000234444	***ZNF736***	zinc finger protein 736	B4DX44
445	ENSG00000173548	***SNX33***	sorting nexin 33	Q8WV41
446	ENSG00000186230	***ZNF749***	zinc finger protein 749	O43361
447	ENSG00000204524	***ZNF805***	zinc finger protein 805	Q5CZA5

Table 1b continued

Order	chr	start	end	baseMean	log2FoldChange	lfcSE	stat	pvalue	padj
1	16	69706996	69726668	346.693	2.457	0.218	11.248	2.37E-29	3.07E-25
2	5	91368631	91383317	3175.559	1.305	0.118	11.035	2.59E-28	1.68E-24
3	11	68754620	68844410	603.707	2.716	0.270	10.057	8.52E-24	3.68E-20
4	16	87830023	87869507	1346.098	1.219	0.133	9.145	5.98E-20	1.55E-16
5	2	10439968	10448327	1660.221	1.023	0.120	8.523	1.56E-17	3.37E-14
6	3	48856926	48898904	267.897	1.144	0.136	8.427	3.55E-17	6.57E-14
7	16	70251983	70289707	4381.327	0.697	0.084	8.341	7.34E-17	1.19E-13
8	9	78297125	78330093	1488.575	1.086	0.136	8.006	1.18E-15	1.53E-12
9	6	53497341	53616970	1258.380	0.872	0.109	7.994	1.31E-15	1.54E-12
10	17	1569268	1628886	169.252	2.053	0.273	7.517	5.60E-14	5.17E-11
11	20	63520765	63522206	1332.610	0.876	0.118	7.443	9.86E-14	8.50E-11
12	11	67317871	67398410	820.146	0.823	0.113	7.257	3.95E-13	3.01E-10
13	13	29509414	29595688	1604.807	0.522	0.073	7.189	6.51E-13	4.68E-10
14	16	56932142	56944864	1950.929	0.623	0.087	7.121	1.07E-12	6.59E-10
15	17	81918270	81927735	646.794	1.042	0.148	7.059	1.68E-12	9.53E-10
16	1	200969390	201023714	5536.202	0.782	0.111	7.058	1.69E-12	9.53E-10
17	22	35380361	35394214	118.300	4.091	0.583	7.021	2.20E-12	1.19E-09
18	1	26472440	26476642	4201.608	0.462	0.066	6.987	2.80E-12	1.45E-09
19	5	33440696	33468091	3883.418	0.621	0.090	6.907	4.95E-12	2.46E-09
20	1	6590724	6614607	1585.720	0.772	0.112	6.885	5.78E-12	2.77E-09
21	11	62856004	62888880	5743.672	1.397	0.204	6.843	7.76E-12	3.58E-09
22	17	7217125	7225266	3071.367	0.541	0.081	6.681	2.37E-11	9.91E-09
23	9	92210207	92293854	3525.848	0.446	0.067	6.618	3.64E-11	1.43E-08
24	15	40952962	40956512	266.899	1.461	0.221	6.603	4.02E-11	1.53E-08
25	2	61476032	61538741	4845.018	0.824	0.126	6.568	5.09E-11	1.88E-08
26	12	10212458	10223128	821.575	1.034	0.158	6.536	6.32E-11	2.27E-08
27	19	48965309	48966879	23835.068	0.800	0.123	6.521	6.97E-11	2.44E-08
28	12	46358188	46372773	5627.146	0.676	0.104	6.477	9.33E-11	3.10E-08
29	12	57475445	57517569	5359.986	0.444	0.069	6.467	1.00E-10	3.24E-08
30	21	34073578	34106260	1939.433	0.860	0.135	6.390	1.66E-10	4.93E-08
31	X	153947557	153971818	2301.842	0.625	0.098	6.370	1.89E-10	5.19E-08

32	6	43770184	43786487	237.099	1.254	0.199	6.315	2.70E-10	7.10E-08
33	3	5187646	5219958	5185.932	0.528	0.084	6.313	2.74E-10	7.10E-08
34	1	119648411	119744218	391.248	1.034	0.168	6.146	7.92E-10	1.86E-07
35	6	41189749	41201149	206.542	1.031	0.171	6.044	1.50E-09	3.24E-07
36	16	1444934	1475084	1872.914	0.599	0.099	6.018	1.76E-09	3.74E-07
37	19	46774883	46788594	1016.999	0.919	0.155	5.947	2.72E-09	5.42E-07
38	11	3000922	3057613	2360.196	0.466	0.078	5.940	2.86E-09	5.60E-07
39	16	23835983	24220611	4291.140	0.703	0.120	5.867	4.45E-09	8.34E-07
40	1	225401502	225428925	2632.433	0.468	0.080	5.858	4.68E-09	8.63E-07
41	1	43991500	44031467	185.179	1.339	0.231	5.789	7.06E-09	1.23E-06
42	4	83535914	83605875	159.535	1.029	0.178	5.779	7.50E-09	1.29E-06
43	12	57234903	57240715	60.173	2.005	0.350	5.733	9.85E-09	1.63E-06
44	4	38823715	38856817	103.130	1.132	0.198	5.718	1.08E-08	1.77E-06
45	19	33373685	33382686	852.214	0.530	0.093	5.694	1.24E-08	1.98E-06
46	6	149963943	149973715	34.668	2.007	0.353	5.686	1.30E-08	2.05E-06
47	7	996986	1138260	361.715	1.036	0.183	5.671	1.42E-08	2.18E-06
48	4	158671968	158710742	320.520	0.758	0.135	5.614	1.98E-08	2.84E-06
49	12	104215779	104350307	2448.960	1.545	0.277	5.589	2.29E-08	3.22E-06
50	7	30580533	30634033	3354.438	0.480	0.086	5.566	2.61E-08	3.59E-06
51	5	179806398	179838078	4740.390	0.618	0.113	5.482	4.20E-08	5.49E-06
52	1	77979175	78017964	133.851	1.210	0.222	5.462	4.72E-08	5.98E-06
53	19	12345944	12365905	52.008	1.626	0.299	5.446	5.15E-08	6.41E-06
54	10	5035354	5107686	247.399	0.819	0.151	5.441	5.31E-08	6.54E-06
55	19	7926718	7943667	815.919	0.618	0.114	5.420	5.94E-08	7.12E-06
56	14	22598290	22612963	392.203	0.616	0.115	5.350	8.81E-08	9.66E-06
57	5	177456608	177474401	115.996	1.140	0.215	5.305	1.12E-07	1.22E-05
58	2	74186172	74217565	914.925	0.631	0.119	5.300	1.16E-07	1.25E-05
59	X	49028726	49043410	1005.926	0.591	0.112	5.278	1.30E-07	1.36E-05
60	6	150865679	151101887	551.201	0.605	0.115	5.275	1.33E-07	1.37E-05
61	10	79347469	79355334	430.883	0.624	0.118	5.268	1.38E-07	1.40E-05
62	11	747415	765012	1811.116	0.586	0.111	5.263	1.42E-07	1.42E-05
63	17	3636459	3663103	432.373	0.663	0.126	5.259	1.45E-07	1.43E-05
64	19	58183029	58213562	1255.529	0.431	0.082	5.249	1.53E-07	1.50E-05
65	14	67619920	67651708	55.833	1.613	0.311	5.185	2.16E-07	2.08E-05
66	1	204422628	204494805	855.076	0.458	0.089	5.152	2.58E-07	2.42E-05

67	X	15384799	15493564	13.512	3.869	0.760	5.093	3.52E-07	3.08E-05
68	16	729760	741329	582.986	0.569	0.112	5.089	3.60E-07	3.10E-05
69	19	1354711	1378431	878.353	0.504	0.100	5.040	4.66E-07	3.94E-05
70	17	75784806	75825799	988.746	0.432	0.086	5.034	4.81E-07	4.01E-05
71	5	77072072	77087285	85.850	1.336	0.266	5.022	5.11E-07	4.21E-05
72	11	20363685	20383782	799.480	0.448	0.090	5.009	5.47E-07	4.46E-05
73	1	32775237	32817380	2954.093	0.376	0.075	5.004	5.63E-07	4.52E-05
74	19	47220822	47232766	502.730	0.725	0.145	5.000	5.72E-07	4.57E-05
75	12	131894622	131923150	946.960	0.562	0.114	4.941	7.78E-07	6.03E-05
76	5	131641714	131797017	540.565	0.608	0.123	4.924	8.50E-07	6.50E-05
77	11	65121780	65134532	1197.317	0.429	0.087	4.914	8.94E-07	6.81E-05
78	17	81932384	81942412	190.242	0.877	0.179	4.888	1.02E-06	7.58E-05
79	1	28259518	28282491	268.983	0.720	0.148	4.865	1.14E-06	8.31E-05
80	16	46884362	46931289	230.896	1.025	0.212	4.843	1.28E-06	9.11E-05
81	19	11767000	11784078	536.847	0.793	0.164	4.834	1.34E-06	9.41E-05
82	7	36852906	37449223	1453.494	0.478	0.099	4.805	1.55E-06	1.05E-04
83	18	24138987	24161600	73.032	1.771	0.369	4.803	1.57E-06	1.05E-04
84	5	1050384	1112063	1444.151	0.561	0.118	4.760	1.94E-06	1.24E-04
85	22	50481543	50486440	196.959	0.964	0.203	4.760	1.94E-06	1.24E-04
86	15	91853708	92172435	425.974	0.536	0.113	4.734	2.20E-06	1.39E-04
87	16	69741871	69754926	1043.531	0.444	0.095	4.675	2.94E-06	1.77E-04
88	8	30678066	30727846	1250.835	0.568	0.122	4.662	3.14E-06	1.86E-04
89	19	19997058	20039505	139.423	0.926	0.200	4.636	3.55E-06	2.05E-04
90	2	11444375	11466177	329.951	0.684	0.148	4.628	3.69E-06	2.12E-04
91	6	7590198	7611967	705.424	0.392	0.085	4.627	3.70E-06	2.12E-04
92	2	219209772	219218994	35.916	2.184	0.473	4.622	3.80E-06	2.15E-04
93	12	54549601	54579239	134.815	0.847	0.184	4.605	4.13E-06	2.32E-04
94	2	64988477	65023865	408.228	0.606	0.132	4.587	4.51E-06	2.50E-04
95	12	48813794	48828941	214.625	0.644	0.141	4.566	4.96E-06	2.71E-04
96	3	113747033	113812056	469.450	0.446	0.098	4.564	5.03E-06	2.73E-04
97	10	35126791	35212958	270.154	0.642	0.141	4.562	5.08E-06	2.75E-04
98	7	98106862	98209638	835.646	0.400	0.088	4.552	5.30E-06	2.85E-04
99	9	35161992	35405338	50.042	1.822	0.402	4.532	5.84E-06	3.07E-04
100	7	74964705	75031528	178.388	0.634	0.141	4.483	7.35E-06	3.73E-04
101	X	153935269	153944687	244.895	0.716	0.161	4.455	8.37E-06	4.15E-04

102	11	74330316	74398433	384.116	0.484	0.109	4.449	8.62E-06	4.24E-04
103	1	45510914	45542732	2765.481	0.411	0.093	4.439	9.03E-06	4.39E-04
104	2	186590056	186680901	202.881	0.598	0.135	4.428	9.51E-06	4.56E-04
105	6	31857659	31862905	502.828	0.580	0.131	4.428	9.52E-06	4.56E-04
106	18	2537525	2571509	650.555	0.455	0.103	4.419	9.93E-06	4.74E-04
107	19	56538948	56556808	333.664	0.532	0.121	4.382	1.17E-05	5.52E-04
108	9	108942569	109013522	127.376	1.092	0.249	4.378	1.20E-05	5.60E-04
109	12	57516588	57521737	667.286	0.577	0.132	4.374	1.22E-05	5.65E-04
110	1	52684449	52698347	170.393	0.737	0.169	4.361	1.30E-05	5.96E-04
111	19	38878555	38899862	978.472	0.481	0.111	4.351	1.36E-05	6.16E-04
112	6	130839347	131063322	373.700	0.589	0.135	4.349	1.37E-05	6.19E-04
113	16	23061406	23149452	134.858	0.807	0.186	4.344	1.40E-05	6.25E-04
114	16	15594387	15643154	3797.525	0.284	0.066	4.327	1.51E-05	6.61E-04
115	3	184155377	184173614	1230.885	0.334	0.077	4.308	1.64E-05	7.11E-04
116	7	4775615	4794397	1449.056	0.534	0.124	4.308	1.65E-05	7.12E-04
117	1	1292390	1309609	1024.820	0.442	0.103	4.302	1.69E-05	7.21E-04
118	22	49960768	49964072	723.432	0.479	0.113	4.240	2.24E-05	9.14E-04
119	7	139777051	140020325	191.045	0.765	0.181	4.230	2.34E-05	9.49E-04
120	12	120210439	120265771	4084.138	0.354	0.084	4.214	2.50E-05	1.01E-03
121	16	30524004	30526821	172.437	0.780	0.185	4.212	2.54E-05	1.02E-03
122	1	207034366	207081024	95.175	0.848	0.202	4.196	2.71E-05	1.08E-03
123	2	65310851	65432637	50.852	1.190	0.284	4.190	2.79E-05	1.10E-03
124	7	75842602	75888926	880.045	0.462	0.111	4.172	3.02E-05	1.17E-03
125	17	7626234	7627876	882.867	0.375	0.090	4.151	3.31E-05	1.26E-03
126	1	93885199	93909456	481.732	0.628	0.152	4.144	3.41E-05	1.29E-03
127	1	960584	965719	459.186	0.475	0.115	4.143	3.43E-05	1.29E-03
128	19	49119544	49151026	109.237	1.110	0.268	4.137	3.52E-05	1.32E-03
129	X	154531391	154547572	690.572	0.658	0.160	4.123	3.74E-05	1.38E-03
130	16	58665109	58684770	293.908	0.543	0.132	4.109	3.97E-05	1.46E-03
131	6	125781161	125932034	1171.682	0.366	0.089	4.104	4.06E-05	1.47E-03
132	1	11806096	11848079	579.504	0.522	0.127	4.104	4.06E-05	1.47E-03
133	17	17212212	17237188	1241.687	0.348	0.085	4.100	4.13E-05	1.49E-03
134	1	10398592	10420511	1045.689	0.458	0.112	4.074	4.63E-05	1.65E-03
135	6	34587288	34696859	2981.232	0.389	0.096	4.068	4.74E-05	1.68E-03
136	11	62792123	62806302	3576.881	0.337	0.083	4.059	4.93E-05	1.73E-03

137	1	225810124	225845563	61.367	0.978	0.241	4.055	5.01E-05	1.75E-03
138	13	32315086	32400268	118.709	0.766	0.190	4.032	5.53E-05	1.89E-03
139	12	75275979	75390928	41.066	1.135	0.282	4.020	5.82E-05	1.97E-03
140	19	18831959	18868230	2564.137	0.356	0.089	4.017	5.90E-05	1.99E-03
141	12	55966781	55972789	518.378	0.380	0.095	4.013	5.99E-05	2.01E-03
142	19	10486125	10503558	756.428	0.458	0.115	4.003	6.26E-05	2.07E-03
143	1	24745382	24844321	109.549	1.131	0.284	3.988	6.66E-05	2.16E-03
144	7	4682295	4771442	1633.596	0.342	0.086	3.978	6.95E-05	2.24E-03
145	8	86514435	86561498	1124.949	0.324	0.082	3.973	7.10E-05	2.28E-03
146	X	149476988	149521096	256.350	0.619	0.156	3.964	7.38E-05	2.36E-03
147	6	158765741	158819368	5951.729	0.271	0.068	3.958	7.57E-05	2.41E-03
148	11	64746389	64759974	183.346	0.946	0.239	3.955	7.67E-05	2.43E-03
149	4	159103013	159360174	602.950	0.477	0.121	3.947	7.92E-05	2.49E-03
150	3	48403854	48430086	81.848	1.030	0.261	3.941	8.11E-05	2.53E-03
151	1	1331280	1335314	54.586	1.171	0.298	3.933	8.40E-05	2.60E-03
152	21	39342315	39349647	4384.869	0.315	0.080	3.928	8.58E-05	2.64E-03
153	2	74471986	74483408	405.663	0.424	0.108	3.923	8.75E-05	2.68E-03
154	4	39498755	39528311	340.340	0.411	0.105	3.912	9.14E-05	2.78E-03
155	5	138352685	138437028	2739.760	0.307	0.079	3.905	9.41E-05	2.84E-03
156	6	41789896	41895375	51.211	1.075	0.276	3.902	9.54E-05	2.88E-03
157	X	48903180	48911958	199.559	0.619	0.159	3.899	9.66E-05	2.90E-03
158	1	28505943	28539300	893.798	0.532	0.137	3.872	1.08E-04	3.18E-03
159	5	150000046	150053142	1106.599	0.343	0.089	3.869	1.09E-04	3.21E-03
160	6	32968594	32981505	9653.030	0.279	0.072	3.860	1.13E-04	3.30E-03
161	17	58556678	58692055	53.090	1.130	0.293	3.854	1.16E-04	3.37E-03
162	10	5154140	5185187	46.217	1.211	0.315	3.842	1.22E-04	3.51E-03
163	16	2520357	2531422	624.770	0.537	0.140	3.840	1.23E-04	3.52E-03
164	19	29665459	29675477	435.149	0.573	0.149	3.839	1.24E-04	3.53E-03
165	7	5306790	5425414	1030.623	0.355	0.093	3.838	1.24E-04	3.53E-03
166	X	54807599	54816015	2479.306	0.365	0.095	3.830	1.28E-04	3.59E-03
167	17	4899418	4902934	19.082	1.826	0.477	3.828	1.29E-04	3.62E-03
168	8	144477969	144502121	264.863	0.552	0.144	3.823	1.32E-04	3.67E-03
169	1	36339624	36385924	1238.098	0.406	0.107	3.810	1.39E-04	3.86E-03
170	2	201700267	201782112	290.162	0.481	0.126	3.803	1.43E-04	3.93E-03
171	17	43014607	43025123	1001.164	0.346	0.091	3.801	1.44E-04	3.95E-03

172	12	120196699	120201235	34378.514	0.379	0.100	3.799	1.45E-04	3.96E-03
173	6	110099819	110180004	53.711	1.218	0.321	3.794	1.48E-04	4.02E-03
174	1	89524829	89597861	298.731	0.416	0.110	3.785	1.54E-04	4.11E-03
175	22	39519695	39522683	8042.525	0.324	0.086	3.781	1.57E-04	4.18E-03
176	6	17615035	17706925	1751.419	0.292	0.077	3.778	1.58E-04	4.22E-03
177	1	45786987	46036122	180.115	0.651	0.172	3.777	1.59E-04	4.23E-03
178	12	56416363	56449426	408.963	0.436	0.115	3.774	1.60E-04	4.26E-03
179	11	278407	285388	190.013	0.585	0.155	3.773	1.61E-04	4.27E-03
180	18	80109262	80147523	762.556	0.337	0.090	3.761	1.69E-04	4.42E-03
181	3	160495007	160565571	1245.738	0.316	0.084	3.760	1.70E-04	4.42E-03
182	12	27523431	27695564	91.557	0.904	0.242	3.731	1.91E-04	4.82E-03
183	17	68267026	68291267	109.133	0.667	0.179	3.723	1.97E-04	4.94E-03
184	6	159969082	160113507	3407.109	0.401	0.108	3.713	2.05E-04	5.13E-03
185	18	10525905	10552764	449.573	0.492	0.133	3.712	2.05E-04	5.13E-03
186	6	26224199	26227473	83.004	0.905	0.244	3.706	2.10E-04	5.20E-03
187	18	23506184	23586506	603.784	0.464	0.125	3.703	2.13E-04	5.26E-03
188	6	151364115	151391559	1150.197	0.319	0.086	3.701	2.15E-04	5.28E-03
189	8	98371228	98942827	43.600	1.077	0.292	3.694	2.21E-04	5.37E-03
190	9	35053928	35072668	3199.826	0.352	0.095	3.693	2.22E-04	5.38E-03
191	17	42851184	42858130	23.201	1.579	0.428	3.691	2.23E-04	5.40E-03
192	19	48872421	48876058	1342.218	0.459	0.125	3.686	2.28E-04	5.47E-03
193	11	67583742	67586656	1300.201	0.413	0.112	3.683	2.30E-04	5.52E-03
194	6	37257772	37332970	368.224	0.439	0.120	3.669	2.43E-04	5.80E-03
195	5	52989340	53094779	18.655	1.553	0.423	3.669	2.44E-04	5.81E-03
196	1	109213918	109238182	3244.651	0.307	0.084	3.661	2.51E-04	5.95E-03
197	15	55180806	55197049	3851.025	0.382	0.104	3.660	2.53E-04	5.96E-03
198	3	126982693	127037389	652.654	0.452	0.124	3.646	2.66E-04	6.20E-03
199	2	117915478	118014133	1484.710	0.269	0.074	3.646	2.66E-04	6.20E-03
200	7	74453790	74602605	139.590	0.649	0.178	3.643	2.69E-04	6.24E-03
201	18	48539031	48863217	78.941	0.815	0.224	3.635	2.77E-04	6.38E-03
202	17	44345246	44353106	450.121	0.685	0.189	3.635	2.78E-04	6.38E-03
203	19	48954815	48961798	1768.990	0.299	0.082	3.634	2.79E-04	6.41E-03
204	X	9463320	9741037	1316.776	0.342	0.094	3.620	2.94E-04	6.66E-03
205	14	51489100	51730727	42.900	1.323	0.366	3.619	2.95E-04	6.68E-03
206	14	24094053	24110598	562.625	0.529	0.146	3.612	3.04E-04	6.84E-03

207	18	22798261	23026488	160.717	0.841	0.234	3.592	3.28E-04	7.29E-03
208	3	50267558	50277546	7.197	2.996	0.835	3.586	3.35E-04	7.40E-03
209	6	33200445	33204439	994.585	0.333	0.093	3.582	3.41E-04	7.50E-03
210	17	20999596	21043760	2541.761	0.292	0.082	3.571	3.56E-04	7.76E-03
211	21	32628759	32728040	506.428	0.401	0.112	3.570	3.57E-04	7.77E-03
212	10	50067888	50133509	1989.709	0.289	0.081	3.569	3.58E-04	7.77E-03
213	21	45073853	45226560	1065.187	0.329	0.092	3.568	3.60E-04	7.79E-03
214	5	36192589	36242279	264.613	0.666	0.187	3.567	3.61E-04	7.80E-03
215	9	34398184	34458570	387.781	0.385	0.108	3.566	3.62E-04	7.80E-03
216	12	123250112	123272334	193.547	0.585	0.164	3.566	3.63E-04	7.80E-03
217	22	40824535	40856639	4568.746	0.340	0.096	3.560	3.71E-04	7.95E-03
218	4	39182504	39285810	676.968	0.387	0.109	3.554	3.79E-04	8.11E-03
219	10	69088103	69104805	1958.142	0.428	0.121	3.548	3.88E-04	8.20E-03
220	4	138164097	138242349	23.749	1.632	0.461	3.544	3.95E-04	8.30E-03
221	10	45727200	45792964	2183.302	0.255	0.072	3.540	4.00E-04	8.37E-03
222	9	137205685	137219361	489.485	0.357	0.101	3.537	4.05E-04	8.44E-03
223	19	52397849	52418412	368.655	0.404	0.115	3.523	4.27E-04	8.78E-03
224	14	50117130	50231578	1222.888	0.345	0.098	3.520	4.32E-04	8.86E-03
225	1	67685201	67688334	164.181	0.532	0.151	3.515	4.40E-04	9.02E-03
226	12	57229573	57234935	1605.536	0.333	0.095	3.513	4.43E-04	9.03E-03
227	1	204396492	204411887	2704.664	0.374	0.107	3.508	4.51E-04	9.16E-03
228	9	128702503	128720916	78.201	0.877	0.250	3.503	4.60E-04	9.28E-03
229	12	122527246	122626396	541.076	0.386	0.110	3.499	4.67E-04	9.38E-03
230	2	20560448	20651130	386.758	0.476	0.137	3.490	4.83E-04	9.60E-03
231	3	47381011	47413435	1017.273	0.341	0.098	3.475	5.12E-04	1.01E-02
232	11	64894546	64917211	803.820	0.583	0.168	3.473	5.15E-04	1.01E-02
233	2	151485336	151734487	43.692	1.155	0.333	3.470	5.20E-04	1.02E-02
234	2	241637213	241673857	1313.332	0.318	0.092	3.465	5.30E-04	1.04E-02
235	11	108157215	108222638	1718.604	0.259	0.075	3.455	5.51E-04	1.07E-02
236	3	49689538	49721529	701.725	0.400	0.116	3.453	5.54E-04	1.08E-02
237	1	160367071	160372846	10.928	2.024	0.587	3.449	5.63E-04	1.09E-02
238	1	211940399	212035557	237.739	0.510	0.148	3.445	5.71E-04	1.10E-02
239	15	72199029	72231819	9492.213	0.397	0.115	3.441	5.80E-04	1.12E-02
240	9	128255829	128276026	1034.948	0.282	0.082	3.438	5.85E-04	1.12E-02
241	12	124324415	124567589	1144.174	0.378	0.110	3.438	5.86E-04	1.12E-02

242	6	89926528	90296908	1369.748	0.262	0.076	3.435	5.93E-04	1.13E-02
243	4	118722823	118838683	423.934	0.357	0.104	3.435	5.93E-04	1.13E-02
244	8	73991392	74029079	108.648	0.764	0.223	3.432	5.98E-04	1.14E-02
245	19	36714383	36772825	101.578	0.642	0.187	3.431	6.02E-04	1.14E-02
246	7	2558972	2614733	180.008	0.586	0.171	3.428	6.07E-04	1.15E-02
247	16	88570403	88631964	1724.444	0.241	0.070	3.428	6.07E-04	1.15E-02
248	1	151540305	151583583	108.761	0.637	0.186	3.428	6.08E-04	1.15E-02
249	X	119574536	119591083	777.535	0.353	0.103	3.426	6.13E-04	1.15E-02
250	17	39980807	39997959	1345.016	0.274	0.080	3.422	6.21E-04	1.16E-02
251	1	155277463	155289848	140.801	0.654	0.191	3.417	6.32E-04	1.18E-02
252	8	23135588	23164027	437.686	0.540	0.158	3.417	6.34E-04	1.18E-02
253	18	50282343	50287839	1827.406	0.328	0.096	3.402	6.70E-04	1.23E-02
254	3	14402576	14489349	1837.922	0.285	0.084	3.398	6.80E-04	1.24E-02
255	19	13906201	13930879	730.166	0.416	0.122	3.395	6.85E-04	1.25E-02
256	3	66378797	66501263	1941.622	0.240	0.071	3.392	6.95E-04	1.26E-02
257	9	93096217	93113283	849.354	0.356	0.105	3.391	6.97E-04	1.26E-02
258	11	68052859	68121444	122.737	0.773	0.228	3.390	6.98E-04	1.26E-02
259	11	125591712	125625215	1432.492	0.323	0.095	3.388	7.03E-04	1.27E-02
260	11	123358428	123627774	197.538	0.505	0.149	3.388	7.05E-04	1.27E-02
261	10	101784443	101818465	6541.235	0.268	0.079	3.386	7.09E-04	1.28E-02
262	19	17281645	17287646	62.345	1.182	0.350	3.378	7.29E-04	1.31E-02
263	12	65169583	65248355	999.471	0.301	0.089	3.373	7.44E-04	1.33E-02
264	14	32076114	32159728	1148.334	0.330	0.098	3.360	7.79E-04	1.39E-02
265	1	2508537	2526597	916.416	0.304	0.091	3.360	7.80E-04	1.39E-02
266	8	126552443	126558478	1740.969	0.280	0.083	3.358	7.85E-04	1.39E-02
267	16	2155698	2178129	894.981	0.301	0.090	3.358	7.86E-04	1.39E-02
268	5	177483394	177497606	76.887	0.789	0.235	3.357	7.88E-04	1.39E-02
269	12	64713445	64759431	1199.590	0.357	0.106	3.356	7.90E-04	1.40E-02
270	17	42659284	42667006	268.897	0.424	0.127	3.353	7.99E-04	1.41E-02
271	16	87696485	87765992	1124.186	0.293	0.088	3.351	8.06E-04	1.42E-02
272	7	139043515	139132122	6059.361	0.234	0.070	3.347	8.18E-04	1.43E-02
273	5	173888349	173961980	230.416	0.465	0.139	3.346	8.20E-04	1.43E-02
274	7	128954180	129055173	1183.186	0.251	0.075	3.343	8.30E-04	1.45E-02
275	18	57600656	57622213	2733.290	0.247	0.074	3.343	8.30E-04	1.45E-02
276	11	57299638	57324952	390.027	0.514	0.154	3.342	8.33E-04	1.45E-02

277	12	132623753	132687376	1148.590	0.440	0.132	3.341	8.36E-04	1.45E-02
278	22	46150521	46243755	427.166	0.509	0.153	3.337	8.46E-04	1.47E-02
279	4	1811479	1856156	784.252	0.303	0.091	3.330	8.68E-04	1.50E-02
280	10	133324072	133336935	37.876	1.254	0.377	3.328	8.75E-04	1.51E-02
281	17	73283624	73312005	13.715	1.681	0.506	3.324	8.88E-04	1.52E-02
282	2	69319780	69387250	690.974	0.292	0.088	3.317	9.11E-04	1.55E-02
283	2	203936763	203961577	1510.687	0.254	0.077	3.313	9.21E-04	1.56E-02
284	21	41986831	42010387	540.917	0.481	0.146	3.306	9.47E-04	1.59E-02
285	3	179236691	179267002	30.613	1.071	0.324	3.303	9.55E-04	1.60E-02
286	6	7107597	7251980	800.861	0.269	0.082	3.303	9.58E-04	1.61E-02
287	9	120751978	120793416	2051.831	0.222	0.067	3.298	9.74E-04	1.63E-02
288	11	32892811	32993316	269.205	0.499	0.151	3.296	9.80E-04	1.64E-02
289	16	3129777	3142804	217.227	0.650	0.197	3.293	9.90E-04	1.65E-02
290	8	73972437	73982783	272.718	0.504	0.153	3.293	9.91E-04	1.65E-02
291	11	64340204	64357534	1591.488	0.369	0.112	3.288	1.01E-03	1.67E-02
292	10	73785606	73801797	1528.886	0.350	0.107	3.288	1.01E-03	1.67E-02
293	19	52031378	52095738	403.796	0.320	0.097	3.285	1.02E-03	1.68E-02
294	5	65722205	65871725	102.806	0.610	0.186	3.285	1.02E-03	1.68E-02
295	4	8182072	8241803	337.506	0.451	0.137	3.282	1.03E-03	1.69E-02
296	8	143566192	143572772	87.811	0.780	0.238	3.281	1.03E-03	1.69E-02
297	6	16299112	16761491	1011.637	0.252	0.077	3.279	1.04E-03	1.70E-02
298	1	145738868	145824095	928.416	0.278	0.085	3.267	1.09E-03	1.76E-02
299	2	238238267	238240662	43.512	0.988	0.302	3.266	1.09E-03	1.77E-02
300	3	49724294	49786542	922.713	0.333	0.102	3.266	1.09E-03	1.77E-02
301	7	151232483	151240979	1163.346	0.275	0.085	3.260	1.12E-03	1.80E-02
302	2	182909115	183038858	123.298	0.640	0.196	3.260	1.12E-03	1.80E-02
303	20	52051663	52204308	213.210	0.505	0.155	3.251	1.15E-03	1.84E-02
304	13	113297239	113323672	3253.795	0.227	0.070	3.250	1.15E-03	1.85E-02
305	3	141876124	141926549	929.798	0.402	0.124	3.243	1.18E-03	1.89E-02
306	22	31122731	31134697	8.971	2.474	0.763	3.240	1.19E-03	1.90E-02
307	2	29097705	29199777	622.103	0.288	0.089	3.237	1.21E-03	1.92E-02
308	7	2251770	2354318	144.543	0.576	0.178	3.230	1.24E-03	1.96E-02
309	17	40140318	40172171	2446.663	0.238	0.074	3.227	1.25E-03	1.98E-02
310	19	6737925	6751530	177.371	0.513	0.160	3.214	1.31E-03	2.03E-02
311	5	10225507	10249897	121.823	0.572	0.178	3.213	1.31E-03	2.03E-02

312	19	20077994	20127076	67.802	0.812	0.253	3.213	1.31E-03	2.03E-02
313	17	29589769	29614761	71.377	0.797	0.248	3.210	1.33E-03	2.05E-02
314	16	28822999	28837237	3684.890	0.264	0.082	3.210	1.33E-03	2.05E-02
315	1	6101787	6180321	12.139	2.009	0.626	3.208	1.34E-03	2.06E-02
316	17	18271428	18315007	745.136	0.274	0.086	3.206	1.35E-03	2.07E-02
317	4	958887	986895	833.885	0.380	0.119	3.205	1.35E-03	2.07E-02
318	22	50508224	50524780	1052.304	0.294	0.092	3.204	1.36E-03	2.08E-02
319	5	173056352	173139284	1738.509	0.287	0.090	3.193	1.41E-03	2.14E-02
320	14	22893204	22919182	1855.696	0.248	0.078	3.192	1.41E-03	2.15E-02
321	1	100872372	100895179	114.259	0.650	0.204	3.189	1.43E-03	2.17E-02
322	1	155234452	155244699	524.151	0.444	0.139	3.186	1.44E-03	2.19E-02
323	11	33703010	33736479	1555.634	0.321	0.101	3.177	1.49E-03	2.25E-02
324	12	57089043	57095476	210.233	0.415	0.131	3.173	1.51E-03	2.28E-02
325	X	53532096	53686728	5356.156	0.226	0.071	3.172	1.51E-03	2.28E-02
326	4	173331376	173334432	2574.831	0.358	0.113	3.169	1.53E-03	2.30E-02
327	12	121804009	121832656	2458.626	0.226	0.071	3.167	1.54E-03	2.31E-02
328	7	101205977	101218420	611.817	0.394	0.125	3.155	1.61E-03	2.39E-02
329	X	101393273	101408012	186.912	0.561	0.178	3.152	1.62E-03	2.41E-02
330	5	177086905	177098144	26.766	1.471	0.467	3.150	1.63E-03	2.42E-02
331	12	115957905	116277693	2097.140	0.300	0.095	3.148	1.64E-03	2.43E-02
332	5	154682986	154817605	4511.854	0.199	0.063	3.146	1.65E-03	2.44E-02
333	19	44954585	44993341	1192.762	0.305	0.098	3.130	1.75E-03	2.54E-02
334	4	47914142	48040173	16.020	1.395	0.446	3.128	1.76E-03	2.55E-02
335	20	36306336	36528637	883.631	0.248	0.079	3.128	1.76E-03	2.55E-02
336	18	49782164	49813953	449.946	0.340	0.109	3.127	1.77E-03	2.55E-02
337	2	60756253	60802086	771.004	0.353	0.113	3.127	1.77E-03	2.55E-02
338	1	149937812	150010726	235.814	0.386	0.124	3.126	1.77E-03	2.55E-02
339	5	69093991	69131069	809.691	0.314	0.100	3.126	1.77E-03	2.56E-02
340	19	7522624	7534009	151.633	0.749	0.240	3.124	1.79E-03	2.57E-02
341	19	10960932	11079426	1959.276	0.245	0.079	3.121	1.80E-03	2.58E-02
342	22	36487190	36507221	123.668	0.631	0.202	3.119	1.81E-03	2.59E-02
343	12	6534512	6538374	10231.479	0.290	0.093	3.117	1.83E-03	2.61E-02
344	12	64404392	64451125	3447.269	0.288	0.093	3.112	1.86E-03	2.64E-02
345	2	196763035	196799725	815.107	0.263	0.085	3.110	1.87E-03	2.65E-02
346	14	61695513	61748259	1297.330	0.292	0.094	3.107	1.89E-03	2.68E-02

347	19	19645198	19663676	2193.763	0.291	0.094	3.100	1.94E-03	2.73E-02
348	1	19074510	19210266	5305.516	0.308	0.099	3.097	1.95E-03	2.75E-02
349	10	63521401	63625128	242.755	0.405	0.131	3.096	1.96E-03	2.76E-02
350	7	5620047	5781696	2690.731	0.227	0.073	3.095	1.97E-03	2.76E-02
351	20	32192504	32207743	530.216	0.329	0.107	3.091	2.00E-03	2.80E-02
352	1	151036321	151047720	60.996	0.971	0.314	3.089	2.01E-03	2.80E-02
353	12	112160188	112382439	1335.289	0.304	0.099	3.086	2.03E-03	2.82E-02
354	16	2088708	2135898	1296.091	0.356	0.115	3.079	2.08E-03	2.87E-02
355	6	127708072	127918631	1060.223	0.291	0.095	3.075	2.11E-03	2.89E-02
356	20	62302093	62308862	1039.828	0.362	0.118	3.074	2.11E-03	2.89E-02
357	1	151159748	151165948	48.348	0.784	0.255	3.070	2.14E-03	2.92E-02
358	9	136428619	136439845	711.679	0.320	0.104	3.069	2.15E-03	2.92E-02
359	19	4174109	4182566	310.244	0.489	0.160	3.063	2.19E-03	2.97E-02
360	13	31134973	31162388	1705.841	0.395	0.129	3.060	2.21E-03	2.99E-02
361	11	72080337	72096895	46.371	0.816	0.268	3.049	2.30E-03	3.08E-02
362	19	925781	975939	134.593	0.496	0.163	3.047	2.31E-03	3.10E-02
363	19	6210381	6279975	1198.825	0.254	0.083	3.046	2.32E-03	3.10E-02
364	21	33642400	33899861	115.889	0.623	0.205	3.044	2.33E-03	3.12E-02
365	7	101232092	101238820	722.578	0.302	0.099	3.040	2.37E-03	3.15E-02
366	12	56162359	56189567	3324.392	0.214	0.070	3.037	2.39E-03	3.18E-02
367	12	2794970	2805423	1151.510	0.388	0.128	3.037	2.39E-03	3.18E-02
368	6	27838545	27855709	9.669	2.837	0.935	3.033	2.42E-03	3.21E-02
369	1	154220179	154271510	2650.422	0.213	0.070	3.028	2.46E-03	3.24E-02
370	11	65615776	65637439	1406.194	0.284	0.094	3.021	2.52E-03	3.30E-02
371	19	12064731	12081565	279.219	0.559	0.185	3.021	2.52E-03	3.30E-02
372	7	6161776	6272644	313.934	0.416	0.138	3.020	2.52E-03	3.30E-02
373	6	70667776	70862011	774.767	0.306	0.101	3.018	2.54E-03	3.32E-02
374	1	149923317	149927803	1095.914	0.340	0.113	3.016	2.56E-03	3.34E-02
375	11	64305497	64316743	374.783	0.412	0.137	3.014	2.58E-03	3.35E-02
376	1	224385146	224437033	3692.519	0.269	0.089	3.013	2.58E-03	3.35E-02
377	6	145793502	145814795	185.453	0.731	0.243	3.010	2.61E-03	3.38E-02
378	21	33559542	33588706	134.865	0.472	0.157	3.005	2.66E-03	3.43E-02
379	19	6463777	6467221	46.576	0.871	0.291	2.995	2.75E-03	3.53E-02
380	4	88457119	88506163	449.158	0.380	0.127	2.993	2.76E-03	3.53E-02
381	1	15836095	15940456	2799.645	0.309	0.104	2.986	2.83E-03	3.61E-02

382	16	57735739	57757244	412.288	0.324	0.109	2.986	2.83E-03	3.61E-02
383	3	187721377	187745725	407.927	0.299	0.100	2.984	2.84E-03	3.61E-02
384	7	135148072	135166215	231.245	0.531	0.178	2.976	2.92E-03	3.69E-02
385	1	200404940	200410056	638.665	0.281	0.094	2.975	2.93E-03	3.69E-02
386	19	58544064	58550722	4206.449	0.273	0.092	2.975	2.93E-03	3.69E-02
387	16	70661204	70686053	51.556	0.846	0.285	2.972	2.96E-03	3.72E-02
388	19	5691834	5720572	1160.826	0.391	0.132	2.966	3.01E-03	3.77E-02
389	1	20633458	20651511	658.939	0.292	0.099	2.966	3.02E-03	3.77E-02
390	7	106248298	106285966	647.638	0.324	0.109	2.964	3.03E-03	3.78E-02
391	19	2236824	2248655	1208.105	0.363	0.123	2.960	3.08E-03	3.82E-02
392	14	24171853	24180257	252.634	0.380	0.128	2.960	3.08E-03	3.82E-02
393	1	113696831	113759489	182.741	0.437	0.148	2.958	3.10E-03	3.84E-02
394	2	189031898	189225312	38.092	1.055	0.357	2.957	3.11E-03	3.84E-02
395	1	235447190	235504452	305.829	0.375	0.127	2.953	3.14E-03	3.87E-02
396	X	48961378	48971844	29.323	1.373	0.465	2.953	3.15E-03	3.87E-02
397	6	30600413	30618612	1457.033	0.253	0.086	2.953	3.15E-03	3.87E-02
398	17	18315293	18328056	2514.776	0.262	0.089	2.951	3.17E-03	3.89E-02
399	19	14408798	14419383	2773.357	0.320	0.108	2.950	3.18E-03	3.89E-02
400	19	39342421	39385710	773.762	0.270	0.092	2.949	3.19E-03	3.90E-02
401	11	57667747	57701182	1995.230	0.256	0.087	2.947	3.21E-03	3.91E-02
402	16	58025754	58046901	17.656	1.484	0.504	2.945	3.23E-03	3.94E-02
403	5	132875395	132963634	1613.285	0.251	0.085	2.943	3.25E-03	3.95E-02
404	17	56978129	57006768	396.648	0.327	0.111	2.940	3.28E-03	3.97E-02
405	1	43389882	43454247	2104.642	0.246	0.084	2.937	3.31E-03	4.02E-02
406	6	26277609	26285638	33.429	1.086	0.370	2.937	3.32E-03	4.02E-02
407	2	218633329	218659655	904.294	0.293	0.100	2.935	3.34E-03	4.03E-02
408	19	50025714	50053414	245.066	0.395	0.135	2.931	3.37E-03	4.07E-02
409	2	128236716	128318868	176.901	0.427	0.146	2.930	3.39E-03	4.08E-02
410	15	72474330	72602987	1591.592	0.229	0.078	2.930	3.39E-03	4.08E-02
411	5	52787916	52804044	209.030	0.591	0.202	2.924	3.45E-03	4.14E-02
412	14	49598761	49614672	198.418	0.448	0.153	2.923	3.47E-03	4.15E-02
413	20	58309715	58367507	1027.789	0.258	0.088	2.923	3.47E-03	4.15E-02
414	1	40450102	40463718	112.614	0.662	0.227	2.919	3.51E-03	4.19E-02
415	17	76712832	76726799	602.355	0.326	0.112	2.918	3.52E-03	4.20E-02
416	18	36108531	36129385	645.418	0.269	0.092	2.918	3.52E-03	4.20E-02

417	X	48973720	49002264	1380.086	0.286	0.098	2.910	3.62E-03	4.28E-02
418	3	46979666	47009704	2388.425	0.262	0.090	2.909	3.62E-03	4.28E-02
419	12	132136594	132144319	1047.244	0.265	0.091	2.909	3.63E-03	4.28E-02
420	11	66257294	66267860	485.103	0.343	0.118	2.908	3.64E-03	4.28E-02
421	7	56011051	56051604	142.892	0.501	0.173	2.904	3.68E-03	4.32E-02
422	1	35713877	35718894	259.982	0.383	0.132	2.901	3.72E-03	4.35E-02
423	14	105489855	105499575	151.684	0.481	0.166	2.900	3.73E-03	4.36E-02
424	2	218264129	218270178	1310.080	0.281	0.097	2.895	3.79E-03	4.41E-02
425	9	122908056	122913323	380.162	0.347	0.120	2.895	3.80E-03	4.41E-02
426	4	89236383	89307800	1296.142	0.227	0.079	2.893	3.82E-03	4.43E-02
427	20	5544439	5611006	1704.064	0.300	0.104	2.890	3.85E-03	4.45E-02
428	19	36687612	36727701	253.288	0.381	0.132	2.890	3.85E-03	4.45E-02
429	8	124474880	124488618	790.798	0.291	0.101	2.889	3.86E-03	4.45E-02
430	4	119494397	119628804	27.265	1.297	0.449	2.889	3.86E-03	4.45E-02
431	19	54173412	54189882	581.329	0.339	0.118	2.887	3.89E-03	4.46E-02
432	6	80003887	80042527	13.090	1.635	0.567	2.886	3.90E-03	4.48E-02
433	1	151510510	151538692	71.481	0.718	0.249	2.882	3.95E-03	4.52E-02
434	6	41546381	41602384	164.183	0.765	0.266	2.881	3.96E-03	4.53E-02
435	12	52022832	52059507	16.061	1.578	0.548	2.879	3.99E-03	4.55E-02
436	18	45847609	45967329	1471.128	0.255	0.089	2.878	4.00E-03	4.55E-02
437	5	69415065	69444330	22.151	1.236	0.430	2.878	4.00E-03	4.55E-02
438	4	6781375	6884170	881.831	0.291	0.101	2.871	4.09E-03	4.64E-02
439	16	67873052	67884499	1263.354	0.265	0.092	2.870	4.10E-03	4.64E-02
440	11	64823052	64844653	252.252	0.390	0.136	2.867	4.15E-03	4.68E-02
441	16	67657512	67660815	523.489	0.362	0.126	2.865	4.17E-03	4.70E-02
442	17	82519713	82644662	1096.320	0.292	0.102	2.854	4.32E-03	4.83E-02
443	19	984332	998438	430.567	0.510	0.179	2.852	4.34E-03	4.85E-02
444	7	64307459	64356634	332.051	0.378	0.133	2.848	4.40E-03	4.90E-02
445	15	75647912	75662301	132.952	0.664	0.234	2.845	4.45E-03	4.94E-02
446	19	57435325	57447101	147.988	0.438	0.154	2.842	4.48E-03	4.96E-02
447	19	57240632	57262728	248.422	0.359	0.126	2.840	4.52E-03	4.99E-02

c

Order	Ensembl ID	Gene Symbol	Gene Name	UniProt
1	ENSG00000205542	***TMSB4X***	thymosin beta 4 X-linked	P62328
2	ENSG00000166598	***HSP90B1***	heat shock protein 90 beta family member 1	P14625
3	ENSG00000044574	***HSPA5***	heat shock protein family A (Hsp70) member 5	P11021
4	ENSG00000184752	***NDUFA12***	NADH:ubiquinone oxidoreductase subunit A12	Q9UI09
5	ENSG00000111348	***ARHGDIB***	Rho GDP dissociation inhibitor beta	P52566
6	ENSG00000065518	***NDUFB4***	NADH:ubiquinone oxidoreductase subunit B4	O95168
7	ENSG00000167004	***PDIA3***	protein disulfide isomerase family A member 3	P30101
8	ENSG00000034510	***TMSB10***	thymosin beta 10	P63313
9	ENSG00000265972	***TXNIP***	thioredoxin interacting protein	Q9H3M7
10	ENSG00000145050	***MANF***	mesencephalic astrocyte derived neurotrophic factor	P55145
11	ENSG00000111481	***COPZ1***	COPI coat complex subunit zeta 1	P61923
12	ENSG00000165629	***ATP5F1C***	ATP synthase F1 subunit gamma	P36542
13	ENSG00000080824	***HSP90AA1***	heat shock protein 90 alpha family class A member 1	P07900

Table 1c continued

Order	chr	start	end	baseMean	log2FoldChange	lfcSE	stat	pvalue	padj
1	X	12975110	12977227	53784.953	-0.300	0.063	-4.761	1.93E-06	1.456E-03
2	12	103930107	103953931	9262.938	-0.336	0.071	-4.751	2.02E-06	1.456E-03
3	9	125234853	125241382	5535.011	-0.382	0.081	-4.735	2.19E-06	1.489E-03
4	12	94895297	95003748	1467.880	-0.347	0.082	-4.211	2.55E-05	1.178E-02
5	12	14942031	14961728	18341.064	-0.224	0.057	-3.913	9.10E-05	3.021E-02
6	3	120596328	120602507	923.826	-0.345	0.090	-3.848	1.19E-04	3.589E-02
7	15	43746394	43773279	5053.715	-0.275	0.072	-3.817	1.35E-04	3.820E-02
8	2	84905656	84906671	18121.778	-0.287	0.076	-3.797	1.46E-04	3.951E-02
9	1	145992435	145996579	19375.847	-0.185	0.049	-3.790	1.50E-04	3.973E-02
10	3	51385291	51389397	661.685	-0.427	0.113	-3.777	1.59E-04	4.114E-02
11	12	54301202	54351846	1920.473	-0.221	0.060	-3.711	2.07E-04	4.787E-02
12	10	7788147	7807815	1803.183	-0.277	0.075	-3.706	2.11E-04	4.787E-02
13	14	102080742	102139699	12647.574	-0.264	0.072	-3.693	2.22E-04	4.787E-02

d

Order	Ensembl ID	Gene Symbol	Gene Name	UniProt
1	ENSG00000110090	***CPT1A***	carnitine palmitoyltransferase 1A	P50416
2	ENSG00000178537	***SLC25A20***	solute carrier family 25 member 20	O43772
3	ENSG00000145860	***RNF145***	ring finger protein 145	Q96MT1
4	ENSG00000072778	***ACADVL***	acyl-CoA dehydrogenase very long chain	P49748
5	ENSG00000143110	***C1orf162***	chromosome 1 open reading frame 162	Q8NEQ5
6	ENSG00000153395	***LPCAT1***	lysophosphatidylcholine acyltransferase 1	Q8NF37
7	ENSG00000184602	***SNN***	stannin	O75324
8	ENSG00000116133	***DHCR24***	24-dehydrocholesterol reductase	Q15392
9	ENSG00000169710	***FASN***	fatty acid synthase	P49327
10	ENSG00000198911	***SREBF2***	sterol regulatory element binding transcription factor 2	Q12772
11	ENSG00000102032	***RENBP***	renin binding protein	P51606
12	ENSG00000130164	***LDLR***	low density lipoprotein receptor	P01130
13	ENSG00000167315	***ACAA2***	acetyl-CoA acyltransferase 2	P42765
14	ENSG00000166575	***TMEM135***	transmembrane protein 135	Q86UB9
15	ENSG00000167106	***FAM102A***	estrogen-induced osteoclastogenesis regulator 1	Q5T9C2
16	ENSG00000103249	***CLCN7***	chloride voltage-gated channel 7	P51798
17	ENSG00000069424	***KCNAB2***	potassium voltage-gated channel subfamily A regulatory beta subunit 2	Q13303
18	ENSG00000113161	***HMGCR***	3-hydroxy-3-methylglutaryl-CoA reductase	P04035
19	ENSG00000198355	***PIM3***	Pim-3 proto-oncogene, serine/threonine kinase	Q86V86
20	ENSG00000171503	***ETFDH***	electron transfer flavoprotein dehydrogenase	Q16134
21	ENSG00000140526	***ABHD2***	abhydrolase domain containing 2, acylglycerol lipase	P08910
22	ENSG00000174903	***RAB1B***	RAB1B, member RAS oncogene family	Q9H0U4
23	ENSG00000106266	***SNX8***	sorting nexin 8	Q9Y5X2
24	ENSG00000079432	***CIC***	capicua transcriptional repressor	Q96RK0
25	ENSG00000163162	***RNF149***	ring finger protein 149	Q8NC42
26	ENSG00000161011	***SQSTM1***	sequestosome 1	Q13501
27	ENSG00000172059	***KLF11***	KLF transcription factor 11	O14901
28	ENSG00000158470	***B4GALT5***	beta-1,4-galactosyltransferase 5	O43286
29	ENSG00000011021	***CLCN6***	chloride voltage-gated channel 6	P51797
30	ENSG00000113163	***CERT1***	ceramide transporter 1	Q9Y5P4
31	ENSG00000148154	***UGCG***	UDP-glucose ceramide glucosyltransferase	Q16739

32	ENSG00000100596	***SPTLC2***	serine palmitoyltransferase long chain base subunit 2	O15270
33	ENSG00000162066	***AMDHD2***	amidohydrolase domain containing 2	Q9Y303
34	ENSG00000167895	***TMC8***	transmembrane channel like 8	Q8IU68
35	ENSG00000154803	***FLCN***	folliculin	Q8NFG4
36	ENSG00000119537	***KDSR***	3-ketodihydrosphingosine reductase	Q06136
37	ENSG00000122515	***ZMIZ2***	zinc finger MIZ-type containing 2	Q8NF64
38	ENSG00000101752	***MIB1***	MIB E3 ubiquitin protein ligase 1	Q86YT6
39	ENSG00000112972	***HMGCS1***	3-hydroxy-3-methylglutaryl-CoA synthase 1	Q01581
40	ENSG00000178607	***ERN1***	endoplasmic reticulum to nucleus signaling 1	O75460
41	ENSG00000155090	***KLF10***	KLF transcription factor 10	Q13118
42	ENSG00000186174	***BCL9L***	BCL9 like	Q86UU0
43	ENSG00000125534	***PPDPF***	pancreatic progenitor cell differentiation and proliferation factor	Q9H3Y8
44	ENSG00000109572	***CLCN3***	chloride voltage-gated channel 3	P51790
45	ENSG00000087074	***PPP1R15A***	protein phosphatase 1 regulatory subunit 15A	O75807
46	ENSG00000186480	***INSIG1***	insulin induced gene 1	O15503
47	ENSG00000168488	***ATXN2L***	ataxin 2 like	Q8WWM7

Table 1d continued

Order	chr	start	end	baseMean	log2FoldChange	lfcSE	stat	pvalue	padj
1	11	68754620	68844410	893.439	3.460	0.110	31.535	2.87E-218	3.71E-214
2	3	48856926	48898904	352.353	1.669	0.121	13.846	1.34E-43	8.65E-40
3	5	159157409	159210053	2335.306	0.638	0.068	9.369	7.35E-21	3.17E-17
4	17	7217125	7225266	3128.902	0.557	0.064	8.717	2.87E-18	9.28E-15
5	1	111473792	111478512	1332.128	0.522	0.072	7.263	3.78E-13	9.78E-10
6	5	1456480	1523962	1527.598	0.473	0.067	7.089	1.35E-12	2.59E-09
7	16	11668455	11679152	905.754	0.581	0.082	7.084	1.40E-12	2.59E-09
8	1	54849627	54887195	474.003	0.588	0.093	6.295	3.08E-10	4.98E-07
9	17	82078338	82098294	866.664	0.680	0.116	5.858	4.68E-09	6.74E-06
10	22	41833079	41907307	3184.981	0.390	0.070	5.582	2.37E-08	3.07E-05
11	X	153935269	153944687	250.558	0.747	0.142	5.245	1.57E-07	1.84E-04
12	19	11089462	11133820	1013.144	0.461	0.088	5.211	1.87E-07	2.02E-04
13	18	49782164	49813953	483.439	0.558	0.108	5.179	2.23E-07	2.22E-04
14	11	87037844	87328824	161.166	0.722	0.146	4.951	7.39E-07	6.83E-04
15	9	127940582	127980989	7915.593	0.290	0.060	4.821	1.43E-06	1.24E-03
16	16	1444934	1475084	1764.038	0.411	0.086	4.766	1.88E-06	1.46E-03
17	1	5990927	6101193	4855.716	0.308	0.065	4.725	2.30E-06	1.49E-03
18	5	75336329	75364001	913.374	0.369	0.081	4.544	5.52E-06	3.40E-03
19	22	49960768	49964072	716.562	0.450	0.102	4.406	1.05E-05	6.20E-03
20	4	158671968	158710742	301.259	0.553	0.127	4.343	1.41E-05	7.92E-03
21	15	89087459	89202355	1283.746	0.291	0.068	4.301	1.70E-05	9.19E-03
22	11	66268590	66277492	2107.877	0.334	0.078	4.271	1.95E-05	1.009E-02
23	7	2251770	2354318	144.357	0.710	0.168	4.224	2.40E-05	1.178E-02
24	19	42268537	42295797	2107.033	0.347	0.082	4.212	2.53E-05	1.178E-02
25	2	101271219	101308701	1907.621	0.340	0.081	4.172	3.02E-05	1.347E-02
26	5	179806398	179838078	3912.137	0.262	0.063	4.155	3.26E-05	1.406E-02
27	2	10042849	10054836	161.305	0.718	0.174	4.129	3.65E-05	1.524E-02
28	20	49632945	49713878	294.347	0.478	0.120	3.986	6.72E-05	2.687E-02
29	1	11806096	11848079	556.903	0.388	0.098	3.982	6.85E-05	2.687E-02
30	5	75356345	75512138	911.346	0.347	0.088	3.959	7.53E-05	2.849E-02
31	9	111896814	111935369	536.183	0.381	0.097	3.948	7.89E-05	2.849E-02

32	14	77505997	77616637	1124.077	0.308	0.078	3.944	8.02E-05	2.849E-02
33	16	2520357	2531422	610.025	0.482	0.122	3.940	8.14E-05	2.849E-02
34	17	78130770	78142968	9200.496	0.262	0.067	3.915	9.04E-05	3.021E-02
35	17	17212212	17237188	1228.137	0.260	0.067	3.896	9.78E-05	3.165E-02
36	18	63327726	63367228	1221.676	0.302	0.079	3.851	1.18E-04	3.589E-02
37	7	44748581	44769881	1865.416	0.271	0.071	3.849	1.18E-04	3.589E-02
38	18	21704957	21870953	891.081	0.361	0.095	3.816	1.36E-04	3.820E-02
39	5	43287470	43313512	800.614	0.315	0.083	3.816	1.36E-04	3.820E-02
40	17	64039080	64130819	2705.223	0.215	0.056	3.807	1.41E-04	3.873E-02
41	8	102648784	102655725	40.814	1.297	0.345	3.762	1.68E-04	4.178E-02
42	11	118893875	118925926	5140.145	0.295	0.079	3.759	1.71E-04	4.178E-02
43	20	63520765	63522206	897.063	0.298	0.079	3.758	1.71E-04	4.178E-02
44	4	169612633	169723673	1250.518	0.275	0.074	3.734	1.88E-04	4.511E-02
45	19	48872421	48876058	1262.788	0.344	0.093	3.696	2.19E-04	4.787E-02
46	7	155297776	155310235	944.944	0.283	0.077	3.696	2.19E-04	4.787E-02
47	16	28822999	28837237	3753.846	0.254	0.069	3.693	2.22E-04	4.787E-02

e

Order	Ensembl ID	Gene Symbol	Gene Name	UniProt
1	ENSG00000117632	***STMN1***	stathmin 1	P16949
2	ENSG00000163659	***TIPARP***	TCDD inducible poly(ADP-ribose) polymerase	Q7Z3E1
3	ENSG00000153395	***LPCAT1***	lysophosphatidylcholine acyltransferase 1	Q8NF37
4	ENSG00000113161	***HMGCR***	3-hydroxy-3-methylglutaryl-CoA reductase	P04035
5	ENSG00000118816	***CCNI***	cyclin I	Q14094
6	ENSG00000145741	***BTF3***	basic transcription factor 3	P20290
7	ENSG00000006451	***RALA***	RAS like proto-oncogene A	P11233
8	ENSG00000145860	***RNF145***	ring finger protein 145	Q96MT1
9	ENSG00000187109	***NAP1L1***	nucleosome assembly protein 1 like 1	P55209
10	ENSG00000128989	***ARPP19***	cAMP regulated phosphoprotein 19	P56211
11	ENSG00000104549	***SQLE***	squalene epoxidase	Q14534
12	ENSG00000117592	***PRDX6***	peroxiredoxin 6	P30041
13	ENSG00000153283	***CD96***	CD96 molecule	P40200
14	ENSG00000130741	***EIF2S3***	eukaryotic translation initiation factor 2 subunit gamma	P41091
15	ENSG00000125868	***DSTN***	destrin, actin depolymerizing factor	P60981

Table 1e continued

Order	chr	start	end	baseMean	log2FoldChange	lfcSE	stat	pvalue	padj
1	1	25884181	25906991	1324.856	-0.407	0.067	-6.050	1.44E-09	2.68E-06
2	3	156673235	156706770	2383.237	-0.329	0.058	-5.653	1.57E-08	2.04E-05
3	5	1456480	1523962	1166.324	-0.276	0.058	-4.718	2.38E-06	2.57E-03
4	5	75336329	75364001	726.112	-0.325	0.070	-4.634	3.58E-06	3.28E-03
5	4	77047155	77076309	4732.443	-0.218	0.047	-4.612	3.99E-06	3.28E-03
6	5	73498408	73505667	10617.724	-0.255	0.056	-4.596	4.30E-06	3.28E-03
7	7	39623565	39708120	1137.560	-0.283	0.067	-4.201	2.66E-05	1.724E-02
8	5	159157409	159210053	1666.498	-0.287	0.069	-4.189	2.81E-05	1.733E-02
9	12	76036585	76084735	12784.530	-0.322	0.077	-4.174	3.00E-05	1.768E-02
10	15	52547045	52569883	1868.746	-0.278	0.067	-4.143	3.43E-05	1.932E-02
11	8	124998497	125022283	334.452	-0.364	0.090	-4.071	4.68E-05	2.427E-02
12	1	173477330	173488815	1454.171	-0.211	0.053	-3.948	7.87E-05	3.518E-02
13	3	111292719	111665750	6532.967	-0.210	0.054	-3.882	1.04E-04	4.298E-02
14	X	24054946	24078810	5440.858	-0.211	0.055	-3.875	1.07E-04	4.298E-02
15	20	17570075	17609919	923.114	-0.270	0.070	-3.869	1.09E-04	4.298E-02

f

Order	Ensembl ID	Gene Symbol	Gene Name	UniProt
1	ENSG00000110090	***CPT1A***	carnitine palmitoyltransferase 1A	P50416
2	ENSG00000178537	***SLC25A20***	solute carrier family 25 member 20	O43772
3	ENSG00000072778	***ACADVL***	acyl-CoA dehydrogenase very long chain	P49748
4	ENSG00000072310	***SREBF1***	sterol regulatory element binding transcription factor 1	P36956
5	ENSG00000143110	***C1orf162***	chromosome 1 open reading frame 162	Q8NEQ5
6	ENSG00000111684	***LPCAT3***	lysophosphatidylcholine acyltransferase 3	Q6P1A2
7	ENSG00000160179	***ABCG1***	ATP binding cassette subfamily G member 1	P45844
8	ENSG00000167315	***ACAA2***	acetyl-CoA acyltransferase 2	P42765
9	ENSG00000166575	***TMEM135***	transmembrane protein 135	Q86UB9
10	ENSG00000149428	***HYOU1***	hypoxia up-regulated 1	Q9Y4L1
11	ENSG00000099194	***SCD***	stearoyl-CoA desaturase	O00767
12	ENSG00000196155	***PLEKHG4***	pleckstrin homology and RhoGEF domain containing G4	Q58EX7
13	ENSG00000182871	***COL18A1***	collagen type XVIII alpha 1 chain	P39060
14	ENSG00000278540	***ACACA***	acetyl-CoA carboxylase alpha	Q13085
15	ENSG00000141524	***TMC6***	transmembrane channel like 6	Q7Z403
16	ENSG00000079432	***CIC***	capicua transcriptional repressor	Q96RK0
17	ENSG00000182095	***TNRC18***	trinucleotide repeat containing 18	O15417
18	ENSG00000155090	***KLF10***	KLF transcription factor 10	Q13118

Table 1f continued

Order	chr	start	end	baseMean	log2FoldChange	lfcSE	stat	pvalue	padj
1	11	68754620	68844410	709.522	3.173	0.112	28.403	1.88E-177	2.44E-173
2	3	48856926	48898904	328.362	1.552	0.099	15.711	1.28E-55	8.30E-52
3	17	7217125	7225266	3226.461	0.642	0.055	11.620	3.26E-31	1.41E-27
4	17	17810399	17837002	748.306	1.252	0.125	10.021	1.23E-23	3.99E-20
5	1	111473792	111478512	1302.359	0.464	0.058	7.952	1.83E-15	4.76E-12
6	12	6976185	7018477	415.027	0.690	0.109	6.342	2.26E-10	4.89E-07
7	21	42199689	42297244	158.548	1.060	0.176	6.019	1.75E-09	2.84E-06
8	18	49782164	49813953	486.997	0.560	0.095	5.893	3.79E-09	5.47E-06
9	11	87037844	87328824	164.063	0.743	0.148	5.003	5.64E-07	6.65E-04
10	11	119044188	119057227	2099.100	0.321	0.069	4.619	3.85E-06	3.28E-03
11	10	100347233	100364826	183.550	0.595	0.129	4.606	4.11E-06	3.28E-03
12	16	67277510	67289499	674.104	0.305	0.072	4.242	2.22E-05	1.599E-02
13	21	45405165	45513720	687.338	0.507	0.120	4.216	2.49E-05	1.698E-02
14	17	37084992	37406836	410.104	0.359	0.088	4.078	4.54E-05	2.427E-02
15	17	78107397	78132407	9025.505	0.274	0.068	4.046	5.20E-05	2.595E-02
16	19	42268537	42295797	2111.939	0.362	0.091	3.967	7.28E-05	3.500E-02
17	7	5306790	5425414	1055.115	0.343	0.087	3.958	7.56E-05	3.504E-02
18	8	102648784	102655725	39.296	1.179	0.302	3.909	9.28E-05	4.013E-02

g.i

Overlapping Genes EPA vs OA vs PA												
**Overlapping Genes**	**ensembl**	**chr**	**log2FC EPA**	**log2FC OA**	**log2FC PA**	**pvalue EPA**	**pvalue OA**	**pvalue PA**	**padj EPA**	**padj OA**	**padj PA**	**Direction Same**
CPT1A	ENSG00000110090	11	2.716	3.460	3.173	8.52E-24	2.87E-218	1.88E-177	3.68E-20	3.71E-214	2.44E-173	Yes Up
SLC25A20	ENSG00000178537	3	1.144	1.669	1.552	3.55E-17	1.34E-43	1.28E-55	6.57E-14	8.65E-40	8.30E-52	Yes Up
ACADVL	ENSG00000072778	17	0.541	0.557	0.642	2.37E-11	2.87E-18	3.26E-31	9.91E-09	9.28E-15	1.41E-27	Yes Up
ACAA2	ENSG00000167315	18	0.340	0.558	0.560	1.77E-03	2.23E-07	3.79E-09	2.55E-02	2.22E-04	5.47E-06	Yes Up

g.ii

Overlapping Genes EPA vs OA									
**Overlapping Genes**	**ensembl**	**chr**	**log2FC EPA**	**log2FC OA**	**pvalue EPA**	**pvalue OA**	**padj EPA**	**padj OA**	**Direction Same**
TMSB4X	ENSG00000205542	X	-0.577	-0.300	7.52E-16	1.93E-06	1.08E-12	1.46E-03	Yes Down
PPDPF	ENSG00000125534	20	0.876	0.298	9.86E-14	1.71E-04	8.50E-11	4.18E-02	Yes Up
TMSB10	ENSG00000034510	2	-0.622	-0.287	1.37E-11	1.46E-04	6.08E-09	3.95E-02	Yes Down
CLCN7	ENSG00000103249	16	0.599	0.411	1.76E-09	1.88E-06	3.74E-07	1.46E-03	Yes Up
ETFDH	ENSG00000171503	4	0.758	0.553	1.98E-08	1.41E-05	2.84E-06	7.92E-03	Yes Up
SQSTM1	ENSG00000161011	5	0.618	0.262	4.20E-08	3.26E-05	5.49E-06	1.41E-02	Yes Up
RENBP	ENSG00000102032	X	0.716	0.747	8.37E-06	1.57E-07	4.15E-04	1.84E-04	Yes Up
PIM3	ENSG00000198355	22	0.479	0.450	2.24E-05	1.05E-05	9.14E-04	6.20E-03	Yes Up
TXNIP	ENSG00000265972	1	-0.278	-0.185	2.93E-05	1.50E-04	1.13E-03	3.97E-02	Yes Down
CLCN6	ENSG00000011021	1	0.522	0.388	4.06E-05	6.85E-05	1.47E-03	2.69E-02	Yes Up
FLCN	ENSG00000154803	17	0.348	0.260	4.13E-05	9.78E-05	1.49E-03	3.17E-02	Yes Up
AMDHD2	ENSG00000162066	16	0.537	0.482	1.23E-04	8.14E-05	3.52E-03	2.85E-02	Yes Up
PPP1R15A	ENSG00000087074	19	0.459	0.344	2.28E-04	2.19E-04	5.47E-03	4.79E-02	Yes Up
HSP90B1	ENSG00000166598	12	-0.305	-0.336	3.50E-04	2.02E-06	7.64E-03	1.46E-03	Yes Down
ARHGDIB	ENSG00000111348	12	-0.247	-0.224	6.67E-04	9.10E-05	1.23E-02	3.02E-02	Yes Down
SNX8	ENSG00000106266	7	0.576	0.710	1.24E-03	2.40E-05	1.96E-02	1.18E-02	Yes Up

g.iii

**Overlapping Genes EPA vs PA**									
**Overlapping Genes**	**ensembl**	**chr**	**log2FC EPA**	**log2FC PA**	**pvalue EPA**	**pvalue PA**	**padj EPA**	**padj PA**	**Direction Same**
CD96	ENSG00000153283	3	-0.419	-0.210	6.55E-08	1.04E-04	7.70E-06	4.30E-02	Yes Down
TNRC18	ENSG00000182095	7	0.355	0.343	1.24E-04	7.56E-05	3.53E-03	3.50E-02	Yes Up
PLEKHG4	ENSG00000196155	16	-0.417	0.305	1.28E-03	2.22E-05	2.01E-02	1.60E-02	No EPA Down PA Up
COL18A1	ENSG00000182871	21	-0.565	0.507	1.96E-03	2.49E-05	2.76E-02	1.70E-02	Yes Up

g.iv

**Overlapping Genes OA vs PA**									
**Overlapping Genes**	**ensembl**	**chr**	**log2FC OA**	**log2FC PA**	**pvalue OA**	**pvalue PA**	**padj OA**	**padj PA**	**Direction Same**
RNF145	ENSG00000145860	5	0.638	-0.287	7.35E-21	2.81E-05	3.17E-17	1.73E-02	No OA Up PA Down
C1orf162	ENSG00000143110	1	0.522	0.464	3.78E-13	1.83E-15	9.78E-10	4.76E-12	Yes Up
LPCAT1	ENSG00000111684	12	0.473	0.690	1.35E-12	2.26E-10	2.59E-09	4.89E-07	Yes Up
TMEM135	ENSG00000166575	11	0.722	0.743	7.39E-07	5.64E-07	6.83E-04	6.65E-04	Yes Up
HMGCR	ENSG00000113161	5	0.369	-0.325	5.52E-06	3.58E-06	3.40E-03	3.28E-03	No OA Up PA Down
CIC	ENSG00000079432	19	0.347	0.362	2.53E-05	7.28E-05	1.18E-02	3.50E-02	Yes Up
KLF10	ENSG00000155090	8	1.297	1.179	1.68E-04	9.28E-05	4.18E-02	4.01E-02	Yes Up

h

Order	Term	Database	Overlap	p value	Adjusted p value
1	Eukaryotic Translation Elongation Homo sapiens R-HSA-156842	Reactome_2016	44/85	7.47E-31	1.75E-27
2	Cytoplasmic Ribosomal Proteins WP477	WikiPathways_2019_Human	44/86	1.44E-30	1.75E-27
3	Peptide chain elongation Homo sapiens R-HSA-156902	Reactome_2016	43/82	1.75E-30	1.75E-27
4	Viral mRNA Translation Homo sapiens R-HSA-192823	Reactome_2016	43/82	1.75E-30	1.75E-27
5	Selenocysteine synthesis Homo sapiens R-HSA-2408557	Reactome_2016	43/84	6.61E-30	5.30E-27
6	Eukaryotic Translation Termination Homo sapiens R-HSA-72764	Reactome_2016	43/85	1.26E-29	8.42E-27
7	Nonsense Mediated Decay (NMD) independent of the Exon Junction Complex (EJC) Homo sapiens R-HSA-975956	Reactome_2016	43/87	4.43E-29	2.54E-26
8	Formation of a pool of free 40S subunits Homo sapiens R-HSA-72689	Reactome_2016	43/94	2.60E-27	1.30E-24
9	Selenoamino acid metabolism Homo sapiens R-HSA-2408522	Reactome_2016	43/96	7.64E-27	3.22E-24
10	Cytoplasmic ribosomal proteins	BioPlanet_2019	44/101	8.05E-27	3.22E-24
11	SRP-dependent cotranslational protein targeting to membrane Homo sapiens R-HSA-1799339	Reactome_2016	44/104	3.59E-26	1.31E-23
12	3' -UTR-mediated translational regulation Homo sapiens R-HSA-157279	Reactome_2016	43/104	4.17E-25	1.11E-22
13	L13a-mediated translational silencing of Ceruloplasmin expression Homo sapiens R-HSA-156827	Reactome_2016	43/104	4.17E-25	1.11E-22
14	Nonsense-Mediated Decay (NMD) Homo sapiens R-HSA-927802	Reactome_2016	43/104	4.17E-25	1.11E-22
15	Nonsense Mediated Decay (NMD) enhanced by the Exon Junction Complex (EJC) Homo sapiens R-HSA-975957	Reactome_2016	43/104	4.17E-25	1.11E-22
16	GTP hydrolysis and joining of the 60S ribosomal subunit Homo sapiens R-HSA-72706	Reactome_2016	43/105	6.66E-25	1.67E-22
17	Ribosome	KEGG_2019_Human	47/127	7.72E-25	1.82E-22
18	Cap-dependent Translation Initiation Homo sapiens R-HSA-72737	Reactome_2016	43/112	1.48E-23	3.12E-21
19	Eukaryotic Translation Initiation Homo sapiens R-HSA-72613	Reactome_2016	43/112	1.48E-23	3.12E-21
20	Influenza Viral RNA Transcription and Replication Homo sapiens R-HSA-168273	Reactome_2016	44/125	2.73E-22	5.48E-20
21	Translation	BioPlanet_2019	47/145	5.43E-22	1.04E-19
22	Influenza viral RNA transcription and replication	BioPlanet_2019	43/122	7.84E-22	1.43E-19
23	Influenza Life Cycle Homo sapiens R-HSA-168255	Reactome_2016	44/133	4.73E-21	8.24E-19
24	Influenza infection	BioPlanet_2019	44/137	1.80E-20	3.00E-18
25	Translation Homo sapiens R-HSA-72766	Reactome_2016	45/146	4.20E-20	6.73E-18
26	Influenza Infection Homo sapiens R-HSA-168254	Reactome_2016	44/143	1.20E-19	1.85E-17
27	Interleukin-2 signaling pathway	BioPlanet_2019	110/728	3.38E-19	5.02E-17
28	antigen processing and presentation	BioCarta_2015	27/54	7.67E-19	1.10E-16
29	Allograft Rejection	MSigDB_Hallmark_2020	47/172	1.47E-18	2.03E-16
30	Major pathway of rRNA processing in the nucleolus Homo sapiens R-HSA-6791226	Reactome_2016	44/158	8.75E-18	1.17E-15
31	Interferon Gamma Response	MSigDB_Hallmark_2020	47/181	1.39E-17	1.80E-15

32	Antigen processing and presentation	BioPlanet_2019	27/62	6.84E-17	8.56E-15
33	rRNA processing Homo sapiens R-HSA-72312	Reactome_2016	44/170	1.81E-16	2.19E-14
34	Antigen processing and presentation	KEGG_2019_Human	25/58	1.33E-15	1.56E-13
35	Metabolism of amino acids and derivatives Homo sapiens R-HSA-71291	Reactome_2016	53/263	1.36E-14	1.55E-12
36	Infectious disease Homo sapiens R-HSA-5663205	Reactome_2016	60/333	4.57E-14	5.08E-12
37	Cell adhesion molecules (CAMs)	BioPlanet_2019	28/84	6.41E-14	6.94E-12
38	Cell adhesion molecules (CAMs)	KEGG_2019_Human	29/91	8.55E-14	9.01E-12
39	Immunoregulatory interactions between a Lymphoid and a non-Lymphoid cell Homo sapiens R-HSA-198933	Reactome_2016	26/77	3.63E-13	3.73E-11
40	Immunoregulatory interactions between a lymphoid and a non-lymphoid cell	BioPlanet_2019	22/55	4.49E-13	4.50E-11
41	Cap-dependent translation initiation	BioPlanet_2019	25/72	5.04E-13	4.92E-11
42	T cell receptor regulation of apoptosis	BioPlanet_2019	76/529	3.50E-12	3.34E-10
43	Viral myocarditis	KEGG_2019_Human	20/50	5.29E-12	4.93E-10
44	Allograft rejection	BioPlanet_2019	16/31	5.98E-12	5.32E-10
45	Allograft rejection	KEGG_2019_Human	16/31	5.98E-12	5.32E-10
46	Autoimmune thyroid disease	BioPlanet_2019	16/32	1.13E-11	9.61E-10
47	Autoimmune thyroid disease	KEGG_2019_Human	16/32	1.13E-11	9.61E-10
48	Interferon gamma signaling Homo sapiens R-HSA-877300	Reactome_2016	24/76	1.53E-11	1.28E-09
49	Interferon-gamma signaling pathway	BioPlanet_2019	25/84	2.48E-11	2.03E-09
50	Viral myocarditis	BioPlanet_2019	20/55	4.21E-11	3.37E-09
51	Allograft Rejection WP2328	WikiPathways_2019_Human	21/61	4.57E-11	3.59E-09
52	Graft-versus-host disease	BioPlanet_2019	15/30	5.05E-11	3.82E-09
53	Graft-versus-host disease	KEGG_2019_Human	15/30	5.05E-11	3.82E-09
54	Immune System Homo sapiens R-HSA-168256	Reactome_2016	127/1169	9.49E-11	7.04E-09
55	Phosphorylation of CD3 and TCR zeta chains Homo sapiens R-HSA-202427	Reactome_2016	12/19	1.14E-10	8.28E-09
56	Type 1 diabetes mellitus	BioPlanet_2019	15/33	2.83E-10	1.99E-08
57	Type I diabetes mellitus	KEGG_2019_Human	15/33	2.83E-10	1.99E-08
58	Formation of the ternary complex, and subsequently, the 43S complex Homo sapiens R-HSA-72695	Reactome_2016	18/49	3.31E-10	2.29E-08
59	Immune system	BioPlanet_2019	94/789	3.97E-10	2.69E-08
60	Asthma	BioPlanet_2019	11/17	4.81E-10	3.16E-08
61	Asthma	KEGG_2019_Human	11/17	4.81E-10	3.16E-08
62	Acute Phase in Atopic Dermatitis	Elsevier_Pathway_Collection	14/30	7.29E-10	4.71E-08
63	CD8+ Naive T-cell -> CD4+ Naive T-cell Surface Expression Markers	Elsevier_Pathway_Collection	15/35	7.87E-10	5.01E-08
64	Generation of second messenger molecules	BioPlanet_2019	13/27	1.85E-09	1.16E-07
65	Generation of second messenger molecules Homo sapiens R-HSA-202433	Reactome_2016	13/28	3.25E-09	2.00E-07

66	Activation of mRNA upon binding of the cap-binding complex and eIFs, and subsequent binding to 43S	BioPlanet_2019	18/56	4.02E-09	2.37E-07
67	Ribosomal scanning and start codon recognition Homo sapiens R-HSA-72702	Reactome_2016	18/56	4.02E-09	2.37E-07
68	Translation initiation complex formation Homo sapiens R-HSA-72649	Reactome_2016	18/56	4.02E-09	2.37E-07
69	Translocation of ZAP-70 to Immunological synapse Homo sapiens R-HSA-202430	Reactome_2016	10/16	5.05E-09	2.93E-07
70	Activation of the mRNA upon binding of the cap-binding complex and eIFs, and subsequent binding to 43S Homo sapiens R-HSA-72662	Reactome_2016	18/57	5.53E-09	3.12E-07
71	Hematopoietic cell lineage	KEGG_2019_Human	20/70	5.53E-09	3.12E-07
72	Interferon Signaling Homo sapiens R-HSA-913531	Reactome_2016	32/164	6.47E-09	3.60E-07
73	Disease	BioPlanet_2019	70/553	7.92E-09	4.35E-07
74	Interferon Alpha Response	MSigDB_Hallmark_2020	23/93	8.59E-09	4.65E-07
75	Natural Killer Cell Activation	Elsevier_Pathway_Collection	19/65	8.81E-09	4.70E-07
76	Leptin influence on immune response	BioPlanet_2019	21/79	9.58E-09	5.05E-07
77	Interferon signaling	BioPlanet_2019	29/143	1.35E-08	7.05E-07
78	MEF2D role in T cell apoptosis	BioPlanet_2019	13/31	1.50E-08	7.71E-07
79	Th1 and Th2 cell differentiation	KEGG_2019_Human	21/81	1.56E-08	7.90E-07
80	Protein metabolism	BioPlanet_2019	53/379	2.24E-08	1.12E-06
81	T-lymphoid precursor cell -> CD8+ Naive T-cell Surface Expression Markers	Elsevier_Pathway_Collection	16/49	2.30E-08	1.14E-06
82	Proteins Involved in Diabetes Mellitus Type 1	Elsevier_Pathway_Collection	17/56	2.87E-08	1.40E-06
83	Myeloblast -> Neutrophil Surface Expression Markers	Elsevier_Pathway_Collection	16/50	3.19E-08	1.54E-06
84	Cytokine Signaling in Immune system Homo sapiens R-HSA-1280215	Reactome_2016	62/483	3.68E-08	1.75E-06
85	Staphylococcus aureus infection	KEGG_2019_Human	13/34	5.67E-08	2.67E-06
86	Th2-Cell Differentiation	Elsevier_Pathway_Collection	16/52	5.96E-08	2.71E-06
87	Th2-Cell Function in Ulcerative Colitis	Elsevier_Pathway_Collection	16/52	5.96E-08	2.71E-06
88	Th2-Cell Response in Asthma	Elsevier_Pathway_Collection	16/52	5.96E-08	2.71E-06
89	PD-1 signaling	BioPlanet_2019	11/24	6.46E-08	2.91E-06
90	Proteins Involved in Rheumatoid Arthritis	Elsevier_Pathway_Collection	17/59	6.82E-08	3.03E-06
91	Immune system signaling by interferons, interleukins, prolactin, and growth hormones	BioPlanet_2019	38/239	8.66E-08	3.81E-06
92	IL-2/STAT5 Signaling	MSigDB_Hallmark_2020	31/173	8.88E-08	3.87E-06
93	T helper cell surface molecules	BioPlanet_2019	8/12	9.17E-08	3.95E-06
94	Immune System Activation in Hyperthyroidism	Elsevier_Pathway_Collection	14/41	9.26E-08	3.95E-06
95	Endosomal/vacuolar pathway	BioPlanet_2019	7/9	1.20E-07	5.08E-06
96	Th2-Cells Function in Systemic Scleroderma	Elsevier_Pathway_Collection	16/55	1.43E-07	5.98E-06
97	PD-1 signaling Homo sapiens R-HSA-389948	Reactome_2016	10/21	1.67E-07	6.84E-06
98	Inflammatory bowel disease (IBD)	KEGG_2019_Human	15/49	1.67E-07	6.84E-06
99	Proteins Involved in Myocarditis	Elsevier_Pathway_Collection	25/125	1.82E-07	7.36E-06

100	Phagosome	BioPlanet_2019	23/112	3.43E-07	1.36E-05
101	Phagosome	KEGG_2019_Human	23/112	3.43E-07	1.36E-05
102	Peripheral Tolerance to Autoantigens Recession in Diabetes Mellitus Type 1	Elsevier_Pathway_Collection	13/39	3.70E-07	1.45E-05
103	Adhesion and diapedesis of lymphocytes	BioPlanet_2019	7/10	3.80E-07	1.45E-05
104	Monocyte and its surface molecules	BioPlanet_2019	7/10	3.80E-07	1.45E-05
105	Endosomal/Vacuolar pathway Homo sapiens R-HSA-1236977	Reactome_2016	7/10	3.80E-07	1.45E-05
106	Rheumatoid arthritis	KEGG_2019_Human	17/66	4.09E-07	1.54E-05
107	Atopic Dermatitis	Elsevier_Pathway_Collection	15/53	5.25E-07	1.97E-05
108	T-Cell Maturation (Hypothesis)	Elsevier_Pathway_Collection	16/61	6.86E-07	2.55E-05
109	MHC1 Causes Antigen Presentation Failure in Cancer Immune Escape	Elsevier_Pathway_Collection	10/24	7.80E-07	2.87E-05
110	lck and fyn tyrosine kinases in initiation of tcr activation	BioCarta_2015	7/11	9.87E-07	3.53E-05
111	Lck and Fyn tyrosine kinases in initiation of T cell receptor activation	BioPlanet_2019	7/11	9.87E-07	3.53E-05
112	Genes with Mutations in Cancer Immune Escape	Elsevier_Pathway_Collection	7/11	9.87E-07	3.53E-05
113	Intestinal immune network for IgA production	BioPlanet_2019	12/36	1.05E-06	3.69E-05
114	Intestinal immune network for IgA production	KEGG_2019_Human	12/36	1.05E-06	3.69E-05
115	CD8+ T-Cell Response in Celiac Disease	Elsevier_Pathway_Collection	16/63	1.10E-06	3.84E-05
116	Th17 cell differentiation	KEGG_2019_Human	20/94	1.11E-06	3.84E-05
117	TCR signaling Homo sapiens R-HSA-202403	Reactome_2016	22/111	1.17E-06	4.00E-05
118	Epstein-Barr virus infection	KEGG_2019_Human	29/177	1.61E-06	5.47E-05
119	Inflammatory Response	MSigDB_Hallmark_2020	25/140	1.70E-06	5.72E-05
120	Adaptive immune system	BioPlanet_2019	59/503	1.79E-06	5.97E-05
121	Interleukin-12-mediated signaling events	BioPlanet_2019	15/58	1.86E-06	6.17E-05
122	CD4+T-Cell Response in Celiac Disease	Elsevier_Pathway_Collection	18/81	1.97E-06	6.41E-05
123	T-Cell Cytotoxic Response against Melanocytes in Vitiligo	Elsevier_Pathway_Collection	18/81	1.97E-06	6.41E-05
124	Primary immunodeficiency	BioPlanet_2019	11/32	2.10E-06	6.79E-05
125	Cytokine-cytokine receptor interaction	KEGG_2019_Human	26/151	2.16E-06	6.91E-05
126	Complement	MSigDB_Hallmark_2020	26/152	2.45E-06	7.79E-05
127	Proteins with Altered Expression in Cancer Immune Escape	Elsevier_Pathway_Collection	12/39	2.75E-06	8.69E-05
128	role of mef2d in t-cell apoptosis	BioCarta_2015	10/27	2.82E-06	8.84E-05
129	Natural Killer Cell Precursor -> Natural Killer Cell Surface Expression Markers	Elsevier_Pathway_Collection	15/60	2.96E-06	9.17E-05
130	Primary immunodeficiency	KEGG_2019_Human	11/33	2.98E-06	9.17E-05
131	Low-Density Lipoproteins and Chemokines in Atherosclerosis	Elsevier_Pathway_Collection	8/17	3.39E-06	1.04E-04
132	Adaptive Immune System Homo sapiens R-HSA-1280218	Reactome_2016	67/610	3.56E-06	1.08E-04
133	Interleukin-17 signaling pathway	BioPlanet_2019	6/9	4.28E-06	1.29E-04
134	Interleukin-4 regulation of apoptosis	BioPlanet_2019	29/186	4.49E-06	1.34E-04

135	CTL mediated immune response against target cells	BioPlanet_2019	7/13	4.59E-06	1.36E-04
136	Interferon alpha/beta signaling Homo sapiens R-HSA-909733	Reactome_2016	14/55	5.04E-06	1.48E-04
137	Disease Homo sapiens R-HSA-1643685	Reactome_2016	64/580	5.16E-06	1.51E-04
138	Proteins Involved in Age-Related Macular Degeneration	Elsevier_Pathway_Collection	19/95	5.44E-06	1.58E-04
139	Proteins Involved in Multiple Sclerosis	Elsevier_Pathway_Collection	11/35	5.72E-06	1.65E-04
140	IL12-mediated signaling events Homo sapiens 7acdea19-6193-11e5-8ac5-06603eb7f303	NCI-Nature_2016	14/56	6.34E-06	1.81E-04
141	CD4+ T-Cell Function Decline in HIV	Elsevier_Pathway_Collection	13/49	6.68E-06	1.90E-04
142	Systemic lupus erythematosus	KEGG_2019_Human	11/36	7.77E-06	2.19E-04
143	Leukocyte Adhesion to Endothelial Cell	Elsevier_Pathway_Collection	9/24	8.00E-06	2.22E-04
144	Antigen Presentation: Folding, assembly and peptide loading of class I MHC Homo sapiens R-HSA-983170	Reactome_2016	9/24	8.00E-06	2.22E-04
145	Costimulation by the CD28 family	BioPlanet_2019	15/65	8.61E-06	2.38E-04
146	Systemic lupus erythematosus	BioPlanet_2019	11/37	1.04E-05	2.86E-04
147	Gluten Impact on Neuronal System (Hypothesis)	Elsevier_Pathway_Collection	12/44	1.11E-05	3.03E-04
148	Leishmaniasis	KEGG_2019_Human	14/59	1.22E-05	3.30E-04
149	Costimulation by the CD28 family Homo sapiens R-HSA-388841	Reactome_2016	15/67	1.28E-05	3.43E-04
150	Interferon alpha/beta signaling	BioPlanet_2019	13/52	1.36E-05	3.62E-04
151	Antigen processing: cross presentation	BioPlanet_2019	16/76	1.52E-05	4.02E-04
152	B-Cell Activation in Crohn's Disease	Elsevier_Pathway_Collection	9/26	1.71E-05	4.50E-04
153	Antigen processing-Cross presentation Homo sapiens R-HSA-1236975	Reactome_2016	16/77	1.81E-05	4.73E-04
154	Hematopoietic cell lineage	BioPlanet_2019	14/61	1.83E-05	4.77E-04
155	TYROBP Causal Network WP3945	WikiPathways_2019_Human	13/54	2.11E-05	5.45E-04
156	non-Suppressive Treg-Cell in Diabetes Mellitus Type 1	Elsevier_Pathway_Collection	12/47	2.31E-05	5.94E-04
157	Proteins Involved in Glomerulonephritis	Elsevier_Pathway_Collection	16/79	2.53E-05	6.46E-04
158	Inflammasomes	BioPlanet_2019	7/16	2.59E-05	6.48E-04
159	Inflammasomes Homo sapiens R-HSA-622312	Reactome_2016	7/16	2.59E-05	6.48E-04
160	Treg-Cell Differentiation	Elsevier_Pathway_Collection	13/55	2.60E-05	6.48E-04
161	Treg-Cell Function in Diabetes Mellitus Type 1	Elsevier_Pathway_Collection	13/55	2.60E-05	6.48E-04
162	Cytokine-cytokine receptor interaction	BioPlanet_2019	23/143	2.62E-05	6.48E-04
163	Hemapoietic Stem Cell -> Lymphoid Multipotent Progenitor Surface Expression Markers	Elsevier_Pathway_Collection	11/41	3.06E-05	7.53E-04
164	Human T-cell leukemia virus 1 infection	KEGG_2019_Human	28/195	3.16E-05	7.72E-04
165	Thymic stromal lymphopoietin (TSLP) pathway	BioPlanet_2019	14/64	3.27E-05	7.86E-04
166	ER-Phagosome pathway Homo sapiens R-HSA-1236974	Reactome_2016	14/64	3.27E-05	7.86E-04
167	Innate Immune System Homo sapiens R-HSA-168249	Reactome_2016	61/577	3.28E-05	7.86E-04
168	Human immunodeficiency virus 1 infection	KEGG_2019_Human	26/175	3.33E-05	7.93E-04
169	T-Cell Receptor -> CREBBP Signaling	Elsevier_Pathway_Collection	8/22	3.37E-05	7.99E-04

170	Th2-Cell Function in Systemic Lupus Erythematosus	Elsevier_Pathway_Collection	12/49	3.64E-05	8.58E-04
171	Natural Killer Cell Activation through Integrins and non-ITAM-Containing Receptors	Elsevier_Pathway_Collection	11/42	3.92E-05	9.18E-04
172	Proteins Involved in Dental Caries	Elsevier_Pathway_Collection	7/17	4.17E-05	9.71E-04
173	Cell surface interactions at the vascular wall Homo sapiens R-HSA-202733	Reactome_2016	15/74	4.47E-05	1.04E-03
174	Natural Killer Cell in Diabetes Mellitus Type 1	Elsevier_Pathway_Collection	12/50	4.52E-05	1.04E-03
175	Leishmaniasis	BioPlanet_2019	13/58	4.76E-05	1.09E-03
176	T-Cell Receptor -> NFATC Signaling	Elsevier_Pathway_Collection	8/23	4.89E-05	1.11E-03
177	Apoptotic Keratinocytes Clearance Recession in Systemic Lupus Erythematosus	Elsevier_Pathway_Collection	10/36	5.08E-05	1.15E-03
178	Ebola Virus Pathway on Host WP4217	WikiPathways_2019_Human	19/111	5.47E-05	1.23E-03
179	Natural killer cell mediated cytotoxicity	KEGG_2019_Human	17/93	5.71E-05	1.28E-03
180	Downstream TCR signaling Homo sapiens R-HSA-202424	Reactome_2016	17/94	6.57E-05	1.46E-03
181	Adhesion and diapedesis of granulocytes	BioPlanet_2019	6/13	7.04E-05	1.55E-03
182	Lipoxins and Resolvins in Inflammation Resolution	Elsevier_Pathway_Collection	6/13	7.04E-05	1.55E-03
183	Interleukin-1-beta (IL-1b) processing pathway	BioPlanet_2019	4/5	7.34E-05	1.61E-03
184	Cell surface interactions at the vascular wall	BioPlanet_2019	14/69	7.90E-05	1.72E-03
185	Natural Killer Cell Activation through ITAM-Containing Receptors	Elsevier_Pathway_Collection	12/53	8.35E-05	1.81E-03
186	Tuberculosis	KEGG_2019_Human	21/135	9.61E-05	2.06E-03
187	activation of csk by camp-dependent protein kinase inhibits signaling through the t cell receptor	BioCarta_2015	8/25	9.63E-05	2.06E-03
188	Chemokines Signaling in Atherosclerosis	Elsevier_Pathway_Collection	5/9	9.82E-05	2.08E-03
189	MHC2-Mediated Antigen Presentation	Elsevier_Pathway_Collection	5/9	9.82E-05	2.08E-03
190	Proteins Involved in Ulcerative Colitis	Elsevier_Pathway_Collection	16/88	1.00E-04	2.11E-03
191	Th17-Cell Differentiation	Elsevier_Pathway_Collection	12/54	1.01E-04	2.11E-03
192	Treg-Cell Activation in Diabetes Mellitus	Elsevier_Pathway_Collection	12/54	1.01E-04	2.11E-03
193	Proteins Involved in Systemic Lupus Erythematosus	Elsevier_Pathway_Collection	11/47	1.20E-04	2.48E-03
194	TLR2 Signaling in Treg-Cell in Type 1 Diabetes (Animal Model)	Elsevier_Pathway_Collection	12/55	1.22E-04	2.53E-03
195	GPCRs, Class A Rhodopsin-like WP455	WikiPathways_2019_Human	14/72	1.28E-04	2.63E-03
196	Natural killer cell-mediated cytotoxicity	BioPlanet_2019	17/99	1.28E-04	2.63E-03
197	Proteins Involved in Psoriatic Arthritis	Elsevier_Pathway_Collection	10/40	1.35E-04	2.74E-03
198	Th17-Cell and Th1 Immune Responsein Psoriatic Arthritis	Elsevier_Pathway_Collection	12/56	1.47E-04	2.96E-03
199	Th17-Cell Differentiation in Asthma	Elsevier_Pathway_Collection	12/56	1.47E-04	2.96E-03
200	CD8+ T-Cell Activation	Elsevier_Pathway_Collection	12/57	1.76E-04	3.52E-03
201	Th1-Cells Activation and Proliferation in Atherosclerosis	Elsevier_Pathway_Collection	13/66	1.94E-04	3.86E-03
202	Natural Killer T-Cell Roles in Diabetes Mellitus Type 1	Elsevier_Pathway_Collection	12/58	2.09E-04	4.15E-03
203	Acute Cytotoxic CD8+T-Cell Response against Melanocytes	Elsevier_Pathway_Collection	11/50	2.16E-04	4.26E-03
204	Binding of chemokines to chemokine receptors	BioPlanet_2019	8/28	2.34E-04	4.57E-03

205	T-Cell Positive Selection and Neglect Induced Death	Elsevier_Pathway_Collection	8/28	2.34E-04	4.57E-03
206	Prostaglandin biosynthesis and regulation	BioPlanet_2019	6/16	2.79E-04	5.43E-03
207	Chemokine receptors bind chemokines Homo sapiens R-HSA-380108	Reactome_2016	8/29	3.06E-04	5.92E-03
208	T cell receptor signaling in naive CD8+ T cells	BioPlanet_2019	11/52	3.12E-04	5.97E-03
209	TCR signaling in naive CD8+ T cells Homo sapiens 15a017bb-6196-11e5-8ac5-06603eb7f303	NCI-Nature_2016	11/52	3.12E-04	5.97E-03
210	Myoblast -> Neutrophil Surface Expression Markers	Elsevier_Pathway_Collection	10/44	3.14E-04	6.00E-03
211	B lymphocyte cell surface molecules	BioPlanet_2019	5/11	3.24E-04	6.09E-03
212	Cells and molecules involved in local acute inflammatory response	BioPlanet_2019	5/11	3.24E-04	6.09E-03
213	Cells and Molecules involved in local acute inflammatory response WP4493	WikiPathways_2019_Human	5/11	3.24E-04	6.09E-03
214	Metabolism of proteins Homo sapiens R-HSA-392499	Reactome_2016	76/824	3.40E-04	6.36E-03
215	Class A GPCRs (rhodopsin-like)	BioPlanet_2019	13/70	3.57E-04	6.65E-03
216	CD8/T cell receptor downstream pathway	BioPlanet_2019	11/53	3.71E-04	6.85E-03
217	Downstream signaling in naive CD8+ T cells Homo sapiens 92180cef-6191-11e5-8ac5-06603eb7f303	NCI-Nature_2016	11/53	3.71E-04	6.85E-03
218	Leukocyte Migration toward the Endothelial Cell in Ulcerative Colitis	Elsevier_Pathway_Collection	7/23	3.77E-04	6.93E-03
219	Natural Killer Cell Inhibitory Receptor Signaling	Elsevier_Pathway_Collection	10/45	3.82E-04	6.99E-03
220	Inhibition of T cell receptor signaling by activated Csk	BioPlanet_2019	6/17	4.08E-04	7.44E-03
221	Proteins Involved in Atherosclerosis	Elsevier_Pathway_Collection	17/109	4.23E-04	7.66E-03
222	TNFR2 non-canonical NF-kB pathway Homo sapiens R-HSA-5668541	Reactome_2016	15/90	4.41E-04	7.96E-03
223	RANKL regulation of apoptosis and immune response	BioPlanet_2019	12/63	4.69E-04	8.36E-03
224	T cell receptor signaling in naive CD4+ T cells	BioPlanet_2019	12/63	4.69E-04	8.36E-03
225	Lymphocyte Mediated Myocardial Injury in Myocarditis	Elsevier_Pathway_Collection	12/63	4.69E-04	8.36E-03
226	T-Cell Receptor -> AP-1 Signaling	Elsevier_Pathway_Collection	8/31	5.02E-04	8.82E-03
227	Th1-Cell Activation in Crohn's Disease	Elsevier_Pathway_Collection	8/31	5.02E-04	8.82E-03
228	Th17-Cell Activation in Crohn's Disease	Elsevier_Pathway_Collection	8/31	5.02E-04	8.82E-03
229	Selective expression of chemokine receptors during T-cell polarization WP4494	WikiPathways_2019_Human	7/24	5.04E-04	8.82E-03
230	GPVI-mediated activation cascade Homo sapiens R-HSA-114604	Reactome_2016	10/47	5.53E-04	9.64E-03
231	Th1/Th2 differentiation pathway	BioPlanet_2019	6/18	5.80E-04	1.01E-02
232	Pertussis	KEGG_2019_Human	11/56	6.11E-04	1.05E-02
233	Prostaglandin Synthesis and Regulation WP98	WikiPathways_2019_Human	7/25	6.62E-04	1.14E-02
234	Interleukin-12/STAT4 pathway	BioPlanet_2019	9/40	6.76E-04	1.16E-02
235	T-Cell antigen Receptor (TCR) Signaling Pathway WP69	WikiPathways_2019_Human	14/84	6.79E-04	1.16E-02
236	Vitiligo	Elsevier_Pathway_Collection	12/66	7.29E-04	1.24E-02
237	Proteins Involved in Endometriosis	Elsevier_Pathway_Collection	21/157	7.95E-04	1.34E-02
238	T-Cell Receptor -> STAT Signaling	Elsevier_Pathway_Collection	6/19	8.04E-04	1.35E-02
239	il 4 signaling pathway	BioCarta_2015	5/13	8.12E-04	1.36E-02

240	T-Cell antigen Receptor (TCR) pathway during Staphylococcus aureus infection WP3863	WikiPathways_2019_Human	11/58	8.33E-04	1.39E-02
241	Hemostasis Homo sapiens R-HSA-109582	Reactome_2016	40/380	8.45E-04	1.40E-02
242	Bystander B cell activation	BioPlanet_2019	4/8	8.82E-04	1.45E-02
243	Calcium/calmodulin-dependent protein kinase activation	BioPlanet_2019	4/8	8.82E-04	1.45E-02
244	Atopic Dermatitis Onset	Elsevier_Pathway_Collection	10/50	9.26E-04	1.51E-02
245	T-Cell Receptor Signaling	Elsevier_Pathway_Collection	10/50	9.26E-04	1.51E-02
246	Eosinophils in the chemokine network of allergy	BioPlanet_2019	3/4	9.42E-04	1.53E-02
247	Regulation of Insulin-like Growth Factor (IGF) transport and uptake by Insulin-like Growth Factor Binding Proteins (IGFBPs) Homo sapiens R-HSA-381426	Reactome_2016	3/4	9.42E-04	1.53E-02
248	Th17-Cell Function in Systemic Lupus Erythematosus	Elsevier_Pathway_Collection	9/42	9.86E-04	1.59E-02
249	Class A/1 (Rhodopsin-like receptors) Homo sapiens R-HSA-373076	Reactome_2016	16/107	9.90E-04	1.59E-02
250	GPCR ligand binding	BioPlanet_2019	19/139	1.07E-03	1.71E-02
251	CD8+ T-Cell Action Impairment in HIV Type 1 Infection	Elsevier_Pathway_Collection	7/27	1.10E-03	1.74E-02
252	Macrophage Function in Diabetes Mellitus Type 1	Elsevier_Pathway_Collection	7/27	1.10E-03	1.74E-02
253	Treg Cells Promote Immunosuppression in Cancer Immune Escape	Elsevier_Pathway_Collection	9/43	1.18E-03	1.87E-02
254	Phototransduction	BioPlanet_2019	5/14	1.20E-03	1.89E-02
255	Toxoplasmosis	KEGG_2019_Human	15/99	1.23E-03	1.93E-02
256	Spinal Cord Injury WP2431	WikiPathways_2019_Human	12/70	1.25E-03	1.96E-02
257	Proteins Involved in HIV Type 1 Infection	Elsevier_Pathway_Collection	11/61	1.29E-03	2.01E-02
258	Megakaryocyte -> Platelet Precursor Cell Surface Expression Markers	Elsevier_Pathway_Collection	7/28	1.38E-03	2.15E-02
259	Neutrophil Chemotaxis	Elsevier_Pathway_Collection	9/44	1.40E-03	2.17E-02
260	NOD-like receptor signaling pathway	KEGG_2019_Human	18/132	1.47E-03	2.26E-02
261	Peptide G-protein coupled receptors	BioPlanet_2019	11/62	1.48E-03	2.26E-02
262	TCR signaling in naive CD4+ T cells Homo sapiens 0c2862fa-6196-11e5-8ac5-06603eb7f303	NCI-Nature_2016	11/62	1.48E-03	2.26E-02
263	HIV-induced T cell apoptosis	BioPlanet_2019	4/9	1.51E-03	2.29E-02
264	Platelet adhesion to exposed collagen	BioPlanet_2019	4/9	1.51E-03	2.29E-02
265	TNF-alpha effects on cytokine activity, cell motility, and apoptosis	BioPlanet_2019	14/91	1.52E-03	2.30E-02
266	Signaling by GPCR Homo sapiens R-HSA-372790	Reactome_2016	47/482	1.59E-03	2.40E-02
267	Proteins Involved in B-Cell Chronic Lymphocytic Leukemia	Elsevier_Pathway_Collection	9/45	1.66E-03	2.48E-02
268	Peptide ligand-binding receptors Homo sapiens R-HSA-375276	Reactome_2016	11/63	1.69E-03	2.52E-02
269	MHC class II antigen presentation	BioPlanet_2019	14/92	1.70E-03	2.52E-02
270	MHC class II antigen presentation Homo sapiens R-HSA-2132295	Reactome_2016	14/92	1.70E-03	2.52E-02
271	Platelet activation, signaling and aggregation Homo sapiens R-HSA-76002	Reactome_2016	22/178	1.73E-03	2.56E-02
272	Proteins Involved in Helicobacter Infections	Elsevier_Pathway_Collection	8/37	1.76E-03	2.59E-02
273	Viral Acute Myocarditis WP4298	WikiPathways_2019_Human	12/73	1.82E-03	2.67E-02

274	Selective expression of chemokine receptors during T-cell polarization	BioPlanet_2019	6/22	1.88E-03	2.75E-02
275	Type II interferon signaling (interferon-gamma)	BioPlanet_2019	7/30	2.13E-03	3.07E-02
276	Proteins Involved in Systemic Scleroderma	Elsevier_Pathway_Collection	7/30	2.13E-03	3.07E-02
277	IL12 signaling mediated by STAT4 Homo sapiens 72cf19b8-6193-11e5-8ac5-06603eb7f303	NCI-Nature_2016	7/30	2.13E-03	3.07E-02
278	Type II interferon signaling (IFNG) WP619	WikiPathways_2019_Human	7/30	2.13E-03	3.07E-02
279	Proteins Involved in Bronchiectasis	Elsevier_Pathway_Collection	3/5	2.24E-03	3.22E-02
280	T-Cell Cytotoxic Mediated Cell Death	Elsevier_Pathway_Collection	9/47	2.28E-03	3.26E-02
281	NO2-dependent IL-12 pathway in NK cells	BioPlanet_2019	5/16	2.35E-03	3.36E-02
282	Ras activation upon calcium influx through NMDA receptor	BioPlanet_2019	4/10	2.39E-03	3.36E-02
283	IL2R -> STAT Signaling	Elsevier_Pathway_Collection	4/10	2.39E-03	3.36E-02
284	MHC1-Mediated Antigen Presentation	Elsevier_Pathway_Collection	4/10	2.39E-03	3.36E-02
285	Platelet Adhesion to exposed collagen Homo sapiens R-HSA-75892	Reactome_2016	4/10	2.39E-03	3.36E-02
286	CD8+ T-Cell Response to Self-Determinants in Diabetes	Elsevier_Pathway_Collection	6/23	2.41E-03	3.38E-02
287	T-Cell Receptor -> NF-kB Signaling	Elsevier_Pathway_Collection	8/39	2.51E-03	3.51E-02
288	NCAM signaling for neurite out-growth Homo sapiens R-HSA-375165	Reactome_2016	23/195	2.55E-03	3.55E-02
289	Epithelial Mesenchymal Transition	MSigDB_Hallmark_2020	15/107	2.71E-03	3.76E-02
290	Platelet Activation via Adhesion Molecules	Elsevier_Pathway_Collection	10/58	2.99E-03	4.12E-02
291	Proteins Involved in Spontaneous Abortion	Elsevier_Pathway_Collection	10/58	2.99E-03	4.12E-02
292	Inflammatory response pathway	BioPlanet_2019	6/24	3.05E-03	4.15E-02
293	Inflammatory Response Pathway WP453	WikiPathways_2019_Human	6/24	3.05E-03	4.15E-02
294	Peptide GPCRs WP24	WikiPathways_2019_Human	6/24	3.05E-03	4.15E-02
295	Proteins Involved in Chronic Obstructive Pulmonary Disease	Elsevier_Pathway_Collection	14/98	3.11E-03	4.22E-02
296	Human cytomegalovirus infection	KEGG_2019_Human	21/175	3.13E-03	4.24E-02
297	T-Cell Receptor -> ATF/CREB Signaling	Elsevier_Pathway_Collection	7/32	3.16E-03	4.26E-02
298	KRAS Signaling Up	MSigDB_Hallmark_2020	17/131	3.36E-03	4.51E-02
299	Proteins Involved in Astrocytoma	Elsevier_Pathway_Collection	10/59	3.41E-03	4.56E-02
300	Proteins Involved in Osteoarthritis	Elsevier_Pathway_Collection	8/41	3.49E-03	4.66E-02
301	CD2 -> NFATC1 Signaling	Elsevier_Pathway_Collection	4/11	3.57E-03	4.75E-02

Table 1h continued

Order	Genes	Upregulated	Downregulated	Gro

1	*RPS24, RPS25, RPS27A, RPL35A, RPS12, RPS15A, RPL30, RPL34, RPS6, RPL23, RPS29, RPL31, RPS7, RPS21, RPS20, RPS23, RPS8, RPS13, RPL21, RPL37, RPL23A, RPL11, RPS27, RPL32, RPL27, RPS3A, EEF1B2, RPL39, RPL38, RPL12, RPL4, RPL10A, RPL41, RPL37A, RPL9, RPL5, RPL24, RPL6, RPL19, UBA52, RPS11, RPL7, RPS18, RPS14*	0	44	1
2	*RPS24, RPS25, RPS27A, RPL35A, RPS12, RPS15A, RPL30, RPL34, RPS6, RPL23, RPS29, RPL31, RPS7, RPS21, RPS20, RPS23, RPS8, RPS13, RPL21, RPL37, RPL23A, RPL11, RPS27, RPL32, RPL27, RPS3A, RPL39, RPL38, RPL12, RPL4, RPL10A, RPL41, RPL37A, RPL9, RPL5, RPL24, RPL6, RPL19, UBA52, RPS11, RPL7, RPS18, RPS6KA1, RPS14*	0	44	1
3	*RPS24, RPS25, RPS27A, RPL35A, RPS12, RPS15A, RPL30, RPL34, RPS6, RPL23, RPS29, RPL31, RPS7, RPS21, RPS20, RPS23, RPS8, RPS13, RPL21, RPL37, RPL23A, RPL11, RPS27, RPL32, RPL27, RPS3A, RPL39, RPL38, RPL12, RPL4, RPL10A, RPL41, RPL37A, RPL9, RPL5, RPL24, RPL6, RPL19, UBA52, RPS11, RPL7, RPS18, RPS14*	0	43	1
4	*RPS24, RPS25, RPS27A, RPL35A, RPS12, RPS15A, RPL30, RPL34, RPS6, RPL23, RPS29, RPL31, RPS7, RPS21, RPS20, RPS23, RPS8, RPS13, RPL21, RPL37, RPL23A, RPL11, RPS27, RPL32, RPL27, RPS3A, RPL39, RPL38, RPL12, RPL4, RPL10A, RPL41, RPL37A, RPL9, RPL5, RPL24, RPL6, RPL19, UBA52, RPS11, RPL7, RPS18, RPS14*	0	43	1
5	*RPS24, RPS25, RPS27A, RPL35A, RPS12, RPS15A, RPL30, RPL34, RPS6, RPL23, RPS29, RPL31, RPS7, RPS21, RPS20, RPS23, RPS8, RPS13, RPL21, RPL37, RPL23A, RPL11, RPS27, RPL32, RPL27, RPS3A, RPL39, RPL38, RPL12, RPL4, RPL10A, RPL41, RPL37A, RPL9, RPL5, RPL24, RPL6, RPL19, UBA52, RPS11, RPL7, RPS18, RPS14*	0	43	1
6	*RPS24, RPS25, RPS27A, RPL35A, RPS12, RPS15A, RPL30, RPL34, RPS6, RPL23, RPS29, RPL31, RPS7, RPS21, RPS20, RPS23, RPS8, RPS13, RPL21, RPL37, RPL23A, RPL11, RPS27, RPL32, RPL27, RPS3A, RPL39, RPL38, RPL12, RPL4, RPL10A, RPL41, RPL37A, RPL9, RPL5, RPL24, RPL6, RPL19, UBA52, RPS11, RPL7, RPS18, RPS14*	0	43	1
7	*RPS24, RPS25, RPS27A, RPL35A, RPS12, RPS15A, RPL30, RPL34, RPS6, RPL23, RPS29, RPL31, RPS7, RPS21, RPS20, RPS23, RPS8, RPS13, RPL21, RPL37, RPL23A, RPL11, RPS27, RPL32, RPL27, RPS3A, RPL39, RPL38, RPL12, RPL4, RPL10A, RPL41, RPL37A, RPL9, RPL5, RPL24, RPL6, RPL19, UBA52, RPS11, RPL7, RPS18, RPS14*	0	43	1
8	*RPS24, RPS25, RPS27A, RPL35A, RPS12, RPS15A, RPL30, RPL34, RPS6, RPL23, RPS29, RPL31, RPS7, RPS21, RPS20, RPS23, RPS8, RPS13, RPL21, RPL37, RPL23A, RPL11, RPS27, RPL32, RPL27, RPS3A, RPL39, RPL38, RPL12, RPL4, RPL10A, RPL41, RPL37A, RPL9, RPL5, RPL24, RPL6, RPL19, UBA52, RPS11, RPL7, RPS18, RPS14*	0	43	1
9	*RPS24, RPS25, RPS27A, RPL35A, RPS12, RPS15A, RPL30, RPL34, RPS6, RPL23, RPS29, RPL31, RPS7, RPS21, RPS20, RPS23, RPS8, RPS13, RPL21, RPL37, RPL23A, RPL11, RPS27, RPL32, RPL27, RPS3A, RPL39, RPL38, RPL12, RPL4, RPL10A, RPL41, RPL37A, RPL9, RPL5, RPL24, RPL6, RPL19, UBA52, RPS11, RPL7, RPS18, RPS14*	0	43	1
10	*RPS24, RPS25, RPS27A, RPL35A, RPS12, RPS15A, RPL30, RPL34, RPS6, RPL23, RPS29, RPL31, RPS7, RPS21, RPS20, RPS23, RPS8, RPS13, RPL21, RPL37, RPL23A, RPL11, RPS27, RPL32, RPL27, RPS3A, RPL39, RPL38, RPL12, RPL4, RPL10A, RPL41, RPL37A, RPL9, RPL5, RPL24, RPL6, RPL19, UBA52, RPS11, RPL7, RPS18, RPS6KA1, RPS14*	0	44	1
11	*RPS24, RPS25, RPS27A, RPL35A, RPS12, RPS15A, RPL30, RPL34, RPS6, RPL23, RPS29, RPL31, RPS7, RPS21, RPS20, RPS23, RPS8, RPS13, RPL21, RPL37, RPL23A, RPL11, RPS27, RPL32, RPL27, RPS3A, RPL39, RPL38, RPL12, RPL4, RPL10A, RPL41, RPL37A, RPL9, RPL5, RPL24, RPL6, RPL19, UBA52, RPS11, RPL7, RPS18, SSR2, RPS14*	0	44	1
12	*RPS24, RPS25, RPS27A, RPL35A, RPS12, RPS15A, RPL30, RPL34, RPS6, RPL23, RPS29, RPL31, RPS7, RPS21, RPS20, RPS23, RPS8, RPS13, RPL21, RPL37, RPL23A, RPL11, RPS27, RPL32, RPL27, RPS3A, RPL39, RPL38, RPL12, RPL4, RPL10A, RPL41, RPL37A, RPL9, RPL5, RPL24, RPL6, RPL19, UBA52, RPS11, RPL7, RPS18, RPS14*	0	43	1

13	*RPS24, RPS25, RPS27A, RPL35A, RPS12, RPS15A, RPL30, RPL34, RPS6, RPL23, RPS29, RPL31, RPS7, RPS21, RPS20, RPS23, RPS8, RPS13, RPL21, RPL37, RPL23A, RPL11, RPS27, RPL32, RPL27, RPS3A, RPL39, RPL38, RPL12, RPL4, RPL10A, RPL41, RPL37A, RPL9, RPL5, RPL24, RPL6, RPL19, UBA52, RPS11, RPL7, RPS18, RPS14*	0	43	1
14	*RPS24, RPS25, RPS27A, RPL35A, RPS12, RPS15A, RPL30, RPL34, RPS6, RPL23, RPS29, RPL31, RPS7, RPS21, RPS20, RPS23, RPS8, RPS13, RPL21, RPL37, RPL23A, RPL11, RPS27, RPL32, RPL27, RPS3A, RPL39, RPL38, RPL12, RPL4, RPL10A, RPL41, RPL37A, RPL9, RPL5, RPL24, RPL6, RPL19, UBA52, RPS11, RPL7, RPS18, RPS14*	0	43	1
15	*RPS24, RPS25, RPS27A, RPL35A, RPS12, RPS15A, RPL30, RPL34, RPS6, RPL23, RPS29, RPL31, RPS7, RPS21, RPS20, RPS23, RPS8, RPS13, RPL21, RPL37, RPL23A, RPL11, RPS27, RPL32, RPL27, RPS3A, RPL39, RPL38, RPL12, RPL4, RPL10A, RPL41, RPL37A, RPL9, RPL5, RPL24, RPL6, RPL19, UBA52, RPS11, RPL7, RPS18, RPS14*	0	43	1
16	*RPS24, RPS25, RPS27A, RPL35A, RPS12, RPS15A, RPL30, RPL34, RPS6, RPL23, RPS29, RPL31, RPS7, RPS21, RPS20, RPS23, RPS8, RPS13, RPL21, RPL37, RPL23A, RPL11, RPS27, RPL32, RPL27, RPS3A, RPL39, RPL38, RPL12, RPL4, RPL10A, RPL41, RPL37A, RPL9, RPL5, RPL24, RPL6, RPL19, UBA52, RPS11, RPL7, RPS18, RPS14*	0	43	1
17	*RPS24, RPS25, RPS27A, RPL35A, RPS12, RPS15A, RPL30, RPL34, RPS6, RPL23, RPS29, RPL31, RPS7, RPS21, RPS20, RPS23, RPS8, RPS13, RPL21, MRPS21, RPL37, RPL23A, RPL11, RPS27, RPL32, RPL27, RPS3A, RPL39, RPL38, RPL12, RPL4, RPL22L1, RPL10A, RPL41, RPL37A, RPL9, RPL5, RPL36AL, RPL24, RPL6, RPL19, UBA52, RPS11, MRPL33, RPL7, RPS18, RPS14*	0	47	1
18	*RPS24, RPS25, RPS27A, RPL35A, RPS12, RPS15A, RPL30, RPL34, RPS6, RPL23, RPS29, RPL31, RPS7, RPS21, RPS20, RPS23, RPS8, RPS13, RPL21, RPL37, RPL23A, RPL11, RPS27, RPL32, RPL27, RPS3A, RPL39, RPL38, RPL12, RPL4, RPL10A, RPL41, RPL37A, RPL9, RPL5, RPL24, RPL6, RPL19, UBA52, RPS11, RPL7, RPS18, RPS14*	0	43	1
19	*RPS24, RPS25, RPS27A, RPL35A, RPS12, RPS15A, RPL30, RPL34, RPS6, RPL23, RPS29, RPL31, RPS7, RPS21, RPS20, RPS23, RPS8, RPS13, RPL21, RPL37, RPL23A, RPL11, RPS27, RPL32, RPL27, RPS3A, RPL39, RPL38, RPL12, RPL4, RPL10A, RPL41, RPL37A, RPL9, RPL5, RPL24, RPL6, RPL19, UBA52, RPS11, RPL7, RPS18, RPS14*	0	43	1
20	*RPS24, RPS25, RPS27A, RPL35A, RPS12, RPS15A, RPL30, RPL34, RPS6, RPL23, RPS29, RPL31, RPS7, RPS21, RPS20, RPS23, RPS8, RPS13, RPL21, RPL37, RPL23A, RPL11, RPS27, RPL32, RPL27, RPS3A, RPL39, RPL38, RPL12, RPL4, RPL10A, RPL41, RPL37A, RPL9, RPL5, RPL24, RPL6, RPL19, UBA52, RPS11, NUP37, RPL7, RPS18, RPS14*	0	44	1
21	*RPS24, RPS25, RPS27A, RPL35A, RPS12, RPS15A, RPL30, RPL34, RPS6, RPL23, RPS29, RPL31, RPS7, RPS21, RPS20, RPS23, RPS8, RPS13, RPL21, RPL37, RPL23A, RPL11, RPS27, RPL32, RPL27, RPS3A, EEF1B2, RPL39, RPL38, RPL12, RPL4, RPL22L1, RPL10A, RPL41, RPL37A, RPL9, RPL5, RPL36AL, RPL24, RPL6, RPL19, UBA52, RPS11, RPL7, RPS18, SSR2, RPS14*	0	47	1
22	*RPS24, RPS25, RPS27A, RPL35A, RPS12, RPS15A, RPL30, RPL34, RPS6, RPL23, RPS29, RPL31, RPS7, RPS21, RPS20, RPS23, RPS8, RPS13, RPL21, RPL37, RPL23A, RPL11, RPS27, RPL32, RPL27, RPS3A, RPL39, RPL38, RPL12, RPL4, RPL10A, RPL41, RPL37A, RPL9, RPL5, RPL24, RPL6, RPL19, UBA52, RPS11, RPL7, RPS18, RPS14*	0	43	1
23	*RPS24, RPS25, RPS27A, RPL35A, RPS12, RPS15A, RPL30, RPL34, RPS6, RPL23, RPS29, RPL31, RPS7, RPS21, RPS20, RPS23, RPS8, RPS13, RPL21, RPL37, RPL23A, RPL11, RPS27, RPL32, RPL27, RPS3A, RPL39, RPL38, RPL12, RPL4, RPL10A, RPL41, RPL37A, RPL9, RPL5, RPL24, RPL6, RPL19, UBA52, RPS11, NUP37, RPL7, RPS18, RPS14*	0	44	1
24	*RPS24, RPS25, RPS27A, RPL35A, RPS12, RPS15A, RPL30, RPL34, RPS6, RPL23, RPS29, RPL31, RPS7, RPS21, RPS20, RPS23, RPS8, RPS13, RPL21, RPL37, RPL23A, RPL11, RPS27, RPL32, RPL27, RPS3A, RPL39, RPL38, RPL12, RPL4, RPL10A, RPL41, RPL37A, RPL9, RPL5, RPL24, RPL6, RPL19, UBA52, RPS11, NUP37, RPL7, RPS18, RPS14*	0	44	1

25	*RPS24, RPS25, RPS27A, RPL35A, RPS12, RPS15A, RPL30, RPL34, RPS6, RPL23, RPS29, RPL31, RPS7, RPS21, RPS20, RPS23, RPS8, RPS13, RPL21, RPL37, RPL23A, RPL11, RPS27, RPL32, RPL27, RPS3A, EEF1B2, RPL39, RPL38, RPL12, RPL4, RPL10A, RPL41, RPL37A, RPL9, RPL5, RPL24, RPL6, RPL19, UBA52, RPS11, RPL7, RPS18, SSR2, RPS14*	0	45	1
26	*RPS24, RPS25, RPS27A, RPL35A, RPS12, RPS15A, RPL30, RPL34, RPS6, RPL23, RPS29, RPL31, RPS7, RPS21, RPS20, RPS23, RPS8, RPS13, RPL21, RPL37, RPL23A, RPL11, RPS27, RPL32, RPL27, RPS3A, RPL39, RPL38, RPL12, RPL4, RPL10A, RPL41, RPL37A, RPL9, RPL5, RPL24, RPL6, RPL19, UBA52, RPS11, NUP37, RPL7, RPS18, RPS14*	0	44	1
27	*VIM, BTN3A1, BTN3A2, BTN3A3, SOCS2, GZMK, CISH, CD69, GBP1, CCR2, DPP4, TAP1, TRIB2, GZMA, CD96, IL2RA, RPS6, S100A11, ITGAM, AIF1, CD52, DENND2D, PTGER2, CASP1, RNF144A, NELL2, FCER1G, NCR3, RPL21, S100A4, MEOX1, CCR5, CD40LG, CTSO, ADTRP, GZMM, MAL, VNN2, CD2, FYN, IL2RG, CTSS, APOBEC3G, IL4R, MT2A, CYP1A1, LITAF, VAMP5, RGS10, SELL, RBM3, CCR1, TNFRSF1B, IL2RB, NMT2, KCNN4, BTG1, LAT, HEMGN, PDCD4, MLLT3, CD27, LCP2, NKG7, AHNAK, GNLY, ETS1, ACP5, CX3CR1, RAB33A, TBCD, FLOT1, PDE7A, TNFRSF25, IRF1, IFIT1, CD300A, ITGB2, MDFIC, BCL2, ADAM19, TRIM21, TAGLN2, LTB, CCR7, NLRP1, PTPRC, CTSZ, SORL1, TXK, PARP8, TTN, SLA2, GATA3, SAMHD1, CCL5, AP3M2, IQGAP2, FCGBP, CXCL8, SPN, E2F3, TNFSF10, CAMK2N1, CTSH, SIT1, JAK3, PIM1, PRF1, EOMES*	0	110	2
28	*HLA-DRA, HLA-DPA1, HLA-DPB1, PSMB9, HLA-E, CD74, HLA-DRB1, B2M, TAP1, PSME1, HLA-DRB5, HLA-DQA1, CIITA, HLA-DMB, CD8A, CTSS, PSMB8, HLA-B, HLA-F, CD4, HLA-DQB1, HLA-DOA, PSME2, TAP2, HLA-A, HLA-C, HSPA5*	0	27	3
29	*HLA-DRA, HLA-E, CD74, B2M, CCR2, TAP1, GZMA, CD96, IL2RA, KRT1, HLA-DQA1, GBP2, HLA-DMB, CD8A, CCR5, CD40LG, FYB1, RPS3A, CD2, IL2RG, CTSS, IL4R, RPL39, CAPG, CD3D, CCR1, IL2RB, CD4, HLA-DOA, CRTAM, ITGAL, TAP2, ST8SIA4, LCP2, HLA-A, RPL9, ETS1, DARS1, CD3E, ITGB2, LTB, CFP, PTPRC, CCL5, NLRP3, SIT1, PRF1*	0	47	4
30	*RPS24, RPS25, RPS27A, RPL35A, RPS12, RPS15A, RPL30, RPL34, RPS6, RPL23, RPS29, RPL31, RPS7, RPS21, RPS20, RPS23, RPS8, RPS13, RPL21, RPL37, RPL23A, RPL11, RPS27, RPL32, RPL27, RPS3A, RPL39, RPL38, RPL12, RPL4, RPL10A, RPL41, RPL37A, RPL9, RPL5, RPL24, RPL6, RPL19, UBA52, RPS11, RPL7, RPS18, IMP3, RPS14*	0	44	1
31	*PSMB9, CD74, HLA-DRB1, B2M, CD69, TAP1, GZMA, PSME1, HLA-DQA1, CIITA, CASP1, SAMD9L, OAS2, TXNIP, IL4R, MT2A, SRI, VAMP5, PSMB8, HLA-B, XAF1, IL2RB, BTG1, NLRC5, PSME2, ST8SIA4, LCP2, HLA-A, ZBP1, IRF1, IFIT1, TRIM21, IL10RA, TRIM14, PNP, SLAMF7, OAS3, PARP12, CFH, SAMHD1, CCL5, TNFSF10, FGL2, ST3GAL5, PIM1, RTP4, SP110*	0	47	5
32	*HLA-DRA, HLA-DPA1, HLA-DPB1, PSMB9, HLA-E, CD74, HLA-DRB1, B2M, TAP1, PSME1, HLA-DRB5, HLA-DQA1, CIITA, HLA-DMB, CD8A, CTSS, PSMB8, HLA-B, HLA-F, CD4, HLA-DQB1, HLA-DOA, PSME2, TAP2, HLA-A, HLA-C, HSPA5*	0	27	3
33	*RPS24, RPS25, RPS27A, RPL35A, RPS12, RPS15A, RPL30, RPL34, RPS6, RPL23, RPS29, RPL31, RPS7, RPS21, RPS20, RPS23, RPS8, RPS13, RPL21, RPL37, RPL23A, RPL11, RPS27, RPL32, RPL27, RPS3A, RPL39, RPL38, RPL12, RPL4, RPL10A, RPL41, RPL37A, RPL9, RPL5, RPL24, RPL6, RPL19, UBA52, RPS11, RPL7, RPS18, IMP3, RPS14*	0	44	1
34	*HLA-DRA, HLA-DPA1, HLA-DPB1, HLA-E, CD74, HLA-DRB1, B2M, TAP1, PSME1, HLA-DRB5, HLA-DQA1, CIITA, HLA-DMB, CD8A, CTSS, HLA-B, HLA-F, CD4, HLA-DQB1, HLA-DOA, PSME2, TAP2, HLA-A, HLA-C, HSPA5*	0	25	3
35	*RPS24, RPS25, PSMB9, RPS27A, RPL35A, RPS12, RPS15A, PSME1, RPL30, RPL34, RPS6, RPL23, RPS29, RPL31, RPS7, RPS21, RPS20, RPS23, RPS8, RPS13, RPL21, RPL37, RPL23A, RPL11, RPS27, RPL32, RPL27, RPS3A, RPL39, IVD, RPL38, PSMB8, RPL12, AGMAT, RPL4, RPL10A, PSME2, RPL41, RPL37A, RPL9, RPL5, OGDH, RPL24, RPL6, RPL19, UBA52, RPS11, QDPR, CSAD, RPL7, RPS18, RPS14, ALDH9A1*	0	53	1

36	*RPS24, RPS25, PSMB9, RPS27A, B2M, RPL35A, RPS12, RPS15A, PSME1, RPL30, RPL34, RPS6, RPL23, RPS29, RPL31, RPS7, RPS21, RPS20, RPS23, RPS8, RPS13, RPL21, RPL37, CCR5, RPL23A, RPL11, RPS27, RPL32, RPL27, RPS3A, FYN, APOBEC3G, RPL39, RPL38, PSMB8, CALM1, RPL12, CD4, NMT2, RPL4, RPL10A, PSME2, RPL41, ANTXR2, HLA-A, RPL37A, PPIA, RPL9, RPL5, RPL24, RPL6, RPL19, UBA52, RPS11, NUP37, RPL7, SKP1, RPS18, GTF2H5, RPS14*	0	60	1
37	*HLA-DRA, HLA-DPA1, HLA-DPB1, HLA-E, HLA-DRB1, CD226, ITGB1, ITGA4, ITGAM, HLA-DRB5, HLA-DQA1, HLA-DMB, CD8A, CD40LG, CD2, HLA-B, HLA-F, SELL, CD4, HLA-DQB1, HLA-DOA, ITGAL, HLA-A, PECAM1, ITGB2, HLA-C, PTPRC, SPN*	0	28	7
38	*HLA-DRA, HLA-DPA1, HLA-DPB1, HLA-E, HLA-DRB1, CD226, TIGIT, ITGB1, ITGA4, ITGAM, HLA-DRB5, HLA-DQA1, HLA-DMB, CD8A, CD40LG, CD2, HLA-B, HLA-F, SELL, CD4, HLA-DQB1, HLA-DOA, ITGAL, HLA-A, PECAM1, ITGB2, HLA-C, PTPRC, SPN*	0	29	7
39	*HLA-E, B2M, CD226, IFITM1, CD96, ITGB1, ITGA4, LAIR1, NCR3, CD8A, CD40LG, CD3D, HLA-B, HLA-F, SELL, HCST, CRTAM, ITGAL, HLA-A, TYROBP, CD3E, CD300A, ITGB2, HLA-C, SLAMF7, CD200R1*	0	26	8
40	*HLA-E, B2M, CD226, IFITM1, CD96, ITGB1, ITGA4, CD8A, CD40LG, CD3D, HLA-B, HLA-F, SELL, HCST, CRTAM, ITGAL, HLA-A, TYROBP, CD3E, ITGB2, HLA-C, CD200R1*	0	22	8
41	*RPL35A, RPL30, RPL34, RPL23, RPL31, RPL21, RPL37, RPL23A, RPL11, RPL32, RPL27, RPL39, RPL38, RPL12, RPL4, RPL10A, RPL41, RPL37A, RPL9, RPL5, RPL24, RPL6, RPL19, UBA52, RPL7*	0	25	9
42	*VIM, RPS24, PSMB9, LGALS1, GBP1, IFITM1, CCR2, TAP1, IL2RA, PSME1, PTMA, RPL23, RPS29, S100A6, RPS20, CASP1, GADD45B, FCER1G, S100A4, RPL37, MCL1, CCR5, CD40LG, MAL, APOL3, RPS3A, CD2, CYBB, PSMB8, HLA-B, CCR1, TNFRSF1B, TRAF1, HLA-DQB1, RAC2, BTG1, ITGAL, RPL10A, PSME2, RPL41, RCBTB2, HSP90B1, TRAF5, PDE7A, HMGB1, TNFRSF25, CD3E, IRF1, ARHGDIB, CLIC5, ITGB2, BCL2, ATM, CDC42, RPL6, LTB, CCR7, NLRP1, PTPRC, RHOH, PNP, TUBB, HSPA5, GATA3, CCL5, IQGAP2, VIPR1, CXCL8, SPN, S100A10, NLRP3, CNBP, DYNLL1, PIM1, RPS14, SH3BGRL*	0	76	10
43	*HLA-DRA, HLA-DPA1, HLA-DPB1, HLA-E, HLA-DRB1, HLA-DRB5, HLA-DQA1, HLA-DMB, CD40LG, FYN, HLA-B, HLA-F, HLA-DQB1, RAC2, HLA-DOA, ITGAL, HLA-A, ITGB2, HLA-C, PRF1*	0	20	11
44	*HLA-DRA, HLA-DPA1, HLA-DPB1, HLA-E, HLA-DRB1, HLA-DRB5, HLA-DQA1, HLA-DMB, CD40LG, HLA-B, HLA-F, HLA-DQB1, HLA-DOA, HLA-A, HLA-C, PRF1*	0	16	11
45	*HLA-DRA, HLA-DPA1, HLA-DPB1, HLA-E, HLA-DRB1, HLA-DRB5, HLA-DQA1, HLA-DMB, CD40LG, HLA-B, HLA-F, HLA-DQB1, HLA-DOA, HLA-A, HLA-C, PRF1*	0	16	11
46	*HLA-DRA, HLA-DPA1, HLA-DPB1, HLA-E, HLA-DRB1, HLA-DRB5, HLA-DQA1, HLA-DMB, CD40LG, HLA-B, HLA-F, HLA-DQB1, HLA-DOA, HLA-A, HLA-C, PRF1*	0	16	11
47	*HLA-DRA, HLA-DPA1, HLA-DPB1, HLA-E, HLA-DRB1, HLA-DRB5, HLA-DQA1, HLA-DMB, CD40LG, HLA-B, HLA-F, HLA-DQB1, HLA-DOA, HLA-A, HLA-C, PRF1*	0	16	11
48	*HLA-DRA, HLA-DPA1, HLA-DPB1, HLA-E, HLA-DRB1, B2M, GBP1, HLA-DRB5, HLA-DQA1, CIITA, GBP2, OAS2, GBP5, MT2A, HLA-B, HLA-F, HLA-DQB1, HLA-A, IRF1, TRIM21, HLA-C, CAMK2G, TRIM14, OAS3*	0	24	12
49	*HLA-DRA, HLA-DPA1, HLA-DPB1, HLA-E, CISH, HLA-DRB1, B2M, GBP1, HLA-DRB5, HLA-DQA1, CIITA, GBP2, CASP1, OAS2, GBP5, MT2A, HLA-B, HLA-F, HLA-DQB1, HLA-A, IRF1, HLA-C, CAMK2G, CALM3, OAS3*	0	25	12
50	*HLA-DRA, HLA-DPA1, HLA-DPB1, HLA-E, HLA-DRB1, HLA-DRB5, HLA-DQA1, HLA-DMB, CD40LG, FYN, HLA-B, HLA-F, HLA-DQB1, RAC2, HLA-DOA, ITGAL, HLA-A, ITGB2, HLA-C, PRF1*	0	20	11

51	*VIM, HLA-DRA, HLA-DPA1, HLA-DPB1, HLA-E, HLA-DRB1, IL2RA, HLA-DRB5, HLA-DQA1, HLA-DMB, CD40LG, HLA-B, HLA-F, HLA-DQB1, HLA-DOA, HLA-A, GNLY, HLA-C, PECR, CXCL8, PRF1*	0	21	11
52	*HLA-DRA, HLA-DPA1, HLA-DPB1, HLA-E, HLA-DRB1, HLA-DRB5, HLA-DQA1, HLA-DMB, HLA-B, HLA-F, HLA-DQB1, HLA-DOA, HLA-A, HLA-C, PRF1*	0	15	11
53	*HLA-DRA, HLA-DPA1, HLA-DPB1, HLA-E, HLA-DRB1, HLA-DRB5, HLA-DQA1, HLA-DMB, HLA-B, HLA-F, HLA-DQB1, HLA-DOA, HLA-A, HLA-C, PRF1*	0	15	11
54	*HLA-DRA, HLA-DPA1, HLA-DPB1, PSMB9, HLA-E, SOCS2, CD74, IL32, TRIM69, LGALS3, CISH, HLA-DRB1, RPS27A, B2M, GBP1, CD226, IFITM1, CCR2, TAP1, CD96, IL2RA, ITGB1, ITGA4, PSME1, NEFL, ITGAM, HLA-DRB5, HLA-DQA1, CIITA, GBP2, TNFSF12, CASP1, HLA-DMB, LAIR1, TNFSF13B, FCER1G, NCR3, CD8A, OAS2, CD40LG, CTSO, GZMM, FYN, TLR5, IL2RG, TXNIP, CTSS, C5AR2, GBP5, P2RX7, MT2A, PTPRJ, CYBB, PSMB8, CD3D, CALM1, HLA-B, HLA-F, SELL, XAF1, TNFRSF1B, GRAP2, IL2RB, HCST, CD4, HLA-DQB1, CNKSR2, LAT, HLA-DOA, CRTAM, ITGAL, NLRC5, CD27, PSME2, TAP2, LCP2, HLA-A, TYROBP, SYNGAP1, MYLIP, HSP90B1, PEBP1, EDAR, ZBP1, HMGB1, TNFRSF25, CD3E, IRF1, ATP6V0E2, IFIT1, CD300A, ITGB2, BCL2, CDC42, TRIM21, HLA-C, CFL1, LTB, THEM4, CAMK2G, ARRB1, NLRP1, PTPRC, TRIM14, SLAMF7, RPS6KA5, ARPC2, TXK, ARPC3, HSPA5, OAS3, FGF9, UBA52, CFH, SAMHD1, UBE2F, FCN1, NUP37, NLRP3, CD200R1, CTSH, AIM2, SKP1, DYNLL1, JAK3, RPS6KA1, BTLA*	0	127	13
55	*HLA-DRA, HLA-DPA1, HLA-DPB1, HLA-DRB1, HLA-DRB5, HLA-DQA1, PTPRJ, CD3D, CD4, HLA-DQB1, CD3E, PTPRC*	0	12	14
56	*HLA-DRA, HLA-DPA1, HLA-DPB1, HLA-E, HLA-DRB1, HLA-DRB5, HLA-DQA1, HLA-DMB, HLA-B, HLA-F, HLA-DQB1, HLA-DOA, HLA-A, HLA-C, PRF1*	0	15	11
57	*HLA-DRA, HLA-DPA1, HLA-DPB1, HLA-E, HLA-DRB1, HLA-DRB5, HLA-DQA1, HLA-DMB, HLA-B, HLA-F, HLA-DQB1, HLA-DOA, HLA-A, HLA-C, PRF1*	0	15	11
58	*RPS24, RPS25, RPS27A, RPS12, RPS15A, RPS6, RPS29, RPS7, RPS21, RPS20, RPS23, RPS8, RPS13, RPS27, RPS3A, RPS11, RPS18, RPS14*	0	18	15
59	*HLA-DRA, HLA-DPA1, HLA-DPB1, PSMB9, HLA-E, SOCS2, CD74, LGALS3, CISH, HLA-DRB1, RPS27A, B2M, GBP1, CD226, IFITM1, CCR2, TAP1, CD96, IL2RA, ITGB1, ITGA4, PSME1, HLA-DRB5, HLA-DQA1, CIITA, GBP2, CASP1, HLA-DMB, CD8A, OAS2, CD40LG, CTSO, FYN, TLR5, IL2RG, TXNIP, CTSS, GBP5, P2RX7, MT2A, CYBB, PSMB8, CD3D, CALM1, HLA-B, HLA-F, SELL, XAF1, GRAP2, IL2RB, HCST, CD4, HLA-DQB1, LAT, HLA-DOA, CRTAM, ITGAL, NLRC5, PSME2, TAP2, LCP2, HLA-A, TYROBP, HSP90B1, ZBP1, HMGB1, CD3E, IRF1, IFIT1, ITGB2, BCL2, CDC42, TRIM21, HLA-C, THEM4, CAMK2G, NLRP1, PTPRC, RPS6KA5, HSPA5, OAS3, UBA52, CFH, UBE2F, NUP37, NLRP3, CD200R1, CTSH, AIM2, SKP1, DYNLL1, JAK3, RPS6KA1, BTLA*	0	94	13
60	*HLA-DRA, HLA-DPA1, HLA-DPB1, HLA-DRB1, HLA-DRB5, HLA-DQA1, HLA-DMB, FCER1G, CD40LG, HLA-DQB1, HLA-DOA*	0	11	16
61	*HLA-DRA, HLA-DPA1, HLA-DPB1, HLA-DRB1, HLA-DRB5, HLA-DQA1, HLA-DMB, FCER1G, CD40LG, HLA-DQB1, HLA-DOA*	0	11	16
62	*IL2RA, MAF, FCER1G, CD40LG, FYN, IL2RG, IL4R, CD3D, IL2RB, CD4, CD3E, PTPRC, GATA3, JAK3*	0	14	17
63	*CD69, IL2RA, CCR5, CD40LG, IL4R, SELL, CD4, ITGAL, CD27, HLA-A, PECAM1, CCR7, PTPRC, BTLA, PRF1*	0	15	18
64	*HLA-DRA, HLA-DPA1, HLA-DPB1, HLA-DRB1, HLA-DRB5, HLA-DQA1, CD3D, GRAP2, CD4, HLA-DQB1, LAT, LCP2, CD3E*	0	13	14
65	*HLA-DRA, HLA-DPA1, HLA-DPB1, HLA-DRB1, HLA-DRB5, HLA-DQA1, CD3D, GRAP2, CD4, HLA-DQB1, LAT, LCP2, CD3E*	0	13	14
66	*RPS24, RPS25, RPS27A, RPS12, RPS15A, RPS6, RPS29, RPS7, RPS21, RPS20, RPS23, RPS8, RPS13, RPS27, RPS3A, RPS11, RPS18, RPS14*	0	18	15
67	*RPS24, RPS25, RPS27A, RPS12, RPS15A, RPS6, RPS29, RPS7, RPS21, RPS20, RPS23, RPS8, RPS13, RPS27, RPS3A, RPS11, RPS18, RPS14*	0	18	15
68	*RPS24, RPS25, RPS27A, RPS12, RPS15A, RPS6, RPS29, RPS7, RPS21, RPS20, RPS23, RPS8, RPS13, RPS27, RPS3A, RPS11, RPS18, RPS14*	0	18	15
69	*HLA-DRA, HLA-DPA1, HLA-DPB1, HLA-DRB1, HLA-DRB5, HLA-DQA1, CD3D, CD4, HLA-DQB1, CD3E*	0	10	14

70	*RPS24, RPS25, RPS27A, RPS12, RPS15A, RPS6, RPS29, RPS7, RPS21, RPS20, RPS23, RPS8, RPS13, RPS27, RPS3A, RPS11, RPS18, RPS14*	0	18	15
71	*HLA-DRA, HLA-DPA1, HLA-DPB1, HLA-DRB1, IL2RA, ITGA4, ITGAM, HLA-DRB5, HLA-DQA1, HLA-DMB, CD8A, MS4A1, CD2, IL4R, CD3D, CD4, HLA-DQB1, HLA-DOA, CD3E, GP5*	0	20	19
72	*HLA-DRA, HLA-DPA1, HLA-DPB1, HLA-E, HLA-DRB1, RPS27A, B2M, GBP1, IFITM1, HLA-DRB5, HLA-DQA1, CIITA, GBP2, OAS2, GBP5, MT2A, PSMB8, HLA-B, HLA-F, XAF1, HLA-DQB1, HLA-A, IRF1, IFIT1, TRIM21, HLA-C, CAMK2G, TRIM14, OAS3, UBA52, SAMHD1, NUP37*	0	32	12
73	*RPS24, RPS25, PSMB9, RPS27A, B2M, RPL35A, LYZ, RPS12, RPS15A, IGFBP4, PSME1, RPL30, RPL34, RPS6, RPL23, RPS29, RPL31, RPS7, RPS21, RPS20, RPS23, RPS8, RPS13, RPL21, RPL37, CCR5, RPL23A, RPL11, RPS27, RPL32, RPL27, RPS3A, FYN, APOBEC3G, RPL39, CYBB, RPL38, PSMB8, CALM1, RPL12, CD4, NMT2, IGFBP3, RPL4, RPL10A, PSME2, RPL41, HLA-A, RPL37A, HSP90B1, PPIA, RPL9, PDIA6, ATP6V0E2, RPL5, CDC42, RPL24, RPL6, THEM4, RPL19, HSPA5, FGF9, UBA52, RPS11, CXCL8, NUP37, RPL7, SKP1, RPS18, RPS14*	0	70	6
74	*PSMB9, CD74, B2M, IFITM1, TAP1, PSME1, GBP2, CASP1, SAMD9L, LPAR6, TXNIP, IL4R, PSMB8, SELL, PSME2, IRF1, TRIM21, HLA-C, TRIM14, PARP12, LAMP3, RTP4, SP110*	0	23	20
75	*GZMA, IL2RA, CD48, FCER1G, NCR3, CD2, FYN, IL2RG, IL2RB, HCST, LAT, ITGAL, LCP2, IRF1, ITGB2, CDC42, TNFSF10, JAK3, PRF1*	0	19	21
76	*LYZ, CCR2, IL2RA, ITGAM, TNFSF12, FCER1G, OAS2, CTSS, CD3D, SELL, CCR1, CD4, CX3CR1, CD3E, BCL2, LTB, CCR7, CCL5, TNFSF10, EDN1, PRF1*	0	21	22
77	*HLA-DRA, HLA-DPA1, HLA-DPB1, HLA-E, HLA-DRB1, RPS27A, B2M, GBP1, IFITM1, HLA-DRB5, HLA-DQA1, CIITA, GBP2, OAS2, GBP5, MT2A, PSMB8, HLA-B, HLA-F, XAF1, HLA-DQB1, HLA-A, IRF1, IFIT1, HLA-C, CAMK2G, OAS3, UBA52, NUP37*	0	29	12
78	*HLA-DRA, HLA-DRB1, CAPN2, FYN, CD3D, CALM1, CD4, LAT, CAPNS1, CD3E, CALM2, CALM3, PTPRC*	0	13	23
79	*HLA-DRA, HLA-DPA1, HLA-DPB1, HLA-DRB1, IL2RA, MAF, HLA-DRB5, HLA-DQA1, HLA-DMB, IL2RG, IL4R, CD3D, MAML2, IL2RB, CD4, HLA-DQB1, LAT, HLA-DOA, CD3E, GATA3, JAK3*	0	21	24
80	*RPS24, RPS25, RPS27A, RPL35A, RPS12, RPS15A, RPL30, RPL34, TOMM7, RPS6, RPL23, RPS29, RPL31, RPS7, RPS21, RPS20, RPS23, RPS8, RPS13, RPL21, RPL37, RPL23A, RPL11, RPS27, RPL32, RPL27, RPS3A, EEF1B2, RPL39, GAS6, RPL38, SUMF2, RPL12, SLC25A12, RPL4, RPL10A, RPL41, RPL37A, RPL9, TBCD, MAN1C1, TBCA, RPL5, RPL24, RPL6, RPL19, UBA52, ST3GAL1, RPS11, RPL7, RPS18, SSR2, RPS14*	0	53	1
81	*CD69, IL2RA, CD52, CD8A, CCR5, CD2, IL4R, SELL, HLA-DQB1, CD27, PECAM1, CD3E, CCR7, PTPRC, TNFSF10, BTLA*	0	16	25
82	*HLA-DPB1, HLA-DRB1, IL2RA, ITGA4, HLA-DRB5, HLA-DQA1, CD8A, CCR5, CD40LG, FYN, IL4R, CD4, HLA-DQB1, COL18A1, ACE, TNFSF10, PRF1*	0	17	26
83	*LGALS3, CD69, CCR2, IL2RA, ITGB1, ITGA4, ITGAM, FCER1G, CD40LG, IL4R, ITGAL, CD300A, ITGB2, PTPRC, ADGRE1, CD200R1*	0	16	27
84	*HLA-DRA, HLA-DPA1, HLA-DPB1, PSMB9, HLA-E, SOCS2, IL32, CISH, HLA-DRB1, RPS27A, B2M, GBP1, IFITM1, IL2RA, PSME1, NEFL, HLA-DRB5, HLA-DQA1, CIITA, GBP2, TNFSF12, CASP1, TNFSF13B, OAS2, CD40LG, FYN, IL2RG, GBP5, MT2A, PSMB8, CALM1, HLA-B, HLA-F, XAF1, TNFRSF1B, IL2RB, CD4, HLA-DQB1, CNKSR2, LAT, CD27, PSME2, HLA-A, SYNGAP1, PEBP1, EDAR, TNFRSF25, IRF1, IFIT1, TRIM21, HLA-C, LTB, CAMK2G, ARRB1, TRIM14, OAS3, FGF9, UBA52, SAMHD1, NUP37, SKP1, JAK3*	0	62	28
85	*HLA-DRA, HLA-DPA1, HLA-DPB1, HLA-DRB1, ITGAM, HLA-DRB5, HLA-DQA1, HLA-DMB, HLA-DQB1, HLA-DOA, ITGAL, ITGB2, CFH*	0	13	29
86	*IL2RA, MAF, FCER1G, CD40LG, FYN, IL2RG, IL4R, CD3D, GRAP2, IL2RB, CD4, LAT, CD3E, PTPRC, GATA3, JAK3*	0	16	17
87	*IL2RA, MAF, FCER1G, CD40LG, FYN, IL2RG, IL4R, CD3D, GRAP2, IL2RB, CD4, LAT, CD3E, PTPRC, GATA3, JAK3*	0	16	17
88	*IL2RA, MAF, FCER1G, CD40LG, FYN, IL2RG, IL4R, CD3D, GRAP2, IL2RB, CD4, LAT, CD3E, PTPRC, GATA3, JAK3*	0	16	17
89	*HLA-DRA, HLA-DPA1, HLA-DPB1, HLA-DRB1, HLA-DRB5, HLA-DQA1, CD3D, CD4, HLA-DQB1, CD3E, PTPRC*	0	11	14
90	*VIM, IL32, HLA-DRB1, CCR2, HLA-DQA1, TNFSF12, TNFSF13B, CD8A, CCR5, CD40LG, CCR1, TNFRSF1B, CD4, HMGB1, CCL5, CXCL8, TNFSF10*	0	17	30

91	*HLA-DRA, HLA-DPA1, HLA-DPB1, HLA-E, SOCS2, CISH, HLA-DRB1, RPS27A, B2M, GBP1, IFITM1, IL2RA, HLA-DRB5, HLA-DQA1, CIITA, GBP2, CASP1, OAS2, FYN, IL2RG, GBP5, MT2A, PSMB8, HLA-B, HLA-F, XAF1, IL2RB, HLA-DQB1, HLA-A, IRF1, IFIT1, HLA-C, CAMK2G, OAS3, UBA52, NUP37, SKP1, JAK3*	0	38	12
92	*SOCS2, CISH, IL2RA, PTGER2, CD48, GADD45B, IL4R, CAPG, HOPX, SELL, TNFRSF1B, IL2RB, TRAF1, NT5E, DCPS, AHNAK, GLIPR2, TTC39B, BCL2, ADAM19, IL10RA, LTB, RHOH, PRKCH, PNP, CTSZ, SERPINB6, TNFSF10, FGL2, PIM1, EOMES*	0	31	31
93	*CD8A, CD2, CD3D, CD4, ITGAL, CD3E, ITGB2, PTPRC*	0	8	32
94	*IL2RA, MAF, FCER1G, CD40LG, FYN, IL2RG, IL4R, CD3D, IL2RB, CD4, LAT, CD3E, GATA3, JAK3*	0	14	17
95	*HLA-E, B2M, CTSS, HLA-B, HLA-F, HLA-A, HLA-C*	0	7	33
96	*IL2RA, MAF, FCER1G, CD40LG, FYN, IL2RG, IL4R, CD3D, GRAP2, IL2RB, CD4, LAT, CD3E, PTPRC, GATA3, JAK3*	0	16	17
97	*HLA-DRA, HLA-DPA1, HLA-DPB1, HLA-DRB1, HLA-DRB5, HLA-DQA1, CD3D, CD4, HLA-DQB1, CD3E*	0	10	14
98	*HLA-DRA, HLA-DPA1, HLA-DPB1, HLA-DRB1, MAF, HLA-DRB5, HLA-DQA1, HLA-DMB, TLR5, IL2RG, IL4R, HLA-DQB1, HLA-DOA, RORC, GATA3*	0	15	34
99	*LGALS1, SOCS2, B2M, CD69, CCR2, GZMA, RPS20, CIITA, MCL1, CCR5, CD40LG, CD2, CYBB, CCR1, CD4, RORC, PPIA, HMGB1, IRF1, ITGB2, ACE, CCL5, EDN1, NLRP3, PRF1*	0	25	35
100	*HLA-DRA, HLA-DPA1, HLA-DPB1, HLA-E, HLA-DRB1, TAP1, ITGB1, ITGAM, HLA-DRB5, HLA-DQA1, HLA-DMB, CTSS, CYBB, HLA-B, HLA-F, HLA-DQB1, HLA-DOA, TAP2, HLA-A, ATP6V0E2, ITGB2, HLA-C, TUBB*	0	23	36
101	*HLA-DRA, HLA-DPA1, HLA-DPB1, HLA-E, HLA-DRB1, TAP1, ITGB1, ITGAM, HLA-DRB5, HLA-DQA1, HLA-DMB, CTSS, CYBB, HLA-B, HLA-F, HLA-DQB1, HLA-DOA, TAP2, HLA-A, ATP6V0E2, ITGB2, HLA-C, TUBB*	0	23	36
102	*IL2RA, HLA-DQA1, FCER1G, FYN, IL2RG, CD3D, IL2RB, CD4, HLA-DQB1, LAT, CD3E, PTPRC, JAK3*	0	13	17
103	*ITGB1, ITGA4, SELL, ITGAL, PECAM1, ITGB2, CXCL8*	0	7	37
104	*ITGB1, ITGA4, ITGAM, SELL, ITGAL, PECAM1, ITGB2*	0	7	37
105	*HLA-E, B2M, CTSS, HLA-B, HLA-F, HLA-A, HLA-C*	0	7	33
106	*HLA-DRA, HLA-DPA1, HLA-DPB1, HLA-DRB1, HLA-DRB5, HLA-DQA1, HLA-DMB, TNFSF13B, HLA-DQB1, HLA-DOA, ITGAL, ACP5, ATP6V0E2, ITGB2, LTB, CCL5, CXCL8*	0	17	38
107	*IL2RA, MAF, FCER1G, CD40LG, FYN, IL2RG, IL4R, CD3D, IL2RB, CD4, DSC1, CD3E, PTPRC, GATA3, JAK3*	0	15	17
108	*IL2RA, FCER1G, CD8A, MCL1, CD40LG, IL2RG, CD3D, IL2RB, CD4, LAT, TRAF5, CD3E, BCL2, GATA3, CXCL8, JAK3*	0	16	39
109	*PSMB9, HLA-E, B2M, TAP1, CD8A, PSMB8, HLA-B, NLRC5, TAP2, HLA-A*	0	10	40
110	*HLA-DRA, HLA-DRB1, FYN, CD3D, CD4, CD3E, PTPRC*	0	7	41
111	*HLA-DRA, HLA-DRB1, FYN, CD3D, CD4, CD3E, PTPRC*	0	7	41
112	*B2M, TAP1, CIITA, HLA-B, NLRC5, TAP2, HLA-A*	0	7	42
113	*HLA-DRA, HLA-DPA1, HLA-DPB1, HLA-DRB1, ITGA4, HLA-DRB5, HLA-DQA1, HLA-DMB, TNFSF13B, CD40LG, HLA-DQB1, HLA-DOA*	0	12	16
114	*HLA-DRA, HLA-DPA1, HLA-DPB1, HLA-DRB1, ITGA4, HLA-DRB5, HLA-DQA1, HLA-DMB, TNFSF13B, CD40LG, HLA-DQB1, HLA-DOA*	0	12	16
115	*GZMA, IL2RA, CD48, FCER1G, NCR3, FYN, IL2RG, IL2RB, HCST, LAT, ITGAL, LCP2, ITGB2, CDC42, JAK3, PRF1*	0	16	21
116	*HLA-DRA, HLA-DPA1, HLA-DPB1, HLA-DRB1, IL2RA, HLA-DRB5, HLA-DQA1, HLA-DMB, IL2RG, IL4R, CD3D, IL2RB, CD4, HLA-DQB1, LAT, HLA-DOA, RORC, CD3E, GATA3, JAK3*	0	20	24

117	*HLA-DRA, HLA-DPA1, HLA-DPB1, PSMB9, HLA-DRB1, RPS27A, PSME1, HLA-DRB5, HLA-DQA1, PTPRJ, PSMB8, CD3D, GRAP2, CD4, HLA-DQB1, LAT, PSME2, LCP2, CD3E, PTPRC, UBA52, SKP1*	0	22	43
118	*VIM, HLA-DRA, HLA-DPA1, HLA-DPB1, HLA-E, HLA-DRB1, B2M, TAP1, HLA-DRB5, HLA-DQA1, HLA-DMB, GADD45B, OAS2, CD3D, HLA-B, HLA-F, HLA-DQB1, HLA-DOA, ITGAL, TAP2, HLA-A, TRAF5, CD3E, BCL2, SEM1, HLA-C, OAS3, E2F3, JAK3*	0	29	44
119	*CD69, IFITM1, BTG2, PTGER2, CD48, GABBR1, P2RX7, IL4R, CYBB, SRI, SELL, TNFRSF1B, IL2RB, LCP2, IRF1, IL10RA, CCR7, ADGRE1, CCL5, CXCL8, TNFSF10, EDN1, NLRP3, LAMP3, RTP4*	0	25	45
120	*HLA-DRA, HLA-DPA1, HLA-DPB1, PSMB9, HLA-E, CD74, HLA-DRB1, RPS27A, B2M, CD226, IFITM1, TAP1, CD96, ITGB1, ITGA4, PSME1, HLA-DRB5, HLA-DQA1, HLA-DMB, CD8A, CD40LG, CTSO, FYN, CTSS, CYBB, PSMB8, CD3D, CALM1, HLA-B, HLA-F, SELL, GRAP2, HCST, CD4, HLA-DQB1, LAT, HLA-DOA, CRTAM, ITGAL, PSME2, TAP2, LCP2, HLA-A, TYROBP, CD3E, ITGB2, CDC42, TRIM21, HLA-C, THEM4, PTPRC, HSPA5, UBA52, UBE2F, CD200R1, CTSH, SKP1, DYNLL1, BTLA*	0	59	46
121	*HLA-DRA, HLA-DRB1, B2M, GZMA, IL2RA, GADD45B, CD8A, CCR5, IL2RG, CD3D, IL2RB, CD4, HLA-A, CD3E, EOMES*	0	15	47
122	*CD74, IL2RA, MAF, FCER1G, CD40LG, FYN, IL2RG, IL4R, CD3D, GRAP2, IL2RB, CD4, LAT, LCP2, CD3E, PTPRC, GATA3, JAK3*	0	18	17
123	*IL2RA, MAF, FCER1G, CD40LG, FYN, IL2RG, IL4R, CD3D, GRAP2, IL2RB, CD4, LAT, LCP2, CD3E, PTPRC, GATA3, CXCL8, JAK3*	0	18	17
124	*TAP1, CIITA, CD8A, CD40LG, IL2RG, CD3D, CD4, TAP2, CD3E, PTPRC, JAK3*	0	11	48
125	*TNFSF8, IL32, CCR2, IL2RA, TNFSF12, TNFSF13B, CCR5, CD40LG, IL2RG, IL4R, CCR1, TNFRSF1B, IL2RB, CD4, CD27, CX3CR1, EDAR, TNFRSF25, IL10RA, LTB, CCR7, CCL5, CXCL8, TNFSF10, GDF11, ACKR3*	0	26	49
126	*PSMB9, LGALS3, GZMK, DPP4, GZMA, ITGAM, CASP1, FCER1G, CD40LG, CTSO, FYN, CTSS, APOBEC3G, CALM1, GNG2, LCP2, CPQ, IRF1, CALM3, HSPA5, CFH, GATA3, CCL5, FCN1, CTSH, PIM1*	0	26	50
127	*PSMB9, LGALS1, HLA-E, B2M, TAP1, PSMB8, HLA-B, SELL, NT5E, NLRC5, TAP2, HLA-A*	0	12	51
128	*HLA-DRA, HLA-DRB1, CAPN2, FYN, CD3D, CD4, LAT, CAPNS1, CD3E, PTPRC*	0	10	23
129	*TNFSF8, CD69, CD226, CD96, IL2RA, CD48, NCR3, CD40LG, CD2, HLA-B, HLA-A, CD300A, ITGB2, CCR7, PTPRC*	0	15	52
130	*TAP1, CIITA, CD8A, CD40LG, IL2RG, CD3D, CD4, TAP2, CD3E, PTPRC, JAK3*	0	11	48
131	*CCR2, ITGB1, ITGAM, CCR5, ITGAL, CX3CR1, ITGB2, CCL5*	0	8	53
132	*HLA-DRA, HLA-DPA1, HLA-DPB1, PSMB9, HLA-E, CD74, TRIM69, HLA-DRB1, RPS27A, B2M, CD226, IFITM1, TAP1, CD96, ITGB1, ITGA4, PSME1, HLA-DRB5, HLA-DQA1, HLA-DMB, LAIR1, NCR3, CD8A, CD40LG, CTSO, FYN, CTSS, PTPRJ, CYBB, PSMB8, CD3D, CALM1, HLA-B, HLA-F, SELL, GRAP2, HCST, CD4, HLA-DQB1, LAT, HLA-DOA, CRTAM, ITGAL, PSME2, TAP2, LCP2, HLA-A, TYROBP, MYLIP, CD3E, CD300A, ITGB2, CDC42, TRIM21, HLA-C, THEM4, PTPRC, SLAMF7, HSPA5, FGF9, UBA52, UBE2F, CD200R1, CTSH, SKP1, DYNLL1, BTLA*	0	67	46
133	*CD8A, CD2, CD3D, CD4, CD3E, CXCL8*	0	6	54
134	*GZMA, EGLN3, MAF, ITGAM, PTGER2, GBP2, GADD45B, FCER1G, CHI3L1, MCM7, MAL, IL4R, GAS6, CYP1A1, LITAF, LRRN3, HLA-DQB1, CD27, DACT1, NKG7, GCSAM, IL10RA, LTB4R, LTB, GATA3, CXCL8, TNFSF10, FGL2, UBL3*	0	29	55
135	*B2M, CD3D, ITGAL, HLA-A, CD3E, ITGB2, PRF1*	0	7	56
136	*HLA-E, IFITM1, GBP2, OAS2, PSMB8, HLA-B, HLA-F, XAF1, HLA-A, IRF1, IFIT1, HLA-C, OAS3, SAMHD1*	0	14	57
137	*RPS24, RPS25, PSMB9, RPS27A, B2M, RPL35A, RPS12, RPS15A, PSME1, RPL30, RPL34, RPS6, RPL23, RPS29, RPL31, RPS7, RPS21, RPS20, RPS23, RPS8, RPS13, RPL21, RPL37, CCR5, RPL23A, RPL11, RPS27, RPL32, RPL27, RPS3A, FYN, TLR5, APOBEC3G, RPL39, RPL38, PSMB8, CALM1, RPL12, MAML2, CD4, NMT2, RPL4, RPL10A, PSME2, RPL41, ANTXR2, HLA-A, RPL37A, PPIA, RPL9, RPL5, RPL24, RPL6, CFP, RPL19, FGF9, UBA52, RPS11, NUP37, RPL7, SKP1, RPS18, GTF2H5, RPS14*	0	64	6

138	*HLA-DRB1, CCR2, ITGAM, CASP1, CHI3L1, CTSS, P2RX7, HLA-B, SELL, HMOX2, CX3CR1, HLA-C, OGG1, COL18A1, CFH, CXCL8, EDN1, NLRP3, FABP5*	0	19	58
139	*HLA-DRB1, CCR2, IL2RA, ITGA4, CCR5, CD40LG, MS4A1, HLA-DQB1, HLA-A, CCL5, TNFSF10*	0	11	59
140	*HLA-DRA, B2M, GZMA, IL2RA, GADD45B, CD8A, CCR5, IL2RG, CD3D, IL2RB, CD4, HLA-A, CD3E, EOMES*	0	14	47
141	*IL2RA, MAF, FCER1G, FYN, IL2RG, IL4R, CD3D, IL2RB, CD4, LAT, CD3E, GATA3, JAK3*	0	13	17
142	*HLA-DRA, HLA-DPA1, HLA-DPB1, HLA-DRB1, HLA-DRB5, HLA-DQA1, HLA-DMB, CD40LG, HLA-DQB1, HLA-DOA, TRIM21*	0	11	16
143	*CD226, ITGB1, ITGA4, ITGAM, CD2, SELL, ITGAL, PECAM1, ITGB2*	0	9	37
144	*HLA-E, B2M, TAP1, HLA-B, HLA-F, TAP2, HLA-A, HLA-C, HSPA5*	0	9	60
145	*HLA-DRA, HLA-DPA1, HLA-DPB1, HLA-DRB1, HLA-DRB5, HLA-DQA1, FYN, CD3D, GRAP2, CD4, HLA-DQB1, CD3E, CDC42, THEM4, BTLA*	0	15	61
146	*HLA-DRA, HLA-DPA1, HLA-DPB1, HLA-DRB1, HLA-DRB5, HLA-DQA1, HLA-DMB, CD40LG, HLA-DQB1, HLA-DOA, TRIM21*	0	11	16
147	*IL2RA, MAF, FCER1G, CD3D, GRAP2, IL2RB, CD4, LAT, CD3E, IL10RA, GATA3, JAK3*	0	12	62
148	*HLA-DRA, HLA-DPA1, HLA-DPB1, HLA-DRB1, ITGB1, ITGA4, ITGAM, HLA-DRB5, HLA-DQA1, HLA-DMB, CYBB, HLA-DQB1, HLA-DOA, ITGB2*	0	14	29
149	*HLA-DRA, HLA-DPA1, HLA-DPB1, HLA-DRB1, HLA-DRB5, HLA-DQA1, FYN, CD3D, GRAP2, CD4, HLA-DQB1, CD3E, CDC42, THEM4, BTLA*	0	15	61
150	*HLA-E, IFITM1, GBP2, OAS2, PSMB8, HLA-B, HLA-F, XAF1, HLA-A, IRF1, IFIT1, HLA-C, OAS3*	0	13	57
151	*PSMB9, HLA-E, RPS27A, B2M, TAP1, PSME1, CTSS, CYBB, PSMB8, HLA-B, HLA-F, PSME2, TAP2, HLA-A, HLA-C, UBA52*	0	16	63
152	*MAF, FCER1G, FYN, CD3D, CD4, LAT, CD3E, GATA3, JAK3*	0	9	17
153	*PSMB9, HLA-E, RPS27A, B2M, TAP1, PSME1, CTSS, CYBB, PSMB8, HLA-B, HLA-F, PSME2, TAP2, HLA-A, HLA-C, UBA52*	0	16	63
154	*HLA-DRA, HLA-DRB1, IL2RA, ITGA4, ITGAM, HLA-DRB5, CD8A, MS4A1, CD2, IL4R, CD3D, CD4, CD3E, GP5*	0	14	64
155	*MAF, ITGAM, BIN2, GIMAP2, DPYD, CAPG, TNFRSF1B, CD4, TYROBP, ITGB2, IL10RA, GAL3ST4, RPS6KA1*	0	13	65
156	*IL2RA, FCER1G, FYN, IL2RG, CD3D, GRAP2, IL2RB, CD4, LAT, CD3E, PTPRC, JAK3*	0	12	17
157	*CCR2, FCER1G, CCR5, CD40LG, TLR5, P2RX7, GAS6, CCR1, TNFRSF1B, CD4, CX3CR1, CFH, CCL5, CXCL8, EDN1, PRF1*	0	16	66
158	*CASP1, TXNIP, P2RX7, BCL2, NLRP1, NLRP3, AIM2*	0	7	67
159	*CASP1, TXNIP, P2RX7, BCL2, NLRP1, NLRP3, AIM2*	0	7	67
160	*IL2RA, FCER1G, CD40LG, FYN, IL2RG, CD3D, GRAP2, IL2RB, CD4, LAT, CD3E, PTPRC, JAK3*	0	13	17
161	*IL2RA, FCER1G, CD40LG, FYN, IL2RG, CD3D, GRAP2, IL2RB, CD4, LAT, CD3E, PTPRC, JAK3*	0	13	17
162	*TNFSF8, CCR2, IL2RA, TNFSF12, TNFSF13B, CCR5, CD40LG, IL2RG, IL4R, CCR1, TNFRSF1B, IL2RB, CD27, CX3CR1, EDAR, TNFRSF25, IL10RA, LTB, CCR7, CCL5, CXCL8, TNFSF10, ACKR3*	0	23	49
163	*IL2RA, ITGB1, CD2, IL4R, SELL, IL2RB, CD4, CD27, PTPRC, SPN, PIM1*	0	11	68
164	*HLA-DRA, HLA-DPA1, HLA-DPB1, HLA-E, HLA-DRB1, B2M, IL2RA, HLA-DRB5, HLA-DQA1, HLA-DMB, IL2RG, CD3D, HLA-B, HLA-F, IL2RB, CD4, HLA-DQB1, HLA-DOA, ITGAL, HLA-A, ETS1, CD3E, MAD1L1, ITGB2, ATM, HLA-C, E2F3, JAK3*	0	28	69
165	*PSMB9, CISH, CCR2, IL2RA, PSME1, CCR5, IL2RG, PSMB8, CCR1, PSME2, IRF1, CCR7, CXCL8, LAMP3*	0	14	70
166	*PSMB9, HLA-E, RPS27A, B2M, TAP1, PSME1, PSMB8, HLA-B, HLA-F, PSME2, TAP2, HLA-A, HLA-C, UBA52*	0	14	63

167	*PSMB9, HLA-E, LGALS3, RPS27A, B2M, CCR2, IL2RA, PSME1, NEFL, ITGAM, CASP1, FCER1G, GZMM, FYN, TLR5, IL2RG, TXNIP, CTSS, C5AR2, P2RX7, PSMB8, CALM1, HLA-B, GRAP2, IL2RB, CD4, CNKSR2, LAT, NLRC5, PSME2, LCP2, TYROBP, SYNGAP1, HSP90B1, PEBP1, ZBP1, HMGB1, IRF1, ITGB2, BCL2, CDC42, TRIM21, HLA-C, CFL1, THEM4, CAMK2G, ARRB1, NLRP1, RPS6KA5, ARPC2, TXK, ARPC3, FGF9, UBA52, CFH, FCN1, NLRP3, AIM2, SKP1, JAK3, RPS6KA1*	0	61	71
168	*HLA-E, B2M, TAP1, CCR5, APOBEC3G, APOBEC3C, CD3D, CALM1, HLA-B, HLA-F, TNFRSF1B, CD4, RAC2, GNG2, TAP2, HLA-A, TRAF5, CD3E, BCL2, ATM, CALM2, HLA-C, CFL1, CALM3, SAMHD1, SKP1*	0	26	72
169	*FCER1G, FYN, CD3D, GRAP2, CD4, LAT, CD3E, PTPRC*	0	8	73
170	*MAF, FCER1G, CD40LG, FYN, IL4R, CD3D, CD4, LAT, CD3E, PTPRC, GATA3, JAK3*	0	12	17
171	*CD226, ITGB1, ITGA4, CD48, CD2, FYN, LAT, ITGAL, LCP2, ITGB2, PRF1*	0	11	74
172	*HLA-DRB1, LYZ, CCR2, CCR5, CD4, HLA-DQB1, NT5E*	0	7	75
173	*CD74, ITGB1, ITGA4, ITGAM, CD48, FCER1G, CD2, FYN, GAS6, SELL, ITGAL, PPIA, PECAM1, ITGB2, SPN*	0	15	76
174	*CD69, GZMA, IL2RA, FCER1G, FYN, IL2RG, IL2RB, HCST, LAT, LCP2, JAK3, PRF1*	0	12	77
175	*HLA-DRA, HLA-DPA1, HLA-DPB1, HLA-DRB1, ITGB1, ITGA4, ITGAM, HLA-DRB5, HLA-DQA1, HLA-DMB, HLA-DQB1, HLA-DOA, ITGB2*	0	13	29
176	*FCER1G, FYN, CD3D, GRAP2, CD4, LAT, CD3E, PTPRC*	0	8	73
177	*CASP1, TLR5, TXNIP, P2RX7, RAC2, HSP90B1, HMGB1, NLRP1, NLRP3, AIM2*	0	10	78
178	*HLA-DRA, HLA-DPA1, HLA-DPB1, HLA-E, HLA-DRB1, ITGB1, ITGA4, HLA-DRB5, HLA-DQA1, HLA-DMB, GAS6, HLA-B, HLA-F, HLA-DQB1, HLA-DOA, HLA-A, CD300A, CDC42, HLA-C*	0	19	79
179	*HLA-E, CD48, FCER1G, NCR3, FYN, HLA-B, HCST, RAC2, LAT, ITGAL, LCP2, HLA-A, TYROBP, ITGB2, HLA-C, TNFSF10, PRF1*	0	17	80
180	*HLA-DRA, HLA-DPA1, HLA-DPB1, PSMB9, HLA-DRB1, RPS27A, PSME1, HLA-DRB5, HLA-DQA1, PSMB8, CD3D, CD4, HLA-DQB1, PSME2, CD3E, UBA52, SKP1*	0	17	43
181	*ITGAM, SELL, ITGAL, PECAM1, ITGB2, CXCL8*	0	6	81
182	*CCR5, SELL, CX3CR1, LTB4R, HPGD, CCL5*	0	6	82
183	*CASP1, P2RX7, ATP8A2, NLRP3*	0	4	83
184	*ITGB1, ITGA4, ITGAM, CD48, FCER1G, CD2, FYN, GAS6, SELL, ITGAL, PPIA, PECAM1, ITGB2, SPN*	0	14	76
185	*HLA-E, FCER1G, NCR3, FYN, HLA-B, LAT, LCP2, HLA-A, TYROBP, CDC42, HLA-C, PRF1*	0	12	84
186	*HLA-DRA, HLA-DPA1, HLA-DPB1, CD74, HLA-DRB1, ITGAM, HLA-DRB5, HLA-DQA1, CIITA, HLA-DMB, FCER1G, CTSS, CALM1, HLA-DQB1, HLA-DOA, ITGB2, BCL2, CALM2, IL10RA, CAMK2G, CALM3*	0	21	85
187	*HLA-DRA, HLA-DRB1, CCR5, CD3D, CD4, CD3E, PTPRC, EDN1*	0	8	41
188	*CCR2, CCR5, CCR1, CX3CR1, CCL5*	0	5	86
189	*CD74, HLA-DMB, CTSS, CD4, HLA-DOA*	0	5	87
190	*HLA-DRB1, LYZ, IL2RA, ITGA4, TNFSF12, CD40LG, TXNIP, HLA-B, SELL, TNFRSF1B, CX3CR1, HLA-C, TUBB, COL18A1, CXCL8, EDN1*	0	16	88
191	*FCER1G, CD40LG, FYN, IL2RG, CD3D, GRAP2, CD4, LAT, RORC, CD3E, PTPRC, JAK3*	0	12	17
192	*IL2RA, FCER1G, FYN, IL2RG, CD3D, GRAP2, IL2RB, CD4, LAT, CD3E, PTPRC, JAK3*	0	12	17
193	*HLA-DRB1, TNFSF13B, CD8A, CD40LG, TLR5, CD4, HMGB1, BCL2, TRIM21, ACE, TNFSF10*	0	11	89
194	*IL2RA, FCER1G, FYN, IL2RG, CD3D, GRAP2, IL2RB, CD4, LAT, CD3E, PTPRC, JAK3*	0	12	17

195	*CCR2, GPR68, RGL4, PTGER2, LPAR6, CCR5, C5AR2, CCR1, CX3CR1, P2RY11, LTB4R, CCR7, SSTR3, ACKR3*	0	14	90
196	*HLA-E, CD48, FCER1G, NCR3, FYN, HLA-B, HCST, RAC2, LAT, ITGAL, LCP2, HLA-A, TYROBP, ITGB2, HLA-C, TNFSF10, PRF1*	0	17	80
197	*HLA-E, IL2RA, TNFSF12, CHI3L1, CD40LG, CYP1A1, HLA-B, TNFRSF1B, CD4, HLA-C*	0	10	91
198	*FCER1G, CD40LG, FYN, IL2RG, CD3D, GRAP2, CD4, LAT, RORC, CD3E, PTPRC, JAK3*	0	12	17
199	*FCER1G, CD40LG, FYN, IL2RG, CD3D, GRAP2, CD4, LAT, RORC, CD3E, PTPRC, JAK3*	0	12	17
200	*GZMA, IL2RA, FCER1G, CD8A, IL2RG, CD3D, IL2RB, LAT, ITGAL, CD3E, JAK3, PRF1*	0	12	92
201	*IL2RA, FCER1G, CD40LG, FYN, IL2RG, CD3D, GRAP2, IL2RB, CD4, LAT, CD3E, PTPRC, JAK3*	0	13	17
202	*CD69, IL2RA, FCER1G, CD40LG, IL2RG, CD3D, IL2RB, CD4, LAT, CD3E, PTPRC, JAK3*	0	12	17
203	*IL2RA, FCER1G, CD8A, FYN, IL2RG, CD3D, IL2RB, LAT, CD3E, JAK3, PRF1*	0	11	92
204	*CCR2, CCR5, CCR1, CX3CR1, CCR7, CCL5, CXCL8, ACKR3*	0	8	93
205	*FCER1G, CD3D, LAT, LCP2, RORC, CD3E, BCL2, BCL11B*	0	8	94
206	*S100A6, PTGER2, PTGDS, HPGD, EDN1, S100A10*	0	6	95
207	*CCR2, CCR5, CCR1, CX3CR1, CCR7, CCL5, CXCL8, ACKR3*	0	8	93
208	*B2M, CD8A, FYN, CD3D, GRAP2, LAT, LCP2, HLA-A, CD3E, PTPRC, PRF1*	0	11	96
209	*B2M, CD8A, FYN, CD3D, GRAP2, LAT, LCP2, HLA-A, CD3E, PTPRC, PRF1*	0	11	96
210	*ITGB1, ITGAM, CD52, TNFSF13B, MCL1, VNN2, IL4R, HLA-A, CCR7, TNFSF10*	0	10	97
211	*HLA-DRA, HLA-DRB1, ITGAL, ITGB2, PTPRC*	0	5	98
212	*ITGB1, ITGA4, ITGAL, ITGB2, CXCL8*	0	5	37
213	*ITGB1, ITGA4, ITGAL, ITGB2, CXCL8*	0	5	37
214	*RPS24, RPS25, EID3, RPS27A, B2M, RPL35A, LYZ, RPS12, DPP4, RPS15A, IGFBP4, RPL30, RPL34, TOMM7, RPS6, RPL23, RPS29, RPL31, RPS7, RPS21, RPS20, RPS23, RPS8, RPS13, RPL21, RPL37, RPL23A, RPL11, RPS27, RPL32, RPL27, RPS3A, EEF1B2, RPL39, GAS6, RPL38, SUMF2, SUMO3, GZMH, RPL12, SLC25A12, IGFBP3, GNG2, RPL4, RPL10A, RPL41, ST8SIA4, NSMCE4A, RPL37A, HSP90B1, RPL9, PDIA6, TBCD, MAN1C1, TBCA, RPL5, RPL24, RPL6, CFP, CTSZ, RPL19, HSPA5, UBA52, ACE, ST3GAL1, RPS11, CXCL8, NUP37, RPL7, CTSH, TRAPPC6A, DYNLL1, RPS18, ST3GAL5, SSR2, RPS14*	0	76	99
215	*GPR68, RGL4, PTGER2, LPAR6, CCR5, C5AR2, CCR1, CX3CR1, P2RY11, LTB4R, CCR7, SSTR3, ACKR3*	0	13	90
216	*B2M, IL2RA, CD8A, IL2RG, CD3D, IL2RB, HLA-A, CD3E, CALM3, PRF1, EOMES*	0	11	100
217	*B2M, IL2RA, CD8A, IL2RG, CD3D, CALM1, IL2RB, HLA-A, CD3E, PRF1, EOMES*	0	11	100
218	*ITGB1, ITGA4, ITGAM, IL4R, ITGAL, ITGB2, CXCL8*	0	7	37
219	*HLA-E, TIGIT, LAIR1, FYN, HLA-B, LAT, LCP2, HLA-A, CD300A, HLA-C*	0	10	101
220	*HLA-DRA, HLA-DRB1, CD3D, CD4, CD3E, PTPRC*	0	6	41
221	*IL32, CCR2, ITGA4, ITGAM, CCR5, PTGDS, TNFRSF1B, TRAF1, CD4, CX3CR1, PECAM1, ITGB2, LTB4R, CCL5, CXCL8, TNFSF10, EDN1*	0	17	102
222	*PSMB9, RPS27A, PSME1, TNFSF12, TNFSF13B, CD40LG, PSMB8, TNFRSF1B, CD27, PSME2, EDAR, TNFRSF25, LTB, UBA52, SKP1*	0	15	103
223	*CCR2, RPS20, TXNIP, MYL6, CALM1, ITGAL, ACP5, BCL2, CDC42, HLA-C, MKI67, CCL5*	0	12	104
224	*HLA-DRA, HLA-DRB1, FYN, CD3D, GRAP2, CD4, LAT, LCP2, CD3E, CDC42, PTPRC, SLA2*	0	12	105
225	*FCER1G, CD40LG, FYN, IL2RG, CD3D, GRAP2, CD4, LAT, RORC, CD3E, PTPRC, JAK3*	0	12	17

226	*FCER1G, FYN, CD3D, GRAP2, CD4, LAT, CD3E, PTPRC*	0	8	73
227	*IL2RA, FCER1G, CCR5, CD3D, CD4, LAT, CD3E, JAK3*	0	8	106
228	*IL2RA, FCER1G, CD3D, CD4, LAT, RORC, CD3E, JAK3*	0	8	106
229	*CCR2, CCR5, CD40LG, IL4R, CCR1, CD4, CCR7*	0	7	107
230	*IL2RA, FCER1G, FYN, IL2RG, IL2RB, RAC2, LAT, LCP2, CDC42, JAK3*	0	10	108
231	*HLA-DRA, HLA-DRB1, IL2RA, CD40LG, IL4R, GATA3*	0	6	109
232	*ITGB1, ITGAM, CASP1, CALM1, IRF1, ITGB2, CALM2, CFL1, CALM3, CXCL8, NLRP3*	0	11	110
233	*S100A6, PTGER2, PTGDS, HPGDS, HPGD, EDN1, S100A10*	0	7	95
234	*HLA-DRA, HLA-DRB1, IL2RA, CCR5, CD3D, CD4, CD3E, IRF1, PRF1*	0	9	111
235	*VIM, SKAP1, CD8A, CCR5, FYB1, FYN, CD3D, GRAP2, CD4, LAT, LCP2, CD3E, CDC42, GATA3*	0	14	112
236	*IL2RA, MAF, IL2RG, IL4R, IL2RB, RAC2, BCL2, CDC42, GATA3, CXCL8, JAK3, PRF1*	0	12	113
237	*LGALS3, CCR2, DPP4, AIF1, PTGER2, CYP1A1, SELL, CCR1, IGFBP3, RRM2, HMGB1, FHIT, BCL2, HPGD, OGG1, COL18A1, FGF9, UBA52, GATA3, CXCL8, EDN1*	0	21	114
238	*FCER1G, FYN, CD3D, CD4, CD3E, JAK3*	0	6	115
239	*HLA-DRA, HLA-DRB1, IL2RG, IL4R, JAK3*	0	5	116
240	*CD8A, CD40LG, FYN, CD3D, CALM1, GRAP2, CD4, LAT, LCP2, CALM2, PTPRC*	0	11	117
241	*TMSB4X, CD74, PDE9A, IL2RA, ITGB1, ITGA4, ITGAM, CD48, FCER1G, CD2, FYN, IL2RG, P2RX7, GAS6, SRI, CALM1, SELL, IL2RB, CAPZB, RAC2, GNG2, LAT, ITGAL, LCP2, PPIA, PECAM1, IRF1, ITGB2, CDC42, GP5, CFL1, ARRB1, PRKCH, TTN, HSPA5, GATA3, SERPINB6, SPN, S100A10, JAK3*	0	40	118
242	*HLA-DRA, HLA-DRB1, CD40LG, CD4*	0	4	119
243	*CALM1, CALM2, CAMK1, CALM3*	0	4	120
244	*FCER1G, CD40LG, FYN, CD3D, GRAP2, CD4, LAT, LCP2, CD3E, PTPRC*	0	10	73
245	*FCER1G, CD40LG, FYN, CD3D, GRAP2, CD4, LAT, LCP2, CD3E, PTPRC*	0	10	73
246	*HLA-DRA, HLA-DRB1, CCL5*	0	3	121
247	*IGFBP4, GZMH, IGFBP3*	0	3	122
248	*FCER1G, FYN, IL2RG, CD3D, CD4, LAT, RORC, CD3E, JAK3*	0	9	17
249	*CCR2, GPR68, PTGER2, LPAR6, CCR5, C5AR2, CCR1, CX3CR1, P2RY11, LTB4R, CCR7, SSTR3, CCL5, CXCL8, EDN1, ACKR3*	0	16	90
250	*CCR2, GPR68, PTGER2, GABBR1, LPAR6, CCR5, C5AR2, CCR1, GNG2, CX3CR1, P2RY11, LTB4R, CCR7, SSTR3, CCL5, VIPR1, CXCL8, EDN1, ACKR3*	0	19	90
251	*GZMA, FCER1G, CD8A, CD3D, LAT, CD3E, PRF1*	0	7	123
252	*ITGAM, CASP1, CYBB, LAT, LCP2, ITGB2, NLRP3*	0	7	124
253	*LGALS1, IL2RA, IL2RG, IL2RB, NT5E, LAT, TNFSF10, JAK3, PRF1*	0	9	125
254	*CALM1, CALM2, ARRB1, CALM3, PDE6B*	0	5	126
255	*HLA-DRA, HLA-DPA1, HLA-DPB1, HLA-DRB1, ITGB1, HLA-DRB5, HLA-DQA1, CIITA, HLA-DMB, CCR5, CD40LG, HLA-DQB1, HLA-DOA, BCL2, IL10RA*	0	15	127

256	*VIM, LGALS3, CCR2, AIF1, BTG2, TNFSF13B, XYLT1, CDC42, LTB4R, LTB, APEX1, CXCL8*	0	12	128
257	*CCR2, CD8A, CCR5, CD40LG, APOBEC3G, CD4, HLA-A, PPIA, CX3CR1, CCL5, TNFSF10*	0	11	129
258	*CD226, ITGB1, LAIR1, CD4, LAT, VIPR1, TNFSF10*	0	7	130
259	*CCR2, ITGA4, CCR5, CYBB, RAC2, CDC42, CFL1, LTB4R, CXCL8*	0	9	131
260	*GBP1, GBP2, CASP1, OAS2, CARD16, TXNIP, GBP5, P2RX7, CYBB, ANTXR2, TRAF5, BCL2, NLRP1, OAS3, CCL5, CXCL8, NLRP3, AIM2*	0	18	132
261	*CCR2, CCR5, C5AR2, CCR1, CX3CR1, CCR7, SSTR3, CCL5, CXCL8, EDN1, ACKR3*	0	11	93
262	*HLA-DRA, FYN, CD3D, GRAP2, CD4, LAT, LCP2, CD3E, CDC42, PTPRC, SLA2*	0	11	105
263	*CCR5, CD3D, CD4, CD3E*	0	4	133
264	*ITGB1, FCER1G, FYN, GP5*	0	4	134
265	*TNFSF8, CD69, IL2RA, CD40LG, CCR1, TRAF1, LCP2, ETS1, IRF1, LTB, CCL5, CXCL8, TNFSF10, ACKR3*	0	14	135
266	*PSMB9, RPS27A, CCR2, IL2RA, GPR68, PSME1, NEFL, PTGER2, GABBR1, LPAR6, CCR5, FYN, IL2RG, C5AR2, ARHGEF6, PSMB8, CALM1, RGS10, CCR1, IL2RB, RAC2, CNKSR2, GNG2, LAT, PSME2, SYNGAP1, PEBP1, CX3CR1, PDE7A, CDC42, P2RY11, LTB4R, CAMK2G, ARRB1, CCR7, PRKCH, SSTR3, FGF9, UBA52, CCL5, VIPR1, CXCL8, EDN1, ACKR3, JAK3, RPS6KA1, PLEKHG2*	0	47	136
267	*CD74, CD52, LAIR1, TNFSF13B, MCL1, CD40LG, BCL2, ATM, CCR7*	0	9	137
268	*CCR2, CCR5, C5AR2, CCR1, CX3CR1, CCR7, SSTR3, CCL5, CXCL8, EDN1, ACKR3*	0	11	93
269	*HLA-DRA, HLA-DPA1, HLA-DPB1, CD74, HLA-DRB1, HLA-DRB5, HLA-DQA1, HLA-DMB, CTSO, CTSS, HLA-DQB1, HLA-DOA, CTSH, DYNLL1*	0	14	138
270	*HLA-DRA, HLA-DPA1, HLA-DPB1, CD74, HLA-DRB1, HLA-DRB5, HLA-DQA1, HLA-DMB, CTSO, CTSS, HLA-DQB1, HLA-DOA, CTSH, DYNLL1*	0	14	138
271	*TMSB4X, IL2RA, FCER1G, FYN, IL2RG, GAS6, CALM1, IL2RB, RAC2, GNG2, LAT, LCP2, PPIA, PECAM1, CDC42, GP5, CFL1, ARRB1, PRKCH, TTN, HSPA5, JAK3*	0	22	139
272	*HLA-DRB1, HLA-DQA1, CASP1, MS4A1, CD4, HLA-DQB1, OGG1, CXCL8*	0	8	140
273	*AIF1, CASP1, CCR5, CD40LG, FYN, TLR5, CD4, RAC2, ITGAL, ITGB2, BCL2, EDN1*	0	12	141
274	*CCR5, CD40LG, IL4R, CCR1, CD4, CCR7*	0	6	107
275	*PSMB9, GBP1, TAP1, CIITA, CYBB, HLA-B, IRF1*	0	7	142
276	*CD226, CCR2, AIF1, TNFSF13B, CD40LG, CXCL8, EDN1*	0	7	143
277	*HLA-DRA, IL2RA, CD3D, CD4, CD3E, IRF1, PRF1*	0	7	111
278	*PSMB9, GBP1, TAP1, CIITA, CYBB, HLA-B, IRF1*	0	7	142
279	*TAP1, TAP2, EDN1*	0	3	144
280	*GZMA, CASP1, CD8A, CD2, ITGAL, IRF1, APEX1, TNFSF10, PRF1*	0	9	145
281	*CCR5, CD2, CD3D, CD4, CD3E*	0	5	133
282	*NEFL, CALM1, CAMK2G, CALM3*	0	4	146
283	*IL2RA, IL2RG, IL2RB, JAK3*	0	4	147
284	*B2M, TAP1, CD8A, TAP2*	0	4	148
285	*ITGB1, FCER1G, FYN, GP5*	0	4	134
286	*FCER1G, CD8A, CD3D, LAT, CD3E, PRF1*	0	6	123
287	*FCER1G, FYN, CD3D, GRAP2, CD4, LAT, LCP2, CD3E*	0	8	73

288	*PSMB9, RPS27A, IL2RA, PSME1, NEFL, FYN, IL2RG, PSMB8, CALM1, IL2RB, CNKSR2, LAT, PSME2, ST8SIA4, SYNGAP1, PEBP1, CAMK2G, ARRB1, RPS6KA5, FGF9, UBA52, COL6A2, JAK3*	0	23	149
289	*VIM, LGALS1, IL32, ITGB1, IGFBP4, GADD45B, TPM2, MCM7, CAPG, IGFBP3, NT5E, GPX7, COL5A3, COL6A2, CXCL8*	0	15	150
290	*ITGB1, FCER1G, CD40LG, FYN, PTPRJ, LAT, LCP2, CDC42, GP5, CXCL8*	0	10	151
291	*HLA-DPB1, CD69, CCR5, GNLY, HLA-C, PTPRC, ACE, GATA3, CD200R1, FGL2*	0	10	152
292	*IL2RA, CD40LG, IL2RG, IL4R, TNFRSF1B, IL2RB*	0	6	153
293	*IL2RA, CD40LG, IL2RG, IL4R, TNFRSF1B, IL2RB*	0	6	153
294	*CCR2, CCR5, CCR1, CX3CR1, CCR7, SSTR3*	0	6	154
295	*IL32, LGALS3, CCR2, GZMA, TNFSF13B, CHI3L1, CCR5, PDE7A, HMGB1, ADAM19, OGG1, CXCL8, EDN1, FGL2*	0	14	155
296	HLA-E, B2M, TAP1, PTGER2, CCR5, CALM1, HLA-B, HLA-F, CCR1, RAC2, GNG2, TAP2, HLA-A, TRAF5, CALM2, HLA-C, IL10RA, CALM3, CCL5, CXCL8, E2F3	0	21	156
297	CD8A, FYN, CD4, LCP2, CDC42, PTPRC, RPS6KA5	0	7	157
298	TRIB2, FCER1G, IL2RG, CTSS, PSMB8, TNFRSF1B, TRAF1, GPNMB, IGFBP3, KCNN4, ETS1, PECAM1, ITGB2, IL10RA, FGF9, CFH, ACE	0	17	158
299	B2M, S100A11, PARK7, GPNMB, MGMT, IRF1, ATM, BCCIP, HSPA5, TNFSF10	0	10	159
300	ITGB1, CASP1, GADD45B, PTGDS, IGFBP3, CCL5, CXCL8, EDN1	0	8	160
301	CD2, FYN, LAT, LCP2	0	4	161

i

Order	Term	Database	Overlap	p value	Adjusted p value
1	NRF2 pathway WP2884	WikiPathways_2019_Human	20/86	6.08E-11	1.90E-07
2	Amino acid transport across the plasma membrane	BioPlanet_2019	9/18	6.40E-09	6.65E-06
3	Amino acid transport across the plasma membrane Homo sapiens R-HSA-352230	Reactome_2016	9/18	6.40E-09	6.65E-06
4	Nuclear Receptors Meta-Pathway WP2882	WikiPathways_2019_Human	26/200	5.13E-08	4.00E-05
5	Glutamine in Cancer Metabolism	Elsevier_Pathway_Collection	9/24	1.40E-07	8.73E-05
6	Amino acid and oligopeptide SLC transporters	BioPlanet_2019	9/27	4.52E-07	2.02E-04
7	Amino acid and oligopeptide SLC transporters Homo sapiens R-HSA-425374	Reactome_2016	9/27	4.52E-07	2.02E-04
8	Xenobiotic Metabolism	MSigDB_Hallmark_2020	19/136	1.12E-06	4.37E-04
9	mTORC1 Signaling	MSigDB_Hallmark_2020	23/195	1.77E-06	6.12E-04
10	Metabolic reprogramming in colon cancer WP4290	WikiPathways_2019_Human	10/40	2.03E-06	6.33E-04
11	Oxidative Stress in Amyotrophic Lateral Sclerosis	Elsevier_Pathway_Collection	6/12	2.47E-06	7.01E-04
12	Phytochemical activity on NRF2 transcriptional activation WP3	WikiPathways_2019_Human	6/13	4.44E-06	1.12E-03
13	Unfolded Protein Response	MSigDB_Hallmark_2020	16/110	4.67E-06	1.12E-03
14	Trans-sulfuration and one carbon metabolism WP2525	WikiPathways_2019_Human	8/28	7.44E-06	1.66E-03
15	Photodynamic therapy-induced NFE2L2 (NRF2) survival signaling WP3612	WikiPathways_2019_Human	7/21	9.03E-06	1.88E-03
16	Serine glycine biosynthesis Homo sapiens P02776	Panther_2016	4/5	1.08E-05	2.07E-03
17	Transport of inorganic cations/anions and amino acids/oligopeptides Homo sapiens R-HSA-425393	Reactome_2016	11/58	1.13E-05	2.07E-03
18	Oxidative Stress, All-Trans-Retinal and Lipofuscin Toxicity in AMD	Elsevier_Pathway_Collection	6/15	1.21E-05	2.10E-03
19	SLC-mediated transmembrane transport Homo sapiens R-HSA-425407	Reactome_2016	19/164	1.84E-05	3.02E-03
20	SLC-mediated transmembrane transport	BioPlanet_2019	18/152	2.26E-05	3.53E-03
21	Transport of inorganic cations/anions and amino acids/oligopeptides	BioPlanet_2019	10/54	3.53E-05	5.24E-03
22	Keap1-Nrf2 pathway	BioPlanet_2019	5/12	5.39E-05	7.65E-03
23	Transmembrane transport of small molecules	BioPlanet_2019	24/260	6.91E-05	9.22E-03
24	serine and glycine biosynthesis Homo sapiens SER-GLYSYN-PWY	HumanCyc_2016	4/7	7.10E-05	9.22E-03
25	Glutathione metabolism	KEGG_2019_Human	8/38	8.33E-05	1.04E-02
26	NRF2-ARE regulation WP4357	WikiPathways_2019_Human	6/21	1.08E-04	1.29E-02
27	Transmembrane transport of small molecules Homo sapiens R-HSA-382551	Reactome_2016	29/357	1.26E-04	1.42E-02
28	Amino acid biosynthesis and interconversion (transamination)	BioPlanet_2019	5/14	1.28E-04	1.42E-02
29	mTOR Signaling Activation by Amino Acids	Elsevier_Pathway_Collection	8/41	1.47E-04	1.58E-02
30	Amino acid synthesis and interconversion (transamination) Homo sapiens R-HSA-70614	Reactome_2016	5/16	2.62E-04	2.72E-02
31	Reactive Oxygen Species Pathway	MSigDB_Hallmark_2020	8/45	2.90E-04	2.91E-02

32	Ferroptosis	KEGG_2019_Human	7/35	3.27E-04	3.09E-02
33	Ferroptosis WP4313	WikiPathways_2019_Human	7/35	3.27E-04	3.09E-02
34	Protein Nuclear Import and Export	Elsevier_Pathway_Collection	5/17	3.59E-04	3.29E-02
35	Glutathione metabolism	BioPlanet_2019	7/36	3.92E-04	3.50E-02
36	Metabolic Reprogramming in Cancer: Overview	Elsevier_Pathway_Collection	9/59	4.05E-04	3.51E-02
37	Glycolysis Activation in Cancer (Warburg Effect)	Elsevier_Pathway_Collection	7/38	5.55E-04	4.68E-02

Table 1i continued

Order	Genes	Upregulated	Downregulated	Gro
1	*NQO1, GCLC, MAFG, HMOX1, FTL, SLC5A3, SLC6A9, TXNRD1, SQSTM1, GSR, PRDX1, GCLM, G6PD, PGD, KEAP1, GSTP1, SLC39A7, SLC7A11, SLC6A6, SLC39A6*	20	0	1
2	*SLC7A5, SLC43A2, SLC7A1, SLC3A2, SLC38A2, SLC1A5, SLC1A4, SLC7A11, SLC6A6*	9	0	2
3	*SLC7A5, SLC43A2, SLC7A1, SLC3A2, SLC38A2, SLC1A5, SLC1A4, SLC7A11, SLC6A6*	9	0	2
4	*NQO1, CPT1A, SLC7A5, GCLC, MAFG, HMOX1, FTL, SLC5A3, SLC6A9, TXNRD1, SQSTM1, GSR, PRDX1, GCLM, G6PD, PGD, KEAP1, GSTP1, BAX, SLC39A7, SRGN, SLC7A11, SLC6A6, CPEB4, PPARA, SLC39A6*	26	0	1
5	*SLC7A5, PSAT1, GCLC, SLC3A2, SLC1A5, PYCR1, GCLM, SLC7A11, PSPH*	9	0	3
6	*SLC7A5, SLC43A2, SLC7A1, SLC3A2, SLC38A2, SLC1A5, SLC1A4, SLC7A11, SLC6A6*	9	0	2
7	*SLC7A5, SLC43A2, SLC7A1, SLC3A2, SLC38A2, SLC1A5, SLC1A4, SLC7A11, SLC6A6*	9	0	2
8	*NQO1, GCLC, HMOX1, GABARAPL1, SLC1A5, ETFDH, AKR1C3, ARG2, PYCR1, GSR, PGD, EPHX1, UGDH, NPC1, SHMT2, SLC6A6, HES6, LONP1, PINK1*	19	0	4
9	*SLC7A5, PSAT1, GCLC, EDEM1, PHGDH, SLC1A5, TXNRD1, SQSTM1, MTHFD2, GSR, SLC1A4, PRDX1, DDIT3, G6PD, PPP1R15A, SLC7A11, SHMT2, SLC6A6, GLA, GAPDH, NAMPT, DDX39A, PSPH*	23	0	5
10	*PSAT1, SLC1A5, TALDO1, PYCR1, G6PD, PGD, SHMT2, PKM, GAPDH, PSPH*	10	0	6
11	*NQO1, GCLC, HMOX1, GSR, GCLM, KEAP1*	6	0	7
12	*NQO1, GCLC, HMOX1, GCLM, KEAP1, SLC7A11*	6	0	7
13	*SLC7A5, PSAT1, HERPUD1, TARS1, IARS1, CHAC1, VEGFA, EDEM1, CEBPG, MTHFD2, SLC1A4, ATF4, CXXC1, SLC30A5, XPOT, EDC4*	16	0	8
14	*PSAT1, GCLC, PHGDH, MTHFD2, MTHFD1L, GCLM, SHMT2, PSPH*	8	0	9
15	*NQO1, GCLC, HMOX1, GCLM, EPHX1, KEAP1, GSTP1*	7	0	7
16	*PSAT1, PHGDH, SHMT2, PSPH*	4	0	10
17	*SLC7A5, SLC43A2, SLC7A1, SLC3A2, SLC38A2, SLC1A5, CTNS, SLC12A7, SLC1A4, SLC7A11, SLC6A6*	11	0	2
18	*NQO1, HMOX1, VEGFA, GCLM, KEAP1, HIF1A*	6	0	11

19	*SLC7A5, SLC43A2, SLC7A1, SLC3A2, SLC38A2, SLC5A3, SLC1A5, SLC6A9, CTNS, SLC12A7, SLCO3A1, SLC1A4, SLC35A2, NUP153, SLC39A7, SLC7A11, SLC6A6, SLC30A5, SLC39A6*	19	0	12
20	*SLC7A5, SLC43A2, SLC7A1, SLC3A2, SLC38A2, SLC5A3, SLC1A5, SLC6A9, SLC12A7, SLCO3A1, SLC1A4, SLC35A2, NUP153, SLC39A7, SLC7A11, SLC6A6, SLC30A5, SLC39A6*	18	0	12
21	*SLC7A5, SLC43A2, SLC7A1, SLC3A2, SLC38A2, SLC1A5, SLC12A7, SLC1A4, SLC7A11, SLC6A6*	10	0	2
22	*NQO1, GCLC, HMOX1, GCLM, KEAP1*	5	0	7
23	*SLC7A5, SLC43A2, SLC7A1, HMOX1, SLC3A2, FTL, SLC38A2, SLC5A3, SLC1A5, SLC6A9, SLC12A7, SLCO3A1, ABCB6, SLC1A4, ATP6V1A, SLC35A2, NUP153, SLC39A7, SLC7A11, SLC6A6, ATP1B3, SLC30A5, MCOLN1, SLC39A6*	24	0	12
24	*PSAT1, PHGDH, SHMT2, PSPH*	4	0	10
25	*ODC1, GCLC, CHAC1, GSR, GCLM, G6PD, PGD, GSTP1*	8	0	13
26	*NQO1, GCLC, HMOX1, GCLM, KEAP1, SLC7A11*	6	0	7
27	*SLC7A5, SLC43A2, SLC7A1, HMOX1, SLC3A2, FTL, SLC38A2, SLC5A3, CLCN7, SLC1A5, SLC6A9, CTNS, SLC12A7, SLCO3A1, ABCB6, SLC1A4, ATP6V1A, CLCN6, SLC35A2, NUP153, SLC39A7, SLC7A11, SLC6A6, ATP1B3, NIPAL1, SLC30A5, MCOLN1, ATP13A1, SLC39A6*	29	0	12
28	*PSAT1, PHGDH, PYCR1, GPT2, PSPH*	5	0	14
29	*SLC7A5, SLC3A2, SLC1A5, FNIP1, SESN2, FLCN, ATF4, PPP1R15A*	8	0	15
30	*PSAT1, PHGDH, PYCR1, GPT2, PSPH*	5	0	14
31	*NQO1, GCLC, FTL, TXNRD1, GSR, PRDX1, GCLM, G6PD*	8	0	16
32	*GCLC, HMOX1, SLC3A2, FTL, SAT2, GCLM, SLC7A11*	7	0	17
33	*GCLC, HMOX1, SLC3A2, FTL, SAT2, GCLM, SLC7A11*	7	0	17
34	*XPO1, NXF1, RCC1, NUP153, XPOT*	5	0	18
35	*ODC1, GCLC, GSR, GCLM, G6PD, PGD, GSTP1*	7	0	13
36	*PSAT1, PHGDH, SLC1A5, PYCR1, SHMT2, PKM, GAPDH, HIF1A, PSPH*	9	0	19
37	*PSAT1, PHGDH, SHMT2, PKM, GAPDH, HIF1A, PSPH*	7	0	19

j

Rank	Motif	Name	P-value	log P-pvalue
1	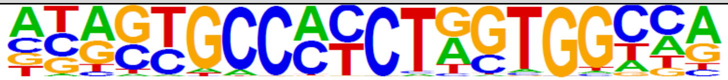	CTCF(Zf)/CD4+-CTCF-ChIP-Seq(Barski_et_al.)	1.00E-40	-9.25E+01
2	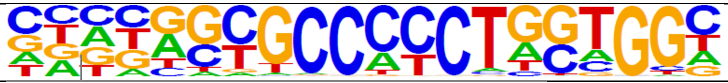	BORIS(Zf)/K562-CTCFL-ChIP-Seq(GSE32465)	1.00E-32	-7.39E+01
3	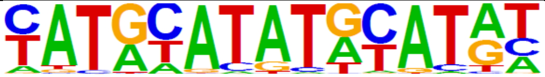	OCT:OCT(POU,Homeobox)/NPC-OCT6-ChIP-Seq(GSE43916)	1.00E-22	-5.25E+01
4	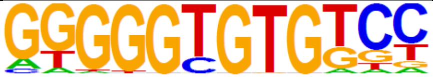	KLF10(Zf)/HEK293-KLF10.GFP-ChIP-Seq(GSE58341)	1.00E-15	-3.55E+01
5	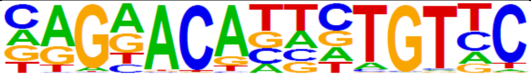	GRE(NR),IR3/RAW264.7-GRE-ChIP-Seq(Unpublished)	1.00E-13	-3.05E+01
6	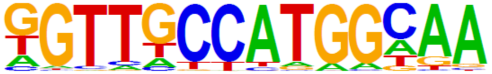	X-box(HTH)/NPC-H3K4me1-ChIP-Seq(GSE16256)	1.00E-13	-3.01E+01
7	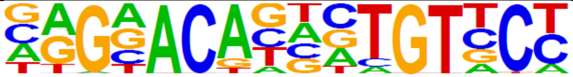	GRE(NR),IR3/A549-GR-ChIP-Seq(GSE32465)	1.00E-09	-2.21E+01
8	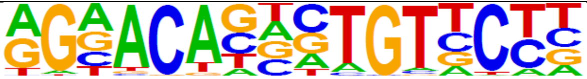	ARE(NR)/LNCAP-AR-ChIP-Seq(GSE27824)	1.00E-09	-2.12E+01
9	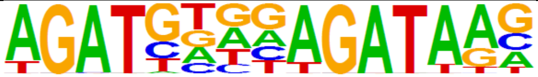	GATA3(Zf),DR4/iTreg-Gata3-ChIP-Seq(GSE20898)	1.00E-09	-2.09E+01
10	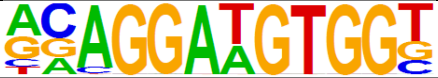	ETS:RUNX(ETS,Runt)/Jurkat-RUNX1-ChIP-Seq(GSE17954)	1.00E-08	-1.87E+01
11	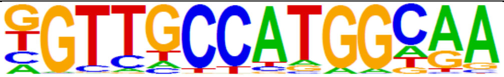	Rfx1(HTH)/NPC-H3K4me1-ChIP-Seq(GSE16256)	1.00E-07	-1.84E+01
12	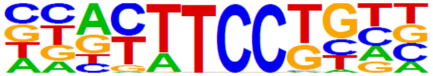	Etv2(ETS)/ES-ER71-ChIP-Seq(GSE59402)	1.00E-07	-1.71E+01
13	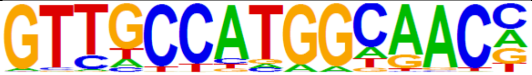	Rfx2(HTH)/LoVo-RFX2-ChIP-Seq(GSE49402)	1.00E-07	-1.67E+01

14	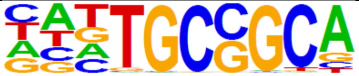	Zfp57(Zf)/H1-ZFP57.HA-ChIP-Seq(GSE115387)	1.00E-07	-1.67E+01
15	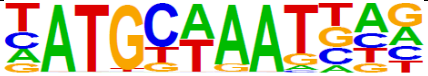	Brn1(POU,Homeobox)/NPC-Brn1-ChIP-Seq(GSE35496)	1.00E-06	-1.59E+01
16	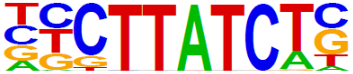	Gata2(Zf)/K562-GATA2-ChIP-Seq(GSE18829)	1.00E-06	-1.45E+01
17	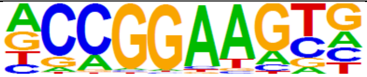	ETV4(ETS)/HepG2-ETV4-ChIP-Seq(ENCODE)	1.00E-06	-1.40E+01
18	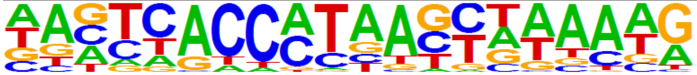	PSE(SNAPc)/K562-mStart-Seq	1.00E-05	-1.31E+01
19	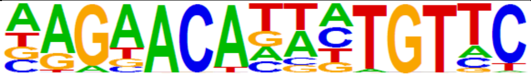	PGR(NR)/EndoStromal-PGR-ChIP-Seq(GSE69539)	1.00E-05	-1.27E+01
20	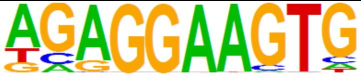	PU.1(ETS)/ThioMac-PU.1-ChIP-Seq(GSE21512)	1.00E-05	-1.24E+01
21	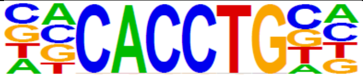	E2A(bHLH),near_PU.1/Bcell-PU.1-ChIP-Seq(GSE21512)	1.00E-05	-1.23E+01
22	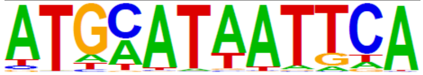	Pit1+1bp(Homeobox)/GCrat-Pit1-ChIP-Seq(GSE58009)	1.00E-05	-1.22E+01
23	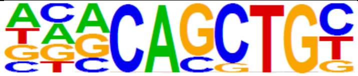	E2A(bHLH)/proBcell-E2A-ChIP-Seq(GSE21978)	1.00E-05	-1.20E+01
24	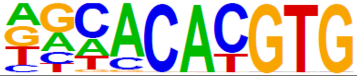	MNT(bHLH)/HepG2-MNT-ChIP-Seq(Encode)	1.00E-05	-1.19E+01
25	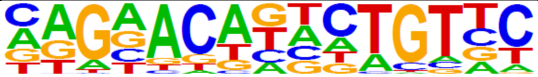	PR(NR)/T47D-PR-ChIP-Seq(GSE31130)	1.00E-05	-1.18E+01
26	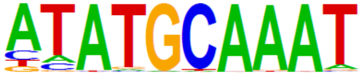	Oct2(POU,Homeobox)/Bcell-Oct2-ChIP-Seq(GSE21512)	1.00E-04	-1.13E+01
27	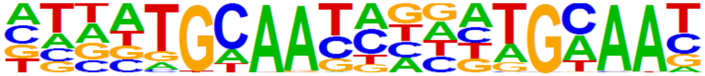	CEBP:CEBP(bZIP)/MEF-Chop-ChIP-Seq(GSE35681)	1.00E-04	-1.12E+01
28	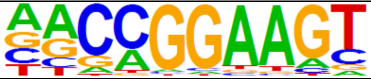	GABPA(ETS)/Jurkat-GABPa-ChIP-Seq(GSE17954)	1.00E-04	-1.12E+01

29	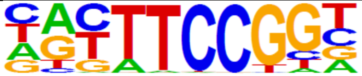	Elk4(ETS)/Hela-Elk4-ChIP-Seq(GSE31477)	1.00E-04	-1.10E+01
30	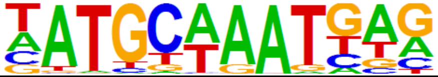	Oct6(POU,Homeobox)/NPC-Pou3f1-ChIP-Seq(GSE35496)	1.00E-04	-1.09E+01
31	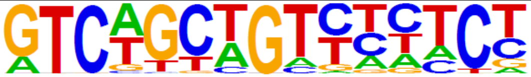	ZNF317(Zf)/HEK293-ZNF317.GFP-ChIP-Seq(GSE58341)	1.00E-04	-1.07E+01
32	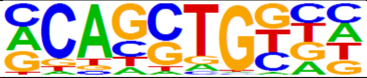	HEB(bHLH)/mES-Heb-ChIP-Seq(GSE53233)	1.00E-04	-1.05E+01
33	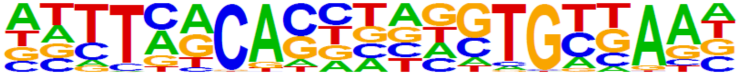	Brachyury(T-box)/Mesoendoderm-Brachyury-ChIP-exo(GSE54963)	1.00E-04	-9.99E+00
34	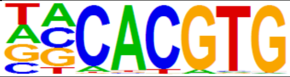	NPAS(bHLH)/Liver-NPAS-ChIP-Seq(GSE39860)	1.00E-04	-9.92E+00
35	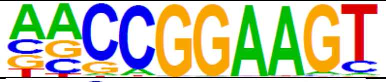	ETS(ETS)/Promoter	1.00E-04	-9.41E+00
36	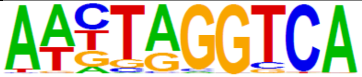	RORgt(NR)/EL4-RORgt.Flag-ChIP-Seq(GSE56019)	1.00E-04	-9.28E+00
37	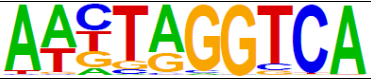	RORgt(NR)/EL4-RORgt.Flag-ChIP-Seq(GSE56019)	1.00E-04	-9.28E+00
38	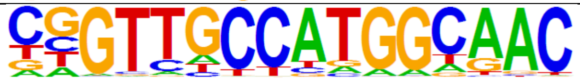	RFX(HTH)/K562-RFX3-ChIP-Seq(SRA012198)	1.00E-03	-9.17E+00
39	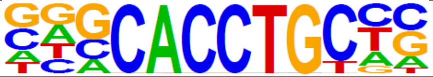	Slug(Zf)/Mesoderm-Snai2-ChIP-Seq(GSE61475)	1.00E-03	-8.72E+00
40	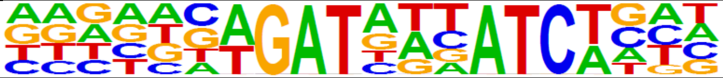	GATA(Zf),IR3/iTreg-Gata3-ChIP-Seq(GSE20898)	1.00E-03	-8.70E+00
41	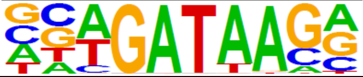	Gata4(Zf)/Heart-Gata4-ChIP-Seq(GSE35151)	1.00E-03	-8.30E+00
42	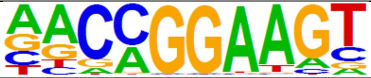	ETV1(ETS)/GIST48-ETV1-ChIP-Seq(GSE22441)	1.00E-03	-8.10E+00
43	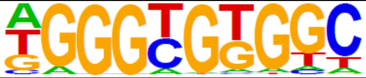	KLF5(Zf)/LoVo-KLF5-ChIP-Seq(GSE49402)	1.00E-03	-8.06E+00

44	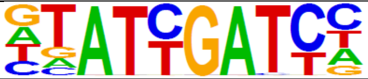	HNF6(Homeobox)/Liver-Hnf6-ChIP-Seq(ERP000394)	1.00E-03	-7.99E+00
45	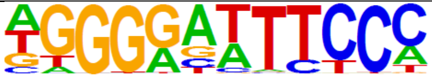	NFkB-p65(RHD)/GM12787-p65-ChIP-Seq(GSE19485)	1.00E-03	-7.97E+00
46	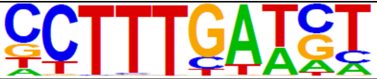	LEF1(HMG)/H1-LEF1-ChIP-Seq(GSE64758)	1.00E-03	-7.91E+00
47	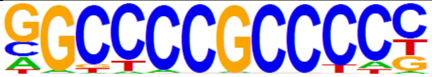	Sp1(Zf)/Promoter	1.00E-03	-7.52E+00
48	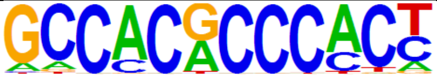	Klf9(Zf)/GBM-Klf9-ChIP-Seq(GSE62211)	1.00E-03	-7.21E+00
49	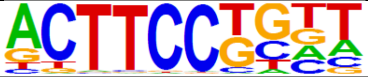	Elf4(ETS)/BMDM-Elf4-ChIP-Seq(GSE88699)	1.00E-03	-7.13E+00
50	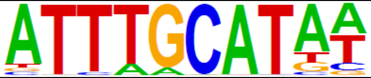	Oct4(POU,Homeobox)/mES-Oct4-ChIP-Seq(GSE11431)	1.00E-03	-7.13E+00
51	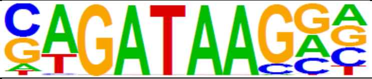	Gata1(Zf)/K562-GATA1-ChIP-Seq(GSE18829)	1.00E-03	-7.11E+00
52	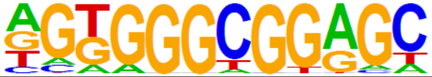	Sp5(Zf)/mES-Sp5.Flag-ChIP-Seq(GSE72989)	1.00E-03	-6.98E+00
53	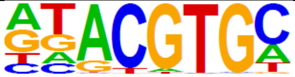	HIF-1b(HLH)/T47D-HIF1b-ChIP-Seq(GSE59937)	1.00E-03	-6.95E+00
54	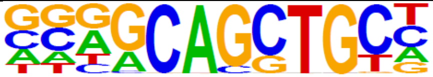	Ascl2(bHLH)/ESC-Ascl2-ChIP-Seq(GSE97712)	1.00E-02	-6.84E+00
55	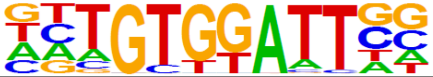	Foxh1(Forkhead)/hESC-FOXH1-ChIP-Seq(GSE29422)	1.00E-02	-6.79E+00
56	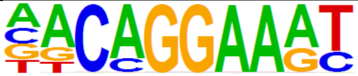	EWS:FLI1-fusion(ETS)/SK_N_MC-EWS:FLI1-ChIP-Seq(SRA014231)	1.00E-02	-6.77E+00
57	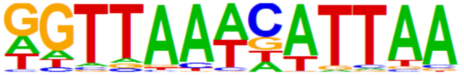	Hnf1(Homeobox)/Liver-Foxa2-Chip-Seq(GSE25694)	1.00E-02	-6.74E+00
58	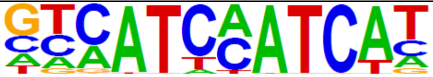	HOXA2(Homeobox)/mES-Hoxa2-ChIP-Seq(Donaldson_et_al.)	1.00E-02	-6.73E+00

59	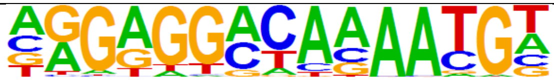	ZNF675(Zf)/HEK293-ZNF675.GFP-ChIP-Seq(GSE58341)	1.00E-02	-6.73E+00
60	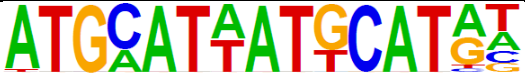	OCT:OCT-short(POU,Homeobox)/NPC-OCT6-ChIP-Seq(GSE43916)	1.00E-02	-6.65E+00
61	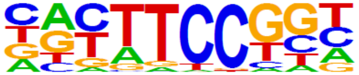	Fli1(ETS)/CD8-FLI-ChIP-Seq(GSE20898)	1.00E-02	-6.43E+00
62	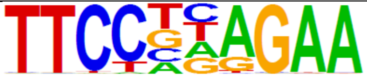	STAT6(Stat)/Macrophage-Stat6-ChIP-Seq(GSE38377)	1.00E-02	-6.30E+00
63	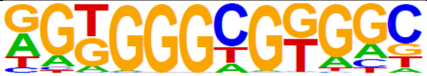	KLF14(Zf)/HEK293-KLF14.GFP-ChIP-Seq(GSE58341)	1.00E-02	-6.07E+00
64	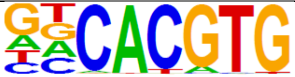	BMAL1(bHLH)/Liver-Bmal1-ChIP-Seq(GSE39860)	1.00E-02	-6.06E+00
65	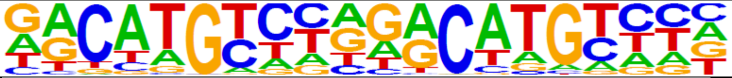	p53(p53)/Saos-p53-ChIP-Seq(GSE15780)	1.00E-02	-5.62E+00
66	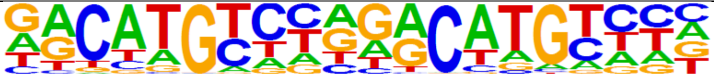	p53(p53)/Saos-p53-ChIP-Seq	1.00E-02	-5.62E+00
67	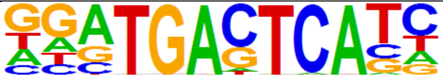	Fra2(bZIP)/Striatum-Fra2-ChIP-Seq(GSE43429)	1.00E-02	-5.46E+00
68	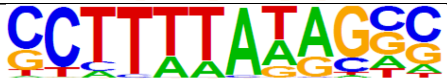	TATA-Box(TBP)/Promoter	1.00E-02	-5.35E+00
69	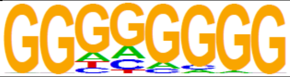	Maz(Zf)/HepG2-Maz-ChIP-Seq(GSE31477)	1.00E-02	-5.32E+00
70	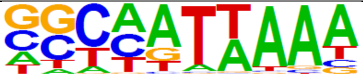	Unknown(Homeobox)/Limb-p300-ChIP-Seq	1.00E-02	-5.19E+00
71	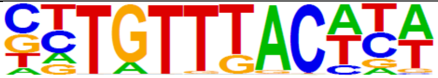	Foxa2(Forkhead)/Liver-Foxa2-ChIP-Seq(GSE25694)	1.00E-02	-5.07E+00
72	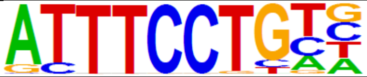	EWS:ERG-fusion(ETS)/CADO_ES1-EWS:ERG-ChIP-Seq(SRA014231)	1.00E-02	-4.96E+00
73	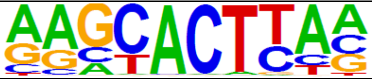	Nkx3.1(Homeobox)/LNCaP-Nkx3.1-ChIP-Seq(GSE28264)	1.00E-02	-4.95E+00

74	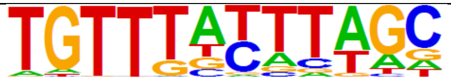	FoxD3(forkhead)/ZebrafishEmbryo-Foxd3.biotin-ChIP-seq(GSE106676)	1.00E-02	-4.91E+00
75	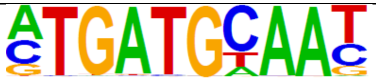	Atf4(bZIP)/MEF-Atf4-ChIP-Seq(GSE35681)	1.00E-02	-4.80E+00
76	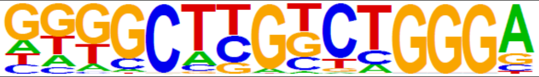	Zfp809(Zf)/ES-Zfp809-ChIP-Seq(GSE70799)	1.00E-02	-4.69E+00
77	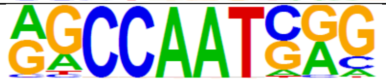	NFY(CCAAT)/Promoter	1.00E-02	-4.62E+00

Table 1j continued

Rank	q-value (Benjamini)	# Target Sequences with Motif	% of Targets Sequences with Motif	# Background Sequences with Motif	% of Background Sequences with Motif
1	0	41	3.40%	0.9	0.14%
2	0	35	2.90%	1	0.15%
3	0	172	14.27%	42.9	6.38%
4	0	110	9.13%	26.3	3.92%
5	0	35	2.90%	4.8	0.72%
6	0	19	1.58%	1.6	0.24%
7	0	21	1.74%	2.9	0.43%
8	0	29	2.41%	4.3	0.64%
9	0	15	1.24%	1.7	0.26%
10	0	14	1.16%	0.6	0.10%
11	0	27	2.24%	4.4	0.66%
12	0	111	9.21%	36.3	5.40%
13	0	13	1.08%	1	0.15%
14	0	13	1.08%	1.6	0.24%
15	0	68	5.64%	19.7	2.94%
16	0	95	7.88%	32	4.75%
17	0	101	8.38%	34.6	5.15%
18	0.0001	97	8.05%	33.5	4.98%
19	0.0001	43	3.57%	11.8	1.75%
20	0.0001	58	4.81%	17.3	2.58%

21	0.0001	146	12.12%	56.8	8.44%
22	0.0001	84	6.97%	28.8	4.28%
23	0.0001	143	11.87%	55.3	8.22%
24	0.0001	195	16.18%	80.4	11.95%
25	0.0001	265	21.99%	116	17.24%
26	0.0002	66	5.48%	21.5	3.20%
27	0.0002	18	1.49%	3.2	0.47%
28	0.0002	98	8.13%	35.8	5.33%
29	0.0003	46	3.82%	13.5	2.01%
30	0.0003	77	6.39%	26.1	3.87%
31	0.0003	14	1.16%	2.2	0.33%
32	0.0004	191	15.85%	80.1	11.91%
33	0.0006	37	3.07%	10.9	1.63%
34	0.0006	265	21.99%	118.8	17.67%
35	0.001	31	2.57%	8.4	1.25%
36	0.0011	13	1.08%	2.9	0.43%
37	0.0011	13	1.08%	2.9	0.43%
38	0.0012	9	0.75%	1	0.15%
39	0.0018	57	4.73%	19.9	2.96%
40	0.0018	19	1.58%	4.7	0.70%
41	0.0026	144	11.95%	60.6	9.01%
42	0.0031	127	10.54%	52.1	7.74%
43	0.0032	106	8.80%	42.7	6.35%
44	0.0033	78	6.47%	30	4.46%
45	0.0033	44	3.65%	14.2	2.11%
46	0.0034	80	6.64%	31	4.61%
47	0.0049	8	0.66%	1.4	0.20%
48	0.0066	23	1.91%	6.2	0.92%
49	0.0068	91	7.55%	36.9	5.48%
50	0.0068	91	7.55%	36.4	5.42%
51	0.0069	76	6.31%	29.9	4.45%
52	0.0077	56	4.65%	20.3	3.02%
53	0.0078	160	13.28%	70.2	10.43%
54	0.0085	109	9.05%	45.3	6.74%
55	0.0088	88	7.30%	35.5	5.27%

56	0.0088	60	4.98%	22.1	3.29%
57	0.0089	25	2.07%	7	1.04%
58	0.0089	17	1.41%	4.6	0.68%
59	0.0089	17	1.41%	4.7	0.70%
60	0.0092	159	13.20%	70.3	10.46%
61	0.0113	116	9.63%	49.1	7.30%
62	0.0126	59	4.90%	22.3	3.32%
63	0.0158	127	10.54%	55.4	8.23%
64	0.0158	275	22.82%	131	19.48%
65	0.0236	13	1.08%	3.4	0.50%
66	0.0236	13	1.08%	3.4	0.50%
67	0.0272	44	3.65%	16.8	2.50%
68	0.0299	166	13.78%	76.7	11.40%
69	0.0303	84	6.97%	35.1	5.22%
70	0.0341	108	8.96%	47.7	7.09%
71	0.038	159	13.20%	74	11.00%
72	0.0419	87	7.22%	37.3	5.55%
73	0.0419	296	24.56%	145.3	21.59%
74	0.0427	121	10.04%	54.6	8.12%
75	0.0468	36	2.99%	13.4	2.00%
76	0.0519	12	1.00%	3.4	0.51%
77	0.0545	90	7.47%	39.8	5.92%

k

Rank	Motif	Name	P-value	log P-pvalue
1	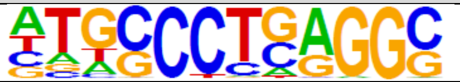	AP-2alpha(AP2)/Hela-AP2alpha-ChIP-Seq(GSE31477)	1.00E-12	-2.84E+01
2	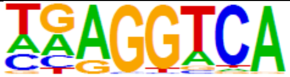	THRb(NR)/Liver-NR1A2-ChIP-Seq(GSE52613)	1.00E-08	-2.00E+01

3	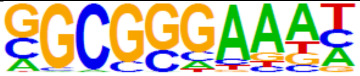	E2F4(E2F)/K562-E2F4-ChIP-Seq(GSE31477)	1.00E-08	-1.95E+01
4	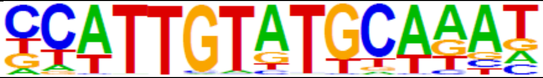	Oct4:Sox17(POU,Homeobox,HMG)/F9-Sox17-ChIP-Seq(GSE44553)	1.00E-05	-1.30E+01
5	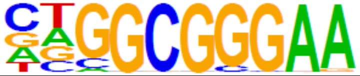	E2F1(E2F)/Hela-E2F1-ChIP-Seq(GSE22478)	1.00E-05	-1.27E+01
6	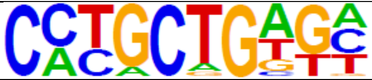	Zic(Zf)/Cerebellum-ZIC1.2-ChIP-Seq(GSE60731)	1.00E-05	-1.18E+01
7	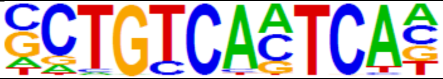	Pbx3(Homeobox)/GM12878-PBX3-ChIP-Seq(GSE32465)	1.00E-05	-1.17E+01
8	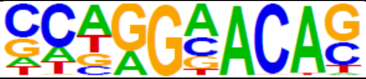	AR-halfsite(NR)/LNCaP-AR-ChIP-Seq(GSE27824)	1.00E-05	-1.16E+01
9	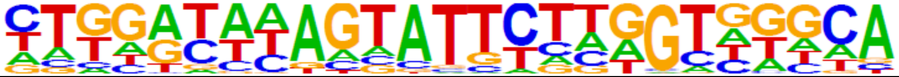	ZNF136(Zf)/HEK293-ZNF136.GFP-ChIP-Seq(GSE58341)	1.00E-04	-1.12E+01
10	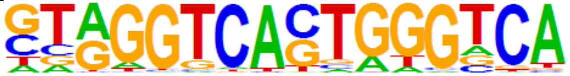	Reverb(NR),DR2/RAW-Reverba.biotin-ChIP-Seq(GSE45914)	1.00E-04	-9.45E+00
11	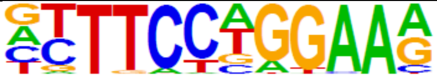	STAT4(Stat)/CD4-Stat4-ChIP-Seq(GSE22104)	1.00E-03	-8.60E+00
12	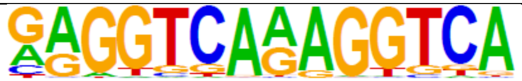	TR4(NR),DR1/Hela-TR4-ChIP-Seq(GSE24685)	1.00E-03	-8.31E+00
13	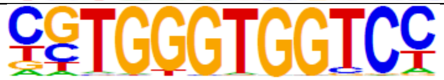	GLI3(Zf)/Limb-GLI3-ChIP-Chip(GSE11077)	1.00E-03	-8.29E+00
14	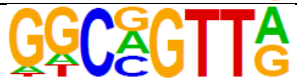	MYB(HTH)/ERMYB-Myb-ChIPSeq(GSE22095)	1.00E-03	-8.17E+00
15	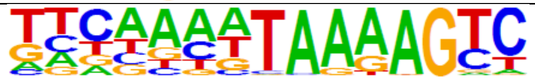	MYNN(Zf)/HEK293-MYNN.eGFP-ChIP-Seq(Encode)	1.00E-03	-8.17E+00
16	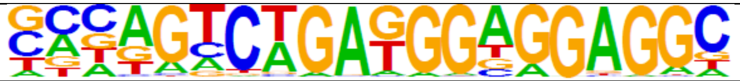	ZSCAN22(Zf)/HEK293-ZSCAN22.GFP-ChIP-Seq(GSE58341)	1.00E-03	-8.10E+00
17	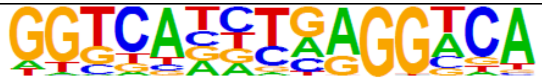	THRa(NR)/C17.2-THRa-ChIP-Seq(GSE38347)	1.00E-03	-7.91E+00

18	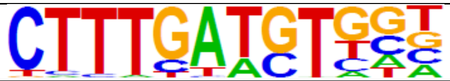	Tcf7(HMG)/GM12878-TCF7-ChIP-Seq(Encode)	1.00E-03	-7.86E+00
19	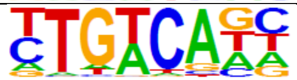	Tgif1(Homeobox)/mES-Tgif1-ChIP-Seq(GSE55404)	1.00E-03	-7.71E+00
20	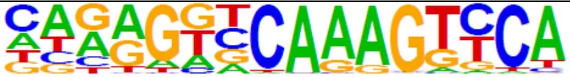	HNF4a(NR),DR1/HepG2-HNF4a-ChIP-Seq(GSE25021)	1.00E-03	-7.50E+00
21	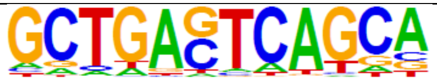	MafK(bZIP)/C2C12-MafK-ChIP-Seq(GSE36030)	1.00E-03	-7.38E+00
22	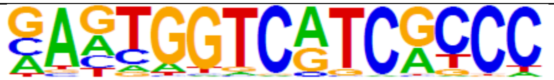	ZNF669(Zf)/HEK293-ZNF669.GFP-ChIP-Seq(GSE58341)	1.00E-03	-7.18E+00
23	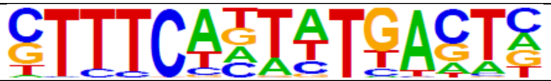	IRF:BATF(IRF:bZIP)/pDC-Irf8-ChIP-Seq(GSE66899)	1.00E-03	-7.16E+00
24	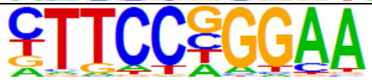	Stat3(Stat)/mES-Stat3-ChIP-Seq(GSE11431)	1.00E-03	-7.00E+00
25	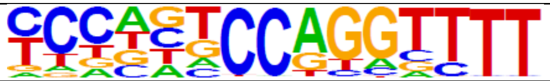	PRDM15(Zf)/ESC-Prdm15-ChIP-Seq(GSE73694)	1.00E-03	-6.95E+00
26	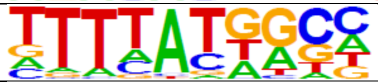	Hoxa11(Homeobox)/ChickenMSG-Hoxa11.Flag-ChIP-Seq(GSE86088)	1.00E-02	-6.71E+00
27	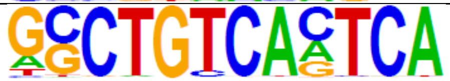	PBX1(Homeobox)/MCF7-PBX1-ChIP-Seq(GSE28007)	1.00E-02	-6.69E+00
28	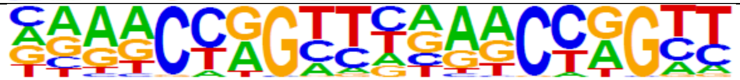	Tcfcp2l1(CP2)/mES-Tcfcp2l1-ChIP-Seq(GSE11431)	1.00E-02	-6.56E+00
29	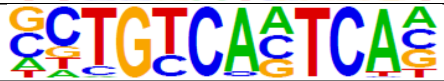	Pknox1(Homeobox)/ES-Prep1-ChIP-Seq(GSE63282)	1.00E-02	-6.40E+00
30	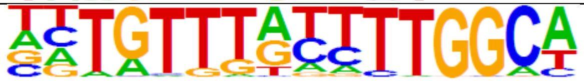	NF1:FOXA1(CTF,Forkhead)/LNCAP-FOXA1-ChIP-Seq(GSE27824)	1.00E-02	-6.33E+00
31	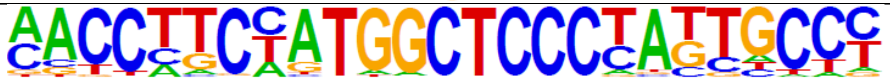	ZNF16(Zf)/HEK293-ZNF16.GFP-ChIP-Seq(GSE58341)	1.00E-02	-6.33E+00
32	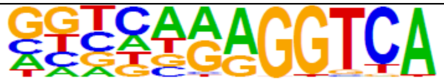	COUP-TFII(NR)/K562-NR2F1-ChIP-Seq(Encode)	1.00E-02	-6.19E+00

33	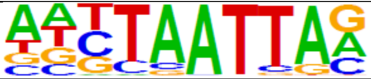	Lhx3(Homeobox)/Neuron-Lhx3-ChIP-Seq(GSE31456)	1.00E-02	-6.16E+00
34	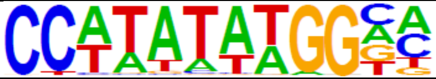	CArG(MADS)/PUER-Srf-ChIP-Seq(Sullivan_et_al.)	1.00E-02	-6.15E+00
35	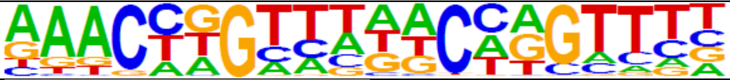	GRHL2(CP2)/HBE-GRHL2-ChIP-Seq(GSE46194)	1.00E-02	-6.07E+00
36	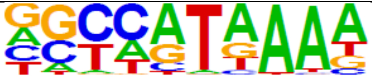	Hoxd11(Homeobox)/ChickenMSG-Hoxd11.Flag-ChIP-Seq(GSE86088)	1.00E-02	-5.76E+00
37	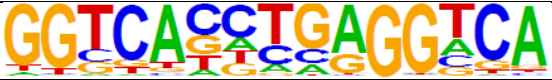	THRb(NR)/HepG2-THRb.Flag-ChIP-Seq(Encode)	1.00E-02	-5.72E+00
38	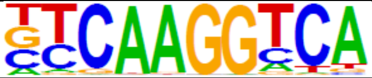	Nr5a2(NR)/Pancreas-LRH1-ChIP-Seq(GSE34295)	1.00E-02	-5.71E+00
39	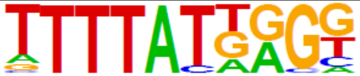	HOXB13(Homeobox)/ProstateTumor-HOXB13-ChIP-Seq(GSE56288)	1.00E-02	-5.65E+00
40	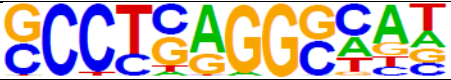	AP-2gamma(AP2)/MCF7-TFAP2C-ChIP-Seq(GSE21234)	1.00E-02	-5.57E+00
41	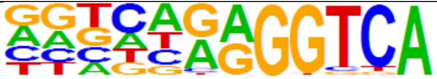	EAR2(NR)/K562-NR2F6-ChIP-Seq(Encode)	1.00E-02	-5.45E+00
42	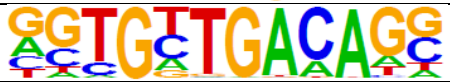	Tbx20(T-box)/Heart-Tbx20-ChIP-Seq(GSE29636)	1.00E-02	-5.42E+00
43	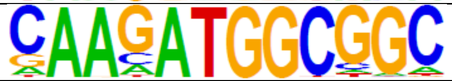	YY1(Zf)/Promoter	1.00E-02	-5.39E+00
44	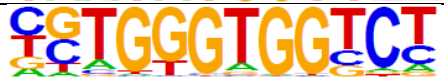	Gli2(Zf)/GM2-Gli2-ChIP-Chip(GSE112702)	1.00E-02	-5.32E+00
45	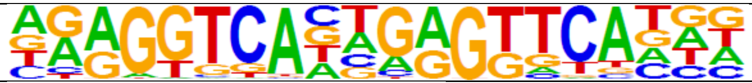	VDR(NR),DR3/GM10855-VDR+vitD-ChIP-Seq(GSE22484)	1.00E-02	-5.20E+00
46	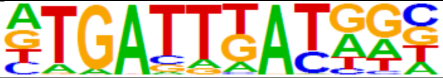	PBX2(Homeobox)/K562-PBX2-ChIP-Seq(Encode)	1.00E-02	-4.75E+00

Table 1k continued

Rank	q-value (Benjamini)	# Target Sequences with Motif	% of Targets Sequences with Motif	# Background Sequences with Motif	% of Background Sequences with Motif
1	0	74	7.91%	34.1	3.08%
2	0	438	46.84%	413.5	37.39%
3	0	14	1.50%	3	0.27%
4	0.0002	17	1.82%	5.3	0.48%
5	0.0003	8	0.86%	0	0.00%
6	0.0005	88	9.41%	64.5	5.84%
7	0.0005	21	2.25%	8.9	0.81%
8	0.0005	399	42.67%	396.4	35.85%
9	0.0007	12	1.28%	3.6	0.32%
10	0.0034	19	2.03%	8.8	0.79%
11	0.0071	98	10.48%	80.3	7.26%
12	0.0088	6	0.64%	1.3	0.12%
13	0.0088	15	1.60%	6.7	0.61%
14	0.0088	141	15.08%	125.2	11.32%
15	0.0088	27	2.89%	16	1.44%
16	0.0088	10	1.07%	3.9	0.35%
17	0.0092	72	7.70%	56.5	5.11%
18	0.0092	35	3.74%	22.2	2.01%
19	0.0101	318	34.01%	320.4	28.98%
20	0.0118	49	5.24%	35.4	3.20%
21	0.0127	21	2.25%	11	0.99%
22	0.0148	11	1.18%	4.5	0.40%
23	0.0148	14	1.50%	7	0.63%
24	0.0163	47	5.03%	34.1	3.08%
25	0.0165	89	9.52%	75.5	6.83%
26	0.0201	238	25.45%	235.2	21.26%
27	0.0201	9	0.96%	3.4	0.30%
28	0.0216	12	1.28%	5.8	0.53%
29	0.0245	16	1.71%	8.9	0.80%
30	0.0247	5	0.53%	0.4	0.03%
31	0.0247	5	0.53%	0.5	0.05%
32	0.0274	186	19.89%	180.5	16.32%
33	0.0274	161	17.22%	153.1	13.85%

34	0.0274	29	3.10%	19.3	1.75%
35	0.0282	41	4.39%	30.9	2.80%
36	0.0376	235	25.13%	236.7	21.40%
37	0.0379	72	7.70%	61.2	5.54%
38	0.0379	68	7.27%	57.2	5.18%
39	0.0385	101	10.80%	91.5	8.28%
40	0.0406	95	10.16%	85.4	7.72%
41	0.0448	163	17.43%	158.3	14.32%
42	0.0451	21	2.25%	13.4	1.22%
43	0.0456	8	0.86%	3.2	0.29%
44	0.0474	31	3.32%	22.5	2.04%
45	0.0524	23	2.46%	15.1	1.36%
46	0.0803	81	8.66%	73.2	6.62%

l

Order	Term	Database	Overlap	p value	Adjusted p value
1	Prion disease pathway WP3995	WikiPathways_2019_Human	3/33	9.94E-06	2.64E-03
2	Co-Translational ER Protein Import	Elsevier_Pathway_Collection	2/6	2.48E-05	2.64E-03
3	Endogenous Peptide Antigen Presentation	Elsevier_Pathway_Collection	2/6	2.48E-05	2.64E-03
4	Protein processing in the endoplasmic reticulum	BioPlanet_2019	4/148	3.25E-05	2.64E-03
5	Protein processing in endoplasmic reticulum	KEGG_2019_Human	4/151	3.52E-05	2.64E-03
6	antigen processing and presentation	BioCarta_2015	3/55	4.70E-05	2.94E-03
7	Antigen processing and presentation	KEGG_2019_Human	3/60	6.11E-05	3.10E-03
8	Antigen processing and presentation	BioPlanet_2019	3/64	7.41E-05	3.10E-03
9	ATF6-alpha activates chaperone genes Homo sapiens R-HSA-381183	Reactome_2016	2/10	7.43E-05	3.10E-03
10	Activation of chaperones by ATF6 alpha	BioPlanet_2019	2/12	1.09E-04	3.71E-03
11	ATF6-alpha activates chaperones Homo sapiens R-HSA-381033	Reactome_2016	2/12	1.09E-04	3.71E-03
12	Protein Folding	Elsevier_Pathway_Collection	2/13	1.28E-04	4.01E-03
13	Electron Transport Chain (OXPHOS system in mitochondria) WP111	WikiPathways_2019_Human	3/87	1.85E-04	5.34E-03
14	Binding and Uptake of Ligands by Scavenger Receptors Homo sapiens R-HSA-2173782	Reactome_2016	2/19	2.80E-04	7.51E-03
15	Oxidative phosphorylation	KEGG_2019_Human	3/106	3.32E-04	8.31E-03
16	Proteins Involved in Cholesteatoma	Elsevier_Pathway_Collection	2/22	3.78E-04	8.40E-03

17	Parkinson disease	KEGG_2019_Human	3/111	3.81E-04	8.40E-03
18	SERPINA1 Associated Liver Damage	Elsevier_Pathway_Collection	2/23	4.14E-04	8.45E-03
19	Antigen Presentation: Folding, assembly and peptide loading of class I MHC Homo sapiens R-HSA-983170	Reactome_2016	2/24	4.51E-04	8.45E-03
20	Photodynamic therapy-induced unfolded protein response WP3613	WikiPathways_2019_Human	2/24	4.51E-04	8.45E-03
21	Apoptosis of Renal Tubule Epithelial Cell in Pyelonephritis	Elsevier_Pathway_Collection	2/29	6.61E-04	1.18E-02
22	ER Stress (Unfolded Protein Response)	Elsevier_Pathway_Collection	2/31	7.56E-04	1.28E-02
23	Alzheimer disease	KEGG_2019_Human	3/142	7.83E-04	1.28E-02
24	Huntington disease	KEGG_2019_Human	3/158	1.07E-03	1.64E-02
25	Proteins Involved in Myocardial Ischemia	Elsevier_Pathway_Collection	3/161	1.13E-03	1.64E-02
26	Apoptotic Keratinocytes Clearance Recession in Systemic Lupus Erythematosus	Elsevier_Pathway_Collection	2/38	1.14E-03	1.64E-02
27	Thyroid hormone synthesis	KEGG_2019_Human	2/44	1.52E-03	2.11E-02
28	Thermogenesis	KEGG_2019_Human	3/185	1.68E-03	2.26E-02
29	Complex I biogenesis Homo sapiens R-HSA-6799198	Reactome_2016	2/49	1.89E-03	2.44E-02
30	Mitochondrial complex I assembly model OXPHOS system WP4324	WikiPathways_2019_Human	2/50	1.96E-03	2.45E-02
31	IL-17 signaling pathway	KEGG_2019_Human	2/55	2.37E-03	2.87E-02
32	Response to elevated platelet cytosolic calcium	BioPlanet_2019	2/56	2.46E-03	2.88E-02
33	Platelet degranulation Homo sapiens R-HSA-114608	Reactome_2016	2/65	3.30E-03	3.64E-02
34	NOD signaling pathway	BioPlanet_2019	2/66	3.40E-03	3.64E-02
35	Myocardial Infarction	Elsevier_Pathway_Collection	2/66	3.40E-03	3.64E-02
36	Respiratory electron transport, ATP biosynthesis by chemiosmotic coupling, and heat production by uncoupling proteins	BioPlanet_2019	2/67	3.50E-03	3.64E-02
37	Response to elevated platelet cytosolic Ca2+ Homo sapiens R-HSA-76005	Reactome_2016	2/69	3.71E-03	3.76E-02
38	Electron transport chain	BioPlanet_2019	2/71	3.92E-03	3.87E-02
39	T cell receptor regulation of apoptosis	BioPlanet_2019	4/529	4.14E-03	3.98E-02
40	Prostate cancer	BioPlanet_2019	2/75	4.36E-03	3.99E-02
41	Unfolded protein response	BioPlanet_2019	2/75	4.36E-03	3.99E-02
42	Prostate cancer	KEGG_2019_Human	2/78	4.71E-03	4.21E-02
43	Unfolded Protein Response (UPR) Homo sapiens R-HSA-381119	Reactome_2016	2/81	5.07E-03	4.42E-02
44	Estrogen signaling pathway	KEGG_2019_Human	2/84	5.44E-03	4.64E-02
45	Cellular response to heat stress Homo sapiens R-HSA-3371556	Reactome_2016	2/86	5.70E-03	4.75E-02
46	Respiratory electron transport Homo sapiens R-HSA-611105	Reactome_2016	2/88	5.96E-03	4.86E-02
47	Retrograde endocannabinoid signaling	KEGG_2019_Human	2/90	6.23E-03	4.97E-02

Table 1l continued

Order	Genes	Upregulated	Downregulated	Gro
1	*HSP90B1, HSPA5, PDIA3*	0	3	1
2	*HSP90B1, HSPA5*	0	2	2
3	*HSPA5, PDIA3*	0	2	3
4	*HSP90B1, HSPA5, PDIA3, HSP90AA1*	0	4	1
5	*HSP90B1, HSPA5, PDIA3, HSP90AA1*	0	4	1
6	*HSPA5, PDIA3, HSP90AA1*	0	3	1
7	*HSPA5, PDIA3, HSP90AA1*	0	3	1
8	*HSPA5, PDIA3, HSP90AA1*	0	3	1
9	*HSP90B1, HSPA5*	0	2	2
10	*HSP90B1, HSPA5*	0	2	2
11	*HSP90B1, HSPA5*	0	2	2
12	*HSP90B1, HSP90AA1*	0	2	4
13	*NDUFA12, NDUFB4, ATP5F1C*	0	3	5
14	*HSP90B1, HSP90AA1*	0	2	4
15	*NDUFA12, NDUFB4, ATP5F1C*	0	3	5
16	*HSP90B1, HSPA5*	0	2	2
17	*NDUFA12, NDUFB4, ATP5F1C*	0	3	5
18	*HSP90B1, HSPA5*	0	2	2
19	*HSPA5, PDIA3*	0	2	3
20	*HSP90B1, HSPA5*	0	2	2
21	*HSP90B1, HSPA5*	0	2	2
22	*HSPA5, HSP90AA1*	0	2	6
23	*NDUFA12, NDUFB4, ATP5F1C*	0	3	5
24	*NDUFA12, NDUFB4, ATP5F1C*	0	3	5
25	*TMSB4X, TXNIP, HSP90AA1*	0	3	7
26	*HSP90B1, TXNIP*	0	2	8
27	*HSP90B1, HSPA5*	0	2	2
28	*NDUFA12, NDUFB4, ATP5F1C*	0	3	5
29	*NDUFA12, NDUFB4*	0	2	9
30	*NDUFA12, NDUFB4*	0	2	9
31	*HSP90B1, HSP90AA1*	0	2	4
32	*TMSB4X, HSPA5*	0	2	10
33	*TMSB4X, HSPA5*	0	2	10
34	*HSP90B1, HSP90AA1*	0	2	4

35	*HSP90B1, HSPA5*	0	2	2
36	*NDUFA12, NDUFB4*	0	2	9
37	*TMSB4X, HSPA5*	0	2	10
38	*NDUFA12, NDUFB4*	0	2	9
39	*HSP90B1, HSPA5, ARHGDIB, HSP90AA1*	0	4	11
40	*HSP90B1, HSP90AA1*	0	2	4
41	*HSP90B1, HSPA5*	0	2	2
42	*HSP90B1, HSP90AA1*	0	2	4
43	*HSP90B1, HSPA5*	0	2	2
44	*HSP90B1, HSP90AA1*	0	2	4
45	*HSPA5, HSP90AA1*	0	2	6
46	*NDUFA12, NDUFB4*	0	2	9
47	*NDUFA12, NDUFB4*	0	2	9

m

Order	Term	Database	Overlap	p value	Adjusted p value
1	Lipid and lipoprotein metabolism	BioPlanet_2019	13/372	2.86E-09	5.90E-07
2	SREBF and miR-33 in cholesterol and lipid homeostasis	BioPlanet_2019	5/15	3.57E-09	5.90E-07
3	SREBF and miR33 in cholesterol and lipid homeostasis WP2011	WikiPathways_2019_Human	5/15	3.57E-09	5.90E-07
4	Metabolism of lipids and lipoproteins Homo sapiens R-HSA-556833	Reactome_2016	14/485	7.08E-09	8.77E-07
5	Fatty acid, triacylglycerol, and ketone body metabolism	BioPlanet_2019	8/148	1.90E-07	1.88E-05
6	Sterol Regulatory Element-Binding Proteins (SREBP) signalling WP1982	WikiPathways_2019_Human	6/62	2.41E-07	1.99E-05
7	Fatty acid, triacylglycerol, and ketone body metabolism Homo sapiens R-HSA-535734	Reactome_2016	8/177	7.49E-07	5.31E-05
8	Fatty Acid Metabolism	MSigDB_Hallmark_2020	7/133	1.45E-06	8.99E-05
9	Cholesterol biosynthesis	BioPlanet_2019	4/23	2.61E-06	1.33E-04
10	srebp control of lipid synthesis	BioCarta_2015	3/7	2.68E-06	1.33E-04
11	Regulation of cholesterol biosynthesis by SREBP (SREBF) Homo sapiens R-HSA-1655829	Reactome_2016	5/55	3.64E-06	1.64E-04
12	SREBP control of lipid biosynthesis	BioPlanet_2019	3/8	4.27E-06	1.76E-04
13	Cholesterol Homeostasis	MSigDB_Hallmark_2020	5/68	1.05E-05	3.99E-04
14	mTORC1 Signaling	MSigDB_Hallmark_2020	7/194	1.77E-05	6.26E-04
15	Activation of gene expression by SREBF (SREBP) Homo sapiens R-HSA-2426168	Reactome_2016	4/42	3.10E-05	1.02E-03
16	Androgen Response	MSigDB_Hallmark_2020	5/86	3.30E-05	1.02E-03

17	Mitochondrial LC-Fatty Acid Beta-Oxidation WP368	WikiPathways_2019_Human	3/16	4.17E-05	1.22E-03
18	TNF-alpha Signaling via NF-kB	MSigDB_Hallmark_2020	6/169	8.24E-05	2.27E-03
19	Mitochondrial fatty acid beta-oxidation	BioPlanet_2019	3/21	9.75E-05	2.54E-03
20	Cholesterol biosynthesis Homo sapiens R-HSA-191273	Reactome_2016	3/22	1.13E-04	2.63E-03
21	Metabolism Homo sapiens R-HSA-1430728	Reactome_2016	16/1398	1.27E-04	2.63E-03
22	HNF3B pathway	BioPlanet_2019	3/23	1.29E-04	2.63E-03
23	superpathway of cholesterol biosynthesis Homo sapiens PWY66-5	HumanCyc_2016	3/23	1.29E-04	2.63E-03
24	FOXA2 and FOXA3 transcription factor networks Homo sapiens b6933be8-6192-11e5-8ac5-06603eb7f303	NCI-Nature_2016	3/23	1.29E-04	2.63E-03
25	Proteins Involved in non-Alcoholic Fatty Liver Disease	Elsevier_Pathway_Collection	5/115	1.33E-04	2.63E-03
26	Metabolic Effects of Oncogenes and Tumor Suppressor in Cancer Cells	Elsevier_Pathway_Collection	4/64	1.64E-04	3.14E-03
27	Metabolism	BioPlanet_2019	14/1144	2.02E-04	3.71E-03
28	ceramide de novo biosynthesis Homo sapiens PWY3DJ-12	HumanCyc_2016	2/6	2.77E-04	4.79E-03
29	Fatty acid beta oxidation	BioPlanet_2019	3/30	2.90E-04	4.79E-03
30	Fatty Acid Beta Oxidation WP143	WikiPathways_2019_Human	3/30	2.90E-04	4.79E-03
31	Fatty acid metabolism	BioPlanet_2019	3/33	3.86E-04	5.63E-03
32	Fatty acid degradation	KEGG_2019_Human	3/33	3.86E-04	5.63E-03
33	Import of palmitoyl-CoA into the mitochondrial matrix	BioPlanet_2019	2/7	3.86E-04	5.63E-03
34	Mevalonate pathway WP3963	WikiPathways_2019_Human	2/7	3.86E-04	5.63E-03
35	Estrogen Response Early	MSigDB_Hallmark_2020	5/147	4.17E-04	5.91E-03
36	mevalonate pathway Homo sapiens PWY-922	HumanCyc_2016	2/8	5.13E-04	6.88E-03
37	Synthesis of UDP-N-acetyl-glucosamine Homo sapiens R-HSA-446210	Reactome_2016	2/8	5.13E-04	6.88E-03
38	RAGE pathway	BioPlanet_2019	3/38	5.87E-04	7.66E-03
39	Sphingolipid metabolism	KEGG_2019_Human	3/40	6.84E-04	8.69E-03
40	Oncostatin M	BioPlanet_2019	5/167	7.47E-04	8.83E-03
41	Lipid metabolism regulation by peroxisome proliferator-activated receptor alpha (PPAR-alpha)	BioPlanet_2019	4/95	7.48E-04	8.83E-03
42	PPARA activates gene expression Homo sapiens R-HSA-1989781	Reactome_2016	4/95	7.48E-04	8.83E-03
43	superpathway of geranylgeranyldiphosphate biosynthesis I (via mevalonate) Homo sapiens PWY-5910	HumanCyc_2016	2/10	8.21E-04	9.05E-03
44	Cholesterol biosynthesis Homo sapiens P00014	Panther_2016	2/10	8.21E-04	9.05E-03
45	Import of palmitoyl-CoA into the mitochondrial matrix Homo sapiens R-HSA-200425	Reactome_2016	2/10	8.21E-04	9.05E-03
46	Regulation of lipid metabolism by Peroxisome proliferator-activated receptor alpha (PPARalpha) Homo sapiens R-HSA-400206	Reactome_2016	4/98	8.40E-04	9.06E-03
47	Lipodystrophy, Familial Partial	Elsevier_Pathway_Collection	2/11	1.00E-03	1.06E-02
48	Terpenoid backbone biosynthesis	BioPlanet_2019	2/13	1.41E-03	1.46E-02
49	Oxidative Phosphorylation	MSigDB_Hallmark_2020	5/198	1.60E-03	1.62E-02
50	AMPK signaling	BioPlanet_2019	3/55	1.73E-03	1.69E-02

51	AMP-activated Protein Kinase (AMPK) Signaling WP1403	WikiPathways_2019_Human	3/55	1.73E-03	1.69E-02
52	Sphingolipid metabolism	BioPlanet_2019	3/56	1.83E-03	1.70E-02
53	Statin pathway	BioPlanet_2019	2/15	1.89E-03	1.70E-02
54	Cholesterol Biosynthesis Pathway WP197	WikiPathways_2019_Human	2/15	1.89E-03	1.70E-02
55	Statin Pathway WP430	WikiPathways_2019_Human	2/15	1.89E-03	1.70E-02
56	Sphingolipid metabolism Homo sapiens R-HSA-428157	Reactome_2016	3/58	2.02E-03	1.76E-02
57	Stimuli-sensing channels Homo sapiens R-HSA-2672351	Reactome_2016	3/58	2.02E-03	1.76E-02
58	Activated AMPK stimulation of fatty-acid oxidation in muscle	BioPlanet_2019	2/16	2.15E-03	1.81E-02
59	PPARGC1A Repression in Huntington Disease	Elsevier_Pathway_Collection	2/16	2.15E-03	1.81E-02
60	Terpenoid backbone biosynthesis	KEGG_2019_Human	2/20	3.37E-03	2.79E-02
61	Hyperglycemia and Hyperlipidemia Trigger beta-Cell Apoptosis	Elsevier_Pathway_Collection	2/22	4.08E-03	3.21E-02
62	Nitric Oxide Effects on beta-Cell	Elsevier_Pathway_Collection	2/22	4.08E-03	3.21E-02
63	Fatty Acid Biosynthesis WP357	WikiPathways_2019_Human	2/22	4.08E-03	3.21E-02
64	SREBP signaling	BioPlanet_2019	2/23	4.45E-03	3.45E-02
65	Proteins with Altered Expression in Cancer Metabolic Reprogramming	Elsevier_Pathway_Collection	3/78	4.69E-03	3.58E-02
66	Fatty acid biosynthesis	BioPlanet_2019	2/24	4.84E-03	3.58E-02
67	Photodynamic therapy-induced unfolded protein response WP3613	WikiPathways_2019_Human	2/24	4.84E-03	3.58E-02
68	Sphingolipid de novo biosynthesis	BioPlanet_2019	2/25	5.25E-03	3.77E-02
69	Sphingolipid pathway WP1422	WikiPathways_2019_Human	2/25	5.25E-03	3.77E-02
70	Phospholipid metabolism	BioPlanet_2019	4/164	5.50E-03	3.90E-02
71	Sphingolipid de novo biosynthesis Homo sapiens R-HSA-1660661	Reactome_2016	2/26	5.67E-03	3.93E-02
72	TGF-beta regulation of extracellular matrix	BioPlanet_2019	6/380	5.70E-03	3.93E-02
73	Proteins Involved in Alzheimer's Disease	Elsevier_Pathway_Collection	3/85	5.97E-03	4.05E-02
74	Lipogenesis/Lipolysis Activation in Cancer Cells and Cancer-Associated Adipocytes	Elsevier_Pathway_Collection	2/28	6.56E-03	4.40E-02
75	Lipid Metabolism Impairement in non-Alcoholic Fatty Liver Disease	Elsevier_Pathway_Collection	2/30	7.51E-03	4.96E-02
76	Lipid and lipoprotein metabolism	BioPlanet_2019	13/372	2.86E-09	5.90E-07

Table 1m continued

Order	Genes	Upregulated	Downregulated	Gro
1	*CPT1A, SLC25A20, ACADVL, LPCAT1, DHCR24, FASN, SREBF2, LDLR, HMGCR, UGCG, SPTLC2, KDSR, HMGCS1*	13	0	1
2	*FASN, SREBF2, LDLR, HMGCR, HMGCS1*	5	0	2
3	*FASN, SREBF2, LDLR, HMGCR, HMGCS1*	5	0	2
4	*CPT1A, SLC25A20, ACADVL, LPCAT1, DHCR24, FASN, SREBF2, LDLR, HMGCR, UGCG, SPTLC2, KDSR, HMGCS1, INSIG1*	14	0	1

5	*CPT1A, SLC25A20, ACADVL, LPCAT1, FASN, SREBF2, HMGCR, HMGCS1*	8	0	3
6	*FASN, SREBF2, LDLR, HMGCR, HMGCS1, INSIG1*	6	0	2
7	*CPT1A, SLC25A20, ACADVL, LPCAT1, FASN, SREBF2, HMGCR, HMGCS1*	8	0	3
8	*CPT1A, ACADVL, DHCR24, FASN, ACAA2, ETFDH, HMGCS1*	7	0	4
9	*DHCR24, SREBF2, HMGCR, HMGCS1*	4	0	5
10	*SREBF2, LDLR, HMGCS1*	3	0	6
11	*FASN, SREBF2, HMGCR, HMGCS1, INSIG1*	5	0	2
12	*SREBF2, LDLR, HMGCS1*	3	0	6
13	*FASN, SREBF2, LDLR, HMGCR, HMGCS1*	5	0	2
14	*DHCR24, LDLR, HMGCR, SQSTM1, HMGCS1, PPP1R15A, INSIG1*	7	0	7
15	*FASN, SREBF2, HMGCR, HMGCS1*	4	0	2
16	*DHCR24, HMGCR, ABHD2, HMGCS1, INSIG1*	5	0	8
17	*CPT1A, SLC25A20, ACADVL*	3	0	9
18	*SNN, LDLR, SQSTM1, B4GALT5, KLF10, PPP1R15A*	6	0	10
19	*CPT1A, SLC25A20, ACADVL*	3	0	9
20	*DHCR24, HMGCR, HMGCS1*	3	0	5
21	*CPT1A, SLC25A20, ACADVL, LPCAT1, DHCR24, FASN, SREBF2, LDLR, HMGCR, ETFDH, B4GALT5, UGCG, SPTLC2, KDSR, HMGCS1, INSIG1*	16	0	1
22	*CPT1A, ACADVL, HMGCS1*	3	0	11
23	*DHCR24, HMGCR, HMGCS1*	3	0	5
24	*CPT1A, ACADVL, HMGCS1*	3	0	11
25	*CPT1A, FASN, SREBF2, LDLR, INSIG1*	5	0	12
26	*FASN, SREBF2, LDLR, HMGCR*	4	0	2
27	*CPT1A, ACADVL, LPCAT1, DHCR24, FASN, LDLR, ACAA2, HMGCR, ETFDH, B4GALT5, UGCG, SPTLC2, KDSR, HMGCS1*	14	0	1
28	*SPTLC2, KDSR*	2	0	13
29	*CPT1A, SLC25A20, ACADVL*	3	0	9
30	*CPT1A, SLC25A20, ACADVL*	3	0	9
31	*CPT1A, ACADVL, ACAA2*	3	0	14
32	*CPT1A, ACADVL, ACAA2*	3	0	14
33	*CPT1A, SLC25A20*	2	0	15
34	*HMGCR, HMGCS1*	2	0	16
35	*FASN, FAM102A, ABHD2, UGCG, KLF10*	5	0	17
36	*HMGCR, HMGCS1*	2	0	16
37	*RENBP, AMDHD2*	2	0	18
38	*SREBF2, LDLR, HMGCR*	3	0	2
39	*UGCG, SPTLC2, KDSR*	3	0	19

40	*DHCR24, LDLR, HMGCR, HMGCS1, KLF10*	5	0	20
41	*CPT1A, SREBF2, HMGCR, HMGCS1*	4	0	21
42	*CPT1A, SREBF2, HMGCR, HMGCS1*	4	0	21
43	*HMGCR, HMGCS1*	2	0	16
44	*HMGCR, HMGCS1*	2	0	16
45	*CPT1A, SLC25A20*	2	0	15
46	*CPT1A, SREBF2, HMGCR, HMGCS1*	4	0	21
47	*FASN, LDLR*	2	0	22
48	*HMGCR, HMGCS1*	2	0	16
49	*CPT1A, SLC25A20, ACADVL, ACAA2, ETFDH*	5	0	23
50	*CPT1A, FASN, HMGCR*	3	0	24
51	*CPT1A, FASN, HMGCR*	3	0	24
52	*UGCG, SPTLC2, KDSR*	3	0	19
53	*LDLR, HMGCR*	2	0	25
54	*HMGCR, HMGCS1*	2	0	16
55	*LDLR, HMGCR*	2	0	25
56	*UGCG, SPTLC2, KDSR*	3	0	19
57	*CLCN7, CLCN6, CLCN3*	3	0	26
58	*CPT1A, SLC25A20*	2	0	15
59	*HMGCR, HMGCS1*	2	0	16
60	*HMGCR, HMGCS1*	2	0	16
61	*CPT1A, ERN1*	2	0	27
62	*ERN1, PPP1R15A*	2	0	28
63	*FASN, ACAA2*	2	0	29
64	*SREBF2, INSIG1*	2	0	30
65	*LPCAT1, FASN, SREBF2*	3	0	31
66	*FASN, ACAA2*	2	0	29
67	*ERN1, PPP1R15A*	2	0	28
68	*SPTLC2, KDSR*	2	0	13
69	*SPTLC2, KDSR*	2	0	13
70	*LPCAT1, UGCG, SPTLC2, KDSR*	4	0	19
71	*SPTLC2, KDSR*	2	0	13
72	*SREBF2, HMGCR, UGCG, HMGCS1, KLF10, INSIG1*	6	0	32
73	*DHCR24, LDLR, HMGCR*	3	0	33
74	*LPCAT1, FASN*	2	0	34

75	*FASN, LDLR*	2	0	22
76	*CPT1A, SLC25A20, ACADVL, LPCAT1, DHCR24, FASN, SREBF2, LDLR, HMGCR, UGCG, SPTLC2, KDSR, HMGCS1*	13	0	1

n

Order	Term	Database	Overlap	p value	Adjusted p value
1	Cholesterol biosynthesis Homo sapiens P00014	Panther_2016	2/10	7.41E-05	1.05E-02
2	Cholesterol Biosynthesis Pathway WP197	WikiPathways_2019_Human	2/15	1.72E-04	1.05E-02
3	Statin pathway	BioPlanet_2019	2/16	1.97E-04	1.05E-02
4	Statin Pathway WP430	WikiPathways_2019_Human	2/16	1.97E-04	1.05E-02
5	Cholesterol biosynthesis Homo sapiens R-HSA-191273	Reactome_2016	2/22	3.77E-04	1.25E-02
6	Cholesterol biosynthesis	BioPlanet_2019	2/23	4.12E-04	1.25E-02
7	superpathway of cholesterol biosynthesis Homo sapiens PWY66-5	HumanCyc_2016	2/23	4.12E-04	1.25E-02
8	Activation of gene expression by SREBF (SREBP) Homo sapiens R-HSA-2426168	Reactome_2016	2/42	1.38E-03	3.68E-02

Table 1n continued

Order	Genes	Upregulated	Downregulated	Gro
1	*HMGCR, SQLE*	0	2	1
2	*HMGCR, SQLE*	0	2	2
3	*HMGCR, SQLE*	0	2	3
4	*HMGCR, SQLE*	0	2	4
5	*HMGCR, SQLE*	0	2	5
6	*HMGCR, SQLE*	0	2	6
7	*HMGCR, SQLE*	0	2	7
8	*HMGCR, SQLE*	0	2	8

o

Order	Term	Database	Overlap	p value	Adjusted p value
1	Estrogen Deficiency in Female Obesity	Elsevier_Pathway_Collection	3/6	6.12E-08	1.44E-05
2	Fatty acid, triacylglycerol, and ketone body metabolism Homo sapiens R-HSA-535734	Reactome_2016	6/175	1.37E-07	1.44E-05
3	Metabolism of lipids and lipoproteins Homo sapiens R-HSA-556833	Reactome_2016	8/481	1.51E-07	1.44E-05
4	Import of palmitoyl-CoA into the mitochondrial matrix Homo sapiens R-HSA-200425	Reactome_2016	3/10	3.66E-07	2.52E-05
5	Lipodystrophy, Familial Partial	Elsevier_Pathway_Collection	3/11	5.02E-07	2.52E-05
6	Lipid and lipoprotein metabolism	BioPlanet_2019	7/370	5.29E-07	2.52E-05
7	Mitochondrial LC-Fatty Acid Beta-Oxidation WP368	WikiPathways_2019_Human	3/16	1.70E-06	6.94E-05
8	Fatty acid, triacylglycerol, and ketone body metabolism	BioPlanet_2019	5/147	2.01E-06	7.17E-05
9	Ghrelin pathway	BioPlanet_2019	3/19	2.93E-06	9.31E-05
10	Mitochondrial fatty acid beta-oxidation	BioPlanet_2019	3/21	4.01E-06	1.11E-04
11	Lipogenesis Regulation in Adipocyte	Elsevier_Pathway_Collection	3/22	4.64E-06	1.11E-04
12	Fatty Acid Biosynthesis WP357	WikiPathways_2019_Human	3/22	4.64E-06	1.11E-04
13	Fatty acid biosynthesis	BioPlanet_2019	3/24	6.09E-06	1.34E-04
14	Adipocyte Hypertrophy and Hyperplasia	Elsevier_Pathway_Collection	3/25	6.91E-06	1.41E-04
15	Lipogenesis/Lipolysis Activation in Cancer Cells and Cancer-Associated Adipocytes	Elsevier_Pathway_Collection	3/28	9.82E-06	1.87E-04
16	Fatty acid beta oxidation	BioPlanet_2019	3/30	1.21E-05	1.89E-04
17	Lipid Metabolism Impairement in non-Alcoholic Fatty Liver Disease	Elsevier_Pathway_Collection	3/30	1.21E-05	1.89E-04
18	Fatty Acid Beta Oxidation WP143	WikiPathways_2019_Human	3/30	1.21E-05	1.89E-04
19	AMPK signaling pathway	KEGG_2019_Human	4/98	1.25E-05	1.89E-04
20	Fatty acid metabolism	BioPlanet_2019	3/33	1.63E-05	2.22E-04
21	Fatty acid degradation	KEGG_2019_Human	3/33	1.63E-05	2.22E-04
22	Liver X Receptor Pathway WP2874	WikiPathways_2019_Human	2/5	2.28E-05	2.84E-04
23	Proteins Involved in non-Alcoholic Fatty Liver Disease	Elsevier_Pathway_Collection	4/114	2.28E-05	2.84E-04
24	Activation of gene expression by SREBF (SREBP) Homo sapiens R-HSA-2426168	Reactome_2016	3/42	3.40E-05	4.05E-04
25	Import of palmitoyl-CoA into the mitochondrial matrix	BioPlanet_2019	2/7	4.77E-05	5.46E-04
26	reversal of insulin resistance by leptin	BioCarta_2015	2/8	6.36E-05	6.27E-04
27	Reversal of insulin resistance by leptin	BioPlanet_2019	2/8	6.36E-05	6.27E-04
28	AMPK Related Catabolism Deceleration in Glucose Insufficiency	Elsevier_Pathway_Collection	2/8	6.36E-05	6.27E-04
29	Leptin and adiponectin WP3934	WikiPathways_2019_Human	2/8	6.36E-05	6.27E-04
30	AMPK signaling	BioPlanet_2019	3/55	7.67E-05	7.08E-04
31	Regulation of cholesterol biosynthesis by SREBP (SREBF) Homo sapiens R-HSA-1655829	Reactome_2016	3/55	7.67E-05	7.08E-04

32	AMP-activated Protein Kinase (AMPK) Signaling WP1403	WikiPathways_2019_Human	3/56	8.10E-05	7.24E-04
33	Berardinelli-Seip Syndrome Progression (Hypothesis)	Elsevier_Pathway_Collection	2/10	1.02E-04	8.84E-04
34	Sterol Regulatory Element-Binding Proteins (SREBP) signalling WP1982	WikiPathways_2019_Human	3/62	1.10E-04	9.24E-04
35	Metabolic Effects of Oncogenes and Tumor Suppressor in Cancer Cells	Elsevier_Pathway_Collection	3/64	1.21E-04	9.87E-04
36	Familial Partial Lipodystrophy Type 2 Progression (Hypothesis)	Elsevier_Pathway_Collection	2/11	1.25E-04	9.89E-04
37	Androgen Induced Sebocyte Hyperfunction	Elsevier_Pathway_Collection	2/12	1.49E-04	1.15E-03
38	Familial Partial Lipodystrophy Type 4 Progression (Hypothesis)	Elsevier_Pathway_Collection	2/13	1.76E-04	1.33E-03
39	Oxidative Phosphorylation	MSigDB_Hallmark_2020	4/198	1.97E-04	1.44E-03
40	Proteins with Altered Expression in Cancer Metabolic Reprogramming	Elsevier_Pathway_Collection	3/76	2.02E-04	1.44E-03
41	SREBF and miR-33 in cholesterol and lipid homeostasis	BioPlanet_2019	2/15	2.37E-04	1.61E-03
42	SREBF and miR33 in cholesterol and lipid homeostasis WP2011	WikiPathways_2019_Human	2/15	2.37E-04	1.61E-03
43	Activated AMPK stimulation of fatty-acid oxidation in muscle	BioPlanet_2019	2/16	2.70E-04	1.80E-03
44	Thyroid Hormones in Adipose Tissue Metabolism	Elsevier_Pathway_Collection	2/20	4.27E-04	2.77E-03
45	Metabolism Homo sapiens R-HSA-1430728	Reactome_2016	8/1398	4.38E-04	2.78E-03
46	HNF3B pathway	BioPlanet_2019	2/23	5.67E-04	3.45E-03
47	FOXA2 and FOXA3 transcription factor networks Homo sapiens b6933be8-6192-11e5-8ac5-06603eb7f303	NCI-Nature_2016	2/23	5.67E-04	3.45E-03
48	RORA activates circadian expression	BioPlanet_2019	2/25	6.71E-04	4.00E-03
49	RORA activates gene expression Homo sapiens R-HSA-1368082	Reactome_2016	2/26	7.26E-04	4.24E-03
50	Fatty Acid Metabolism	MSigDB_Hallmark_2020	3/132	1.02E-03	5.80E-03
51	Fatty Acyl-CoA Biosynthesis Homo sapiens R-HSA-75105	Reactome_2016	2/31	1.03E-03	5.80E-03
52	Circadian rhythm related genes WP3594	WikiPathways_2019_Human	3/135	1.09E-03	5.99E-03
53	Donohue Syndrome Progression (Hypothesis)	Elsevier_Pathway_Collection	2/34	1.24E-03	6.71E-03
54	AMPK Signaling	Elsevier_Pathway_Collection	2/36	1.39E-03	7.39E-03
55	PPAR signaling pathway WP3942	WikiPathways_2019_Human	2/40	1.72E-03	8.95E-03
56	PPAR signaling pathway	BioPlanet_2019	2/43	1.99E-03	1.01E-02
57	Activation of chaperones by IRE1 alpha	BioPlanet_2019	2/45	2.17E-03	1.07E-02
58	Adipokines and Cytokines in Insulin Resistance in Skeletal Muscles	Elsevier_Pathway_Collection	2/45	2.17E-03	1.07E-02
59	PPAR signaling pathway	KEGG_2019_Human	2/46	2.27E-03	1.10E-02
60	Triglyceride Biosynthesis Homo sapiens R-HSA-75109	Reactome_2016	2/49	2.57E-03	1.21E-02
61	XBP1(S) activates chaperone genes Homo sapiens R-HSA-381038	Reactome_2016	2/49	2.57E-03	1.21E-02
62	IRE1alpha activates chaperones Homo sapiens R-HSA-381070	Reactome_2016	2/51	2.79E-03	1.28E-02
63	Circadian rhythm	BioPlanet_2019	2/54	3.12E-03	1.39E-02
64	Proteins Involved in Insulin Resistance	Elsevier_Pathway_Collection	2/54	3.12E-03	1.39E-02
65	Androgens in Sebocyte Maturation	Elsevier_Pathway_Collection	2/55	3.23E-03	1.42E-02
66	Circadian Clock Homo sapiens R-HSA-400253	Reactome_2016	2/56	3.35E-03	1.45E-02

67	Nuclear Receptors Meta-Pathway WP2882	WikiPathways_2019_Human	3/202	3.45E-03	1.47E-02
68	oleate biosynthesis Homo sapiens PWY-5996	HumanCyc_2016	1/3	4.70E-03	1.98E-02
69	Metabolism	BioPlanet_2019	6/1142	5.20E-03	2.15E-02
70	Unfolded protein response	BioPlanet_2019	2/75	5.93E-03	2.39E-02
71	Glucagon signaling pathway	KEGG_2019_Human	2/75	5.93E-03	2.39E-02
72	Beta-oxidation of myristoyl-CoA to lauroyl-CoA	BioPlanet_2019	1/4	6.26E-03	2.45E-02
73	VEGFR -> CTNND Signaling	Elsevier_Pathway_Collection	1/4	6.26E-03	2.45E-02
74	Unfolded Protein Response (UPR) Homo sapiens R-HSA-381119	Reactome_2016	2/81	6.89E-03	2.66E-02
75	Thermogenesis WP4321	WikiPathways_2019_Human	2/84	7.39E-03	2.82E-02
76	Acyl chain remodelling of phosphatidylserine	BioPlanet_2019	1/5	7.82E-03	2.86E-02
77	mitochondrial L-carnitine shuttle Homo sapiens PWY-6111	HumanCyc_2016	1/5	7.82E-03	2.86E-02
78	Acyl chain remodelling of PS Homo sapiens R-HSA-1482801	Reactome_2016	1/5	7.82E-03	2.86E-02
79	Insulin resistance	KEGG_2019_Human	2/87	7.91E-03	2.86E-02
80	Adipogenesis	BioPlanet_2019	2/92	8.82E-03	3.11E-02
81	Adipogenesis WP236	WikiPathways_2019_Human	2/92	8.82E-03	3.11E-02
82	Lipid metabolism regulation by peroxisome proliferator-activated receptor alpha (PPAR-alpha)	BioPlanet_2019	2/94	9.19E-03	3.12E-02
83	PPARA activates gene expression Homo sapiens R-HSA-1989781	Reactome_2016	2/94	9.19E-03	3.12E-02
84	ChREBP activates metabolic gene expression	BioPlanet_2019	1/6	9.37E-03	3.12E-02
85	ChREBP activates metabolic gene expression Homo sapiens R-HSA-163765	Reactome_2016	1/6	9.37E-03	3.12E-02
86	Scavenging by Class F Receptors Homo sapiens R-HSA-3000484	Reactome_2016	1/6	9.37E-03	3.12E-02
87	Regulation of lipid metabolism by Peroxisome proliferator-activated receptor alpha (PPARalpha) Homo sapiens R-HSA-400206	Reactome_2016	2/97	9.76E-03	3.21E-02
88	srebp control of lipid synthesis	BioCarta_2015	1/7	1.09E-02	3.36E-02
89	Mitochondrial beta-oxidation of saturated fatty acids	BioPlanet_2019	1/7	1.09E-02	3.36E-02
90	VEGFR -> FOXO3A Signaling	Elsevier_Pathway_Collection	1/7	1.09E-02	3.36E-02
91	Defective HLCS causes multiple carboxylase deficiency Homo sapiens R-HSA-3371599	Reactome_2016	1/7	1.09E-02	3.36E-02
92	Defects in biotin (Btn) metabolism Homo sapiens R-HSA-3323169	Reactome_2016	1/7	1.09E-02	3.36E-02
93	mitochondrial fatty acid beta-oxidation of saturated fatty acids Homo sapiens R-HSA-77286	Reactome_2016	1/7	1.09E-02	3.36E-02
94	Diabetes pathways	BioPlanet_2019	2/107	1.18E-02	3.59E-02
95	Angiopoietin Like Protein 8 Regulatory Pathway WP3915	WikiPathways_2019_Human	2/110	1.24E-02	3.64E-02
96	Fatty acid elongation in mitochondria	BioPlanet_2019	1/8	1.25E-02	3.64E-02
97	SREBP control of lipid biosynthesis	BioPlanet_2019	1/8	1.25E-02	3.64E-02
98	VEGFR -> STAT Signaling	Elsevier_Pathway_Collection	1/8	1.25E-02	3.64E-02
99	insulin signaling pathway	BioCarta_2015	2/111	1.26E-02	3.65E-02
100	Insulin signaling pathway	KEGG_2019_Human	2/115	1.35E-02	3.87E-02

101	PNPLA3 in non-Alcoholic Fatty Liver Disease	Elsevier_Pathway_Collection	1/10	1.56E-02	4.24E-02
102	VEGFR -> CTNNB Signaling	Elsevier_Pathway_Collection	1/10	1.56E-02	4.24E-02
103	bile acid biosynthesis, neutral pathway Homo sapiens PWY-6061	HumanCyc_2016	1/10	1.56E-02	4.24E-02
104	Fatty acid biosynthesis	KEGG_2019_Human	1/10	1.56E-02	4.24E-02
105	Acyl chain remodelling of PE Homo sapiens R-HSA-1482839	Reactome_2016	1/10	1.56E-02	4.24E-02
106	Fatty Acids Cause Oxidative Stress and Mitochondria Decline in Aging	Elsevier_Pathway_Collection	1/11	1.71E-02	4.58E-02
107	Biotin transport and metabolism Homo sapiens R-HSA-196780	Reactome_2016	1/11	1.71E-02	4.58E-02
108	Activation of matrix metalloproteinases	BioPlanet_2019	1/12	1.87E-02	4.81E-02
109	HDL-mediated lipid transport	BioPlanet_2019	1/12	1.87E-02	4.81E-02
110	Acyl chain remodelling of PC Homo sapiens R-HSA-1482788	Reactome_2016	1/12	1.87E-02	4.81E-02
111	Transcriptional cascade regulating adipogenesis WP4211	WikiPathways_2019_Human	1/12	1.87E-02	4.81E-02
112	TGF-beta regulation of extracellular matrix	BioPlanet_2019	3/381	1.97E-02	4.86E-02
113	ABCA transporters in lipid homeostasis	BioPlanet_2019	1/13	2.02E-02	4.86E-02
114	Acyl chain remodelling of phosphatidylcholine	BioPlanet_2019	1/13	2.02E-02	4.86E-02
115	Inhibitor of DNA binding (ID) signaling pathway	BioPlanet_2019	1/13	2.02E-02	4.86E-02
116	fatty acid &beta-oxidation Homo sapiens FAO-PWY	HumanCyc_2016	1/13	2.02E-02	4.86E-02
117	ABC transporters in lipid homeostasis Homo sapiens R-HSA-1369062	Reactome_2016	1/13	2.02E-02	4.86E-02
118	Activation of Matrix Metalloproteinases Homo sapiens R-HSA-1592389	Reactome_2016	1/13	2.02E-02	4.86E-02
119	ID signaling pathway WP53	WikiPathways_2019_Human	1/13	2.02E-02	4.86E-02

Table 1o continued

Order	Genes	Upregulated	Downregulated	Gro
1	*SREBF1, SCD, ACACA*	3	0	1
2	*CPT1A, SLC25A20, ACADVL, SREBF1, SCD, ACACA*	6	0	2
3	*CPT1A, SLC25A20, ACADVL, SREBF1, LPCAT3, ABCG1, SCD, ACACA*	8	0	2
4	*CPT1A, SLC25A20, ACACA*	3	0	3
5	*SREBF1, SCD, ACACA*	3	0	1
6	*CPT1A, SLC25A20, ACADVL, SREBF1, LPCAT3, ABCG1, ACACA*	7	0	2
7	*CPT1A, SLC25A20, ACADVL*	3	0	4
8	*CPT1A, SLC25A20, ACADVL, SREBF1, ACACA*	5	0	2
9	*SREBF1, ABCG1, ACACA*	3	0	5
10	*CPT1A, SLC25A20, ACADVL*	3	0	4
11	*SREBF1, SCD, ACACA*	3	0	1

12	*ACAA2, SCD, ACACA*	3	0	6
13	*ACAA2, SCD, ACACA*	3	0	6
14	*SREBF1, SCD, ACACA*	3	0	1
15	*SREBF1, SCD, ACACA*	3	0	1
16	*CPT1A, SLC25A20, ACADVL*	3	0	4
17	*SREBF1, SCD, ACACA*	3	0	1
18	*CPT1A, SLC25A20, ACADVL*	3	0	4
19	*CPT1A, SREBF1, SCD, ACACA*	4	0	1
20	*CPT1A, ACADVL, ACAA2*	3	0	4
21	*CPT1A, ACADVL, ACAA2*	3	0	4
22	*SREBF1, SCD*	2	0	7
23	*CPT1A, SREBF1, SCD, ACACA*	4	0	1
24	*SREBF1, SCD, ACACA*	3	0	1
25	*CPT1A, SLC25A20*	2	0	8
26	*CPT1A, ACACA*	2	0	9
27	*CPT1A, ACACA*	2	0	9
28	*SREBF1, ACACA*	2	0	10
29	*CPT1A, ACACA*	2	0	9
30	*CPT1A, SREBF1, ACACA*	3	0	1
31	*SREBF1, SCD, ACACA*	3	0	1
32	*CPT1A, SREBF1, ACACA*	3	0	1
33	*SREBF1, ACACA*	2	0	10
34	*SREBF1, SCD, ACACA*	3	0	1
35	*SREBF1, SCD, ACACA*	3	0	1
36	*SREBF1, ACACA*	2	0	10
37	*SREBF1, SCD*	2	0	7
38	*SREBF1, ACACA*	2	0	10
39	*CPT1A, SLC25A20, ACADVL, ACAA2*	4	0	4
40	*SREBF1, SCD, ACACA*	3	0	1
41	*SREBF1, SCD*	2	0	7
42	*SREBF1, SCD*	2	0	7
43	*CPT1A, SLC25A20*	2	0	8
44	*SREBF1, ACACA*	2	0	10
45	*CPT1A, SLC25A20, ACADVL, SREBF1, LPCAT3, ABCG1, SCD, ACACA*	8	0	2
46	*CPT1A, ACADVL*	2	0	11

47	*CPT1A, ACADVL*	2	0	11
48	*CPT1A, SREBF1*	2	0	12
49	*CPT1A, SREBF1*	2	0	12
50	*CPT1A, ACADVL, ACAA2*	3	0	4
51	*SCD, ACACA*	2	0	13
52	*CPT1A, SREBF1, KLF10*	3	0	14
53	*SREBF1, ACACA*	2	0	10
54	*SREBF1, ACACA*	2	0	10
55	*CPT1A, SCD*	2	0	15
56	*CPT1A, SCD*	2	0	15
57	*ACADVL, HYOU1*	2	0	16
58	*SREBF1, ACACA*	2	0	10
59	*CPT1A, SCD*	2	0	15
60	*SCD, ACACA*	2	0	13
61	*ACADVL, HYOU1*	2	0	16
62	*ACADVL, HYOU1*	2	0	16
63	*CPT1A, SREBF1*	2	0	12
64	*SREBF1, SCD*	2	0	7
65	*SREBF1, SCD*	2	0	7
66	*CPT1A, SREBF1*	2	0	12
67	*CPT1A, SREBF1, SCD*	3	0	1
68	*SCD*	1	0	17
69	*CPT1A, ACADVL, LPCAT3, ABCG1, ACAA2, ACACA*	6	0	18
70	*ACADVL, HYOU1*	2	0	16
71	*CPT1A, ACACA*	2	0	9
72	*ACADVL*	1	0	19
73	*COL18A1*	1	0	20
74	*ACADVL, HYOU1*	2	0	16
75	*CPT1A, SLC25A20*	2	0	8
76	*LPCAT3*	1	0	21
77	*CPT1A*	1	0	22
78	*LPCAT3*	1	0	21
79	*CPT1A, SREBF1*	2	0	12
80	*SREBF1, SCD*	2	0	7
81	*SREBF1, SCD*	2	0	7

82	*CPT1A, SREBF1*	2	0	12
83	*CPT1A, SREBF1*	2	0	12
84	*ACACA*	1	0	23
85	*ACACA*	1	0	23
86	*HYOU1*	1	0	24
87	*CPT1A, SREBF1*	2	0	12
88	*SREBF1*	1	0	25
89	*ACADVL*	1	0	19
90	*COL18A1*	1	0	20
91	*ACACA*	1	0	23
92	*ACACA*	1	0	23
93	*ACADVL*	1	0	19
94	*ACADVL, HYOU1*	2	0	16
95	*SREBF1, SCD*	2	0	7
96	*ACAA2*	1	0	26
97	*SREBF1*	1	0	25
98	*COL18A1*	1	0	20
99	*SREBF1, ACACA*	2	0	10
100	*SREBF1, ACACA*	2	0	10
101	*SREBF1*	1	0	25
102	*COL18A1*	1	0	20
103	*ACAA2*	1	0	26
104	*ACACA*	1	0	23
105	*LPCAT3*	1	0	21
106	*SREBF1*	1	0	25
107	*ACACA*	1	0	23
108	*COL18A1*	1	0	20
109	*ABCG1*	1	0	27
110	*LPCAT3*	1	0	21
111	*SREBF1*	1	0	25
112	*ABCG1, SCD, KLF10*	3	0	28
113	*ABCG1*	1	0	27
114	*LPCAT3*	1	0	21
115	*SREBF1*	1	0	25
116	*ACAA2*	1	0	26

117	*ABCG1*	1	0	27
118	*COL18A1*	1	0	20
119	*SREBF1*	1	0	25

p

Rank	Motif	Name	P-value	log P-pvalue
1	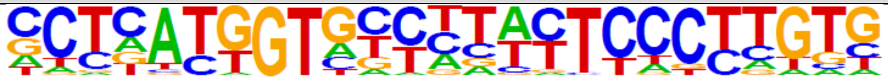	ZNF41(Zf)/HEK293-ZNF41.GFP-ChIP-Seq(GSE58341)/Homer	1.00E-02	-6.16E+00
2	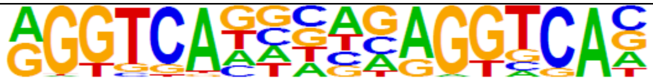	RAR:RXR(NR),DR5/ES-RAR-ChIP-Seq(GSE56893)/Homer	1.00E-02	-4.96E+00
3	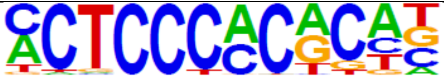	WT1(Zf)/Kidney-WT1-ChIP-Seq(GSE90016)/Homer	1.00E-02	-4.71E+00

Table 1p continued

Rank	q-value (Benjamini)	# Target Sequences with Motif	% of Targets Sequences with Motif	# Background Sequences with Motif	% of Background Sequences with Motif
1	0.9003	5	0.54%	6.4	0.10%
2	1	5	0.54%	8.3	0.13%
3	1	48	5.15%	228.8	3.60%

q

Rank	Motif	Name	P-value	log P-pvalue
1	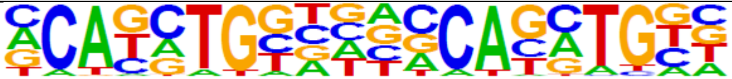	Twist(bHLH)/HMLE-TWIST1-ChIP-Seq(Chang_et_al)	1.00E-20	-4.79E+01

2	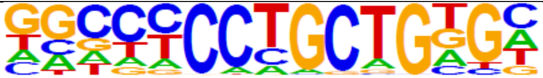	Zic3(Zf)/mES-Zic3-ChIP-Seq(GSE37889)	1.00E-20	-4.78E+01
3	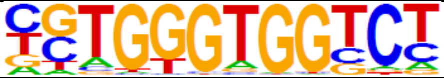	Gli2(Zf)/GM2-Gli2-ChIP-Chip(GSE112702)	1.00E-20	-4.62E+01
4	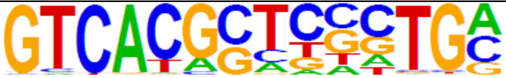	PAX5(Paired,Homeobox),condensed/GM12878-PAX5-ChIP-Seq(GSE32465)	1.00E-17	-4.13E+01
5	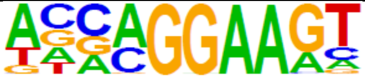	EHF(ETS)/LoVo-EHF-ChIP-Seq(GSE49402)	1.00E-16	-3.88E+01
6	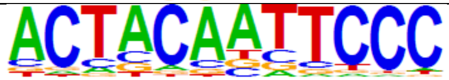	GFY(?)/Promoter	1.00E-11	-2.76E+01
7	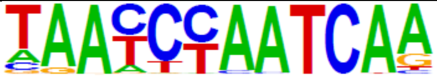	Duxbl(Homeobox)/NIH3T3-Duxbl.HA-ChIP-Seq(GSE119782)	1.00E-11	-2.58E+01
8	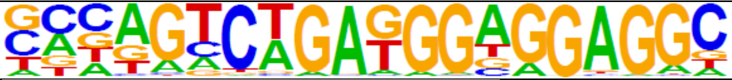	ZSCAN22(Zf)/HEK293-ZSCAN22.GFP-ChIP-Seq(GSE58341)	1.00E-10	-2.53E+01
9	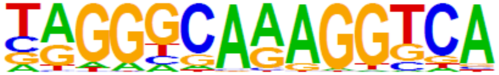	RXR(NR),DR1/3T3L1-RXR-ChIP-Seq(GSE13511)	1.00E-10	-2.49E+01
10	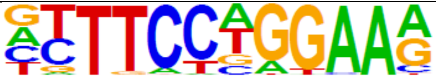	STAT4(Stat)/CD4-Stat4-ChIP-Seq(GSE22104)	1.00E-10	-2.46E+01
11	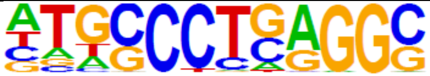	AP-2alpha(AP2)/Hela-AP2alpha-ChIP-Seq(GSE31477)	1.00E-10	-2.38E+01
12	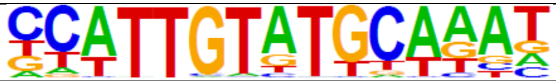	Oct4:Sox17(POU,Homeobox,HMG)/F9-Sox17-ChIP-Seq(GSE44553)	1.00E-09	-2.11E+01
13	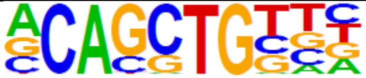	Ptf1a(bHLH)/Panc1-Ptf1a-ChIP-Seq(GSE47459)	1.00E-09	-2.09E+01
14	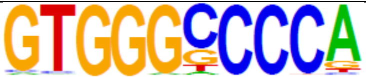	ZNF692(Zf)/HEK293-ZNF692.GFP-ChIP-Seq(GSE58341)	1.00E-08	-2.01E+01
15	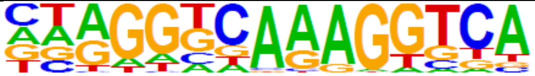	PPARa(NR),DR1/Liver-Ppara-ChIP-Seq(GSE47954)	1.00E-08	-1.97E+01
16	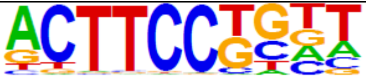	Elf4(ETS)/BMDM-Elf4-ChIP-Seq(GSE88699)	1.00E-08	-1.91E+01

17	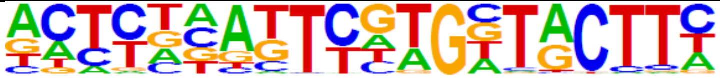	Mouse_Recombination_Hotspot(Zf)/Testis-DMC1-ChIP-Seq(GSE24438)	1.00E-08	-1.89E+01
18	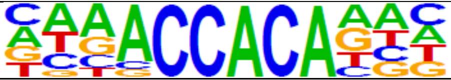	RUNX2(Runt)/PCa-RUNX2-ChIP-Seq(GSE33889)	1.00E-08	-1.88E+01
19	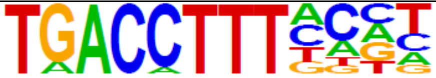	Nur77(NR)/K562-NR4A1-ChIP-Seq(GSE31363)	1.00E-07	-1.82E+01
20	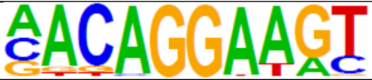	Ets1-distal(ETS)/CD4+-PolII-ChIP-Seq(Barski_et_al.)	1.00E-07	-1.74E+01
21	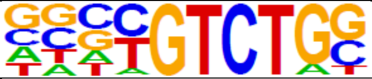	Smad4(MAD)/ESC-SMAD4-ChIP-Seq(GSE29422)	1.00E-07	-1.73E+01
22	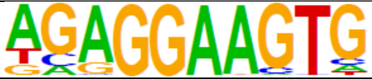	PU.1(ETS)/ThioMac-PU.1-ChIP-Seq(GSE21512)	1.00E-07	-1.71E+01
23	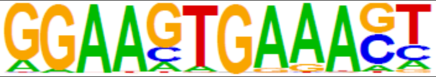	PU.1:IRF8(ETS:IRF)/pDC-Irf8-ChIP-Seq(GSE66899)	1.00E-07	-1.68E+01
24	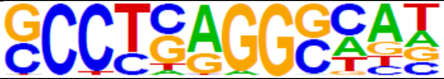	AP-2gamma(AP2)/MCF7-TFAP2C-ChIP-Seq(GSE21234)	1.00E-07	-1.63E+01
25	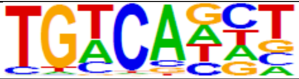	Tgif2(Homeobox)/mES-Tgif2-ChIP-Seq(GSE55404)	1.00E-07	-1.62E+01
26	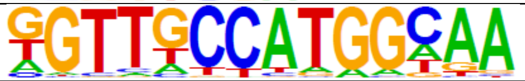	X-box(HTH)/NPC-H3K4me1-ChIP-Seq(GSE16256)	1.00E-06	-1.61E+01
27	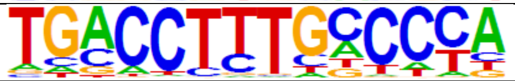	PPARE(NR),DR1/3T3L1-Pparg-ChIP-Seq(GSE13511)	1.00E-06	-1.51E+01
28	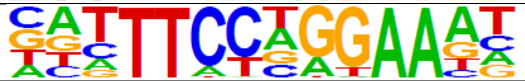	STAT1(Stat)/HelaS3-STAT1-ChIP-Seq(GSE12782)	1.00E-06	-1.51E+01
29	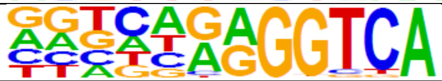	EAR2(NR)/K562-NR2F6-ChIP-Seq(Encode)	1.00E-06	-1.51E+01
30	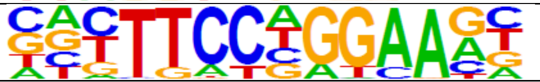	Stat3+il21(Stat)/CD4-Stat3-ChIP-Seq(GSE19198)	1.00E-06	-1.49E+01
31	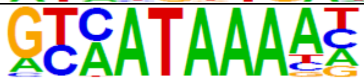	Cdx2(Homeobox)/mES-Cdx2-ChIP-Seq(GSE14586)	1.00E-06	-1.45E+01

32	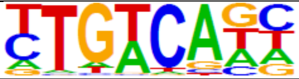	Tgif1(Homeobox)/mES-Tgif1-ChIP-Seq(GSE55404)	1.00E-06	-1.43E+01
33	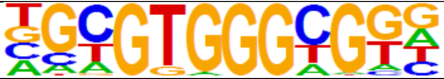	Egr2(Zf)/Thymocytes-Egr2-ChIP-Seq(GSE34254)	1.00E-06	-1.42E+01
34	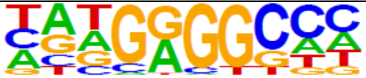	Zac1(Zf)/Neuro2A-Plagl1-ChIP-Seq(GSE75942)	1.00E-05	-1.34E+01
35	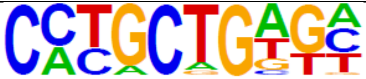	Zic(Zf)/Cerebellum-ZIC1.2-ChIP-Seq(GSE60731)	1.00E-05	-1.33E+01
36	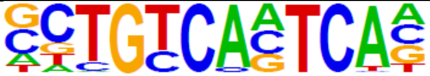	Pknox1(Homeobox)/ES-Prep1-ChIP-Seq(GSE63282)	1.00E-05	-1.33E+01
37	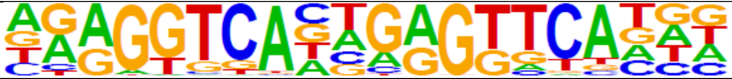	VDR(NR),DR3/GM10855-VDR+vitD-ChIP-Seq(GSE22484)	1.00E-05	-1.32E+01
38	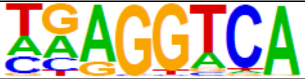	THRb(NR)/Liver-NR1A2-ChIP-Seq(GSE52613)	1.00E-05	-1.28E+01
39	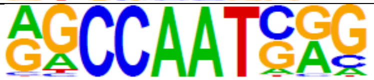	NFY(CCAAT)/Promoter	1.00E-05	-1.27E+01
40	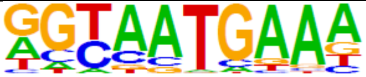	Hoxa10(Homeobox)/ChickenMSG-Hoxa10.Flag-ChIP-Seq(GSE86088)	1.00E-05	-1.18E+01
41	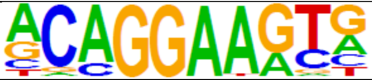	ERG(ETS)/VCaP-ERG-ChIP-Seq(GSE14097)	1.00E-05	-1.17E+01
42	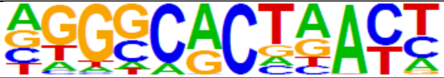	ZNF264(Zf)/HEK293-ZNF264.GFP-ChIP-Seq(GSE58341)	1.00E-05	-1.17E+01
43	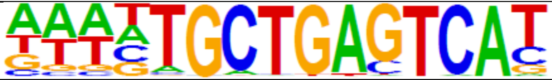	Bach1(bZIP)/K562-Bach1-ChIP-Seq(GSE31477)	1.00E-04	-1.14E+01
44	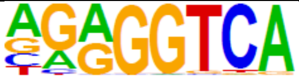	COUP-TFII(NR)/Artia-Nr2f2-ChIP-Seq(GSE46497)	1.00E-04	-1.13E+01
45	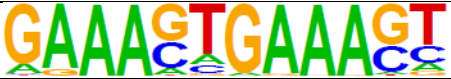	IRF1(IRF)/PBMC-IRF1-ChIP-Seq(GSE43036)	1.00E-04	-1.13E+01
46	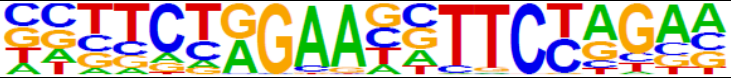	HRE(HSF)/HepG2-HSF1-ChIP-Seq(GSE31477)	1.00E-04	-1.12E+01

47	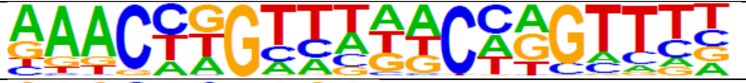	GRHL2(CP2)/HBE-GRHL2-ChIP-Seq(GSE46194)	1.00E-04	-1.09E+01
48	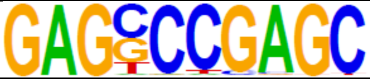	ZNF519(Zf)/HEK293-ZNF519.GFP-ChIP-Seq(GSE58341)	1.00E-04	-1.09E+01
49	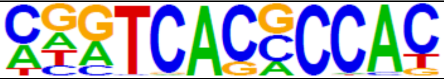	Srebp2(bHLH)/HepG2-Srebp2-ChIP-Seq(GSE31477)	1.00E-04	-1.07E+01
50	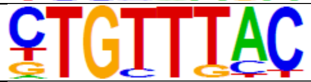	Foxo1(Forkhead)/RAW-Foxo1-ChIP-Seq(Fan_et_al.)	1.00E-04	-1.07E+01
51	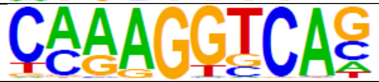	Erra(NR)/HepG2-Erra-ChIP-Seq(GSE31477)	1.00E-04	-1.07E+01
52	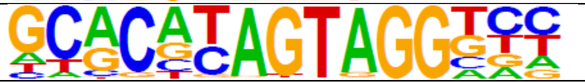	ZKSCAN1(Zf)/HepG2-ZKSCAN1-ChIP-Seq(Encode)	1.00E-04	-1.04E+01
53	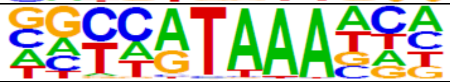	CDX4(Homeobox)/ZebrafishEmbryos-Cdx4.Myc-ChIP-Seq(GSE48254)	1.00E-04	-1.01E+01
54	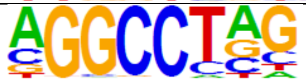	ZNF711(Zf)/SHSY5Y-ZNF711-ChIP-Seq(GSE20673)	1.00E-04	-1.01E+01
55	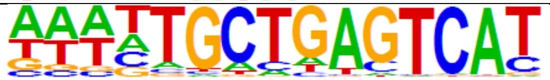	NFE2L2(bZIP)/HepG2-NFE2L2-ChIP-Seq(Encode)	1.00E-04	-1.01E+01
56	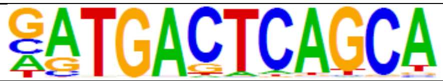	NF-E2(bZIP)/K562-NFE2-ChIP-Seq(GSE31477)	1.00E-04	-1.01E+01
57	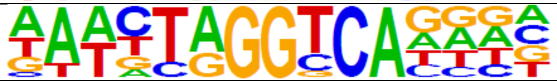	RORa(NR)/Liver-Rora-ChIP-Seq(GSE101115)	1.00E-04	-9.85E+00
58	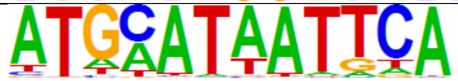	Pit1+1bp(Homeobox)/GCrat-Pit1-ChIP-Seq(GSE58009)	1.00E-04	-9.69E+00
59	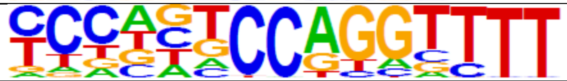	PRDM15(Zf)/ESC-Prdm15-ChIP-Seq(GSE73694)	1.00E-04	-9.64E+00
60	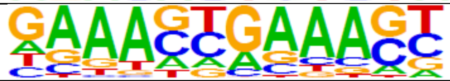	IRF2(IRF)/Erythroblas-IRF2-ChIP-Seq(GSE36985)	1.00E-04	-9.53E+00
61	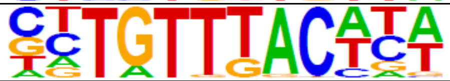	Foxa2(Forkhead)/Liver-Foxa2-ChIP-Seq(GSE25694)	1.00E-03	-9.19E+00

62	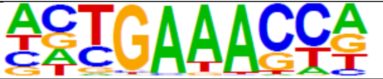	IRF4(IRF)/GM12878-IRF4-ChIP-Seq(GSE32465)	1.00E-03	-8.98E+00
63	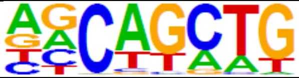	SCL(bHLH)/HPC7-Scl-ChIP-Seq(GSE13511)	1.00E-03	-8.95E+00
64	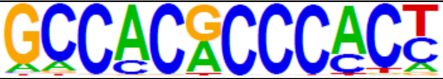	Klf9(Zf)/GBM-Klf9-ChIP-Seq(GSE62211)	1.00E-03	-8.75E+00
65	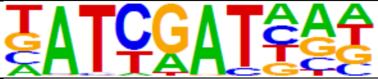	CUX1(Homeobox)/K562-CUX1-ChIP-Seq(GSE92882)	1.00E-03	-8.67E+00
66	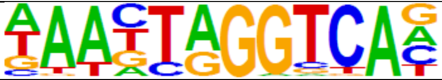	RORg(NR)/Liver-Rorc-ChIP-Seq(GSE101115)	1.00E-03	-8.47E+00
67	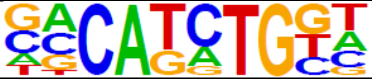	TCF4(bHLH)/SHSY5Y-TCF4-ChIP-Seq(GSE96915)	1.00E-03	-8.39E+00
68	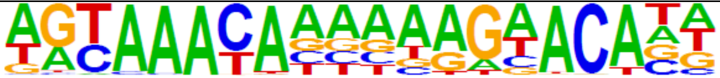	FOXA1:AR(Forkhead,NR)/LNCAP-AR-ChIP-Seq(GSE27824)	1.00E-03	-8.36E+00
69	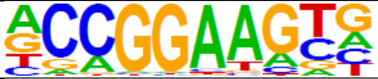	ETV4(ETS)/HepG2-ETV4-ChIP-Seq(ENCODE)	1.00E-03	-8.32E+00
70	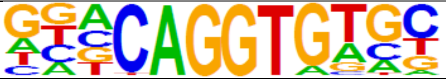	ZEB2(Zf)/SNU398-ZEB2-ChIP-Seq(GSE103048)	1.00E-03	-8.25E+00
71	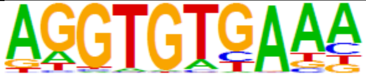	Tbx21(T-box)/GM12878-TBX21-ChIP-Seq(Encode)	1.00E-03	-8.06E+00
72	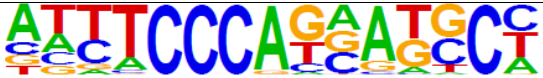	ZNF143|STAF(Zf)/CUTLL-ZNF143-ChIP-Seq(GSE29600)	1.00E-03	-7.96E+00
73	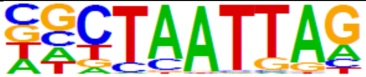	LXH9(Homeobox)/Hct116-LXH9.V5-ChIP-Seq(GSE116822)	1.00E-03	-7.84E+00
74	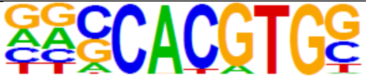	c-Myc(bHLH)/mES-cMyc-ChIP-Seq(GSE11431)	1.00E-03	-7.70E+00
75	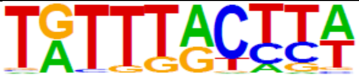	FOXM1(Forkhead)/MCF7-FOXM1-ChIP-Seq(GSE72977)	1.00E-03	-7.69E+00
76	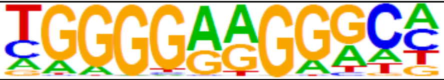	ZNF467(Zf)/HEK293-ZNF467.GFP-ChIP-Seq(GSE58341)	1.00E-03	-7.63E+00

77	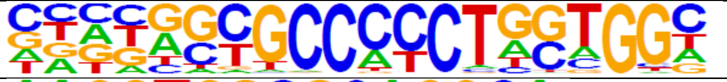	BORIS(Zf)/K562-CTCFL-ChIP-Seq(GSE32465)	1.00E-03	-7.62E+00
78	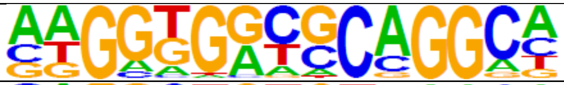	ZNF165(Zf)/WHIM12-ZNF165-ChIP-Seq(GSE65937)	1.00E-03	-7.62E+00
79	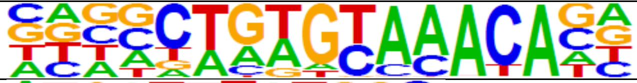	Fox:Ebox(Forkhead,bHLH)/Panc1-Foxa2-ChIP-Seq(GSE47459)	1.00E-03	-7.57E+00
80	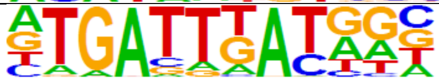	PBX2(Homeobox)/K562-PBX2-ChIP-Seq(Encode)	1.00E-03	-7.47E+00
81	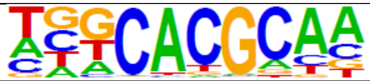	Arnt:Ahr(bHLH)/MCF7-Arnt-ChIP-Seq(Lo_et_al.)	1.00E-03	-7.46E+00
82	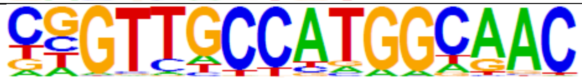	RFX(HTH)/K562-RFX3-ChIP-Seq(SRA012198)	1.00E-03	-7.44E+00
83	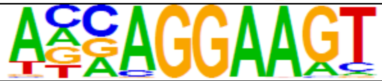	ELF3(ETS)/PDAC-ELF3-ChIP-Seq(GSE64557)	1.00E-03	-7.36E+00
84	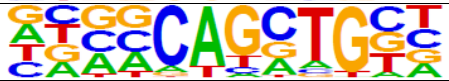	Ascl1(bHLH)/NeuralTubes-Ascl1-ChIP-Seq(GSE55840)	1.00E-03	-7.34E+00
85	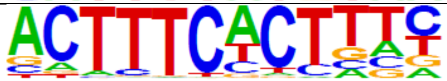	PRDM1(Zf)/Hela-PRDM1-ChIP-Seq(GSE31477)	1.00E-03	-7.21E+00
86	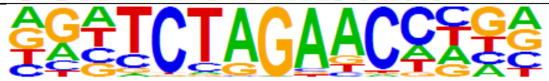	ZBTB12(Zf)/HEK293-ZBTB12.GFP-ChIP-Seq(GSE58341)	1.00E-03	-7.17E+00
87	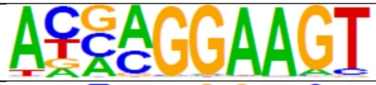	ELF5(ETS)/T47D-ELF5-ChIP-Seq(GSE30407)	1.00E-03	-7.06E+00
88	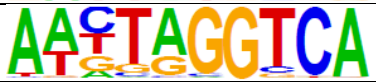	RORgt(NR)/EL4-RORgt.Flag-ChIP-Seq(GSE56019)	1.00E-03	-7.06E+00
89	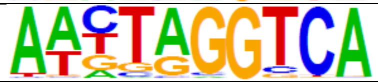	RORgt(NR)/EL4-RORgt.Flag-ChIP-Seq(GSE56019)	1.00E-03	-7.06E+00
90	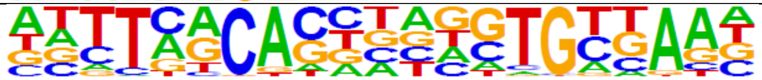	Brachyury(T-box)/Mesoendoderm-Brachyury-ChIP-exo(GSE54963)	1.00E-03	-6.94E+00
91	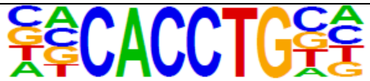	E2A(bHLH),near_PU.1/Bcell-PU.1-ChIP-Seq(GSE21512)	1.00E-02	-6.88E+00

92	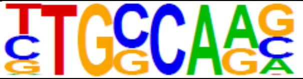	NF1-halfsite(CTF)/LNCaP-NF1-ChIP-Seq(Unpublished)	1.00E-02	-6.84E+00
93	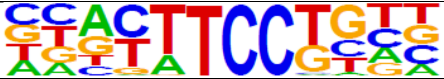	Etv2(ETS)/ES-ER71-ChIP-Seq(GSE59402)	1.00E-02	-6.81E+00
94	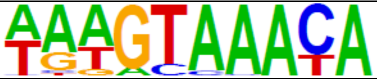	FOXA1(Forkhead)/MCF7-FOXA1-ChIP-Seq(GSE26831)	1.00E-02	-6.79E+00
95	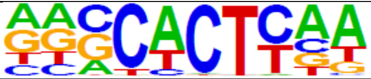	Nkx2.5(Homeobox)/HL1-Nkx2.5.biotin-ChIP-Seq(GSE21529)	1.00E-02	-6.79E+00
96	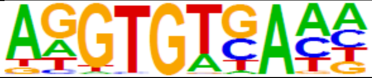	Tbet(T-box)/CD8-Tbet-ChIP-Seq(GSE33802)	1.00E-02	-6.72E+00
97	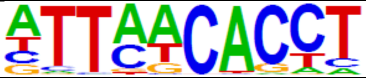	Eomes(T-box)/H9-Eomes-ChIP-Seq(GSE26097)	1.00E-02	-6.68E+00
98	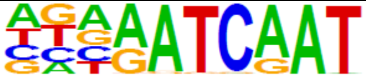	Cux2(Homeobox)/Liver-Cux2-ChIP-Seq(GSE35985)	1.00E-02	-6.65E+00
99	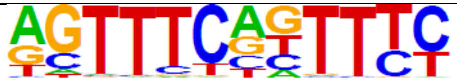	IRF3(IRF)/BMDM-Irf3-ChIP-Seq(GSE67343)	1.00E-02	-6.60E+00
100	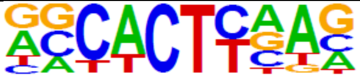	Nkx2.1(Homeobox)/LungAC-Nkx2.1-ChIP-Seq(GSE43252)	1.00E-02	-6.57E+00
101	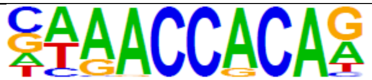	RUNX(Runt)/HPC7-Runx1-ChIP-Seq(GSE22178)	1.00E-02	-6.51E+00
102	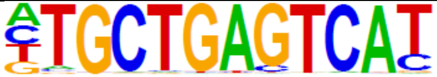	Nrf2(bZIP)/Lymphoblast-Nrf2-ChIP-Seq(GSE37589)	1.00E-02	-6.42E+00
103	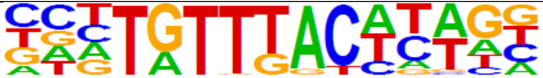	Foxa3(Forkhead)/Liver-Foxa3-ChIP-Seq(GSE77670)	1.00E-02	-6.31E+00
104	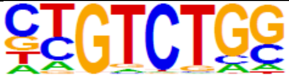	Smad2(MAD)/ES-SMAD2-ChIP-Seq(GSE29422)	1.00E-02	-6.09E+00
105	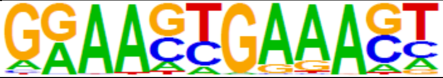	IRF8(IRF)/BMDM-IRF8-ChIP-Seq(GSE77884)	1.00E-02	-5.92E+00
106	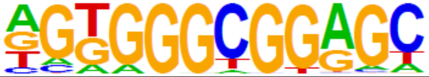	Sp5(Zf)/mES-Sp5.Flag-ChIP-Seq(GSE72989)	1.00E-02	-5.86E+00

107	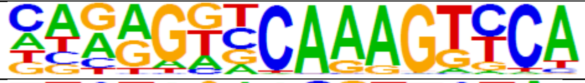	HNF4a(NR),DR1/HepG2-HNF4a-ChIP-Seq(GSE25021)	1.00E-02	-5.78E+00
108	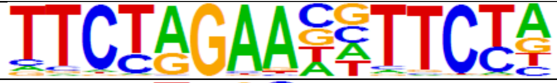	HRE(HSF)/Striatum-HSF1-ChIP-Seq(GSE38000)	1.00E-02	-5.76E+00
109	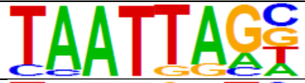	Lhx2(Homeobox)/HFSC-Lhx2-ChIP-Seq(GSE48068)	1.00E-02	-5.74E+00
110	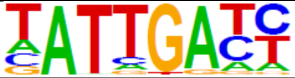	Hnf6b(Homeobox)/LNCaP-Hnf6b-ChIP-Seq(GSE106305)	1.00E-02	-5.67E+00
111	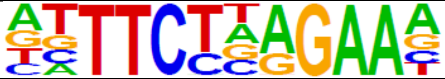	STAT5(Stat)/mCD4+-Stat5-ChIP-Seq(GSE12346)	1.00E-02	-5.64E+00
112	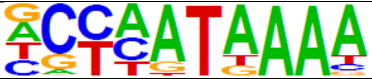	Hoxd13(Homeobox)/ChickenMSG-Hoxd13.Flag-ChIP-Seq(GSE86088)	1.00E-02	-5.49E+00
113	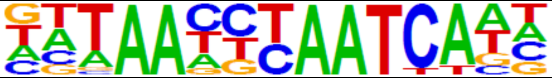	DUX4(Homeobox)/Myoblasts-DUX4.V5-ChIP-Seq(GSE75791)	1.00E-02	-5.36E+00
114	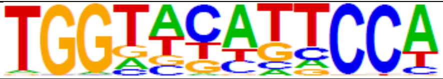	PRDM10(Zf)/HEK293-PRDM10.eGFP-ChIP-Seq(Encode)	1.00E-02	-5.30E+00
115	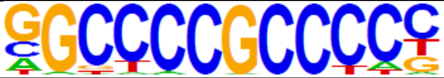	Sp1(Zf)/Promoter	1.00E-02	-5.28E+00
116	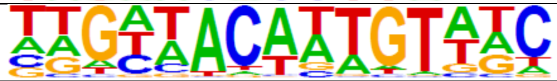	DMRT1(DM)/Testis-DMRT1-ChIP-Seq(GSE64892)	1.00E-02	-5.23E+00
117	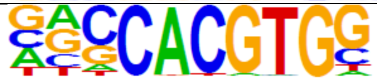	n-Myc(bHLH)/mES-nMyc-ChIP-Seq(GSE11431)	1.00E-02	-5.14E+00
118	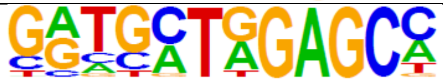	ZNF415(Zf)/HEK293-ZNF415.GFP-ChIP-Seq(GSE58341)	1.00E-02	-4.97E+00
119	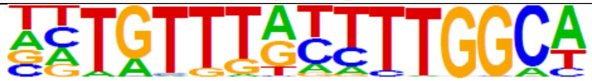	NF1:FOXA1(CTF,Forkhead)/LNCAP-FOXA1-ChIP-Seq(GSE27824)	1.00E-02	-4.94E+00
120	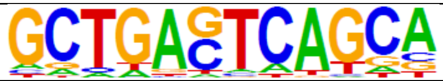	MafK(bZIP)/C2C12-MafK-ChIP-Seq(GSE36030)	1.00E-02	-4.87E+00
121	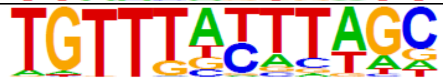	FoxD3(forkhead)/ZebrafishEmbryo-Foxd3.biotin-ChIP-seq(GSE106676)	1.00E-02	-4.83E+00

122	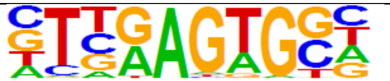	Nkx2.2(Homeobox)/NPC-Nkx2.2-ChIP-Seq(GSE61673)	1.00E-02	-4.81E+00
123	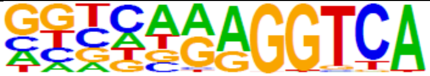	COUP-TFII(NR)/K562-NR2F1-ChIP-Seq(Encode)	1.00E-02	-4.78E+00
124	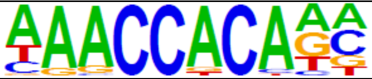	RUNX1(Runt)/Jurkat-RUNX1-ChIP-Seq(GSE29180)	1.00E-02	-4.76E+00
125	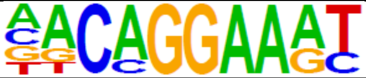	EWS:FLI1-fusion(ETS)/SK_N_MC-EWS:FLI1-ChIP-Seq(SRA014231)	1.00E-02	-4.69E+00
126	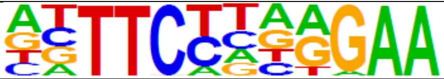	STAT6(Stat)/CD4-Stat6-ChIP-Seq(GSE22104)	1.00E-02	-4.63E+00

Table 1q continued

Rank	q-value (Benjamini)	# Target Sequences with Motif	% of Targets Sequences with Motif	# Background Sequences with Motif	% of Background Sequences with Motif
1	0	49	1.28%	2.7	0.30%
2	0	217	5.68%	25.4	2.85%
3	0	109	2.85%	9	1.01%
4	0	33	0.86%	1.8	0.20%
5	0	456	11.94%	71.3	8.01%
6	0	26	0.68%	0	0.00%
7	0	25	0.65%	1.2	0.13%
8	0	35	0.92%	2.5	0.28%
9	0	442	11.58%	75.8	8.51%
10	0	320	8.38%	51.8	5.82%
11	0	313	8.20%	50.3	5.65%
12	0	48	1.26%	4.5	0.50%
13	0	1198	31.38%	240.9	27.06%
14	0	93	2.44%	11.5	1.29%
15	0	412	10.79%	72.2	8.11%
16	0	351	9.19%	60.7	6.82%
17	0	21	0.55%	0	0.00%
18	0	370	9.69%	64.6	7.26%
19	0	59	1.55%	6.2	0.69%
20	0	118	3.09%	16.7	1.87%
21	0	600	15.72%	113.1	12.70%
22	0	188	4.92%	30	3.37%
23	0	70	1.83%	8.3	0.93%
24	0	401	10.50%	72.4	8.13%
25	0	1326	34.73%	274.8	30.86%
26	0	43	1.13%	4.1	0.46%
27	0	368	9.64%	66.2	7.43%
28	0	97	2.54%	13.9	1.56%
29	0	605	15.85%	116.5	13.08%
30	0	234	6.13%	39.3	4.41%
31	0	283	7.41%	49.2	5.53%

32	0	1218	31.90%	252.7	28.37%
33	0	41	1.07%	4.2	0.47%
34	0	1257	32.92%	262	29.42%
35	0	352	9.22%	64.7	7.27%
36	0	59	1.55%	7.3	0.82%
37	0	88	2.30%	12.1	1.35%
38	0	1631	42.72%	348	39.08%
39	0	287	7.52%	51.2	5.75%
40	0.0001	175	4.58%	30	3.36%
41	0.0001	613	16.06%	121.2	13.61%
42	0.0001	190	4.98%	32	3.60%
43	0.0001	16	0.42%	1.9	0.22%
44	0.0001	739	19.36%	149.9	16.84%
45	0.0001	44	1.15%	5.2	0.58%
46	0.0001	50	1.31%	6.2	0.70%
47	0.0002	116	3.04%	18.9	2.12%
48	0.0002	67	1.75%	9.1	1.02%
49	0.0002	78	2.04%	11.1	1.25%
50	0.0002	722	18.91%	146.5	16.45%
51	0.0002	898	23.52%	185.2	20.80%
52	0.0003	23	0.60%	2	0.23%
53	0.0003	359	9.40%	68.1	7.65%
54	0.0003	791	20.72%	162.7	18.27%
55	0.0003	15	0.39%	2	0.22%
56	0.0003	15	0.39%	1.9	0.21%
57	0.0004	48	1.26%	6.3	0.71%
58	0.0005	108	2.83%	17.5	1.96%
59	0.0005	371	9.72%	72	8.08%
60	0.0005	29	0.76%	3.6	0.40%
61	0.0007	303	7.94%	57.2	6.43%
62	0.0009	157	4.11%	27.8	3.12%
63	0.0009	1816	47.56%	397.3	44.61%
64	0.0011	116	3.04%	19.8	2.23%
65	0.0011	210	5.50%	38.4	4.31%
66	0.0014	40	1.05%	5.6	0.63%

67	0.0015	636	16.66%	130.1	14.61%
68	0.0015	21	0.55%	2.9	0.32%
69	0.0015	365	9.56%	71.2	7.99%
70	0.0016	494	12.94%	99.3	11.16%
71	0.0019	424	11.11%	84.6	9.51%
72	0.0021	124	3.25%	21.2	2.38%
73	0.0023	510	13.36%	103.3	11.60%
74	0.0026	192	5.03%	35.2	3.95%
75	0.0026	348	9.11%	68.9	7.73%
76	0.0027	357	9.35%	70.7	7.94%
77	0.0027	61	1.60%	9.3	1.04%
78	0.0027	61	1.60%	9	1.02%
79	0.0028	366	9.59%	72.2	8.11%
80	0.003	333	8.72%	65.4	7.34%
81	0.0031	162	4.24%	29.9	3.36%
82	0.0031	20	0.52%	2.4	0.27%
83	0.0033	248	6.50%	47.8	5.37%
84	0.0033	643	16.84%	133.4	14.98%
85	0.0037	195	5.11%	36.8	4.14%
86	0.0038	161	4.22%	29.5	3.31%
87	0.0042	242	6.34%	46.4	5.21%
88	0.0042	49	1.28%	7.4	0.83%
89	0.0042	49	1.28%	7.4	0.83%
90	0.0046	111	2.91%	19.9	2.24%
91	0.0049	743	19.46%	156.3	17.55%
92	0.005	698	18.28%	146.7	16.47%
93	0.0051	339	8.88%	67.5	7.58%
94	0.0051	325	8.51%	64.9	7.28%
95	0.0051	969	25.38%	207.7	23.33%
96	0.0054	467	12.23%	95.9	10.77%
97	0.0055	884	23.15%	188.5	21.17%
98	0.0057	125	3.27%	22.7	2.55%
99	0.0059	110	2.88%	19.9	2.23%
100	0.006	1231	32.24%	267	29.99%
101	0.0063	249	6.52%	48.9	5.49%

102	0.0068	12	0.31%	0.5	0.06%
103	0.0076	119	3.12%	21	2.36%
104	0.0094	585	15.32%	122.8	13.79%
105	0.011	98	2.57%	17.9	2.01%
106	0.0115	366	9.59%	74.1	8.33%
107	0.0124	175	4.58%	33.6	3.77%
108	0.0125	57	1.49%	9.7	1.08%
109	0.0126	347	9.09%	70.1	7.88%
110	0.0135	296	7.75%	59.1	6.64%
111	0.0137	107	2.80%	19.4	2.17%
112	0.0158	621	16.27%	131.5	14.77%
113	0.0179	11	0.29%	1.5	0.17%
114	0.0188	294	7.70%	59.1	6.63%
115	0.019	61	1.60%	10.8	1.22%
116	0.0198	86	2.25%	15.6	1.75%
117	0.0214	247	6.47%	49.2	5.52%
118	0.0252	195	5.11%	38.4	4.31%
119	0.0257	17	0.45%	3	0.33%
120	0.0273	75	1.96%	13.1	1.47%
121	0.0281	273	7.15%	55.8	6.26%
122	0.0287	868	22.73%	188.5	21.16%
123	0.0291	651	17.05%	139.3	15.64%
124	0.0296	400	10.48%	83.5	9.38%
125	0.0314	189	4.95%	37.7	4.23%
126	0.0332	170	4.45%	33	3.71%

r

Rank	Motif	Name	P-value	log P-pvalue
1	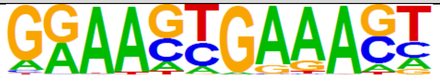	IRF8(IRF)/BMDM-IRF8-ChIP-Seq(GSE77884)	1.00E-04	-9.86E+00
2	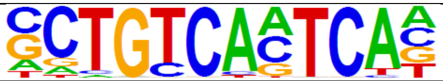	Pbx3(Homeobox)/GM12878-PBX3-ChIP-Seq(GSE32465)	1.00E-03	-7.31E+00
3	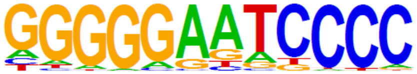	NFkB-p50,p52(RHD)/Monocyte-p50-ChIP-Chip(Schreiber_et_al.)	1.00E-03	-7.24E+00
4	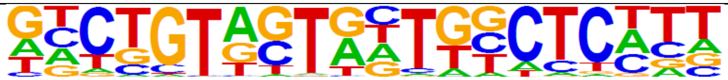	ZNF382(Zf)/HEK293-ZNF382.GFP-ChIP-Seq(GSE58341)	1.00E-03	-7.24E+00
5	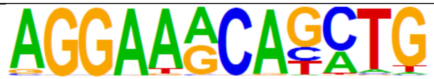	ETS:E-box(ETS,bHLH)/HPC7-Scl-ChIP-Seq(GSE22178)	1.00E-02	-6.87E+00
6	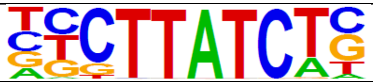	Gata2(Zf)/K562-GATA2-ChIP-Seq(GSE18829)	1.00E-02	-6.19E+00
7	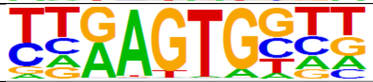	Bapx1(Homeobox)/VertebralCol-Bapx1-ChIP-Seq(GSE36672)	1.00E-02	-5.80E+00
8	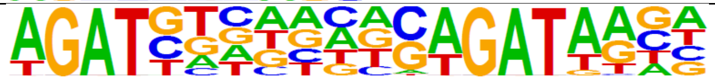	GATA3(Zf),DR8/iTreg-Gata3-ChIP-Seq(GSE20898)	1.00E-02	-5.32E+00
9	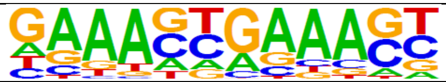	IRF2(IRF)/Erythroblas-IRF2-ChIP-Seq(GSE36985)	1.00E-02	-5.32E+00
10	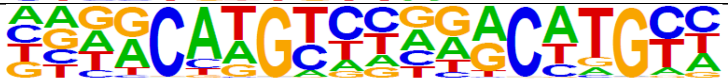	p63(p53)/Keratinocyte-p63-ChIP-Seq(GSE17611)	1.00E-02	-5.30E+00
11	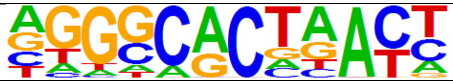	ZNF264(Zf)/HEK293-ZNF264.GFP-ChIP-Seq(GSE58341)	1.00E-02	-5.24E+00
12	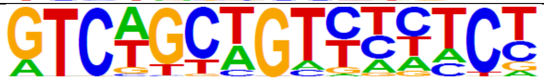	ZNF317(Zf)/HEK293-ZNF317.GFP-ChIP-Seq(GSE58341)	1.00E-02	-5.22E+00
13	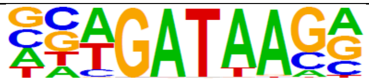	Gata4(Zf)/Heart-Gata4-ChIP-Seq(GSE35151)	1.00E-02	-5.12E+00
14	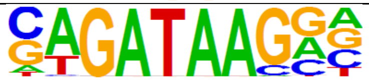	Gata1(Zf)/K562-GATA1-ChIP-Seq(GSE18829)	1.00E-02	-4.86E+00

15	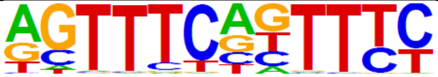	IRF3(IRF)/BMDM-Irf3-ChIP-Seq(GSE67343)	1.00E-02	-4.77E+00
16	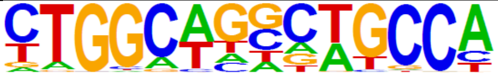	Tlx?(NR)/NPC-H3K4me1-ChIP-Seq(GSE16256)	1.00E-02	-4.66E+00
17	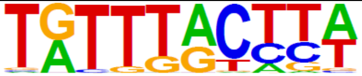	FOXM1(Forkhead)/MCF7-FOXM1-ChIP-Seq(GSE72977)	1.00E-02	-4.65E+00
18	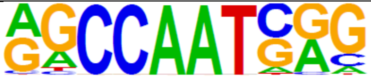	NFY(CCAAT)/Promoter	1.00E-02	-4.64E+00
19	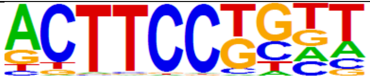	Elf4(ETS)/BMDM-Elf4-ChIP-Seq(GSE88699)	1.00E-02	-4.61E+00

Table 1r continued

Rank	q-value (Benjamini)	# Target Sequences with Motif	% of Targets Sequences with Motif	# Background Sequences with Motif	% of Background Sequences with Motif
1	0.0223	25	4.62%	27	1.96%
2	0.1428	18	3.33%	19.1	1.38%
3	0.1428	4	0.74%	0.8	0.06%
4	0.1428	4	0.74%	0.9	0.06%
5	0.1428	11	2.03%	9.2	0.67%
6	0.1468	48	8.87%	79.6	5.77%
7	0.185	174	32.16%	369.4	26.78%
8	0.234	9	1.66%	8.2	0.60%
9	0.234	9	1.66%	8.8	0.64%
10	0.234	25	4.62%	37	2.68%
11	0.234	34	6.28%	54.7	3.97%
12	0.234	6	1.11%	5	0.36%
13	0.234	67	12.38%	125.9	9.12%
14	0.2371	41	7.58%	70.2	5.09%
15	0.2424	20	3.70%	28.2	2.04%
16	0.2529	25	4.62%	38.7	2.80%
17	0.2529	72	13.31%	139.5	10.11%
18	0.2529	48	8.87%	86.7	6.28%
19	0.2529	53	9.80%	97.1	7.04%

s

Rank	Motif	Name	P-value	log P-pvalue
1	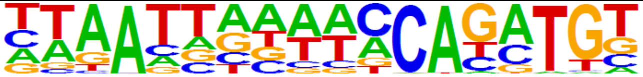	Pitx1:Ebox(Homeobox,bHLH)/Hindlimb-Pitx1-ChIP-Seq(GSE41591)	1.00E-09	-2.09E+01
2	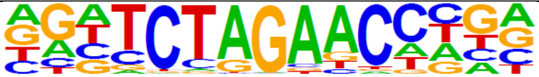	ZBTB12(Zf)/HEK293-ZBTB12.GFP-ChIP-Seq(GSE58341)	1.00E-08	-1.85E+01
3	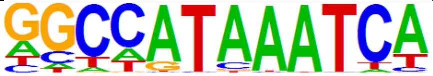	Hoxc9(Homeobox)/Ainv15-Hoxc9-ChIP-Seq(GSE21812)	1.00E-07	-1.78E+01
4	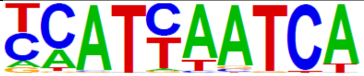	Pdx1(Homeobox)/Islet-Pdx1-ChIP-Seq(SRA008281)	1.00E-07	-1.77E+01
5	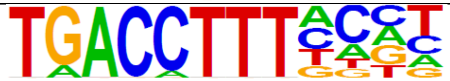	Nur77(NR)/K562-NR4A1-ChIP-Seq(GSE31363)	1.00E-07	-1.74E+01
6	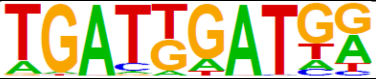	HOXA1(Homeobox)/mES-Hoxa1-ChIP-Seq(SRP084292)	1.00E-05	-1.31E+01
7	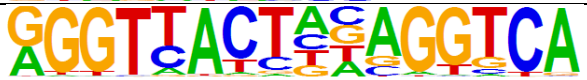	LXRE(NR),DR4/RAW-LXRb.biotin-ChIP-Seq(GSE21512)	1.00E-05	-1.23E+01
8	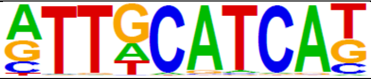	Chop(bZIP)/MEF-Chop-ChIP-Seq(GSE35681)	1.00E-04	-1.11E+01
9	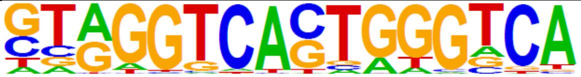	Reverb(NR),DR2/RAW-Reverba.biotin-ChIP-Seq(GSE45914)	1.00E-04	-1.09E+01
10	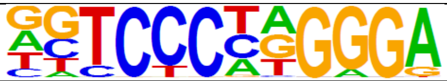	EBF(EBF)/proBcell-EBF-ChIP-Seq(GSE21978)	1.00E-04	-1.05E+01
11	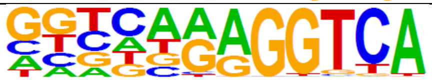	COUP-TFII(NR)/K562-NR2F1-ChIP-Seq(Encode)	1.00E-04	-1.04E+01
12	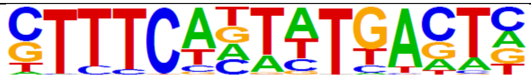	IRF:BATF(IRF:bZIP)/pDC-Irf8-ChIP-Seq(GSE66899)	1.00E-04	-9.28E+00
13	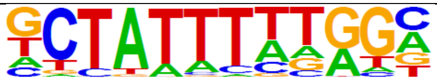	Mef2b(MADS)/HEK293-Mef2b.V5-ChIP-Seq(GSE67450)	1.00E-03	-9.14E+00

14	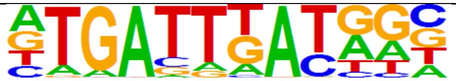	PBX2(Homeobox)/K562-PBX2-ChIP-Seq(Encode)	1.00E-03	-9.02E+00
15	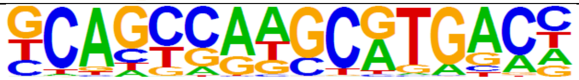	PAX5(Paired,Homeobox)/GM12878-PAX5-ChIP-Seq(GSE32465)	1.00E-03	-8.74E+00
16	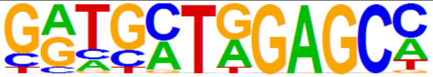	ZNF415(Zf)/HEK293-ZNF415.GFP-ChIP-Seq(GSE58341)	1.00E-03	-8.29E+00
17	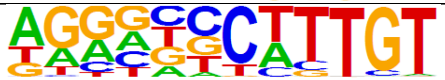	Sox9(HMG)/Limb-SOX9-ChIP-Seq(GSE73225)	1.00E-03	-8.23E+00
18	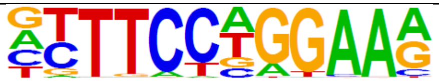	STAT4(Stat)/CD4-Stat4-ChIP-Seq(GSE22104)	1.00E-03	-8.23E+00
19	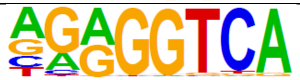	COUP-TFII(NR)/Artia-Nr2f2-ChIP-Seq(GSE46497)	1.00E-03	-8.19E+00
20	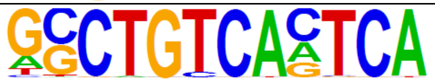	PBX1(Homeobox)/MCF7-PBX1-ChIP-Seq(GSE28007)	1.00E-03	-7.97E+00
21	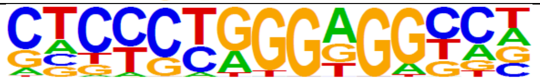	GLIS3(Zf)/Thyroid-Glis3.GFP-ChIP-Seq(GSE103297)	1.00E-03	-7.97E+00
22	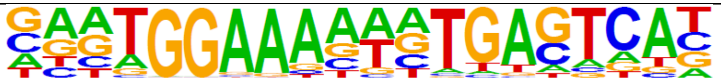	NFAT:AP1(RHD,bZIP)/Jurkat-NFATC1-ChIP-Seq(Jolma_et_al.)	1.00E-03	-7.91E+00
23	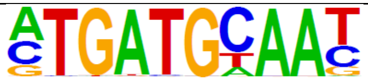	Atf4(bZIP)/MEF-Atf4-ChIP-Seq(GSE35681)	1.00E-03	-7.61E+00
24	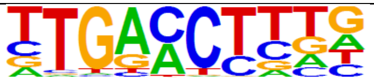	RARa(NR)/K562-RARa-ChIP-Seq(Encode)	1.00E-03	-7.57E+00
25	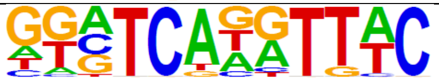	Six1(Homeobox)/Myoblast-Six1-ChIP-Chip(GSE20150)	1.00E-03	-7.38E+00
26	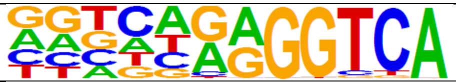	EAR2(NR)/K562-NR2F6-ChIP-Seq(Encode)	1.00E-03	-7.20E+00
27	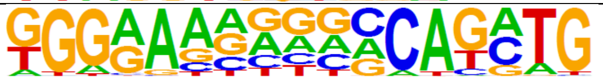	RBPJ:Ebox(?,bHLH)/Panc1-Rbpj1-ChIP-Seq(GSE47459)	1.00E-02	-6.70E+00
28	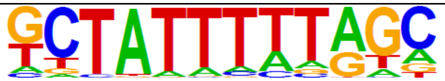	Mef2d(MADS)/Retina-Mef2d-ChIP-Seq(GSE61391)	1.00E-02	-6.68E+00

29	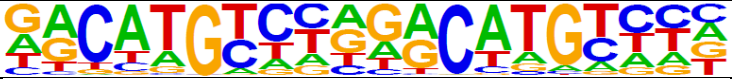	p53(p53)/Saos-p53-ChIP-Seq(GSE15780)	1.00E-02	-6.58E+00
30	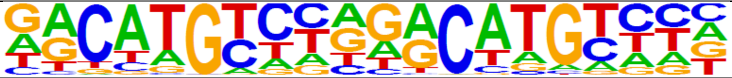	p53(p53)/Saos-p53-ChIP-Seq	1.00E-02	-6.58E+00
31	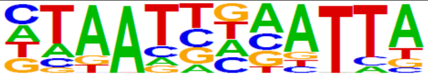	Prop1(Homeobox)/GHFT1-PROP1.biotin-ChIP-Seq(GSE77302)	1.00E-02	-6.49E+00
32	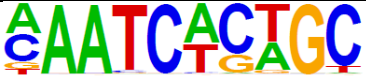	Gfi1b(Zf)/HPC7-Gfi1b-ChIP-Seq(GSE22178)	1.00E-02	-6.47E+00
33	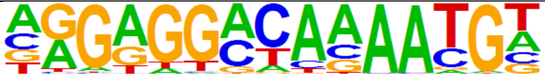	ZNF675(Zf)/HEK293-ZNF675.GFP-ChIP-Seq(GSE58341)	1.00E-02	-6.39E+00
34	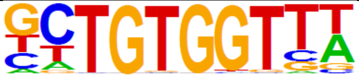	RUNX-AML(Runt)/CD4+-PolII-ChIP-Seq(Barski_et_al.)	1.00E-02	-6.39E+00
35	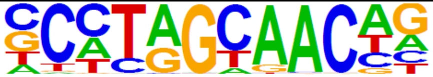	Rfx5(HTH)/GM12878-Rfx5-ChIP-Seq(GSE31477)	1.00E-02	-6.11E+00
36	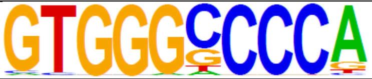	ZNF692(Zf)/HEK293-ZNF692.GFP-ChIP-Seq(GSE58341)	1.00E-02	-5.96E+00
37	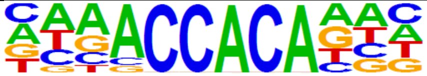	RUNX2(Runt)/PCa-RUNX2-ChIP-Seq(GSE33889)	1.00E-02	-5.86E+00
38	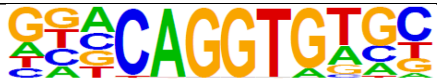	ZEB2(Zf)/SNU398-ZEB2-ChIP-Seq(GSE103048)	1.00E-02	-5.83E+00
39	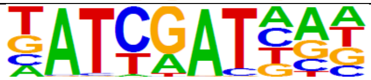	CUX1(Homeobox)/K562-CUX1-ChIP-Seq(GSE92882)	1.00E-02	-5.81E+00
40	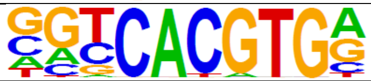	USF1(bHLH)/GM12878-Usf1-ChIP-Seq(GSE32465)	1.00E-02	-5.56E+00
41	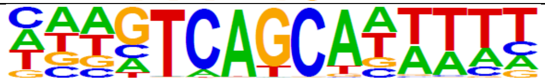	MafF(bZIP)/HepG2-MafF-ChIP-Seq(GSE31477)	1.00E-02	-5.39E+00
42	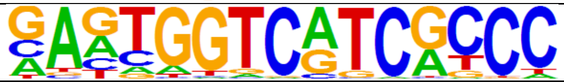	ZNF669(Zf)/HEK293-ZNF669.GFP-ChIP-Seq(GSE58341)	1.00E-02	-5.29E+00
43	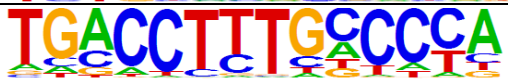	PPARE(NR),DR1/3T3L1-Pparg-ChIP-Seq(GSE13511)	1.00E-02	-5.29E+00

44	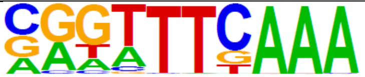	CHR(?)/Hela-CellCycle-Expression	1.00E-02	-5.20E+00
45	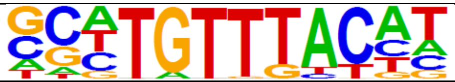	FOXK2(Forkhead)/U2OS-FOXK2-ChIP-Seq(E-MTAB-2204)	1.00E-02	-5.16E+00
46	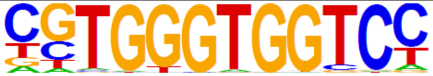	GLI3(Zf)/Limb-GLI3-ChIP-Chip(GSE11077)	1.00E-02	-5.13E+00
47	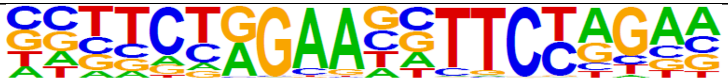	HRE(HSF)/HepG2-HSF1-ChIP-Seq(GSE31477)	1.00E-02	-5.13E+00
48	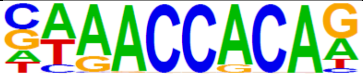	RUNX(Runt)/HPC7-Runx1-ChIP-Seq(GSE22178)	1.00E-02	-5.03E+00
49	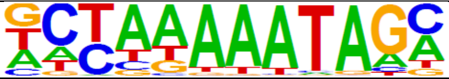	Mef2c(MADS)/GM12878-Mef2c-ChIP-Seq(GSE32465)	1.00E-02	-5.01E+00
50	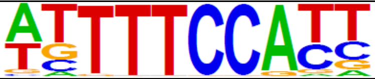	NFAT(RHD)/Jurkat-NFATC1-ChIP-Seq(Jolma_et_al.)	1.00E-02	-4.67E+00
51	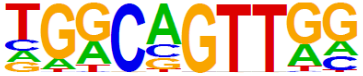	AMYB(HTH)/Testes-AMYB-ChIP-Seq(GSE44588)	1.00E-02	-4.65E+00

Table 1s continued

Rank	q-value (Benjamini)	# Target Sequences with Motif	% of Targets Sequences with Motif	# Background Sequences with Motif	% of Background Sequences with Motif
1	0	30	2.16%	3.1	0.57%
2	0	63	4.54%	11.8	2.19%
3	0	95	6.84%	20.3	3.75%
4	0	183	13.17%	47.1	8.74%
5	0	22	1.58%	2.7	0.49%
6	0.0001	37	2.66%	6.9	1.27%
7	0.0003	32	2.30%	5.2	0.97%
8	0.0008	46	3.31%	9.6	1.77%
9	0.0009	22	1.58%	3.7	0.68%
10	0.0012	17	1.22%	2.9	0.53%
11	0.0012	230	16.56%	69.5	12.88%
12	0.0033	16	1.15%	2.5	0.46%
13	0.0036	171	12.31%	51	9.45%
14	0.0037	153	11.02%	44.3	8.21%
15	0.0046	53	3.82%	12.8	2.38%
16	0.0067	75	5.40%	19.6	3.62%
17	0.0067	112	8.06%	31.2	5.79%
18	0.0067	130	9.36%	37.4	6.92%
19	0.0067	248	17.85%	78.2	14.50%
20	0.0074	10	0.72%	0	0.00%
21	0.0074	250	18.00%	79.5	14.74%
22	0.0074	27	1.94%	5.4	1.00%
23	0.0092	51	3.67%	12.7	2.35%
24	0.0092	480	34.56%	164.2	30.44%
25	0.0107	30	2.16%	6.7	1.24%
26	0.0123	202	14.54%	63.7	11.80%
27	0.0194	36	2.59%	8.2	1.52%
28	0.0194	29	2.09%	6.9	1.27%
29	0.0197	9	0.65%	1.6	0.29%
30	0.0197	9	0.65%	1.6	0.29%
31	0.0209	119	8.57%	35.3	6.54%

32	0.0209	116	8.35%	34.3	6.35%
33	0.0219	25	1.80%	5.1	0.95%
34	0.0219	95	6.84%	27.4	5.08%
35	0.0273	35	2.52%	9	1.66%
36	0.0307	17	1.22%	3.9	0.73%
37	0.0331	117	8.42%	35.1	6.51%
38	0.0331	160	11.52%	50.3	9.32%
39	0.0331	111	7.99%	33.4	6.20%
40	0.0411	47	3.38%	12.9	2.40%
41	0.0477	56	4.03%	15.2	2.82%
42	0.0512	8	0.58%	1.8	0.34%
43	0.0512	118	8.50%	36.2	6.71%
44	0.0539	109	7.85%	33.1	6.14%
45	0.0544	106	7.63%	32.3	5.98%
46	0.0544	16	1.15%	3.6	0.67%
47	0.0544	16	1.15%	3.7	0.68%
48	0.0582	91	6.55%	27.1	5.02%
49	0.0584	117	8.42%	36.5	6.77%
50	0.0802	130	9.36%	41.4	7.66%
51	0.0802	197	14.18%	65	12.05%
